# Inhibition of hepatocellular carcinoma by metabolic normalization

**DOI:** 10.1371/journal.pone.0218186

**Published:** 2019-06-26

**Authors:** Huabo Wang, Jie Lu, James Dolezal, Sucheta Kulkarni, Weiqi Zhang, Angel Chen, Joanna Gorka, Jordan A. Mandel, Edward V. Prochownik

**Affiliations:** 1 Section of Hematology/Oncology, Children’s Hospital of Pittsburgh of UPMC, Pittsburgh, Pennsylvania, United States of America; 2 Tsinghua University School of Medicine, Beijing, People’s Republic of China; 3 The Department of Microbiology and Molecular Genetics, The University of Pittsburgh School of Medicine, Pittsburgh, Pennsylvania, United States of America; 4 The Hillman Cancer Center, The University of Pittsburgh Medical Center, Pittsburgh, Pennsylvania, United States of America; 5 The University of Pittsburgh Liver Research Center, The University of Pittsburgh Medical Center, Pittsburgh, Pennsylvania, United States of America; University of Cordoba, SPAIN

## Abstract

In two different mouse liver cancer models, we recently showed that a switch from oxidative phosphorylation (Oxphos) to glycolysis (the Warburg effect) is invariably accompanied by a marked decline in fatty acid oxidation (FAO) and a reciprocal increase in the activity of pyruvate dehydrogenase (PDH), which links glycolysis to the TCA cycle. We now show that short-term implementation of either medium-chain (MC) or long-chain (LC) high fat diets (HFDs) nearly doubled the survival of mice with c-Myc oncoprotein-driven hepatocellular carcinoma (HCC). Mechanistically, HFDs forced tumors to become more reliant on fatty acids as an energy source, thus normalizing both FAO and PDH activities. More generally, both MC- and LC-HFDs partially or completely normalized the expression of 682 tumor-dysregulated transcripts, a substantial fraction of which are involved in cell cycle control, proliferation and metabolism. That these same transcripts were responsive to HFDs in livers strongly suggested that the changes were the cause of tumor inhibition rather than its consequence. In seven different human cancer cohorts, patients with tumors containing high ratios of FAO-related:glycolysis-related transcripts had prolonged survival relative to those with low ratios. Furthermore, in 13 human cancer types, the expression *patterns* of transcripts encoding enzymes participating in FAO and/or cholesterol biosynthesis also correlated with significantly prolonged survival. Collectively, our results support the idea that the survival benefits of HFDs are due to a reversal of the Warburg effect and other tumor-associated metabolic and cell cycle abnormalities. They also suggest that short-term dietary manipulation, either alone or in combination with more traditional chemotherapeutic regimens, might be employed as a relatively non-toxic and cost-effective means of enhancing survival in certain cancer types.

## Introduction

Metabolic re-programming is one of the “hallmark” features of cancer [1]. Numerous tumor-associated metabolic phenotypes have been documented and range from quantitative alterations in the activities of normal pathways such as glycolysis, fatty acid metabolism and glutaminolysis to mutations in metabolic enzymes that generate so-called onco-metabolites, drive epigenetic modifications and alter gene expression [2–4]. These changes are diverse, complex, dynamic and may differ among histologically identical tumors or even within different regions of the same tumor [5, 6]. Their common purpose is to participate in the direct provision and/or synthesis of anabolic precursors to cancer cells and to generate ATP, thus optimizing growth and survival under otherwise inimical intratumoral conditions characterized by hypoxia, acidosis and/or nutrient deprivation [7]. However, even when these barriers are otherwise overcome, the now unrestrained proliferative signals emanating from oncogenic signaling must still be balanced by concomitant changes in anabolic activities, redox state and energy supplies to sustain macromolecular precursor synthesis and assembly, biomass accretion and cell division. The targeting of tumor metabolism as a therapeutic strategy has thus garnered considerable interest while accompanied by variable degrees of success. Limitations of this approach include the often narrow therapeutic window afforded by the differential metabolism of normal and transformed cells and by the tendency of the latter to circumvent pharmacologic blocks of individual enzymes in metabolic pathways [8–13].

Among the most well-known and versatile of cancer-associated metabolic changes is the “Warburg effect” whereby the normally anaerobic process of glycolysis continues to function at a high rate despite sufficient oxygen to enable oxidative phosphorylation (Oxphos) [4, 13–16]. This ensures that glycolytic intermediates, rather than being channeled directly into the TCA cycle are instead variably diverted into anabolic pathways so as to provide macromolecular precursors such as pentose sugars, nucleotides and amino acids. The energetically wasteful lactate dehydrogenase (LDH) reaction that accompanies the Warburg effect and generates lactate from glycolytically-derived pyruvate provides a critical source of NAD+, an obligate electron acceptor required to sustain upstream glycolytic reactions.

A major alternate source of AcCoA during periods of glycolytic compromise is fatty acid β-oxidation (FAO). In some normal tissues, such as the liver, glycolysis and FAO are mutually inhibitory. This negative feedback process, termed the Randle cycle or the glucose-fatty acid cycle, controls fuel selection and balances the supply of and demand for energy-generating substrates in coordination with insulin signaling. The increased AcCoA:CoA and NADH:NAD^+^ ratios that accompany high rates of FAO inhibit glycolysis primarily at the level of the mitochondrial enzyme complex pyruvate dehydrogenase (PDH) and to a lesser extent at the level of phosphofruktokinase (PFK) [15, 16].

We have previously encountered this behavior in two mouse models of hepatoblastoma (HB) and hepatocellular carcinoma (HCC). In both tumor types, FAO is markedly reduced relative to normal liver whereas the activity of PDH is increased [17–19]. While not considered a glycolytic enzyme, PDH nonetheless links glycolysis and the TCA cycle by virtue of its catalysis of pyruvate to AcCoA. PDH is tightly controlled by the inhibitory PDH kinase PDK1, the stimulatory kinase PDP2 and by certain small molecules. The latter include fatty acids and the ratios of ATP:ADP, NADH:NAD+ and AcCoA:CoA which affect the activities of all three enzymes [16, 20–22]. We have attributed the high tumor PDH activity of tumors to changes in the abundance of these factors and/or to reduced pyruvate levels [17–19, 23].

The HCC model relies on the conditional, hepatocyte-specific over-expression of the c-Myc (Myc) oncoprotein to drive tumorigenesis, leading to the demise of virtually all animals within 25–30 days [17, 24, 25]. Because Myc up-regulates most glycolytic genes, enhances glucose uptake and stimulates Warburg-type respiration [26, 27], we were interested in determining whether forced metabolic “normalization” via manipulation of the Randle cycle affected survival and in defining the underlying mechanism by which this was achieved. We report here that tumor-bearing mice maintained on either medium-chain or long-chain high-fat diets (MC-HFDs and LC-HFDs, respectively) survived nearly twice as long as those provided with standard, normal fat content diets (NFDs). Along with other metabolic alterations, tumors from HFD groups assumed a more liver-like metabolic profile marked by higher rates of FAO and decreased PDH activity. We further identified a core set of transcripts that were specifically and similarly altered by the combination of transformation and either of the two HFDs. Finally, in large cohorts of multiple human cancer types, high ratios of FAO-:glycolysis-related transcripts and/or certain expression patterns of transcripts involved in FAO or cholesterol biosynthesis were associated with superior long-term survival.

## Materials and methods

### Animals, induction of HCCs and preparation and storage of tissues

All animal work was conducted in accord with the Public Health Service Policy on Humane Care and Use of Laboratory Animal Research (DLAR) Guide for Care and Use of Laboratory Animals. All procedures were approved by The University of Pittsburgh’s Institutional Animal Care and Use Committee (IACUC). FVB/N-Tg(tetO-MYC)36aBop/J and LAP-tTA mice (B6.Cg-Tg[Cebpb-tTA]5Bjd/J) (Jackson Laboratories, Bar Harbor, ME) were genotyped as previously described [17, 25] and were maintained in a pathogen-free facility with *ad libitum* access to food and water which, unless otherwise stated, contained doxycycline (Dox) (100 μg/ml). HCCs were induced by discontinuing Dox and thereby inducing high-level expression of human Myc. MC-HFDs and LC-HFDs were purchased from Research Diets, Inc. (New Brunswick, NJ) and were comprised of 45 kcal% LC or MC fatty acids. In the former case, these consisted primarily of palmitic acid, palmitoleic acid, oleic acid, steric acid and linoleic acid whereas in the latter case the primary source of fatty acid was MC triglyceride (MCT) Oil. At 6–8 wks of age, appropriate cohorts of mice, containing equal numbers of males and females, were switched from standard NFDs to HFDs one wk before the inductions of HCCs. HFDs were maintained throughout the duration of the study, defined as the time when tumors reached 2 cm in diameter or animals showed signs of weight loss, hunching or other obvious stress. For some studies, mice maintained on Dox and without tumors but otherwise provided the same diets were used as sources of control liver tissues. At the time of sacrifice, tumors or livers were weighed and maintained on ice. Tissues were then apportioned for immediate use in assays as described below and elsewhere [17–19]. Remaining small aliquots of tissue were immediately snap frozen in liquid nitrogen and stored at -80C for latter use.

### Immuno-blotting

Frozen tissues were re-suspended in 1 x SDS-PAGE buffer containing protease and phosphatase inhibitors and disrupted using a bullet blender (Next Advance, Inc., Troy, NY) [17–19, 28]. After adjusting sample volumes to account for differences in total protein content, adding β-mercaptoethanol and boiling for 5 min, samples were stored at -80C. SDS-PAGE, electro-transfer of protein to PVDF membranes and probing with antibodies were all performed as previously described [17, 25, 28, 29]. Antibodies used, vendors from which they were purchased and conditions are detailed in S1 Table. Where necessary, immuno-blot band intensities were quantified and averaged across multiple blots using a Protein Simple FluorChem M instrument according to the methods and instructions provided by the vendor (Protein Simple, Inc. San Jose, CA).

### Enzyme and triglyceride assays and oxygen consumption assays

All assays were performed as previously described [17–19, 28]. Those for PFK were performed on previously frozen tissue samples using a 96-well plate assay kit according to the directions of the vendor (MyBioSource, San Diego, CA). Triglyceride levels were also determined on frozen tissue samples as previously described [19, 30]. FAO assays were performed on isolated mitochondria by measuring the release of water-soluble products from ^3^H-labeled palmitate-BSA [17–19, 28].

Oxygen consumption rates (OCRs) of disrupted tissues were performed as previously described [17, 18]. Briefly, ~40–50 mg of finely minced tissue was suspended in 2 ml of Mir05 buffer containing 10 μM cytochrome *c*, 5 mM malate and 5 mM ADP. Pyruvate was then added (5 mM final concentration) with the change in OCR being used as a measure of PDH activity. This was found to be in good agreement with previous assays using minced tissues, which measured the release of ^14^CO_2_ following the addition of ^14^C-pyruvate [17–19]. Glutaminolysis was then assessed by the addition of glutamate (10 mM final concentration). After achieving plateau OCR, which assessed the maximal activity of Complex I, succinate was added (10 mM final concentration) to determine the additional maximal contribution of Complex II. 0.5 μM rotenone was then added to inhibit Complex I and allow the proportional contribution of Complex II to be verified. As previously described for both livers and tumors [17–19, 25], Complex II comprised ~80% of ETC activity. All activities were normalized to total protein.

### RNA purification, RNAseq and analytic methods

Total RNAs were extracted from randomly selected samples of previously snap-frozen livers and tumors from each of the groups using RNA Easy columns (Qiagen, Inc., Valencia, CA). RNA quantification was performed with a Nanodrop ND-1000 instrument (NanoDrop Technologies Inc., Wilmington, DE, USA) and RNA integrity was measured with an Agilent 2100 Bioanalyzer (Agilent Technologies, Santa Clara, CA). Only samples with RIN values >8.0 were processed further. RNAseq was performed by the CHP of UPMC Core Genomics Facility using paired-end single-indexed sequencing on an Illumina NextSeq 500 sequencer (Illumina, Inc., San Diego, CA) essentially as previously described [11, 17]. Read counts were normalized among samples and significance was assessed by DESeq, which quantified transcript abundance (expressed as FPKM) and statistical significance when comparing transcript abundance levels of different groups (Bonferroni FDR-adjusted q value <0.05). Heatmaps were generated by comparing expression values in each experimental group to control livers or tumors maintained on standard diets and were generally expressed as log_2_-transformed fold-change. Where appropriate, pathway analyses were performed with Ingenuity Pathway Analysis (IPA) software (IPA) (www.qiagen.com/ingenuity). All RNAseq data were deposited in the Gene Expression Omnibus (GEO) data base (https://www.ncbi.nlm.nih.gov/geo/query/acc.cgi?acc=GSE116463).

To analyze gene transcripts involved in cholesterol biosynthesis, FAO and glycolysis in human tumor samples, expression levels and survival statistics were downloaded from the GDC-TCGA data base using the UCSC Xenabrowser (https://xenabrowser.net). The former data included RNAseq (HTSeq-FPKM) data for the genes involved in each pathway. Data were filtered to contain only primary tumor samples and, if appropriate, matched adjacent normal tissue, and further filtered for complete survival data. Heat maps showing expression levels for the genes in each tumor and normal tissue sample were generated using Microsoft Excel (Microsoft Corporation, Redmond, WA), where color intensity values were determined using the base-two logarithm of the ratio of each FPKM value (transformed from the initial incremented, log_2_ transformed state) to the average of all FPKM values across each sample. For transcripts related to glycolysis, cholesterol biosynthesis and FAO and glycolysis (S1 and S2 Figs), the average of the incremented log_2_ transformed expression value of each transcript was averaged across each primary tumor sample, generating a score for each. Tumors were classified as having high or low expression based on whether the score for each group was respectively above or below the median value for the group. Survival difference among groups were assessed using a log-rank test. FPKM-UQ values were also normalized to the sum of FPKM-UQ values across each gene in their respective sample and visualized via t-SNE analysis [31] using TensorFlow r1.0 and Tensorboard (https://tensorflow.org), with learning rate and perplexity parameter values annotated on the output projections with iterations sufficient for the visualization to stabilize (n~10,000). The significance of survival differences between the patients whose tumors fell into the resulting clusters was assessed using log-rank tests. For all survival analyses, the time variable for the survival curves was taken as the maximum of the “days to death” or the “days to last follow-up”, and data were censored in cases where a “days to death” value was not provided. The cholesterol biosynthesis genes included in these analyses included all those depicted in S1 Fig.

To analyze transcripts involved in FAO and glycolysis (S1 and S2 Figs), expression levels and survival statistics from patients were also downloaded from the GDC Pan-Cancer (PANCAN) dataset as described above. Data were filtered to contain only primary tumor samples. The expression levels were averaged in their original incremented, log_2_-transformed state, resulting in an FAO score and a glycolysis score for each sample. The groups of interest for survival analysis were samples with concurrent above-median glycolysis and below-median FAO scores, and samples with concurrent below-median glycolysis and above-median FAO scores. The time variable for survival was taken as the maximum of either the “days to death” or “days to last follow-up”, and data were censored in cases where no “days to death” value was provided. Significance of survival differences between the groups of interest was assessed using log-rank tests. All log-rank tests and associated Kaplan-Meier plots were executed in GraphPad Prism 7 (GraphPad Software, La Jolla, CA).

## Results

### Short-term HFDs prolong survival of HCC-bearing mice and force partial metabolic normalization

**Fig 1:**
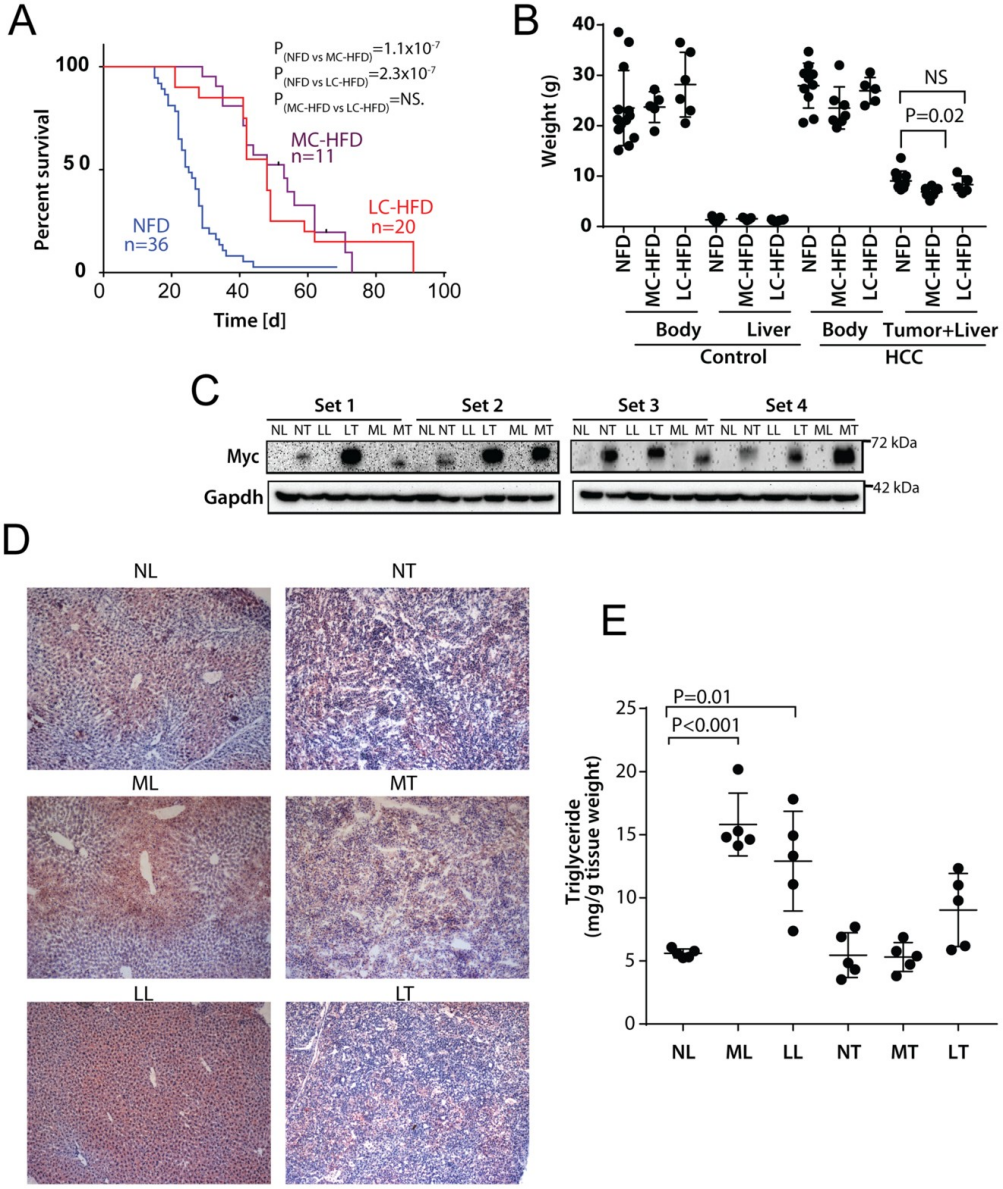
HFDs extend lifespans of HCC-bearing mice. **A**, Kaplan-Meier survival curves. NFDs, MC-HFDs or LC-HFDs were initiated one wk prior to Myc induction. Median survival times for each group were: NFD = 25+/-1.7 d, MC-HFD = 53+/-6.3 d, LC-HFD = 48+/-4.2 d. P values were determined by a log-rank comparison between each of the indicated groups. **B**. Total body, liver and tumor weights at the time of sacrifice. The control groups were comprised of non-tumor-bearing mice maintained on Dox and provided with the indicated diets for three months. **C**. Representative Myc protein levels in four sets of livers (L) and tumors (T) maintained on NFDs (NL or NT), MC-HFDs (ML or MT) or LC-HFDs (LL or LT). **D**, Representative liver and tumor sections stained with H&E and Oil Red O. **E**, Triglyceride content of livers and tumors maintained on the indicated diets.

Mice bearing a human Myc transgene driven by the hepatocyte-specific, doxycycline-suppressible LAP-promoter [19, 24], were placed on NFDs, MC-HFDs or LC-HFDs one week prior to Myc induction and maintained on these continuously during the course of HCC development. The latter two groups showed significantly prolonged survival (Fig 1A). Total body weights did not vary significantly among the three groups during this time, most likely because of the relatively short duration of HFD exposure (Fig 1B). Despite the longer survival of LC-HFD-maintained mice, their tumor weights were nearly identical to those from HFD mice and tumors from MC-HFD mice were actually somewhat smaller (Fig 1B). Thus, despite their significantly longer survival, mice from both HFD groups harbored HCCs that, at the time of sacrifice, were no larger than those from the NFD group. Myc protein, barely observable in livers, was readily detected in all tumor groups indicating that HFDs did not suppress transgene expression [19, 24] (Fig 1C). Histologic examination, Oil Red O staining and quantification of triglyceride levels showed increased lipid stores in livers from non-tumor-bearing mice maintained on both HFDs but not in the corresponding tumors from similarly maintained mice (Fig 1D and 1E).

**Fig 2:**
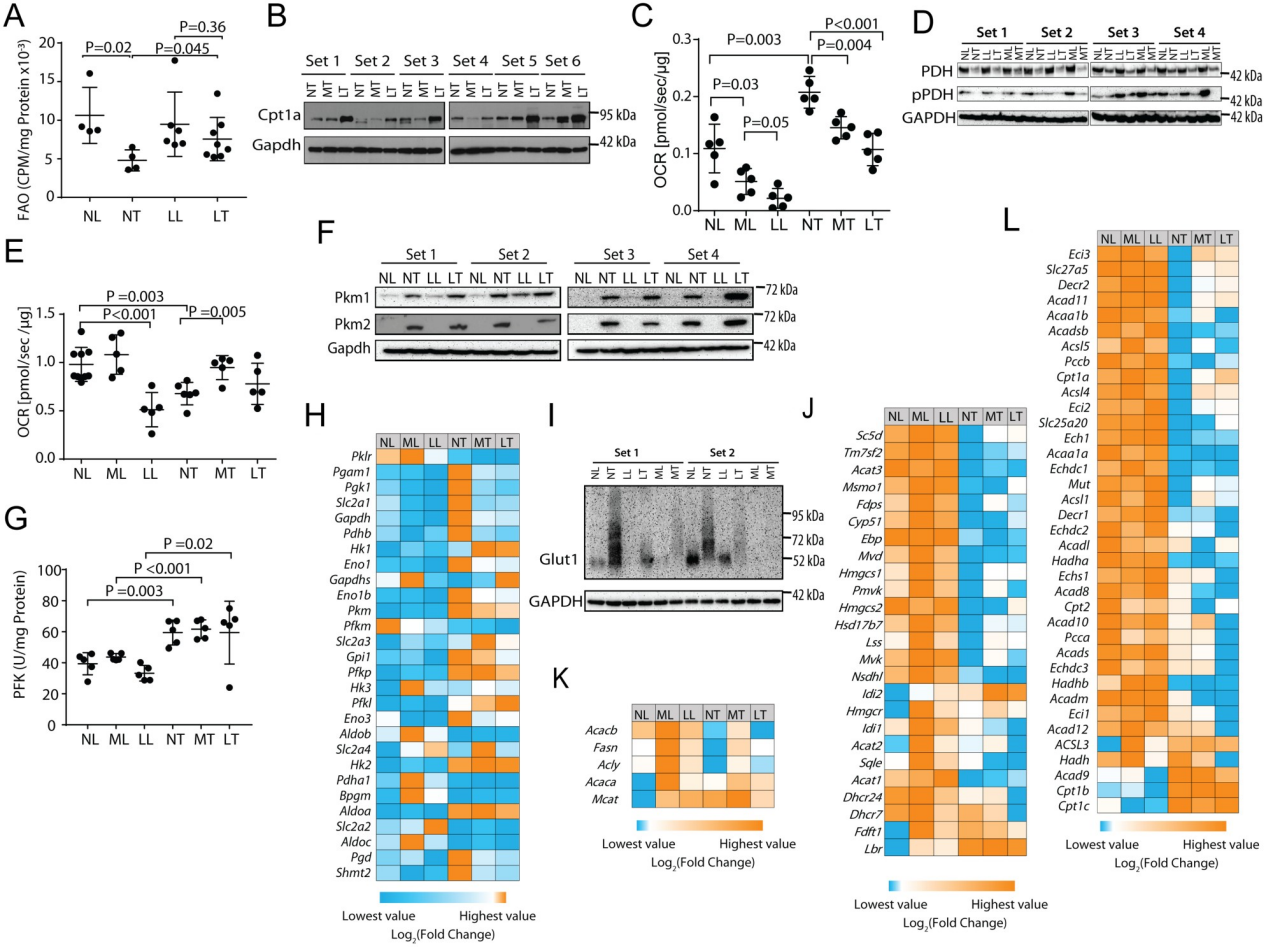
HFDs force metabolic re-programming. **A**, FAO by isolated mitochondria. Mitochondria from the indicated tissues were incubated with ^3^H-palmitate-BSA and the release of water-soluble products was quantified. **B**, Cpt1a immuno-blots showing increased expression of Cpt1a in tumors from mice maintained on LC-HFDs but not on MC-HFDs (P = 0.025). **C**, PDH activities. OCRs were quantified following the addition of malate, ADP and pyruvate to isolated mitochondria. **D**, PDH and pPDH immuno-blots. No significant differences among the different tumor groups were noted. **E**, OCRs in response to the TCA substrates pyruvate, malate, glutamate and succinate in each of the six tissue groups [17–19]. **F**, Immuno-blots of pyruvate kinase isoforms, PKM1 and PKM2 in the indicated tissues. No significant differences among the different tumor groups were noted. **G**, PFK activities were quantified on extracts from the indicated tissues. Each point represents the mean of duplicate assays of the same tissue. **H**, Heat map for transcripts encoding key glycolytic enzymes as well as the glucose transporter Slc2a1/GLUT1 and the rate-limiting enzymes 6Pgd and Shmt2. Each column depicts the mean expression of samples obtained from five randomly chosen mice. See S1A Fig for quantification of transcript levels. **I**, Immuno-blots for Slc2a1/GLUT1 in the indicated tissues. **J**, Expression of transcripts encoding cholesterol biosynthetic enzymes. See S1B Fig for the proper order of these enzymes along the pathway and S1C Fig for actual expression levels of each transcript across all tissues. **K**, Heat map for transcripts encoding key enzymes in the FAS pathway. These include malonyl-CoA-acyl carrier protein transacylase (Mcat), AcCoA carboxylase a&b (Acaca & Acacb), ATP citrate lyase (acly) and fatty acid synthase (Fasn). See S2A Fig for actual quantification of each transcript across all tissues. **L**, Heat map for transcripts encoding key enzymes in the FAO pathway. See S2B & S2C Fig for each transcript’s position in the FAO pathway and its actual quantification across all tissues, respectively. For panels H,J,K and L, each column represents the mean values obtained from five tissues.

FAO down-regulation and PDH up-regulation are features of HCCs in this animal model as well as in another model of HB [17–19, 25]. Standard FAO assays, typically performed with minced tissues, could not be conducted in the current study due to variations in the amounts of stored lipids (Fig 1D & 1E), which competed with the ^3^H-labeled palmitate substrate. We therefore performed our assay with isolated mitochondria. As previously shown in whole liver [17–19, 25], mitochondria from NFD-tumors had significantly lower FAO rates than NFD-livers (Fig 2A). In contrast, FAO rates of LC-HFD tumors more closely resembled those of NFD-livers. Thus, HFDs forced the preferential utilization of fats as an energy source and partially normalized this aspect of tumor metabolic re-programming.

Unlike the oxidation of MC or short-chain fatty acids, which freely enter mitochondria, LC fatty acids must be actively transported across the mitochondrial membrane by carnitine palmitoyltransferase Ia (Cpt1a), the liver-specific isoform of Cpt1 and the rate-limiting enzyme in the FAO pathway [32]. In keeping with the above-discussed findings, LC-HFD tumors but not MC-HFD tumors up-regulated Cpt1a by an average of 2.0-fold (P = 0.025), consistent with the results shown in Fig 2A and indicating that LC fatty acid transport into oxidative pathways was an active process (Fig 2B).

Further confirming previous findings [17–19, 25] was the significant increase in the OCR of NFD tumors in response to pyruvate (Fig 2C). Along with the results shown in Fig 2A and 2B, this is consistent with the notion that Myc-driven HCC metabolic re-programming involves increased reliance on glucose oxidation at the expense of FAO. In contrast, both livers and tumors from LC-HFD- and MC-HFD-maintained mice showed a pronounced dampening of the response to pyruvate that again correlated inversely with FAO activity. Although total PDH protein levels declined by 60% in all tumors relative to livers as previously reported [17] (P<0.001), PDHE1 Ser_293_-phosphorylation declined somewhat more (~70%, P<0.001) and was indistinguishable among the different tumor groups (Fig 2D). These findings strongly suggested that changes in pyruvate flux were more likely due to direct small-molecule inhibition of the PDH complex itself rather than to its post-translational modification [15, 16, 21].

HFDs also re-programmed OCRs in response to the TCA substrates pyruvate, malate and succinate as well as glutamate although in distinct ways (Fig 2E). For example, LC-HFDs suppressed OCRs in liver mitochondria whereas the MC-HFDs had no significant effect. In NFD-tumors, OCRs were significantly reduced as previously reported [17–19], whereas the response was normalized in both HFD groups. All OCRs were inhibited by <10% in response to rotenone, indicating that, regardless of diets, the vast majority of the OCR in both livers and HCCs was driven by Complex II (not shown) [17–19]. These studies provide additional evidence that HFDs reprogram tumor mitochondria to allow for a more efficient use of standard TCA cycle substrates, while simultaneously reducing the influx of glycolytically-derived pyruvate (Fig 2C).

To better localize HFD-responsive nodes, we examined two additional, highly regulated glycolytic enzymes, pyruvate kinase (PK) and PFK. PK catalyzes the conversion of phosphoenol pyruvate (PEP) to pyruvate and exists in two isoforms, PKM1 and PKM2, as a result of alternate mRNA splicing [33]. PKM1 predominates in most quiescent tissues whereas PKM2 is more often expressed by rapidly dividing normal and cancer cells. PKM2’s higher K_m_ for phosphoenol pyruvate may facilitate the accumulation of upstream glycolytic intermediates thus enhancing their diversion into anabolic pathways [23, 33]. Although both PKM1 and PKM2 proteins were equally up-regulated in tumors relative to livers (8.3-30-fold, P<0.001), they were not responsive to LC-HFDs (Fig 2F).

PFK is the rate-limiting enzyme in glycolysis and, like PDH, is inhibited by AcCoA and ATP as well as by citrate [34–36]. Consistent with the idea that HCCs in general up-regulate glycolysis at the expense of FAO [17, 19, 25] PFK was up-regulated by ~50% in tumors but, like each of the PK isoforms, did not respond to either of the HFDs (Fig 2G). Thus, unlike PDH, which was sensitive to both transformation and dietary intervention (Fig 2C), both PK and PFK were only responsive to transformation.

To obtain a more comprehensive view of how HFDs affected the glycolytic pathway, we quantified a panel of glycolysis-related transcripts from each of the above cohorts using RNAseq data. As expected, these transcripts were up-regulated an average of 8.7-fold in NFD tumors relative to NFD livers, attesting to the well-known increase in glycolysis and the Warburg effect that accompanies the former (Fig 2H and S1A Fig). As a group, the mean abundance of these transcripts was not altered significantly in HFD tumors but certain key individual transcripts were normalized, including Slc2a1/Glut1 the major glucose transporter in liver and the key determinant of glucose availability for the glycolytic pathway. Immuno-blotting of representative livers and tumors were generally consistent with these transcript profiling studies showing that Slc2a1 levels in both livers and tumors from animals maintained on HFDs were reduced by nearly 90% (Fig 2I).

MC-HFD and LC-HFD tumor cohorts also significantly down-regulated (by 32% and 41%, respectively) transcripts encoding mitochondrial serine hydroxymethyl transferase 2 (Shmt2), the rate-limiting enzyme for glycine and 5,10-methylenetetrahydrofolate biosynthesis, with the latter also being an essential intermediate for purine anabolism [37] (Fig 2H and S1A Fig). Shmt2 generates the majority of one-carbon units used for thymidylate and methionine synthesis, is a direct Myc target and can rescue the profound growth defect of *myc-/-* fibroblasts [38] Finally, HFD tumors also significantly down-regulated transcripts encoding 6-phosphogluconate dehydrogenase (6Pgd), the rate-limiting enzyme of the pentose phosphate pathway (Fig 2H and S1A Fig). Together with the afore-mentioned changes in FAO and PDH activity in HFD-tumors, these data support the idea that HFDs, most likely by operating through the Randle cycle, reduce glycolysis in tumors by directing this pathway away from high rates of glucose uptake and Warburg-type anabolic metabolism while concurrently reducing pyruvate flux into mitochondria.

Because HFDs can also drive *de novo* cholesterol biosynthesis [39], we quantified the key transcripts encoding the major enzymes comprising this pathway (S1B & S1C Fig). As a group, these were up-regulated in both HFD liver groups relative to those of ND-livers (P<10^−4^ in both cases) but more so in the MC-HFD group (mean up-regulation = 2.8-fold versus 1.5-fold, P = 0.0003) (Fig 2J and S1C Fig). Relative to livers, transcripts were down-regulated by 63–77% among the three tumor groups and did not differ significantly.

Much like transcripts involved in cholesterol biosynthesis, those encoding the smaller group of enzymes involved in fatty acid synthesis (FAS) were up-regulated by 6.0-fold in MC-HFD livers and by 1.5-fold in LC-HFD livers relative to ND-livers (P = 0.009 and P = 0.03, respectively). (Fig 2K and S2A Fig). On average, NFD-tumors expressed these transcripts at levels 24% lower than those of NFD-livers. MC-HFD tumors expressed these transcripts at levels exceeding those of NFD-livers by 100%, (P = 0.02) whereas LC-HFD tumors expressed these at essentially the same levels (95%). Thus, HFDs reversed the suppression of FAS-related transcripts seen in NFD-tumors.

The suppression of FAO in NFD-tumors [17, 25] (Fig 2A), was reflected in the levels of transcripts encoding the enzymes in this pathway, which were reduced by 64% (Fig 2L and S2B & S2C Fig) (P = 0.03). Consistent with the partial normalization of FAO in these tumors by a LC-HFD (Fig 2A), the transcripts as a group were up-regulated by 11% and 7% in tumors from mice on MC-HFDs and LC-HFDs, respectively with over one-quarter of the individual transcripts being significantly altered. These and the above findings regarding Cpt1a expression (Fig 2B) indicate that tumors express lower levels of transcripts involved in both FAS and FAO and that some, but not all, of these declines can be partially reversed by HFDs.

### Regulation of the extended Myc network of transcription factors

Myc and its close relatives, N-Myc and L-Myc, are members of a larger group of bHLH-ZIP transcription factors that includes the positively acting members MondoA and ChREBP along with the negative regulator Mnt (S3A Fig)) [40–42]. The Max-like factor Mlx interacts with these proteins to control target gene expression. Canonical binding sites for these, termed “ChoRE” elements, are comprised of two E-Box-like motifs (CA^C^/_T_GTG) separated by five nucleotides [41]. Together, the Myc and MondoA/ChREBP families form an “extended network” with particular regulatory overlap for genes involved in lipid and glucose homeostasis [19,40–44]. Transcriptional profiling of the extended network, along with several other transcripts whose over-expression restores various Myc functions in *myc-/-* fibroblasts [38, 45], indicated complex responses to both HFDs and/or transformation. For example, in addition to the expected marked increase in human Myc transcripts in all tumors due to the induction of the transgene, either or both of the HFDs were associated with ≥2-fold changes in transcripts encoding N-Myc, B-Myc, Mnt and ChREBP among both livers and tumors relative to their NFD counterparts (S3B Fig). Altered in response to transformation but not HFDs were transcripts encoding Max, Mga, Mlx, Mxd2-4, Myct1 and MondoA with the most dramatic changes being a 43-fold increase in Mxd3 transcripts in NFD-tumors relative to NFD-livers. Consistent with the transcript and protein disparities for PKM1 and PKM2 mentioned above (Fig 2F), protein levels for ChREBP and MondoA decreased in tumors, were largely unaffected by diet and did not reflect changes in transcript levels which, for ChREBP, were decreased by both HFDs and, for MondoA, remained unchanged (S3C Fig). Neither N-Myc nor L-Myc proteins were detcted, despite changes in transcript levels of as much as 10-28-fold. Thus, while numerous transcripts encoding members of the extended Myc network were responsive to diet and transformation, some of the protein levels were altered by transformation only and did not always conform with changes in their respective transcripts.

### Global transcriptional profiling identifies pathways involved in HFD-mediated tumor inhibition

**Fig 3:**
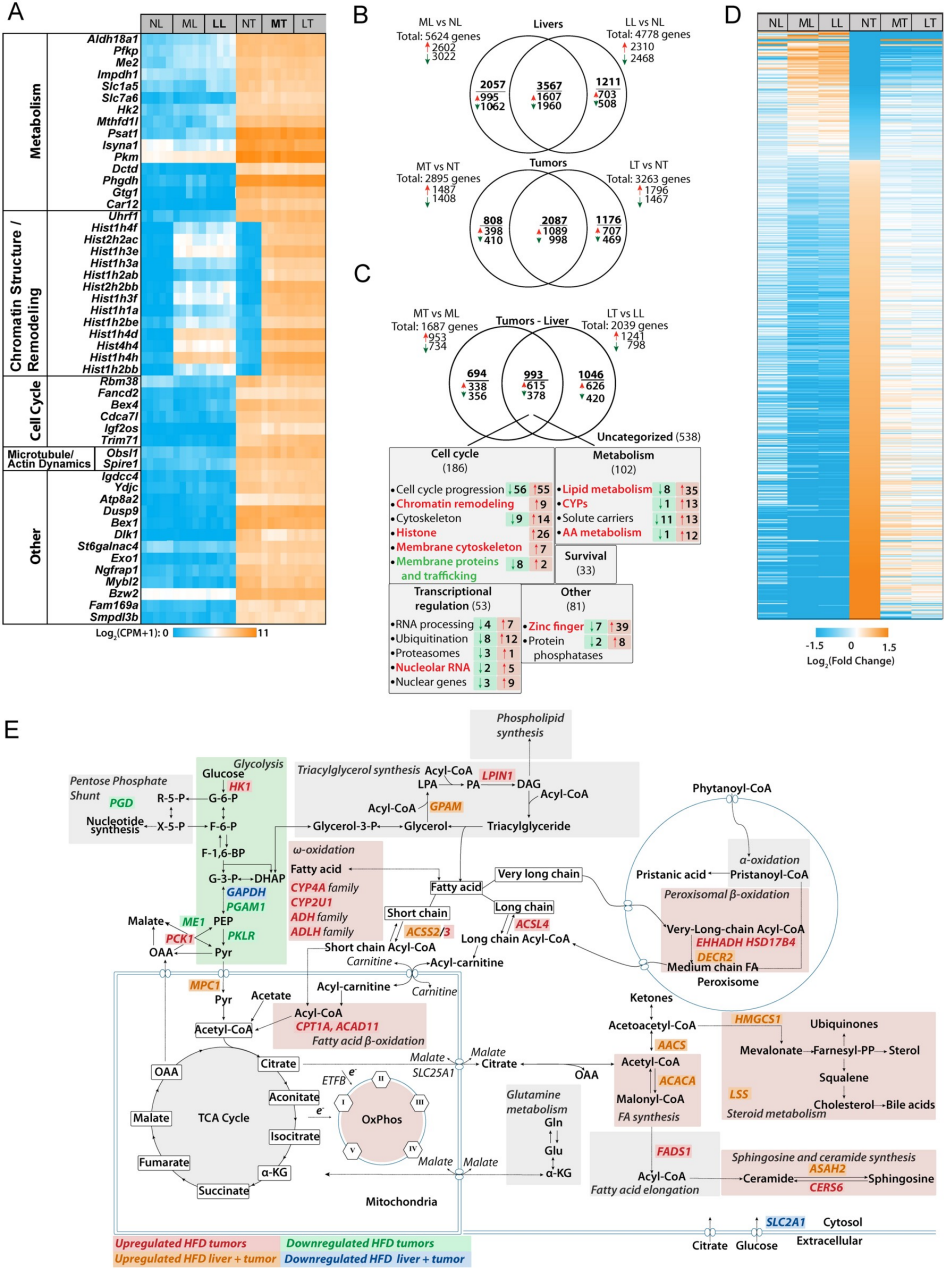
Differences among liver and tumor groups maintained on NDs or HFDs. **A**, The 50 most highly de-regulated transcripts that distinguish livers and tumors. Transcripts are grouped according to their known or assumed functional categories. See S4 Fig for actual expression levels and S2 Table for the actual functional categories into which these transcripts could be grouped. **B**, Venn diagram of differential transcript expression among NFD livers and tumors and those maintained on MC-HFDs and LC-HFDs. **C**, Venn diagram of differential transcript expression that distinguishes NFD-tumors from MC-HFD tumors and LC-HFD tumors. The 993 common transcripts represent those that are uniquely deregulated only in tumors maintained on both HFDs. See S3 Table for a full list of these. **D**, Heat map of the 682 transcripts that are de-regulated in NFD tumors and normalized by both MC-LFDs and LC-HFD (q<0.05) (See S4 Table for the complete list of these transcripts and their expression levels in each of the tumor groups. **E**, Predicted pathway de-regulation in HFD tumors based on IPA predictions. The indicated genes are taken from **C** and **D** and S3 Table. They represent transcripts that de-regulated in NFD tumors and normalized by both MC-LFDs and LC-HFD.

Unbiased RNAseq analyses of the above six groups identified numerous differentially expressed transcripts notable for several features. First, the 50 most dysregulated transcripts were all up-regulated in tumors relative to their respective liver groups regardless of diet (Fig 3A and S4 Fig). Second, 37 of these (74%) comprised four functional categories pertaining to metabolism, chromatin structure and remodeling, cell cycle control and microtubule and actin re-modeling (S2 Table). Third, 13 of 14 transcripts in the “chromatin structure and remodeling” group encoded 10 isoforms of histone H1, a single isoform each of histone H2A and H2B and one isoform of histone H4. Although these transcripts as a group were highly up-regulated in response to either HFDs or transformation alone (avg. up-regulation 121.3-fold relative to ND livers and 334-fold relative to ND tumors), the combination of HFD plus transformation led to a synergistic up-regulation (1138-fold relative to ND livers) (S4 Fig). Other histone-encoding transcripts, particularly those encoding additional histone H1 isoforms, were similarly up-regulated, albeit in a less dramatic but still synergistic manner (S5 Fig). For example, in MC-HFD livers and LC-HFD livers, these transcripts as a group were up-regulated 1.52 and 1.71-fold, respectively relative to ND livers (P = 1.26 x 10^−10^ and 1.69 x 10^−12^). In NFD tumors, the transcripts were up-regulated by 2.02 fold relative to NFD-livers (P = 3.75 x 10^−7^). In MC-MFD tumors and LC-HFD tumors, the transcripts were up-regulated by 4.38-fold and 3.70-fold, respectively relative to NFD tumors (P = 1.5 x 10^−13^ and 3.77 x 10^−12^, respectively). Again in keeping with the less dramatic but still synergistic effect of HFD and transformation, MC-HFD tumors and LC-HFD tumors up-regulated these transcripts 8.8-fold and 7.5-fold, respectively relative to ND livers (P<10^−15^ for both). Exceptions to this synergistic joint up-regulation included transcripts encoding three of the four isoforms of histone H3, histone 2H3c1, histone H2afz&x, and several others. In livers, 5624 transcripts were dysregulated in response to MC-HFDs (2602 increased and 3022 decreased) and 4778 were dysregulated in response to LC-HFDs (2310 increased and 2468 decreased) (Fig 3B). 3567 (52.2%) of these overlapped and were regulated in the same direction (1607 increased and 1960 decreased). Similarly in tumors, 2895 transcripts were dysregulated in response to MC-HFDs relative to NFD tumors (1487 increased and 1408 decreased) and 3263 transcripts were dysregulated in response to LC-HFDs (1796 increased and 1467 decreased). 2018 (51.3%) of these were shared between MC-HFD tumors and LC-HFD tumors (1089 increased and 998 decreased relative to NFD tumors).

2733 gene expression differences distinguished the slowly growing MC-HFD and LC-HFD tumors from rapidly growing NFD-tumors (Fig 3C). Of these, 993 (36.3%) were shared and thus were dysregulated only in tumors maintained on either HFDs. We focused on this subset as being the most likely responsible for the slower growth of these tumor groups. IPA categorized 455 (45.6%) of these transcripts into five distinct groups pertaining to cell cycle progression, chromatin structure & remodeling, metabolism, RNA processing and ubiquitination (S6A–S6E Fig and S3 Table).

Finally, we identified 682 tumor-specific, protein-coding transcripts that were partially or completely normalized by both HFDs (Fig 3D and S4 Table), with some of these having been identified by the prior analyses (S6A–S6E Fig and S3 Table). Evaluation by IPA showed that the 532 transcripts down-regulated in response to HFDs included those involved in cell cycle progression (E2F1,3,4,5; Mdm2, p21^*CIP1*^) mitogenic signaling (c-jun, ERK1,2) and mRNA/rRNA processing (CDK9, MED25 and SF3A1) whereas 150 transcripts up-regulated in response to HFD tumors included the tumor suppressors NF1 and FAT1. Importantly 241 of these transcripts (35.3%) encoded enzymes or other proteins involved in metabolic processes or their regulation.

Based on a combination of the above-described functional and gene expression data, we constructed a hybrid model summarizing the most likely means through which HFDs normalize HCC metabolism and thus likely suppress growth (Fig 3E). Relative to NFD HCCs, these included a down-regulation of glycolysis and variable but significant increases in Oxphos, FAO and *de novo* FAS.

### Transcripts involved in cholesterol biosynthesis, FAO and glycolysis correlate with patient survival

**Fig 4:**
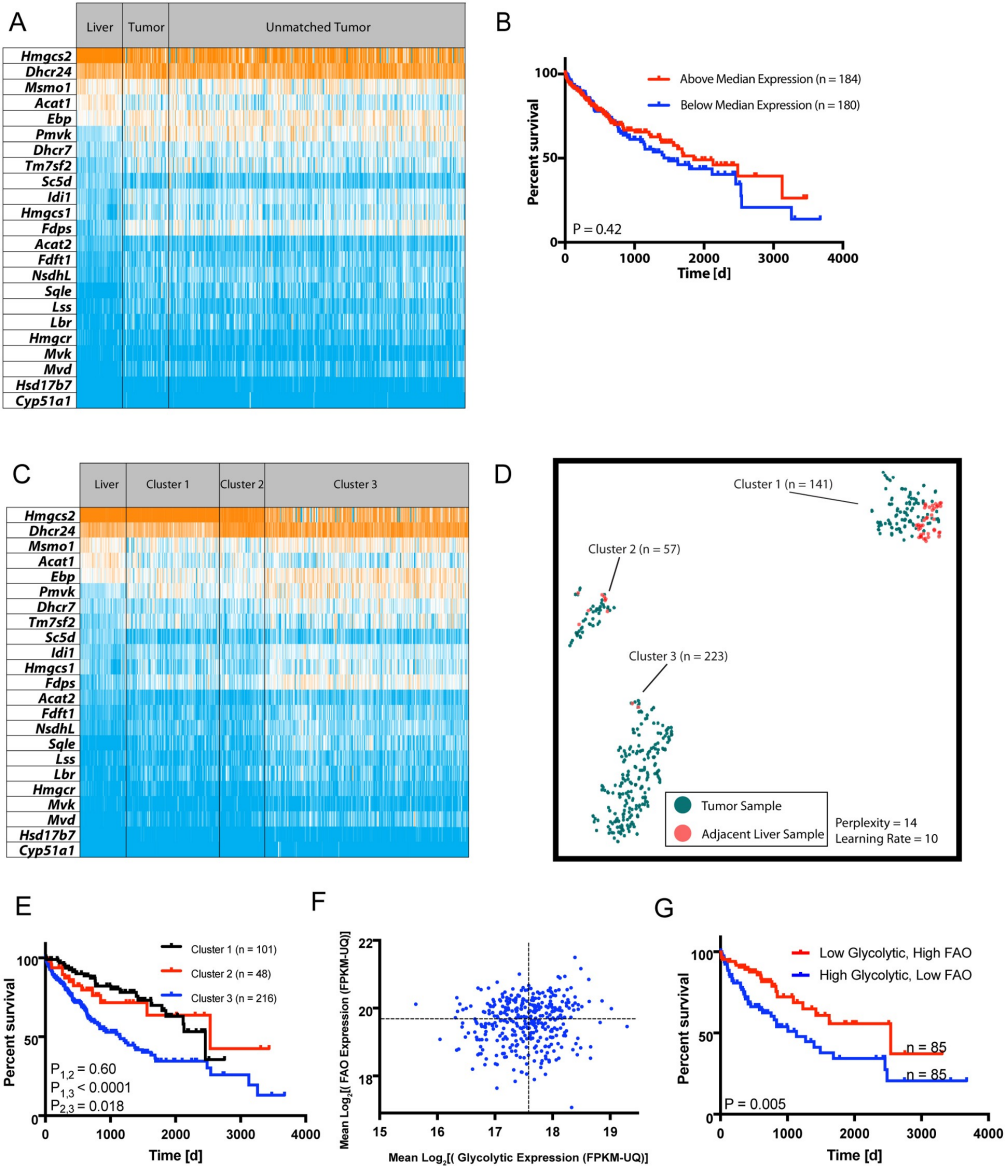
Deregulation of transcripts involved in lipid metabolism correlate with human HCC survival. **A**, Expression of transcripts encoding the cholesterol biosynthetic enzymes depicted in S1B Fig in 50 matched liver and HCC samples along with 371 additional unmatched HCCs. Transcripts are arranged from the most to the least abundant based on their mean expression in NFD murine livers. All values were obtained from analyses of human tissues previously deposited in TCGA. **B**, Kaplan-Meier survival curves of HCC patients with the highest and lowest mean levels of cholesterol transcript expression relative to control livers from **A**. **C**, The tumors depicted in **A** were re-arranged into three groups with distinct differences in their patterns of transcript expression. **D**, Unsupervised t-SNE-based clustering of the normal liver and three tumor groups from panel **C** showing improved resolution of transcript expression pattern differences [17]. **E**, Kaplan-Meier survival curves of the three HCC patient cohorts shown in **C** and **D**. Significant differences in survival among the groups based on log-rank test are indicated. **F**, HCCs from **A** were analyzed for their mean levels of transcripts encoding FAO- and glycolysis-related enzymes and plotted on corresponding axes of the graph. **G**, Kaplan-Meier survival of HCC patients with FAO- and glycolysis-related transcript level ratios from the upper left quartile (high FAO/low glycolysis) and the lower right quartile (low FAO/high glycolysis) of panel **F**. Significant differences in survival among the groups based on log-rank test are indicated.

Because transcripts involved in lipid metabolism and glycolysis were altered by HFDs, we asked whether similar findings applied to primary human HCCs and other cancers. The mean expression levels of cholesterol biosynthetic enzyme-encoding transcripts (S1B Fig) did not significantly differ among 371 human HCC samples and 50 matched liver samples (Fig 4A) (average fold-differences between liver and tumor groups = 1.042, P = 0.54, paired ratio t-test) and the survival of patients whose tumors expressed the highest and lowest levels of these transcripts was similar (Fig 4B). However, as observed in murine HCCs (Fig 2J), differences in transcript patterns were evident (Fig 4C), particularly when analyzed by t-SNE, a dimensionality reduction technique of particular utility for analyzing non-linear relationships [31]. This identified three distinct HCC clusters (Fig 4D) one of which (Cluster 3) was associated with a particularly unfavorable clinical course (Fig 4E). Eight additional human tumor types were identified whose patterns of cholesterol related transcript expression were similarly predictive of survival (S7 Fig). A Random Forest Classifier model [19, 31] showed that, in eight of the nine tumor cohorts, these patterns were largely determined by a small subset of transcripts, comprised of DHCR24, HMGCS2, PMVK and ACAT1/2 (S8 Fig).

The same human TCGA data were next used to show that individuals whose HCCs were in the quartile with the highest FAO:glycolytic transcript ratios (i.e. the most “liver-like”, Fig 4F) experienced longer survival relative to those with ratios in the lowest quartile (i.e. the most “tumor-like”) (Fig 4G). Similar survival differences were noted in six other disparate tumor groups (S9 Fig).

Like those for cholesterol biosynthesis, FAO transcript expression patterns were also found to be predictive of survival in HCC and six other cancers (S10 Fig). Random Forest Classification again identified a small number of transcripts, particularly those for Acadvl and Echs1 as being the primary determinants of pattern diversity (S11b Fig).

## Discussion

The correlation between obesity and cancer incidence is well-established although the impact on actual survival remains controversial. The etiology of this association is complex but is largely believed to reflect collective long-term changes in acute and chronic inflammatory responses, reactive oxygen species production and hormonal signaling [46–48]. Less well-studied is whether shorter-term dietary interventions affect cancer incidence and survival and what the underlying mechanisms of any beneficial effects might be.

Among the potential advantages offered by nutrient-based metabolic normalization are its ease of administration, its low cost and toxicity and its potential for simultaneously impacting multiple pathways. This latter feature might afford protection against the inherent metabolic plasticity of cancer cells that allows them to readily circumvent therapies directed against individual metabolic enzymes [49]. The relatively short time frames for such interventions would also potentially avoid their longer-term obesity- and cancer-promoting tendencies [46–48]. For example, the diets used in the current study did not lead to a significant weight gain in non-tumor-bearing mice (Fig 1B). Experience with ketogenic diets (KGDs) provides an additional example of the benefits of HFDs with regard to the current study. Their high fat and low carbohydrate content imposes a state of pseudo-starvation by forcing fatty acids rather than glucose to be the primary source of AcCoA [50]. KGDs also reduce insulin levels, thereby limiting glycolysis at its most proximal step and depriving tumor cells and their stroma of important nutritional and hormonal support [50, 51]. Cancers such as the HCCs studied here might be particularly prone to such dietary intervention given their inherently robust Randle cycle [15, 16].

KGDs, either alone or in combination with other therapies, have shown some benefit in experimental settings [50, 52–55]. However, these studies almost exclusively employed cancer xenografts in immuno-compromised mice and neither metabolic nor molecular profiling was performed. Also lacking are any studies linking “HFD-like” molecular and/or metabolic signatures to cancer survival in large groups of human cancers.

The Warburg effect is among the fundamental metabolic properties that distinguish cancer cells from their normal counterparts [1, 14, 33]. Its benefits include the provision of critical macromolecular precursors and anti-oxidants, increased and beneficial micro-environmental acidification via the release of lactate and a guaranteed source of ATP during the episodic hypoxia that typifies most cancers [2, 12, 50, 56]. Enhanced glutaminolysis, another common attribute of cancer cells, can furnish additional macromolecular precursors when tissue oxygenation has been restored while generating reducing equivalents and ATP in an AcCoA-independent manner [2, 7, 12]. Targeting these or other hyperactive metabolic pathways has been viewed as a plausible therapeutic strategy [4, 13, 14, 18].

We employed the HCC model described herein for several reasons. First, it allows dietary manipulation of an endogenously-arising tumor. Second, HCCs originate as the result of a single molecular “hit” (i.e. de-regulated Myc), upon which they remain permanently and reversibly dependent [17, 24]. This allows any observable dietary impact to be attributed to a single, well-defined initiating oncogenic event. Third, Myc orchestrates global metabolic re-programming at multiple levels thus making it an ideal oncoprotein to target with dietary-based approaches [4, 12, 7, 11, 13, 17, 26–29, 41, 57]. Finally, the liver’s highly active Randle cycle afforded the opportunity to examine the regulation and co-dependency of glycolysis, Oxphos and FAO by HFDs [15, 58].

Rather than KGDs, we employed diets with only moderately elevated fat content that allowed us to ask whether the fatty acid chain length (i.e. long vs. medium) differentially affected any of the studied parameters. Although both HFDs normalized the same selective aspects of tumor metabolism, MC-HFD tended to perform somewhat better. This may have been the result of the mitochondrial transport of MC fatty acids being passive and thus not being subject to the constraints of the rate-limiting enzyme Cpt1a. Despite these small differences on metabolism, both HFDs equally extended survival (Fig 1A).

A prominent effect of both HFDs on HCC metabolism included the normalization of PDH complex activity (Fig 2C). Randle cycle-mediated suppression of glucose utilization occurs predominantly through PDH and is achieved via several distinct mechanisms reflecting PDH’s complex regulation. Among these are the direct and non-mutually exclusive inhibition of the enzyme complex by free fatty acids, citrate and high ratios of AcCoA:CoA, ATP:ADP and NADH:NAD+, all of which are increased by dietary fat [22, 59, 60]. The fact that tumor-associated PDHE1 subunit phosphorylation was unaffected by diet (Fig 2D) was consistent with this notion of direct enzymatic inhibition rather than the more indirect inhibition resulting from changes in stimulatory PDP2 phosphatase and inhibitory PDK1 kinase. However, these results must be interpreted cautiously. While we have not previously observed differences in pyruvate levels between livers and tumors of mice maintained on NFDs [18], it is possible that HFDs, by virtue of their ability to inhibit HCC growth, reduce the Warburg effect and redirect glycolysis towards the production of pyruvate. A reduced overall rate of glycolysis would also likely reduce the need for NAD^+^ as an electron acceptor during glucose oxidation, thus reducing the need for the NAD^+^-generating LDH reaction and thereby contributing further to the pyruvate pool.

In contrast to PDH, PFK activity was increased by ~50% in tumors and remained unaltered in response to HFDs diet (Fig 2G). These results are consistent with previous reports that PDH, more so than PFK, is the key modulator of the Randle cycle [15, 58]. Combined with the reduced expression of the glucose transporter Slc2a1/Glut1, the key determinant of glucose uptake and transcripts for 6Pgd, the rate-limiting step in the pentose phosphate pathway (Fig 2H and 2I), these findings collectively suggest that a major effect of HFDs on tumor growth may occur via the down-regulation of glucose uptake and the shunting of its immediate metabolite, glucose-6-phosphate, into the pentose phosphate pathway without necessarily altering the state of PFK. Despite the above-documented alterations of the glycolytic pathway in response to HFDs, we found there to be no differences in the serum glucose levels of tumor-bearing mice at the time of sacrifice and were otherwise indistinguishable from those of their non-tumor-bearing counterparts (not shown). As expected, serum lactate levels were elevated by approximately two-fold in the former group but also remained unaffected by diet (not shown). This latter finding, in combination with the presumptive reduced glucose uptake and its conversion to AcCoA by HFD tumors suggests that a greater fraction of their available pyruvate is being converted to lactate, despite their slower rates of growth and a likely reduction in glycolytic flux into anabolic pathways.

NFD livers prefer glycolytically-derived AcCoA as the initial substrate for *de novo* FAS [17, 19]. When maintained on HFDs, however, these livers up-regulated FAS-related transcripts an average of 1.5-6-fold (Fig 2K and S2A Fig). On the other hand, rapidly growing HCCs and HBs prefer to incorporate pre-existing lipids such as palmitate and cholesterol into new membranes, thus likely explaining their paucity of stored neutral lipids and their down-regulation of transcripts relevant to lipid biosynthesis (Figs 1C, 1D and 2K and S1B and S2A Figs) [17, 19].

Similar but less pronounced behaviors were seen with cholesterol synthesis-related transcripts (Fig 2J and S1B Fig). Although absolute levels of these also did not correlate with survival in a cohort of human HCC patterns (Fig 4B), their expression patterns did and extended to several other cancer types (Fig 4D & 4E and S7 Fig). These results recalled our recent findings concerning the prognostic value of ribosomal protein transcript patterns in multiple cancers [31]. It remains unclear as to precisely how such differential patterning affects patient outcome. However, of the small subset of cholesterol biosynthesis-related transcripts implicated as being the most responsible for determining the specific tumor patterns (S8 Fig), namely HMGCS2, DHCR24 and PMVK, the former is a direct Myc target [59] and all three have individually been previously shown to be de-regulated in and correlated with survival in diverse cancer types [59, 61–65].

The relationship between FAO and glycolysis in murine HCCs was extended to multiple human cancers and showed that tumors with the highest FAO-:glycolysis-related transcript ratios were associated with longer survival than those with the lowest ratios (Fig 4F and 4G and S9 Fig). These findings support studies in mice showing that deliberately manipulating these pathways can slow tumor progression [9, 49, 50, 52–56, 66–70]. It remains unknown whether particular levels and/or patterns of FAO, glycolytic and cholesterol-related transcripts that are associated with differential patient survival represesent intrinsic or acquired differences in tumor metabolic pathways.

A key question raised by our studies is the degree to which the above-described HFD-mediated molecular and metabolic alterations are the direct cause rather than simply the consequence of impaired tumor growth. The fact that many of the changes were also observed in MC-HFD and LC-HFD livers argues in favor of the former. This was particularly notable for the 682 tumor transcripts whose expression was normalized by both HFDs (Fig 3D and S4 Table). Collectively, these observations provided strong evidence that these changes were directly responsible for tumor growth inhibition.

What is clear from our studies is that certain select pathways are more amenable to metabolic normalization than others. In the future, it will be important to determine whether the more refractory pathways can eventually be normalized as well, whether through more intense or prolonged exposure to HFDs or by the employment of other dietary interventions, perhaps in combination with those described here.

In conclusion, the short-term administration of HFDs significantly prolonged survival in a murine model of aggressive HCC [11, 24]. The underlying mechanisms for this effect are likely multiple, although related by virtue of the fact that they appear to involve a combination of molecular, epigenetic and metabolic modifications centered around the Warburg effect and the Randle cycle. In the latter case, where FAO and glycolysis tend to be mutually inhibitory [15, 16], HFDs re-programmed tumors to acquire more “liver-like” metabolic profiles for some pathways. In addition to normalization of the Warburg effect these changes included up-regulation of Oxphos and increased reliance on FAO as an energy source as occurs in normal liver. Molecular analyses revealed that MC-HFD and LC-HFD tumors shared nearly 1000 dysregulated transcripts with over one-third of them being implicated in metabolic regulation, cell cycle control, chromatin structure and remodeling, ubiquitination and RNA processing. A profound and synergistic up-regulation of a large majority of histone-encoding transcripts was an important feature of the gene signature profile in HFD-tumors and suggested that some of the observed re-programming involved epigenetic changes. An additional subset of 682 genes that was significantly dysregulated in tumors was partially-completely normalized by both HFDs, strongly suggesting a collective role in mediating tumor suppression (Fig 3D and S4 Table). Finally, marked differences in patient survival for several different tumors, based solely on the FAO:glycolytic transcript ratios, fully corroborated the notion that dietary manipulations similar to those described here might offer the prospect of simple, cost-effective and non-toxic therapeutic alternatives, particularly for those individuals who refuse or are not candidates for standard chemotherapies or who wish to supplement established regimens with less toxic alternatives. For those who do opt for standard drug-based regimens, stratification of certain tumor types based on the patterns of expression of FAO and/or cholesterol biosynthetic transcripts could aid in identifying the most suitable chemotherapeutic and/or dietary options and contributre to decisions regarding the frequency of post-therapy follow-up.

## Supporting information

S1 Fig Expression of transcripts encoding enzymes involved in glycolysis and cholesterol biosynthesis. **A**, Heat map of glycolysis-related transcripts. The depicted heat map is identical to that shown in Fig 2H except that mean expression values for each transcript based on RNAseq profiling are included. **B**, The pathway of cholesterol biosynthesis. Enzymes whose respective transcripts were used for the construction of heat maps, are indicated in red. **C**, Heat map of cholesterol biosynthesis transcript expression. Transcripts for IDI2 were excluded from the analyses due to very low expression values across all samples. The depicted heat map is identical to that shown in Fig 2J except that mean expression values for each transcript based on RNAseq profiling are included.
(PDF)

S2 Fig Expression of transcripts encoding proteins involved in FA metabolism. **A**, The heat map for FAS-related transcripts is identical to that shown in Fig 2I except that mean expression values for each transcript based on RNAseq profiling have now been included. **B**, Pathway for FAO. Some of the enzymes whose respective transcripts were used for the construction of heat maps, are indicated in red. **C**, Heat map of FAO transcript expression. Transcripts are arranged as depicted in Fig 2J except that mean expression values are now included.
(PDF)

S3 Fig Complex regulation of members of the “Extended Myc Network” in response to HFDs and/or transformation. A, Myc pathway members, comprised of the bHLH-ZIP proteins c-, N-, L-Myc, Max and Mxd(1–4), bind to E-Boxes as homo- or heterodimers and positively or negatively regulate transcription as shown. MondoA/ChREBP pathway members, comprised of MondoA, ChREBP, Mlx and Mnt, also bind to target gene sites containing ChORE elements [4, 19, 34–36]. Cross-talk between the Myc and MondoA/ChREBP pathways is mediated via Mxd1, Mxd4 and Mnt, which can associate with Max or Mlx to negatively regulate either pathway. The Extended Myc Network directly regulates overlapping groups of genes involved primarily in carbohydrate and lipid metabolism [34–36, 40]. B, Heat map of transcripts across all members of the Extended Myc Network in NFD and HFD livers and tumors. Note that transcripts for Myc include those of human origin, encoded by the Dox-regulatable transgene. Additional transcripts include Myc targets Myct1, Hmga1 and Shmt, which can restore certain Myc functions in *myc-/-* fibroblasts [41, 42] C, Immuno-blots for select members of the Myc Network in four set of tissues.
(PDF)

S4 Fig The 50 most highly de-regulated transcripts that distinguish livers and tumors. Transcripts are grouped as shown in Fig 3A with mean expression values shown for each group.
(PDF)

S5 Fig Synergistic up-regulation of transcripts encoding the majority of histones in HFD tumors. The histone transcripts from Fig 3A are indicated at the top of the heat map in bold-faced print and were among the 50 most dysregulated transcripts. The vast majority of histone H2 member transcripts were expressed at extremely low-undetectable levels in livers, were not significantly up-regulated in response to dietary intervention or transformation and are therefore not included in this heat map.
(PDF)

S6 Fig Heat maps of transcripts from Fig 3C showing expression of genes that are altered only in tumors from mice maintained HFDs of either type. **A**, Transcripts related to cell cycle progression. **B**, Transcripts related to chromatin structure and remodeling. **C**, Transcripts related to metabolism. D Transcripts related to RNA processing. **E**, Transcripts related to ubiquitylation. See S3 Table for a full list of these and the remaining members of the 993 common transcript group (Fig 3C).
(PDF)

S7 Fig t-SNE analysis of cholesterol-related transcripts identifies distinct tumor cohorts that correlate with patient survival. t-SNE patterns for the transcripts listed in Fig 2J were calculated from TCGA expression profiles and displayed as previously described (blue dots) [17]. Where available, t-SNE patterns for matched normal human tissues were similarly calculated and plotted (red dots). Kaplan-Meier survival data for each of the tumor cohorts were then plotted as shown in Fig 4E.
(PDF)

S8 Fig Random Forest Classification of cholesterol biosynthesis-related transcripts most responsible for t-SNE clustering patterns in nine tumors. Each of the histograms indicates the transcripts that were the most deterministic of the patterns depicted in S7 Fig.
(PDF)

S9 Fig Distribution of FAO- and glycolysis-related transcripts and Kaplan-Meier survival curves as depicted in Fig 4F & 4G for six other human cancers. Data from TCGA were analyzed as described in Materials and Methods. Points on the scattergrams represent the mean expression levels for both FAO-related transcripts and glycolysis-related transcripts in each sample as depicted in Fig 4F.
(PDF)

S10 Fig t-SNE analysis of FAO-related transcripts identifies distinct tumor cohorts that correlate with patient survival. t-SNE for the FAO transcripts depicted in S2B & S2C Fig were analyzed in TCGA tumor types. Kaplan-Meier survival curves were then plotted for each of the clusters where significant survival differences for the indicated tumor type were observed.
(PDF)

S11 Fig Random Forest Classification of FAO-related transcripts most responsible for t-SNE clustering patterns. Each of the histograms indicates those transcripts which were the most deterministic of the patterns depicted in S10 Fig.
(PDF)

S1 Table Antibodies and conditions employed for the current study. (PDF)

S2 Table Functional categories of the top 50 most dysregulated transcripts. (PDF)

S3 Table XLS spread sheet of the 993 overlapping genes shown in Fig 3C. The transcripts depicted in S6 Fig are listed at the top of the Table.
(PDF)

S4 Table XLS spread sheet of the 682 transcripts from Fig 3D that were deregulated in NFD tumors and significantly normalized by both HFDs. (PDF)

S1 File Original images of western blot data in Figs 1C, 2B, 2F, 2D and 2I and S3C Fig. (PDF)
