# Supplementary material for: Inhibition of hepatocellular carcinoma by metabolic normalization
Source: PLoS One. 2019 Jun 26;14(6):e0218186. doi: 10.1371/journal.pone.0218186 (PMC6594671; doi:10.1371/journal.pone.0218186)
Supplement: S1 Fig — A, Heat map of glycolysis-related transcripts. The depicted heat map is identical to that shown in Fig 2H except that mean expression values for each transcript based on RNAseq profiling are included. B, The pathway of cholesterol biosynthesis. Enzymes whose respective transcripts were used for the construction of heat maps, are indicated in red. C, Heat map of cholesterol biosynthesis transcript expression. Transcripts for IDI2 were excluded from the analyses due to very low expression values across all samples. The depicted heat map is identical to that shown in Fig 2J except that mean expression values for each transcript based on RNAseq profiling are included. (PDF) [file pone.0218186.s001.pdf]

A

	NL	ML	LL	NT	MT	LT
Pklr	233.1	260.0	179.5	54.1	** 32.4	*** 30.8
Pgam1	271.8	238.3	* 181.6	768.2	* 501.9	** 422.2
Pgk1	344.2	252.5	* 228.0	570.4	* 361.8	** 313.6
Slc2a1	14.7	** 7.6	*** 6.5	23.0	** 12.1	** 13.0
Gapdh	79.9	*** 52.5	*** 46.2	126.2	102.3	** 94.1
Pdhb	141.1	* 112.4	132.8	177.5	144.1	* 134.5
Hk1	3.2	* 4.8	3.9	6.0	** 9.3	* 9.1
Eno1	326.8	289.3	* 212.0	1461.1	1136.1	* 949.2
Gapdhs	0.6	0.8	** 0.3	0.2	0.3	* 0.3
Eno1b	169.0	163.1	122.2	832.1	684.0	593.8
Pkm	13.0	** 22.6	18.9	959.1	782.7	704.8
Pfkm	22.3	19.4	* 14.3	8.5	7.8	6.2
Slc2a3	0.4	*** 1.0	* 0.7	1.5	* 2.5	2.1
Gpi1	166.2	186.5	* 115.1	378.5	357.5	304.1
Pfkp	2.1	*** 5.4	*** 4.0	125.2	130.0	108.6
Hk3	2.5	*** 7.5	*** 5.1	4.7	6.7	5.8
Pfkl	44.8	50.1	43.0	137.9	147.6	156.2
Eno3	5.5	*** 2.8	*** 2.8	6.0	5.0	5.5
Aldob	3593.9	* 5483.8	4167.2	954.7	1081.4	1096.1
Slc2a4	0.6	* 1.3	** 1.5	0.9	1.5	1.1
Hk2	1.1	*** 3.4	*** 1.9	56.3	60.8	59.2
Pdha1	145.5	** 204.1	171.3	123.3	118.4	118.5
Bpgm	22.7	* 29.1	28.2	21.9	22.6	22.6
Aldoa	81.3	99.9	78.5	497.6	511.0	482.4
Slc2a2	326.5	408.2	* 579.6	178.0	218.9	186.4
Aldoc	15.0	*** 31.0	16.1	4.7	6.3	4.8
Pgd	46.4	** 67.3	*** 20.0	136.2	*** 83.5	*** 63.6
Shmt2	221.9	*** 121.5	** 145.5	377.3	* 257.0	** 222.5

Lowest value Highest value

Log_2(Fold Change)

P for NL vsML/LL or NT vs MT/LT

* P≤0.05 **P≤0.01 ***P≤0.001

B

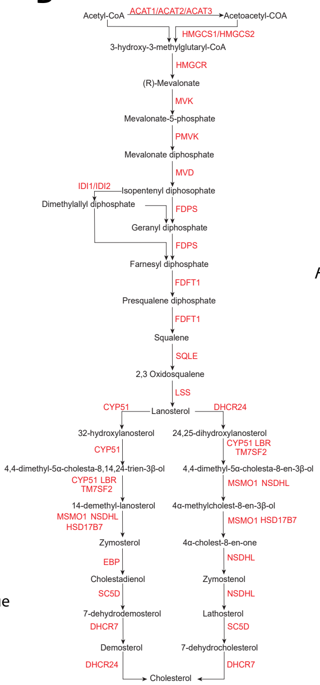

C

	NL	ML	LL	NT	MT	LT
Sc5d	488.6	*** 1161.8	* 825.8	37.6	53.1	* 63.0
Tm7sf2	148.3	** 259.4	160.7	12.0	13.7	15.1
Acat3	412.9	282.7	382.2	43.4	47.2	44.5
Msmo1	185.3	*** 429.5	** 367.3	41.1	39.6	51.6
Fdps	192.0	*** 788.2	** 376.4	63.4	88.2	74.5
Cyp51	201.5	*** 558.3	* 332.3	56.5	57.2	68.6
Ebp	236.3	237.9	238.0	36.4	32.4	35.0
Mvd	36.2	*** 115.7	43.2	10.3	10.6	11.0
Hmgcs1	379.3	*** 2132.8	** 776.5	179.7	235.2	234.3
Pmvk	39.1	*** 204.5	*** 67.1	17.6	* 25.0	18.4
Hmgcs2	2717.4	* 1678.8	2398.0	413.2	415.0	608.4
Hsd17b7	95.0	*** 257.7	* 140.0	35.0	42.1	41.6
Lss	86.1	*** 495.3	* 128.0	54.9	79.3	68.8
Mvk	25.1	** 46.1	* 40.6	9.9	12.2	10.6
Nsdhl	79.1	*** 153.0	95.7	28.0	25.2	25.1
Idi2	0.0	0.0	0.0	0.0	*** 0.2	** 0.1
Hmgcr	94.9	*** 674.4	** 189.4	115.7	** 218.3	136.2
Idi1	249.4	*** 814.4	** 478.8	203.4	172.8	154.9
Acat2	86.3	*** 441.0	** 126.3	94.6	115.3	81.2
Sqle	70.8	*** 278.3	* 112.2	71.5	59.5	58.8
Acat1	488.3	598.1	* 744.5	307.7	315.1	325.6
Dhcr24	852.8	958.4	598.0	428.5	461.6	350.0
Dhcr7	107.6	123.1	* 76.4	94.6	73.5	*** 53.0
Fdft1	116.9	*** 314.5	* 177.8	239.0	204.3	* 152.3
Lbr	15.9	*** 33.8	*** 27.7	121.0	140.2	115.0

Lowest value Highest value

Log_2(Fold Change)

P for NL vsML/LL or NT vs MT/LT

* P≤0.05 **P≤0.01 ***P≤0.001

S1 Fig. Expression of transcripts encoding enzymes involved in glycolysis and cholesterol biosynthesis. A, Heat map of glycolysis-related transcripts. The depicted heat map is identical to that shown in Fig. 2H except that mean expression values for each transcript based on RNAseq profiling are included. **B**, The pathway of cholesterol biosynthesis. Enzymes whose respective transcripts were used for the construction of heat maps, are indicated in red. **C,** Heat map of cholesterol biosynthesis transcript expression. Transcripts for IDI2 were excluded from the analyses due to very low expression values across all samples. The depicted heat map is identical to that shown in Fig. 2J except that mean expression values for each transcript based on RNAseq profiling are included.
