# Supplementary material for: Inhibition of hepatocellular carcinoma by metabolic normalization
Source: PLoS One. 2019 Jun 26;14(6):e0218186. doi: 10.1371/journal.pone.0218186 (PMC6594671; doi:10.1371/journal.pone.0218186)
Supplement: S2 Fig — A, The heat map for FAS-related transcripts is identical to that shown in Fig 2I except that mean expression values for each transcript based on RNAseq profiling have now been included. B, Pathway for FAO. Some of the enzymes whose respective transcripts were used for the construction of heat maps, are indicated in red. C, Heat map of FAO transcript expression. Transcripts are arranged as depicted in Fig 2J except that mean expression values are now included. (PDF) [file pone.0218186.s002.pdf]

A

	NL	ML	LL	NT	MT	LT
Acacb	108.2	*** 518.4	106.0	17.6	*** 44.6	15.5
Fasn	740.4	*** 4611.9	1199.1	602.9	*** 1335.4	754.0
Acly	317.0	*** 1922.9	438.8	234.9	362.8	268.8
Acaca	151.3	*** 950.3	*** 227.8	197.7	*** 441.9	288.7
Mcat	26.1	*** 33.0	*** 34.8	36.0	39.2	30.6

Lowest value   Highest value

Log_2(Fold Change)

P for NL vsML/LL or NT vs MT/LT

* P≤0.05 **P≤0.01 ***P≤0.001

B

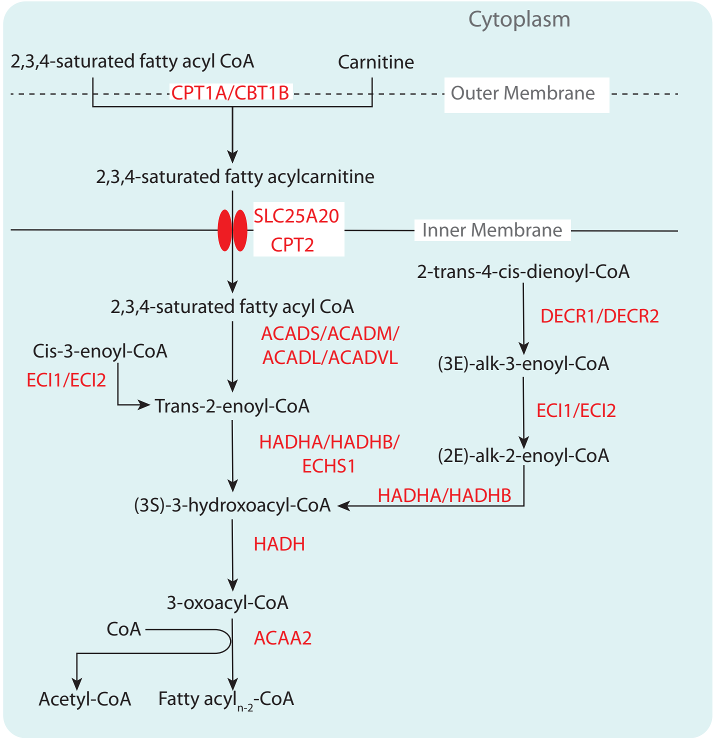

C

	NL	ML	LL	NT	MT	LT
Eci3	0.6	1.0	0.3	0.0	* 0.1	0.0
Slc27a5	989.7	883.8	970.3	35.5	64.2	* 88.7
Decr2	217.9	*** 391.9	* 332.1	15.7	21.4	** 28.6
Acad11	447.6	505.7	544.9	58.3	* 86.7	** 108.6
Acaa1b	1351.7	1213.6	893.3	131.6	192.9	119.3
Acadsb	253.1	** 165.9	277.9	42.7	44.4	46.8
Acsl5	521.0	*** 1431.6	654.3	168.9	211.5	174.7
Pccb	190.1	* 134.0	178.4	35.4	33.3	38.0
Cpt1a	377.5	* 562.3	476.6	114.9	145.9	*** 264.0
Acsl4	101.9	126.6	96.3	28.7	** 47.2	* 42.0
Eci2	180.3	185.0	* 264.8	53.4	62.2	66.1
Slc25a20	124.3	131.8	148.6	35.8	36.3	42.2
Ech1	282.2	343.0	366.3	90.9	96.9	97.6
Acaa1a	241.1	** 165.7	209.4	61.2	60.2	60.4
Echdc1	104.5	*** 160.8	120.8	40.6	42.5	41.0
Mut	251.9	230.9	289.9	84.0	96.4	93.9
Acsl1	1550.9	1083.8	1592.3	492.6	680.5	546.0
Decr1	217.6	267.7	* 303.3	99.5	100.7	93.5
Echdc2	221.1	** 148.4	207.4	80.4	75.9	67.6
Acadl	234.9	*** 146.1	*** 128.0	77.8	*** 48.8	65.0
Hadha	465.0	425.0	442.5	235.7	231.4	237.0
Echs1	288.6	335.0	383.2	178.3	170.5	137.9
Acad8	83.8	74.4	86.5	47.7	42.8	41.8
Cpt2	200.6	*** 105.7	*** 126.4	85.8	67.0	75.1
Acad10	40.8	36.3	38.9	25.7	31.9	23.1
Pcca	109.4	*** 57.0	** 74.7	49.5	48.3	38.3
Acads	199.0	193.3	208.6	123.7	113.6	102.7
Echdc3	68.3	*** 44.7	56.1	36.4	36.3	* 28.2
Hadhb	313.8	391.5	390.4	269.7	268.6	269.3
Acadm	384.0	381.8	382.1	282.4	260.6	260.5
Eci1	118.3	128.0	136.9	100.6	95.0	89.6
Acad12	14.3	17.0	14.2	11.9	11.5	* 8.8
ACSL3	35.9	*** 134.2	46.5	85.8	98.9	84.3
Hadh	350.1	369.9	316.4	364.0	335.2	305.9
Acad9	55.8	58.6	52.3	101.7	100.2	81.1
Cpt1b	2.6	2.8	2.1	72.5	86.9	84.6
Cpt1c	0.6	0.4	0.4	8.7	* 5.2	7.9

Lowest value   Highest value

Log_2(Fold Change)

P for NL vsML/LL or NT vs MT/LT

* P≤0.05 **P≤0.01 ***P≤0.001

S2 Fig. Expression of transcripts encoding proteins involved in FA metabolism. A, The heat map for FAS-related transcripts is identical to that shown in Fig. 2I except that mean expression values for each transcript based on RNAseq profiling have now been included. **B,** Pathway for FAO. Some of the enzymes whose respective transcripts were used for the construction of heat maps, are indicated in red. **C,** Heat map of FAO transcript expression. Transcripts are arranged as depicted in Fig. 2J except that mean expression values are now included.
