# Supplementary figures and images for: Inhibition of hepatocellular carcinoma by metabolic normalization
Source: PLoS One. 2019 Jun 26;14(6):e0218186. doi: 10.1371/journal.pone.0218186 (PMC6594671; doi:10.1371/journal.pone.0218186)

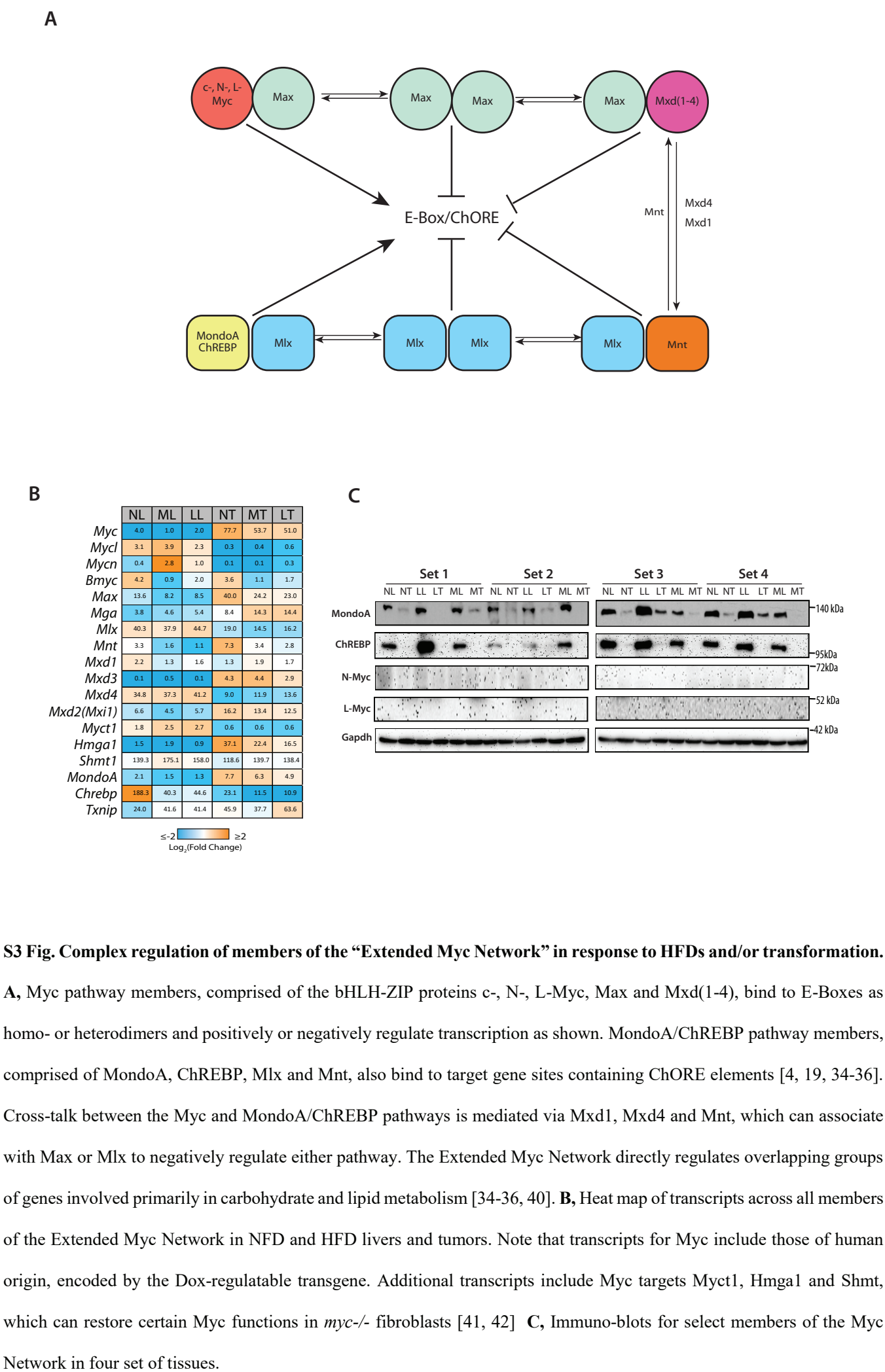

Supplement: S3 Fig — A, Myc pathway members, comprised of the bHLH-ZIP proteins c-, N-, L-Myc, Max and Mxd(1–4), bind to E-Boxes as homo- or heterodimers and positively or negatively regulate transcription as shown. MondoA/ChREBP pathway members, comprised of MondoA, ChREBP, Mlx and Mnt, also bind to target gene sites containing ChORE elements [4, 19, 34–36]. Cross-talk between the Myc and MondoA/ChREBP pathways is mediated via Mxd1, Mxd4 and Mnt, which can associate with Max or Mlx to negatively regulate either pathway. The Extended Myc Network directly regulates overlapping groups of genes involved primarily in carbohydrate and lipid metabolism [34–36, 40]. B, Heat map of transcripts across all members of the Extended Myc Network in NFD and HFD livers and tumors. Note that transcripts for Myc include those of human origin, encoded by the Dox-regulatable transgene. Additional transcripts include Myc targets Myct1, Hmga1 and Shmt, which can restore certain Myc functions in myc-/- fibroblasts [41, 42] C, Immuno-blots for select members of the Myc Network in four set of tissues. (PDF) [file pone.0218186.s003.pdf]
