# Supplementary material for: Inhibition of hepatocellular carcinoma by metabolic normalization
Source: PLoS One. 2019 Jun 26;14(6):e0218186. doi: 10.1371/journal.pone.0218186 (PMC6594671; doi:10.1371/journal.pone.0218186)
Supplement: S4 Fig — Transcripts are grouped as shown in Fig 3A with mean expression values shown for each group. (PDF) [file pone.0218186.s004.pdf]

		NL	ML	LL	NT	MT	LT
Metabolism	*Aldh18a1*	1.36	2.67	1.90	7.07	6.97	6.94
	Pfkp	1.64	2.68	2.31	6.98	7.03	6.78
	Me2	1.50	2.63	2.34	6.51	6.49	6.23
	Impdh1	2.24	2.27	1.94	6.84	6.25	6.29
	Slc1a5	1.62	2.17	1.07	6.59	6.01	5.85
	Slc7a6	0.50	0.59	0.52	5.88	5.52	5.43
	Hk2	1.08	2.15	1.56	5.84	5.95	5.91
	Mthfd1l	1.05	2.27	1.06	6.95	6.91	6.83
	Psat1	1.06	1.61	1.53	9.46	9.15	9.05
	Isyna1	3.48	2.95	2.50	8.99	8.00	8.43
	Pkm	3.81	4.56	4.31	9.91	9.61	9.46
	Dctd	0.27	0.15	0.16	5.74	5.32	5.02
	Phgdh	0.48	0.33	0.87	9.23	9.34	9.11
	Ggt1	0.13	0.08	0.47	7.52	7.26	7.08
	Car12	0.05	0.02	0.07	6.71	6.18	6.16
Chromatin Structure and Remodeling	*Uhrf1*	0.74	1.18	0.70	7.16	7.34	7.14
	Hist1h4f	0.00	2.77	2.19	0.33	6.82	6.46
	Hist2h2ac	0.12	3.20	3.20	0.26	6.47	6.21
	Hist1h3e	0.07	3.71	3.72	0.49	7.30	7.09
	Hist1h3a	0.02	2.26	1.98	0.41	6.12	5.87
	Hist1h2ab	0.00	1.40	1.05	0.18	5.71	5.45
	Hist2h2bb	0.10	3.21	2.65	0.37	7.53	7.44
	Hist1h3f	0.13	3.08	3.01	0.23	7.20	6.90
	Hist1h1a	0.01	2.26	1.66	0.65	6.88	6.70
	Hist1h2be	0.61	2.65	2.66	0.69	5.92	5.64
	Hist1h4d	0.08	5.08	5.39	0.53	8.01	7.78
	Hist4h4	0.15	3.75	3.87	0.64	6.50	6.27
	Hist1h4h	0.50	6.03	6.16	0.77	8.15	7.88
	Hist1h2bb	0.03	3.00	2.40	0.30	7.37	7.16
Cell Cycle	*Rbm38*	2.08	1.20	1.14	6.97	6.22	6.00
	Fancd2	0.55	0.78	0.35	4.90	5.41	5.05
	Bex4	0.58	1.10	0.34	7.16	6.65	6.71
	Cdca7l	0.46	0.64	0.38	6.24	6.19	5.94
	Igf2os	0.03	0.01	0.03	5.62	4.87	4.94
	Trim71	0.13	0.16	0.25	5.76	6.99	6.55
Microtubule and Actin Dynamics	*Obsl1*	1.14	0.85	1.19	7.12	7.06	7.11
	Spire1	0.81	1.45	1.22	6.39	6.36	6.15
Other	*Igdcc4*	0.31	0.26	0.49	6.19	6.18	5.74
	Ydjc	0.45	0.47	0.60	6.56	6.30	6.08
	Atp8a2	0.11	0.45	0.33	4.42	5.45	4.94
	Dusp9	0.18	0.22	0.27	8.05	8.06	7.64
	Bex1	0.24	0.21	0.20	8.13	7.61	7.74
	Dlk1	0.04	0.01	0.04	6.68	5.67	4.52
	St6galnac4	1.87	1.49	1.24	6.38	6.20	5.99
	Exo1	0.30	0.42	0.30	5.51	5.70	5.62
	Ngfrap1	0.73	0.83	0.69	6.72	6.30	6.05
	Mybl2	0.95	0.39	0.37	7.28	6.67	6.24
	Bzw2	3.31	3.28	3.40	7.66	7.50	7.32
	Fam169a	0.71	0.46	0.88	4.99	5.11	5.46
	Smpdl3b	0.42	0.56	0.53	5.39	5.41	5.02

$Log_2(CPM+1)$: 0 – 10

S4 Fig. The 50 most highly de-regulated transcripts that distinguish livers and tumors. Transcripts are grouped as shown in Fig. 3A with mean expression values shown for each group.
