# Supplementary material for: Inhibition of hepatocellular carcinoma by metabolic normalization
Source: PLoS One. 2019 Jun 26;14(6):e0218186. doi: 10.1371/journal.pone.0218186 (PMC6594671; doi:10.1371/journal.pone.0218186)
Supplement: S5 Fig — The histone transcripts from Fig 3A are indicated at the top of the heat map in bold-faced print and were among the 50 most dysregulated transcripts. The vast majority of histone H2 member transcripts were expressed at extremely low-undetectable levels in livers, were not significantly up-regulated in response to dietary intervention or transformation and are therefore not included in this heat map. (PDF) [file pone.0218186.s005.pdf]

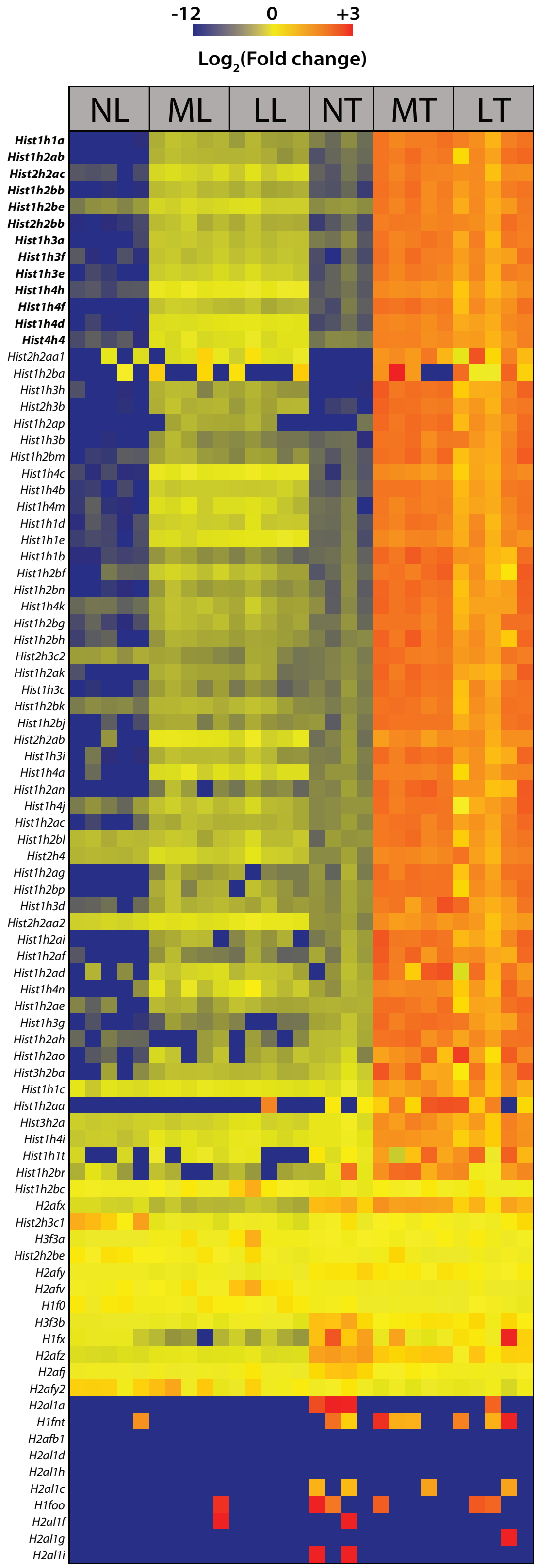

S5 Fig. Synergistic up-regulation of transcripts encoding the majority of histones in HFD tumors. The histone transcripts from Fig. 3A are indicated at the top of the heat map in bold-faced print and were among the 50 most dysregulated transcripts. The vast majority of histone H2 member transcripts were expressed at extremely low-undetectable levels in livers, were not significantly up-regulated in response to dietary intervention or transformation and are therefore not included in this heat map.
