# Supplementary material for: Inhibition of hepatocellular carcinoma by metabolic normalization
Source: PLoS One. 2019 Jun 26;14(6):e0218186. doi: 10.1371/journal.pone.0218186 (PMC6594671; doi:10.1371/journal.pone.0218186)
Supplement: S6 Fig — A, Transcripts related to cell cycle progression. B, Transcripts related to chromatin structure and remodeling. C, Transcripts related to metabolism. D Transcripts related to RNA processing. E, Transcripts related to ubiquitylation. See S3 Table for a full list of these and the remaining members of the 993 common transcript group (Fig 3C). (PDF) [file pone.0218186.s006.pdf]

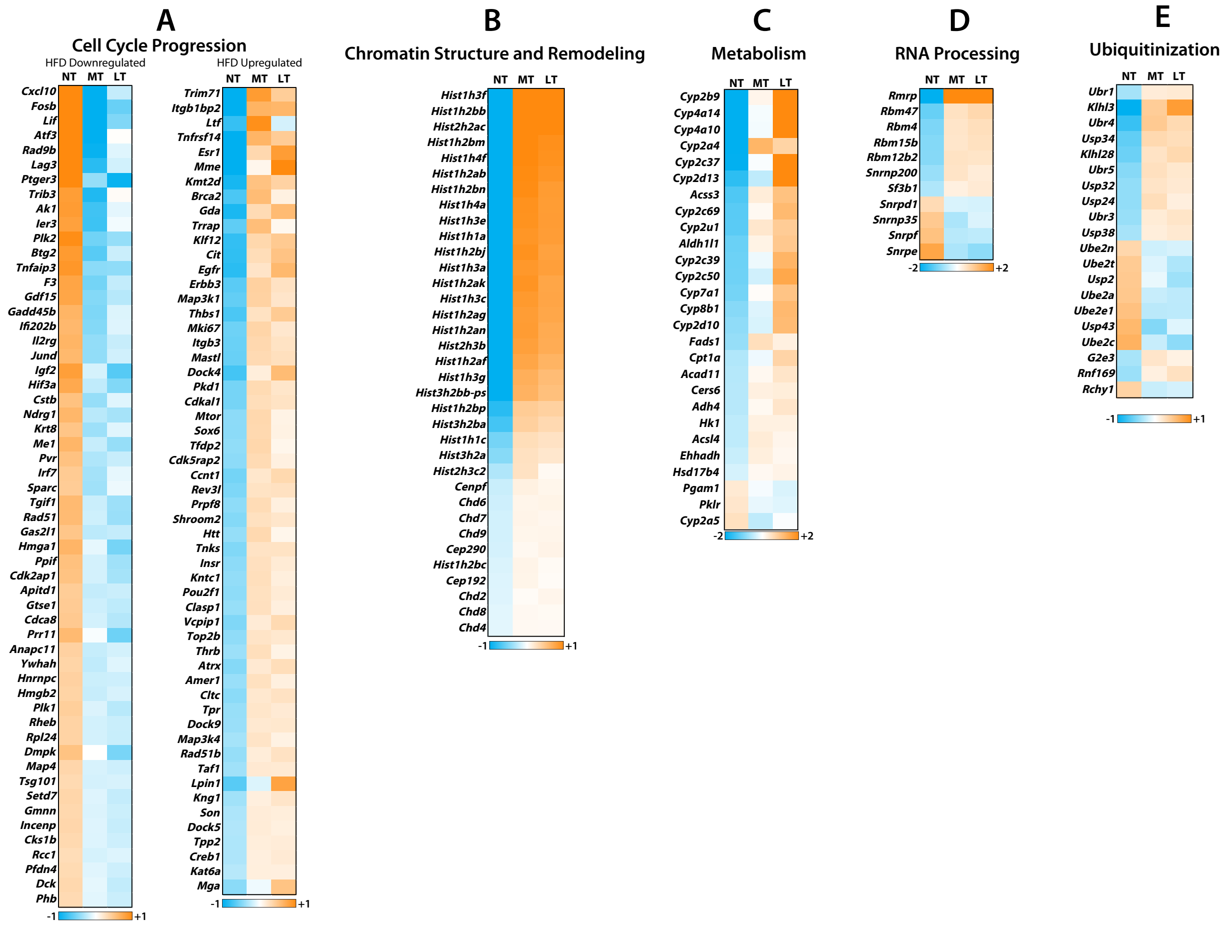

S6 Fig. Heat maps of transcripts from Fig. 3C showing expression of genes that are altered only in tumors from mice maintained HFDs of either type. A, Transcripts related to cell cycle progression. **B,** Transcripts related to chromatin structure and remodeling. **C,** Transcripts related to metabolism. D Transcripts related to RNA processing. **E,** Transcripts related to ubiquitylation. See S3 Table for a full list of these and the remaining members of the 993 common transcript group (Fig. 3C).
