# Supplementary material for: Inhibition of hepatocellular carcinoma by metabolic normalization
Source: PLoS One. 2019 Jun 26;14(6):e0218186. doi: 10.1371/journal.pone.0218186 (PMC6594671; doi:10.1371/journal.pone.0218186)
Supplement: S7 Fig — t-SNE patterns for the transcripts listed in Fig 2J were calculated from TCGA expression profiles and displayed as previously described (blue dots) [17]. Where available, t-SNE patterns for matched normal human tissues were similarly calculated and plotted (red dots). Kaplan-Meier survival data for each of the tumor cohorts were then plotted as shown in Fig 4E. (PDF) [file pone.0218186.s007.pdf]

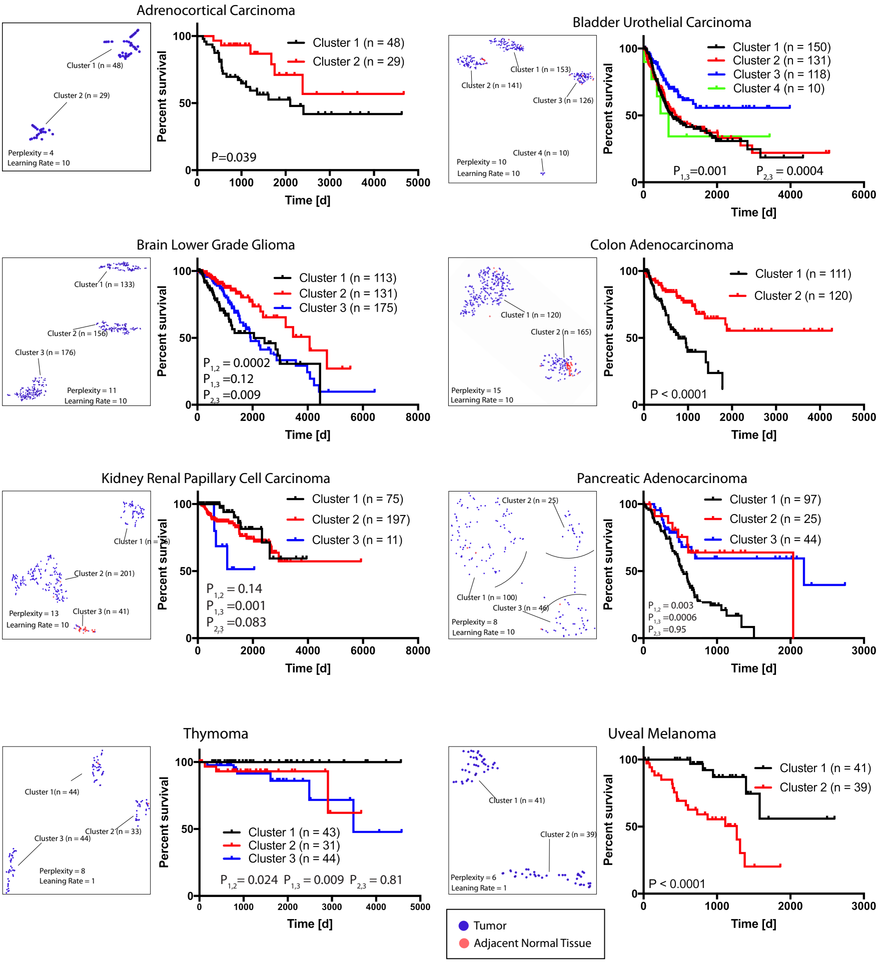

S7 Fig. t-SNE analysis of cholesterol-related transcripts identifies distinct tumor cohorts that correlate with patient survival. t-SNE patterns for the transcripts listed in Fig. 2J were calculated from TCGA expression profiles and displayed as previously described (blue dots) [17]. Where available, t-SNE patterns for matched normal human tissues were similarly calculated and plotted (red dots). Kaplan-Meier survival data for each of the tumor cohorts were then plotted as shown in Fig. 4E.
