# Supplementary material for: Inhibition of hepatocellular carcinoma by metabolic normalization
Source: PLoS One. 2019 Jun 26;14(6):e0218186. doi: 10.1371/journal.pone.0218186 (PMC6594671; doi:10.1371/journal.pone.0218186)
Supplement: S8 Fig — Each of the histograms indicates the transcripts that were the most deterministic of the patterns depicted in S7 Fig. (PDF) [file pone.0218186.s008.pdf]

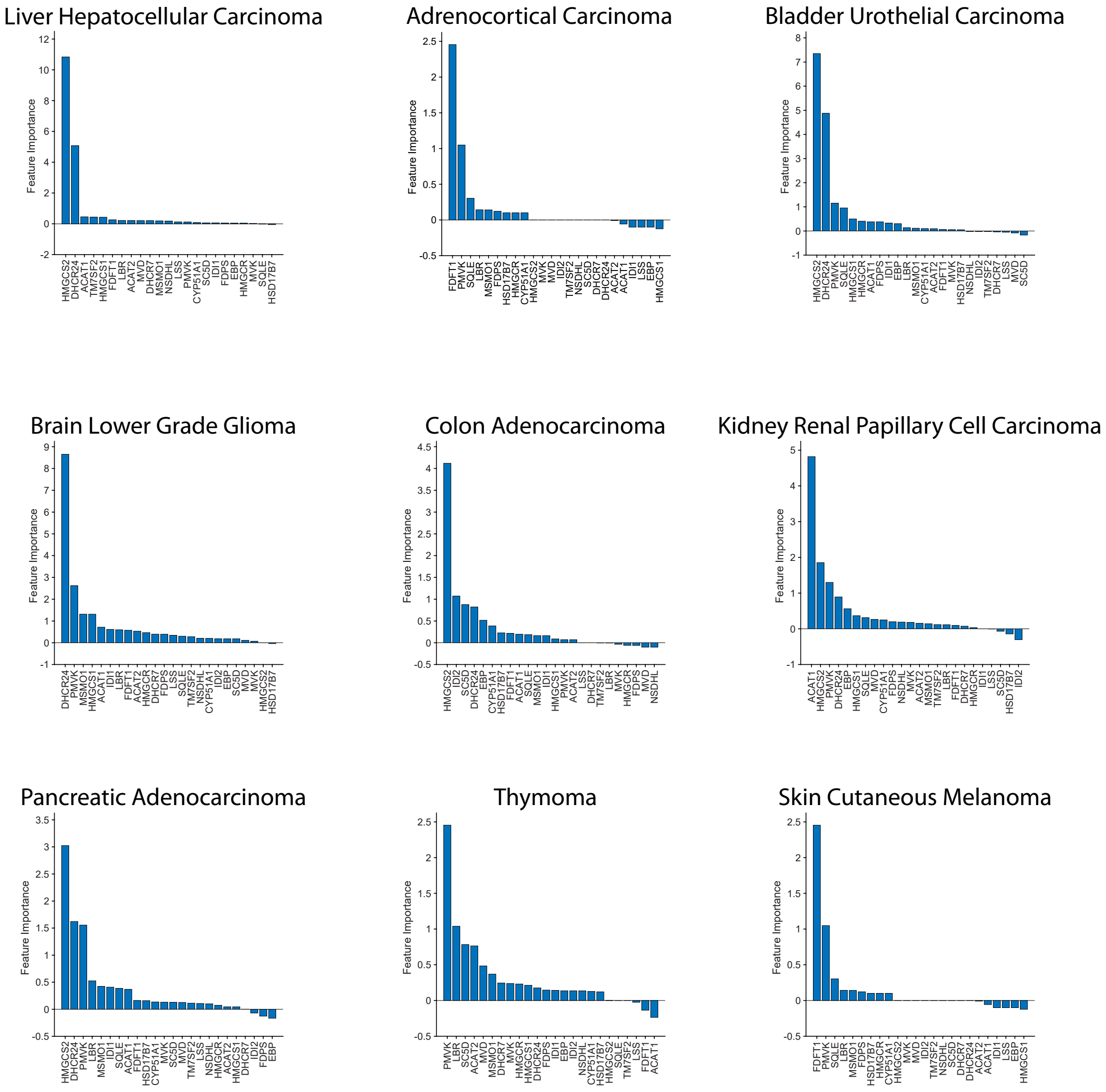

S8 Fig. Random Forest classification of cholesterol biosynthesis-related transcripts most responsible for t-SNE clustering patterns in nine tumors. Each of the histograms indicates the transcripts that were the most deterministic of the patterns depicted in S7 Fig.
