# Supplementary material for: Inhibition of hepatocellular carcinoma by metabolic normalization
Source: PLoS One. 2019 Jun 26;14(6):e0218186. doi: 10.1371/journal.pone.0218186 (PMC6594671; doi:10.1371/journal.pone.0218186)
Supplement: S9 Fig — Data from TCGA were analyzed as described in Materials and Methods. Points on the scattergrams represent the mean expression levels for both FAO-related transcripts and glycolysis-related transcripts in each sample as depicted in Fig 4F. (PDF) [file pone.0218186.s009.pdf]

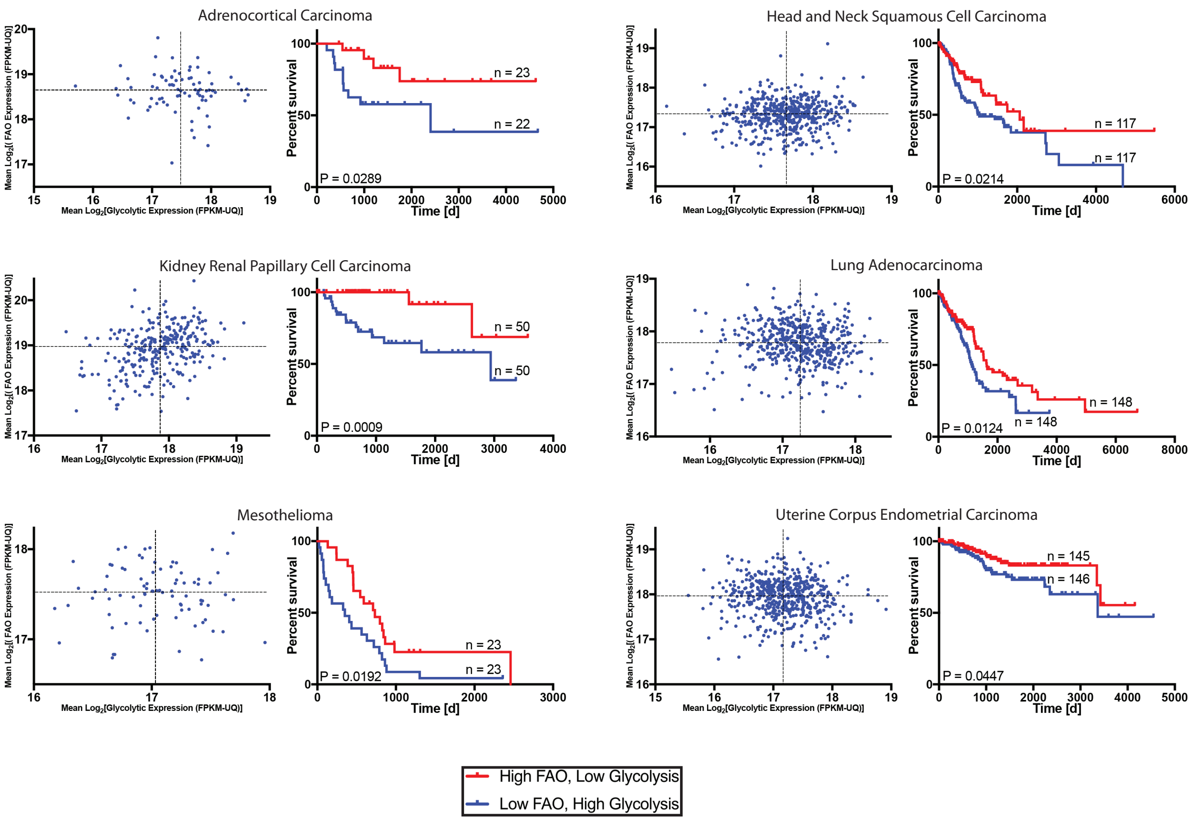

S9 Fig. Distribution of FAO- and glycolysis-related transcripts and Kaplan-Meier survival curves as depicted in Fig. 4F&G for six other human cancers. Data from TCGA were analyzed as described in Materials and Methods. Points on the scattergrams represent the mean expression levels for both FAO-related transcripts and glycolysis-related transcripts in each sample as depicted in Fig. 4F.
