# Supplementary material for: Inhibition of hepatocellular carcinoma by metabolic normalization
Source: PLoS One. 2019 Jun 26;14(6):e0218186. doi: 10.1371/journal.pone.0218186 (PMC6594671; doi:10.1371/journal.pone.0218186)
Supplement: S10 Fig — t-SNE for the FAO transcripts depicted in S2B & S2C Fig were analyzed in TCGA tumor types. Kaplan-Meier survival curves were then plotted for each of the clusters where significant survival differences for the indicated tumor type were observed. (PDF) [file pone.0218186.s010.pdf]

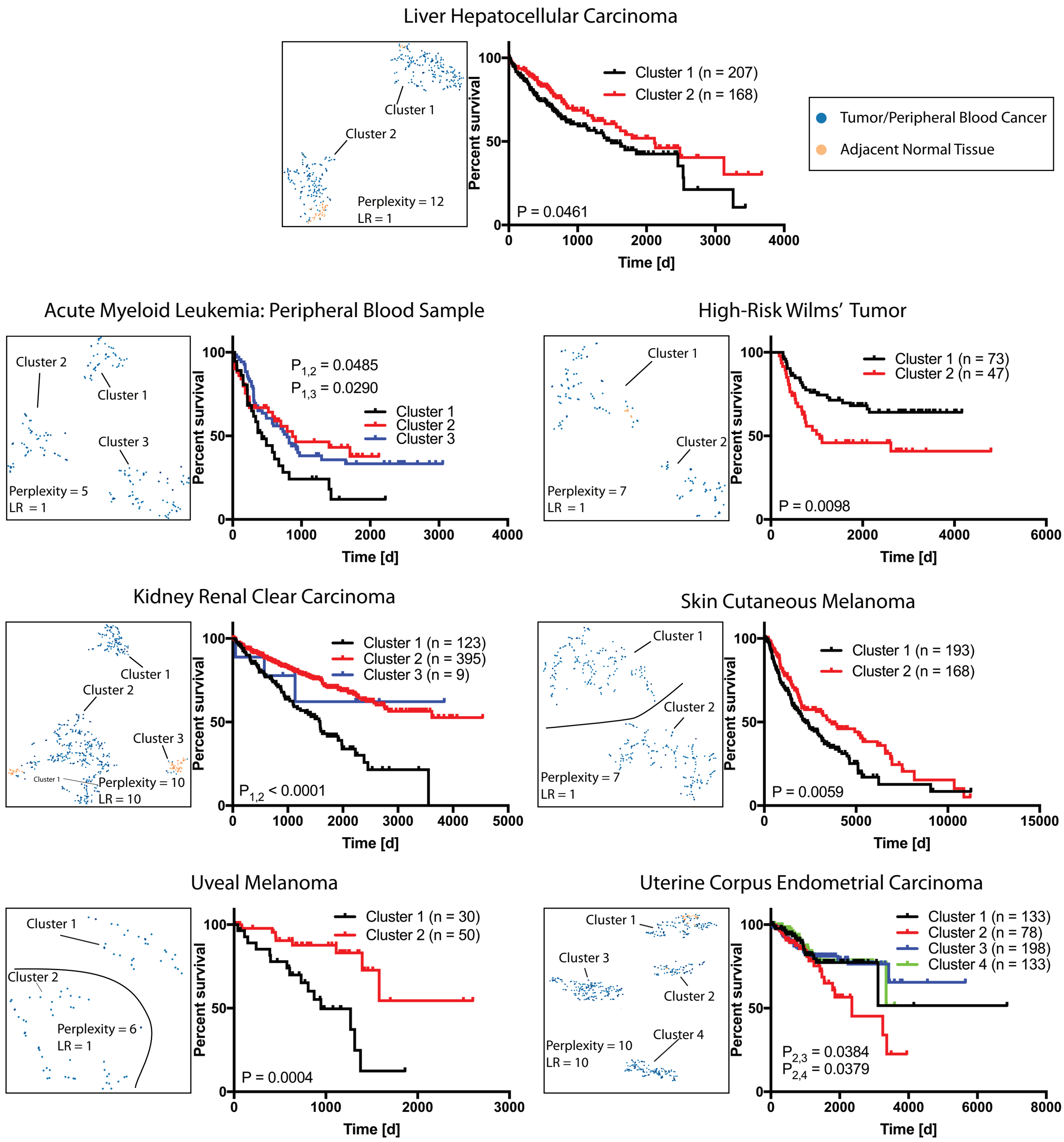

S10 Fig. t-SNE analysis of FAO-related transcripts identifies distinct tumor cohorts that correlate with patient survival. t-SNE for the FAO transcripts depicted in S2B&C Fig were analyzed in TCGA tumor types. Kaplan-Meier survival curves were then plotted for each of the clusters where significant survival differences for the indicated tumor type were observed.
