# Supplementary material for: Inhibition of hepatocellular carcinoma by metabolic normalization
Source: PLoS One. 2019 Jun 26;14(6):e0218186. doi: 10.1371/journal.pone.0218186 (PMC6594671; doi:10.1371/journal.pone.0218186)
Supplement: S11 Fig — Each of the histograms indicates those transcripts which were the most deterministic of the patterns depicted in S10 Fig. (PDF) [file pone.0218186.s011.pdf]

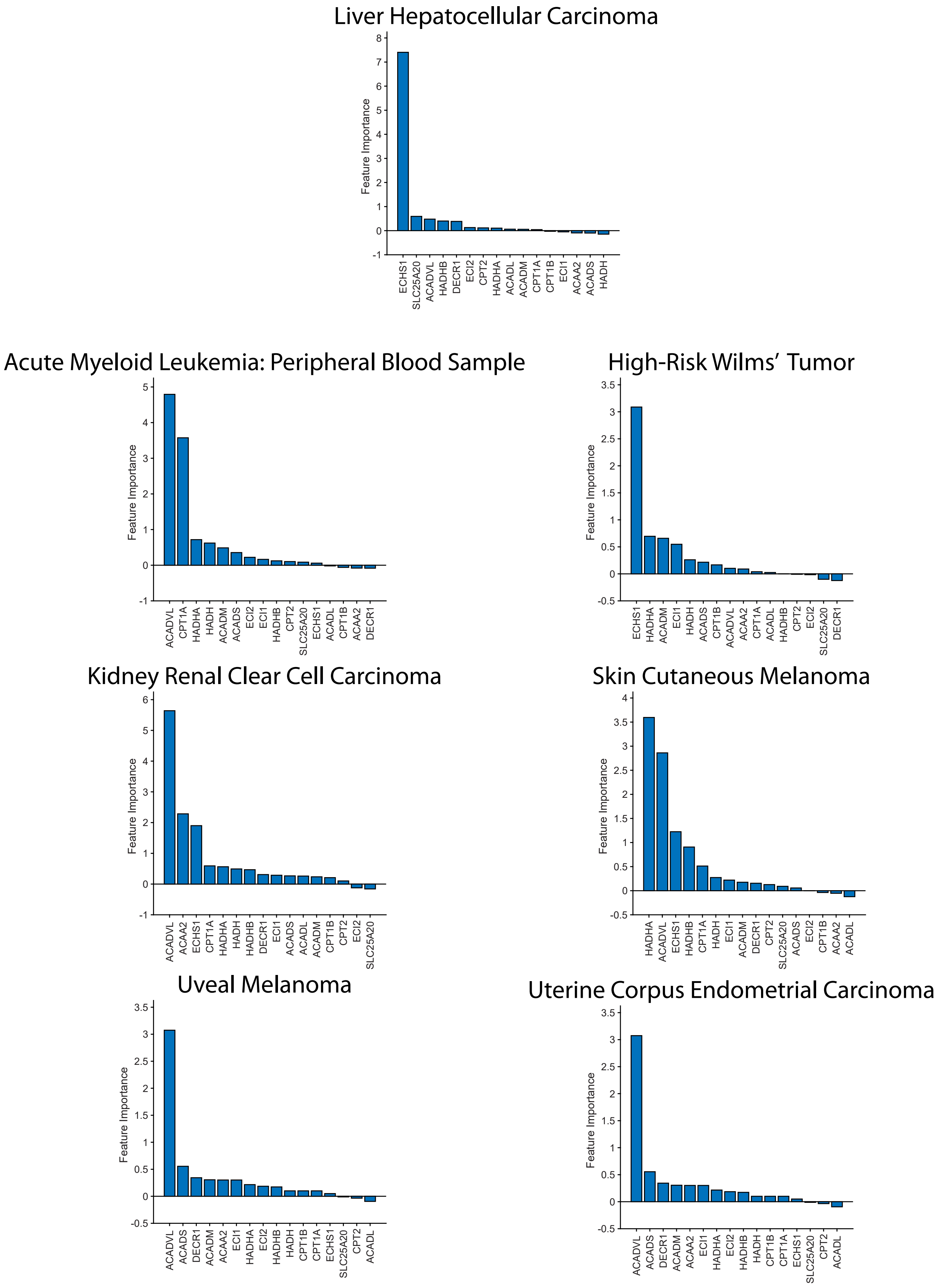

S11 Fig. Random Forest classification of FAO-related transcripts most responsible for t-SNE clustering patterns.

Each of the histograms indicates those transcripts which were the most deterministic of the patterns depicted in S10 Fig.
