# Supplementary material for: Inhibition of hepatocellular carcinoma by metabolic normalization
Source: PLoS One. 2019 Jun 26;14(6):e0218186. doi: 10.1371/journal.pone.0218186 (PMC6594671; doi:10.1371/journal.pone.0218186)
Supplement: S1 Table — (PDF) [file pone.0218186.s012.pdf]

S1 Table. Antibodies and conditions employed for the current study

Name of Protein	Antibody vendor and catalog no.	Antibody concentration used
c-Myc	Santa Cruz sc-40 clone 9E10	1:1000
N-Myc	Santa Cruz sc-791	1:500
L-Myc	Santa Cruz sc-790	1:500
PKM1	Cell Signaling #7076	1:1000
PKM2	Cell Signaling #3198	1:1000
PDH	Santa Cruz SC-377092	1:1000
pPDH	Calbiochem #ap1062	1:2000
Cpt1a	Abcam #128568	1:1000
ChREBP	Novusbio #NB500-135	1:500
MondoA	Bethyl Laboratories #A303-195A-M	1:1000
Glut1	Abcam #115730	1:15000
GAPDH	Sigma #G8795	1:10000
HRP-anti mouse	Cell Signaling #7076	1:10000
HRP-anti rabbit	Cell Signaling #7074	1:5000
