# Supplementary material for: Inhibition of hepatocellular carcinoma by metabolic normalization
Source: PLoS One. 2019 Jun 26;14(6):e0218186. doi: 10.1371/journal.pone.0218186 (PMC6594671; doi:10.1371/journal.pone.0218186)
Supplement: S2 Table — (PDF) [file pone.0218186.s013.pdf]

# S2 Table. Functional categories of the top 50 most dysregulated transcripts

**Supplementary Table S2: Major Functional Categories of the Proteins Encoded by the 50 Most De-regulated Transcripts in Livers and Tumors (Fig. 3A)**

**A. METABOLISM**

1. Aldh18a1: Alcohol dehydrogenase 18a1. Catalyzes the reduction of glutamate to delta1-pyrroline-5-carboxylate, a critical step in the de novo biosynthesis of proline, ornithine and arginine
2. PFK-phosphofructokinase. Glycolytic enzyme Catalyzes fructose-6-phosphate to fructose-1,6,bisphosphate
3. ME2-malic enzyme 2
4. IMPDH1: Inosine monophosphate dehydrogenase: catalyzes the synthesis of xanthine monophosphate (XMP) from inosine-5'-monophosphate (IMP). The rate-limiting step in the de novo synthesis of guanine nucleotides.
5. SLC1A5: Na+-dependent neutral amino acid transporter
6. SLC7A6: Participates in the sodium-independent uptake of dibasic amino acids and sodium-dependent uptake of some neutral amino acids.
7. Hexokinase 2: -Glycolytic enzyme. Converts glucose to glucose-6-phosphate
8. MTHFD1L: Methyltetrahydroflate dehydrogenase. Involved in the synthesis of tetrahydrofolate (THF) in the mitochondrion. Involved in the synthesis of purines and thymidylate and the regeneration of methionine from homocysteine.
9. Psat1: A class-V pyridoxal-phosphate-dependent aminotransferase. Catalyzes 3-phosphohydroxypyruvate to phosphoserine and of 3-hydroxy-2-oxo-4-phosphonooxybutanoate to phosphohydroxythreonine.
10. Isyna1: An inositol-3-phosphate synthase. Catalyzes the rate-limiting conversion of glucose 6-phosphate to myoinositol 1-phosphate.
11. PKM: Glycolytic enzyme
12. DCTD: DCMP deaminase. Deaminates dCMP to dUMP, the substrate for thymidylate synthase.
13. Phgdh: Phosphoglycerate dehydrogenase. Involved in initial steps of L-serine synthesis.
14. Ggt1: Gamma-Glutamyltransferase 1: A type I gamma-glutamyltransferase that catalyzes the transfer of the glutamyl moiety of glutathione to amino acid and dipeptide acceptors.
15. Car12: carbonic anhydrase. Catalyzes the hydration of carbon dioxide to bicarbonate.

**B. CHROMATIN STRUCTURE AND REMODELING**

1. Uhrf1-A RING-finger type E3 ubiquitin ligase. Binds to specific DNA sequences, and recruits HDAC to regulate gene expression. Expression peaks at late G1 phase and continues during G2/M. Ha role G1/S transition by regulating topoisomerase IIalpha and Rb expression, and functions in the p53-dependent DNA damage checkpoint. A hub protein for the integration of epigenetic information. Up-regulated in various cancers.
2. Histone 1h4f
3. Histone 2h2ac
4. Histone 1h3e
5. Histone 1h3a
6. Histone 1h2ab
7. Histone 2h2bb
8. Histone 1h3f
9. Histone 1h1a
10. Histone 1h2be
11. Histone 1h4d
12. Histone 4h4
13. Histone 1h4h
14. Histone 1h2bb

**C. CELL CYCLE**

1. Rbm 38: Binds the 3’-UTR of the TP53-induced CDKN1A transcripts and stabilizes them.
2. FANCD2: Required for maintenance of chromosomal stability. Helps to repair of double-stranded DNA breaks by homologous recombination and single-strand annealing. Possible role in S and G2 checkpoint activation following DNA damage.
3. BEX4: May play a role in microtubule deacetylation and cell cycle control by negatively regulating SIRT2 deacetylase toward alpha-tubulin.
4. CDC7L: Cell division cycle protein with kinase activity that is critical for the G1/S transition.
5. Igf2-AS: Possible tumor suppressor.
6. Trim7: An E3 ubiquitin-protein ligase. Helps to regulate JUN transactivation and cellular proliferation.

**D. MICROTUBLE/ACTIN DYNAMICS**

1. OBSL1: Cytoskeletal adaptor proteins that participates in the linking of the internal cytoskeleton of cells to the cell membrane.
2. PIRE1: Required for asymmetric spindle positioning and asymmetric cell division during meiosis.
3. Spire 1: Involved in actin organization.
