# Supplementary material for: Inhibition of hepatocellular carcinoma by metabolic normalization
Source: PLoS One. 2019 Jun 26;14(6):e0218186. doi: 10.1371/journal.pone.0218186 (PMC6594671; doi:10.1371/journal.pone.0218186)
Supplement: S3 Table — The transcripts depicted in S6 Fig are listed at the top of the Table. (PDF) [file pone.0218186.s014.pdf]

S3 Table. XLS sheet of all 993 genes overlap shown in Fig. 3C. The 34% of transcripts depicted in the heat maps of Suppl. Fig. 6 are listed at the top of the Table

Symbol	Category	Subcategory	Entrez ID	NL (FPKM)	ML (FPKM)	LL (FPKM)	NT (FPKM)	MT (FPKM)	LT (FPKM)
Cxcl10	Cell cycle	Cell cycle progression	15945	2.35751	2.00845	1.69551	54.8861	4.54222	12.4506
Fosb	Cell cycle	Cell cycle progression	14282	0.0798376	0.0133713	0.043339	1.30044	0.142629	0.233222
Lif	Cell cycle	Cell cycle progression	16878	0.0205198	0.0241065	0.00784607	0.854523	0.104233	0.177832
Atf3	Cell cycle	Cell cycle progression	11910	1.21319	2.28101	1.09448	33.1324	5.41576	13.6295
Rad9b	Cell cycle	Cell cycle progression	231724	1.19535	0.866781	0.982535	8.0012	1.91883	3.43943
Lag3	Cell cycle	Cell cycle progression	16768	0.654209	0.129507	0.359451	0.480488	0.132842	0.210277
Ptger3	Cell cycle	Cell cycle progression	19218	0.0886496	0.156577	0.219686	1.44421	0.416975	0.285461
Trib3	Cell cycle	Cell cycle progression	228775	2.23757	1.68562	2.90959	7.95019	2.42909	4.56479
Ak1	Cell cycle	Cell cycle progression	11636	0.178721	0.138948	0.146024	1.30269	0.430231	0.67281
Ier3	Cell cycle	Cell cycle progression	15937	0.37241	1.25483	0.534708	2.80591	0.949057	1.54942
Plk2	Cell cycle	Cell cycle progression	20620	9.023	8.3524	9.39193	15.3482	5.3282	5.8762
Btg2	Cell cycle	Cell cycle progression	12227	4.67051	15.0536	3.93685	17.9987	6.30693	8.59636
Tnfaip3	Cell cycle	Cell cycle progression	21929	0.843449	0.91903	0.804763	4.81276	1.8972	1.91824
F3	Cell cycle	Cell cycle progression	14066	3.60826	3.11422	3.83257	1.39152	0.55814	0.691545
Gdf15	Cell cycle	Cell cycle progression	23886	5.23838	3.77479	2.17637	19.5244	8.24083	9.43952
Gadd45b	Cell cycle	Cell cycle progression	17873	1.49397	1.3648	0.498092	35.815	16.1983	20.9224
Ifi202b	Cell cycle	Cell cycle progression	26388	1.59623	4.40457	1.77064	2.80718	1.32009	1.68476
Il2rg	Cell cycle	Cell cycle progression	16186	0.973821	1.53185	0.836145	2.05154	0.981307	1.13207
Jund	Cell cycle	Cell cycle progression	16478	26.3724	21.2172	19.3713	51.2187	25.0527	29.6785
Igf2	Cell cycle	Cell cycle progression	16002	0.52471	0.0931806	0.628001	988.944	500.018	354.069
Hif3a	Cell cycle	Cell cycle progression	53417	0.0310494	0.0171117	0.0185391	16.3567	8.29462	6.97649
Cstb	Cell cycle	Cell cycle progression	13014	73.6692	105.028	87.99	83.0403	43.337	52.8644
Ndrg1	Cell cycle	Cell cycle progression	17988	12.1802	5.78224	9.76786	86.6274	46.709	44.2921
Krt8	Cell cycle	Cell cycle progression	16691	99.3075	101.968	76.5828	178.777	97.0929	116.541
Me1	Cell cycle	Cell cycle progression	17436	61.7134	161.383	32.2446	7.95435	4.40877	3.87259
Pvr	Cell cycle	Cell cycle progression	52118	3.8385	3.03808	3.10126	8.63716	4.83531	5.16156
Irf7	Cell cycle	Cell cycle progression	54123	9.11147	16.4095	9.04305	11.3165	6.4694	7.73005
Sparc	Cell cycle	Cell cycle progression	20692	71.1364	38.64	57.3865	63.0397	36.0678	44.4853
Tgif1	Cell cycle	Cell cycle progression	21815	12.5733	10.0681	9.17216	16.8054	9.66851	8.54997
Rad51	Cell cycle	Cell cycle progression	19361	0.353028	0.270504	0.150463	24.7732	14.5067	12.6052
Gas2l1	Cell cycle	Cell cycle progression	78926	9.81098	8.44011	5.46334	10.3193	6.12615	6.26113
Hmga1	Cell cycle	Cell cycle progression	15361	1.54126	1.87008	0.856709	37.148	22.3665	16.5041
Ppif	Cell cycle	Cell cycle progression	105675	33.2248	25.8665	19.2362	154.095	94.3955	82.1615
Cdk2ap1	Cell cycle	Cell cycle progression	13445	6.18923	4.26656	4.91378	16.6094	10.2901	9.06882
Apitd1	Cell cycle	Cell cycle progression	69928	0.207498	0.273534	0.111165	10.2385	6.47585	6.58001
Gtse1	Cell cycle	Cell cycle progression	29870	0.484594	0.31004	0.222985	18.1772	11.6766	11.2074
Cdca8	Cell cycle	Cell cycle progression	52276	1.40155	0.929021	0.393875	53.7639	34.6394	31.8709
Prr11	Cell cycle	Cell cycle progression	270906	0.178179	0.160507	0.0449635	4.00246	2.60893	1.79205
Anapc11	Cell cycle	Cell cycle progression	66156	4.63555	4.03735	5.1531	6.76569	4.41428	4.57494

S3 Table. Continue...

Ywhah	Cell cycle	Cell cycle progression	22629	16.079	14.355	10.8907	39.2556	25.7099	27.9416
Hnrnpc	Cell cycle	Cell cycle progression	15381	40.8251	26.565	33.1114	94.0281	62.2201	62.6122
Hmgb2	Cell cycle	Cell cycle progression	97165	0.815646	0.93611	0.827388	30.8717	20.4477	21.3577
Plk1	Cell cycle	Cell cycle progression	18817	0.515616	0.605408	0.04896	43.0985	29.2612	26.5952
Rheb	Cell cycle	Cell cycle progression	19744	39.4933	28.4909	36.0737	68.648	46.7444	45.6183
Rpl24	Cell cycle	Cell cycle progression	68193	148.322	186.025	162.423	391.868	267.74	259.843
Dmpk	Cell cycle	Cell cycle progression	13400	8.92875	6.92378	3.61059	8.85346	6.19527	4.29181
Map4	Cell cycle	Cell cycle progression	17758	12.4541	10.0608	8.639	23.4433	16.494	16.0376
Tsg101	Cell cycle	Cell cycle progression	22088	22.283	15.8768	18.5244	19.2981	13.7391	13.8422
Setd7	Cell cycle	Cell cycle progression	73251	2.08484	2.61637	1.568	5.26204	3.76562	3.5099
Gmnn	Cell cycle	Cell cycle progression	57441	9.36174	12.1946	9.79908	49.6095	35.5777	34.1032
Incenp	Cell cycle	Cell cycle progression	16319	1.14538	0.773024	0.666986	28.8044	20.6759	19.352
Cks1b	Cell cycle	Cell cycle progression	54124	13.3362	9.9589	12.8716	87.4412	63.4644	60.7691
Rcc1	Cell cycle	Cell cycle progression	100088	4.67736	3.23512	3.38564	63.8135	46.3359	47.3564
Pfdn4	Cell cycle	Cell cycle progression	109054	11.0313	22.0999	17.0273	72.8532	53.8249	51.103
Dck	Cell cycle	Cell cycle progression	13178	2.75543	3.33051	2.47433	14.1473	10.469	9.50016
Phb	Cell cycle	Cell cycle progression	18673	26.1269	19.9973	21.2769	52.7365	39.178	36.7404
Mga	Cell cycle	Cell cycle progression	29808	3.83618	4.61797	5.40552	10.9406	14.3493	21.3112
Kat6a	Cell cycle	Cell cycle progression	244349	4.67924	4.1011	4.66858	7.20295	9.66471	9.63949
Creb1	Cell cycle	Cell cycle progression	12912	3.64662	4.32389	5.03119	5.67301	7.77489	8.00478
Tpp2	Cell cycle	Cell cycle progression	22019	18.9924	18.2677	21.6678	17.7943	24.5623	24.8115
Dock5	Cell cycle	Cell cycle progression	68813	3.97761	3.21258	3.80796	3.24989	4.51447	4.40424
Son	Cell cycle	Cell cycle progression	20658	33.1033	25.9905	29.5471	27.5418	38.5084	38.1353
Kng1	Cell cycle	Cell cycle progression	16644	1269.43	1400.95	1557.52	188.909	268.269	284.589
Lpin1	Cell cycle	Cell cycle progression	14245	21.9506	38.3855	20.4391	3.80914	5.46082	10.3505
Taf1	Cell cycle	Cell cycle progression	270627	4.45926	5.93541	5.5907	4.85525	7.15996	7.13324
Rad51b	Cell cycle	Cell cycle progression	19363	1.80882	20.279	1.54063	4.47732	6.64749	7.07217
Map3k4	Cell cycle	Cell cycle progression	26407	6.30492	8.06311	8.09442	10.3134	15.4565	14.284
Dock9	Cell cycle	Cell cycle progression	105445	3.11002	4.7258	3.71514	4.98154	7.48545	7.54661
Tpr	Cell cycle	Cell cycle progression	108989	13.4839	17.1073	16.1485	35.4528	54.5988	53.1353
Cltc	Cell cycle	Cell cycle progression	67300	61.8093	76.2764	73.4435	53.7823	84.6517	87.6552
Amer1	Cell cycle	Cell cycle progression	72345	1.4235	1.32879	1.68028	2.16352	3.42698	3.13778
Atrx	Cell cycle	Cell cycle progression	22589	9.02525	10.5132	10.0374	11.2744	17.8919	19.2024
Thrb	Cell cycle	Cell cycle progression	21834	10.66	10.4289	11.8406	2.13596	3.39248	3.01422
Top2b	Cell cycle	Cell cycle progression	21974	15.4396	18.2605	21.4321	16.3065	25.9039	25.3708
Vcpip1	Cell cycle	Cell cycle progression	70675	10.4706	11.4706	13.9779	7.17192	11.4038	12.646
Clasp1	Cell cycle	Cell cycle progression	76707	3.47902	3.45028	3.42437	4.74455	7.5493	6.91285
Pou2f1	Cell cycle	Cell cycle progression	18986	1.34917	1.41857	1.48281	3.33368	5.42075	5.11462
Kntc1	Cell cycle	Cell cycle progression	208628	0.113894	0.264277	0.0620975	6.66894	10.8508	9.85564
Insr	Cell cycle	Cell cycle progression	16337	21.2925	22.671	22.7179	11.1361	18.2643	17.1114
Tnks	Cell cycle	Cell cycle progression	21951	5.3393	5.02989	6.1812	6.58274	10.9455	10.9771
Htt	Cell cycle	Cell cycle progression	15194	4.25918	4.98371	4.93318	7.42999	12.4233	10.4949

S3 Table. Continue...

Shroom2	Cell cycle	Cell cycle progression	110380	5.0209	5.23519	6.97367	3.12267	5.22571	5.06335
Prpf8	Cell cycle	Cell cycle progression	192159	43.5594	39.2532	44.086	55.2284	92.7228	82.4656
Rev3l	Cell cycle	Cell cycle progression	19714	3.86182	3.84707	4.66206	3.18229	5.35887	5.40785
Ccnt1	Cell cycle	Cell cycle progression	12455	5.56666	6.9675	8.30161	6.23265	10.5208	11.4365
Cdk5rap2	Cell cycle	Cell cycle progression	214444	1.1171	1.78215	1.52382	5.55549	9.3924	8.27022
Tfdp2	Cell cycle	Cell cycle progression	211586	3.74831	3.0322	4.08068	9.33648	15.953	13.3171
Sox6	Cell cycle	Cell cycle progression	20679	3.1099	2.87314	3.55766	3.0718	5.26949	4.55461
Mtor	Cell cycle	Cell cycle progression	56717	8.86527	12.8162	9.72813	6.82879	11.774	10.034
Cdkal1	Cell cycle	Cell cycle progression	68916	5.35432	7.28086	7.2316	5.34335	9.46192	9.18635
Pkd1	Cell cycle	Cell cycle progression	18763	3.4549	3.66352	3.79228	2.22578	4.01981	3.76621
Dock4	Cell cycle	Cell cycle progression	238130	6.2259	4.76607	6.55332	0.780951	1.43719	1.93432
Mastl	Cell cycle	Cell cycle progression	67121	0.1324	0.384122	0.139215	2.36972	4.43855	4.3049
Itgb3	Cell cycle	Cell cycle progression	16416	0.560837	1.28788	0.879751	0.846243	1.59835	1.50358
Mki67	Cell cycle	Cell cycle progression	17345	0.622245	1.49314	0.342402	25.9278	49.392	44.3999
Thbs1	Cell cycle	Cell cycle progression	21825	0.38197	0.532145	0.467713	1.23514	2.40234	2.74715
Map3k1	Cell cycle	Cell cycle progression	26401	3.1708	5.08178	3.72592	7.8806	15.7261	14.032
Erbb3	Cell cycle	Cell cycle progression	13867	32.4132	48.3885	40.6087	9.70138	19.981	18.0588
Egfr	Cell cycle	Cell cycle progression	13649	196.271	71.1479	343.081	3.5277	7.38188	9.54937
Cit	Cell cycle	Cell cycle progression	12704	0.115158	0.229504	0.23404	1.28925	2.77032	3.22455
Klf12	Cell cycle	Cell cycle progression	16597	7.50587	7.15713	9.16489	1.15722	2.54796	2.76033
Trrap	Cell cycle	Cell cycle progression	100683	4.59464	5.18345	4.9817	7.50298	17.0008	12.1197
Gda	Cell cycle	Cell cycle progression	14544	11.096	8.29349	10.2592	0.73866	1.70992	2.05796
Brca2	Cell cycle	Cell cycle progression	12190	0.358787	0.684303	0.546527	3.47185	8.41079	6.1699
Kmt2d	Cell cycle	Cell cycle progression	381022	3.29325	2.55196	2.6148	2.70643	7.3066	6.63919
Mme	Cell cycle	Cell cycle progression	17380	5.84434	15.3215	6.53528	0.142545	0.392141	0.91165
Esr1	Cell cycle	Cell cycle progression	13982	5.47002	9.50399	6.86553	0.179773	0.510912	0.725638
Tnfrsf14	Cell cycle	Cell cycle progression	230979	4.4764	5.50207	6.05195	1.4725	4.83437	4.2058
Ltf	Cell cycle	Cell cycle progression	17002	0.0198347	0.00806038	0.0592014	2.94185	9.82589	4.53018
Itgb1bp2	Cell cycle	Cell cycle progression	26549	0.0808137	0.162565	0.127081	0.167747	0.633799	0.622714
Trim71	Cell cycle	Cell cycle progression	636931	0.00611795	0.0118489	0.023358	3.0563	13.3628	9.93205
Hist1h3f	Cell cycle	Histone	260423	0.0191911	6.39078	5.85143	0.111673	126.073	105.236
Hist1h2bb	Cell cycle	Histone	319178	0.0290223	23.8672	15.5405	0.516127	504.454	438.907
Hist2h2ac	Cell cycle	Histone	319176	0.0743266	22.9288	23.0932	0.32866	259.825	230.519
Hist1h2bm	Cell cycle	Histone	319186	0.171728	5.94462	2.7551	0.479648	298.758	231.676
Hist1h4f	Cell cycle	Histone	319157	0	38.7197	23.291	1.17135	657.114	515.898
Hist1h2ab	Cell cycle	Histone	319172	0	4.70752	3.12846	0.344445	138.018	116.619
Hist1h2bn	Cell cycle	Histone	319187	0.0430138	3.98769	2.61406	0.791295	270.722	207.853
Hist1h4a	Cell cycle	Histone	326619	0.0758218	44.084	43.043	0.972333	274.215	227.512
Hist1h3e	Cell cycle	Histone	319151	0.0820236	17.1375	15.8341	0.698369	194.281	163.156
Hist1h1a	Cell cycle	Histone	80838	0.0124245	9.93139	5.46006	1.21753	294.814	258.637
Hist1h2bj	Cell cycle	Histone	319183	0	2.24475	1.16767	0.549369	140.673	106.357
Hist1h3a	Cell cycle	Histone	360198	0.0203762	6.69101	5.21541	0.556651	120.564	106.073

S3 Table. Continue...

Hist1h2ak	Cell cycle	Histone	319169	0	6.83214	4.00323	1.44754	322.513	236.217
Hist1h3c	Cell cycle	Histone	319148	0.103835	4.56794	1.72086	1.82442	336.158	274.225
Hist1h2ag	Cell cycle	Histone	319167	0	1.12743	0.418235	0.398569	63.8402	53.6755
Hist1h2an	Cell cycle	Histone	319170	0	0.861398	0.414611	0.449107	68.7092	53.3491
Hist2h3b	Cell cycle	Histone	319154	0	0.336642	0.46768	0.0218712	2.86178	2.28914
Hist1h2af	Cell cycle	Histone	319173	0	0.894456	0.54634	0.764597	71.5931	55.8873
Hist1h3g	Cell cycle	Histone	97908	0.0529124	1.26236	0.587392	3.59084	206.436	164.184
Hist3h2bb-ps	Cell cycle	Histone	382522	0.0293523	0.388478	0.23591	0.232784	11.2208	8.85456
Hist1h2bp	Cell cycle	Histone	319188	0	0.101933	0.11682	0.458004	6.54185	5.98055
Hist3h2ba	Cell cycle	Histone	78303	0.0369786	0.288103	0.127619	0.569869	6.01964	5.14079
Hist1h1c	Cell cycle	Histone	50708	33.5454	45.1998	41.1496	36.5949	205.289	187.991
Hist3h2a	Cell cycle	Histone	319162	2.40454	3.05762	3.7575	6.4541	29.3178	26.3265
Hist2h3c2	Cell cycle	Histone	97114	2.28149	0.633065	0.948851	0.689767	2.19382	1.47306
Cenpf	Cell cycle	Chromatin remodeling	108000	0.139102	0.450102	0.0999664	6.41535	12.888	11.7833
Chd6	Cell cycle	Chromatin remodeling	71389	3.03214	2.82637	3.25058	2.5528	4.57649	4.66914
Chd7	Cell cycle	Chromatin remodeling	320790	2.34313	2.2132	2.66909	4.01888	7.07876	6.80264
Chd9	Cell cycle	Chromatin remodeling	109151	4.58848	4.77413	5.02853	3.39426	5.79496	5.83811
Cep290	Cell cycle	Chromatin remodeling	216274	0.643772	0.8345	0.796026	1.75651	2.83672	3.08008
Hist1h2bc	Cell cycle	Histone	68024	159.293	163.206	237.384	90.0784	149.161	133.956
Cep192	Cell cycle	Chromatin remodeling	70799	0.902445	1.88448	1.23914	7.49093	11.9801	10.7471
Chd2	Cell cycle	Chromatin remodeling	244059	5.63415	7.37787	5.75398	5.22738	7.42963	8.16594
Chd8	Cell cycle	Chromatin remodeling	67772	6.98414	5.15599	5.97996	8.43697	12.101	11.835
Chd4	Cell cycle	Chromatin remodeling	107932	25.4638	22.0237	21.8217	52.334	73.7899	73.1919
Cyp2b9	Metabolism	CYPs	13094	77.2415	106.896	1.12558	0.225175	2.00592	12.4468
Cyp4a14	Metabolism	CYPs	13119	71.0551	145.526	20.0438	2.68198	13.1403	75.2113
Cyp4a10	Metabolism	CYPs	13117	119.341	102.595	62.0441	1.24706	5.54371	29.6492
Cyp2a4	Metabolism	CYPs	13086	70.2593	249.05	0.275768	0.166719	1.61637	1.12989
Cyp2c37	Metabolism	CYPs	13096	98.9245	66.2175	85.7436	2.20962	8.47024	35.3005
Cyp2d13	Metabolism	CYPs	68444	255.998	121.413	220.596	0.84652	1.86542	11.6297
Acss3	Metabolism		380660	13.5322	43.6318	17.1642	0.432678	1.40475	2.33439
Cyp2c69	Metabolism	CYPs	100043108	304.138	28.4813	32.4265	0.229155	0.611403	1.28668
Cyp2u1	Metabolism	CYPs	71519	16.9941	18.0979	78.7664	0.190481	0.623613	0.853672
Aldh1l1	Metabolism		107747	500.869	393.101	406.878	10.3625	26.9798	44.5274
Cyp2c39	Metabolism	CYPs	13098	93.2452	199.688	33.4426	7.00811	13.9951	37.393
Cyp2c50	Metabolism	CYPs	107141	380.618	160.039	389.311	6.54028	11.0895	40.3206
Cyp7a1	Metabolism	CYPs	13122	53.8058	35.7535	41.2255	1.73575	3.80073	7.40489
Cyp8b1	Metabolism	CYPs	13124	198.072	219.673	148.149	2.72278	4.3455	11.9756
Cyp2d10	Metabolism	CYPs	13101	478.213	339.788	599.885	10.1907	15.1322	41.901
Fads1	Metabo,ism		76267	264.662	863.668	323.872	13.9886	36.34	29.6276
Cpt1a	Metabolism	Lipid metabolism	12894	106.632	158.336	134.676	27.9387	38.8716	70.8377
Acad11	Metabolism		102632	165.468	185.066	201.256	18.4631	30.1223	37.7361
Cers6	Metabolism	Lipid metabolism	241447	4.33747	5.78022	3.46376	6.50645	11.5044	11.906

S3 Table. Continue...

Adh4	Metabolism		26876	134.487	114.623	255.194	6.00349	9.61727	12.0967
Hk1	Metabolism		15275	0.913635	1.367	1.10113	1.42656	2.40787	2.41143
Acsl4	Metabolism	Lipid metabolism	50790	24.6434	30.4813	23.2315	5.95779	10.6362	9.51579
Ehhadh	Metabolism	Lipid metabolism	74147	195.055	346.445	105.307	13.5978	22.2135	20.1241
Hsd17b4	Metabolism	Lipid metabolism	15488	236.723	214.312	229.161	54.1627	73.9368	78.3482
Pgam1	Meetabolism		18648	20.1614	17.168	13.1709	48.9461	35.9675	30.9867
Pklr	Metabolism		18770	108.087	120.752	83.5485	21.7928	14.2675	13.5912
Cyp2a5	Metabolism	CYPs	13087	146.698	53.0907	106.957	25.2354	12.4241	17.1806
Rmrp	Post-transcriptional regulation	RNA processing / splicing	19782	3.28425	81958.2	53052.2	4.88978	66374	59242.3
Rbm47	Post-transcriptional regulation	RNA processing / splicing	245945	19.8351	12.329	15.2875	11.2093	20.1726	21.3704
Rbm4	Post-transcriptional regulation	RNA processing / splicing	19653	6.3899	6.89105	8.2768	5.47331	9.12265	9.46287
Rbm15b	Post-transcriptional regulation	RNA processing / splicing	109095	4.87775	6.48205	6.73783	7.50992	12.1753	12.594
Rbm12b2	Post-transcriptional regulation	RNA processing / splicing	77604	1.91282	1.88281	2.52644	1.95186	3.2152	3.17763
Snrnp200	Post-transcriptional regulation	RNA processing / splicing	320632	18.6439	18.9326	19.9952	30.3446	47.0252	44.79
Sf3b1	Post-transcriptional regulation	RNA processing / splicing	81898	30.3864	35.6911	35.8316	36.0959	48.9889	50.712
Snrpd1	Post-transcriptional regulation	RNA processing / splicing	20641	20.1071	17.1355	22.135	157.836	115.034	113.92
Snrnp35	Post-transcriptional regulation	RNA processing / splicing	76167	6.83848	4.0024	5.00251	10.6549	6.17337	7.0093
Snrpf	Post-transcriptional regulation	RNA processing / splicing	69878	11.7201	6.62396	7.96299	108.219	61.9255	63.0176
Snrpe	Post-transcriptional regulation	RNA processing / splicing	20643	63.1784	71.7708	74.1459	301.943	141.887	130.848
Ubr1	Post-transcriptional regulation	Ubiquitin	22222	5.68556	7.09896	7.32846	6.32601	9.05061	9.17414
Klhl3	Post-transcriptional regulation	Ubiquitin	100503085	1.46738	2.73457	2.2058	0.173004	0.563008	0.747861
Ubr4	Post-transcriptional regulation	Ubiquitin	69116	13.5608	14.4621	14.1159	8.38805	19.7291	17.9688
Usp34	Post-transcriptional regulation	Ubiquitin	17847	8.0982	10.2201	10.7676	12.5239	24.1177	23.4663
Klhl28	Post-transcriptional regulation	Ubiquitin	66689	2.41575	3.31839	3.33799	1.41797	2.49865	2.66014
Ubr5	Post-transcriptional regulation	Ubiquitin	70790	17.1872	17.5252	18.7132	23.5342	39.2041	37.5288
Usp32	Post-transcriptional regulation	Ubiquitin	237898	7.68694	7.57327	8.72583	6.20814	9.82887	9.5243
Usp24	Post-transcriptional regulation	Ubiquitin	329908	15.7196	16.4584	18.3231	16.1681	26.5563	23.8927
Ubr3	Post-transcriptional regulation	Ubiquitin	68795	23.5498	24.5986	30.4244	12.6435	18.8011	19.5955
Usp38	Post-transcriptional regulation	Ubiquitin	74841	9.96725	11.2171	14.0213	8.7373	12.3154	12.6829
Ube2n	Post-transcriptional regulation	Ubiquitin	93765	6.23024	4.58662	5.77911	8.37516	5.78184	5.91125
Ube2t	Post-transcriptional regulation	Ubiquitin	67196	0.7252	0.976741	0.463063	21.5037	14.3796	12.6637
Usp2	Post-transcriptional regulation	Ubiquitin	53376	2.57003	0.703229	1.70308	15.4103	10.5552	8.60896
Ube2a	Post-transcriptional regulation	Ubiquitin	22209	24.4022	19.4202	21.1484	19.7562	12.2044	11.8797
Ube2e1	Post-transcriptional regulation	Ubiquitin	22194	37.0558	26.6325	32.3939	73.1945	42.0787	42.2374
Usp43	Post-transcriptional regulation	Ubiquitin	216835	0.0526554	0.0133305	0.00801977	0.928271	0.435897	0.560265
Ube2c	Post-transcriptional regulation	Ubiquitin	68612	3.22501	3.23176	0.809964	199.097	108.192	92.5313
G2e3	Post-transcriptional regulation	Ubiquitin	217558	1.58705	2.14846	2.26922	7.04313	10.4066	9.68219
Rnf169	Post-transcriptional regulation	Ubiquitin	108937	8.27288	7.53729	10.2745	5.29684	7.59225	8.29047
Rchy1	Post-transcriptional regulation	Ubiquitin	68098	20.4409	14.7552	17.3754	15.1981	10.0663	10.4058
Tat	Metabolism	AA Metabolism	234724	907.417	444.779	424.052	8.19351	21.868	58.6961
Aadat	Metabolism	AA Metabolism	23923	124.119	42.4278	130.782	1.35246	3.60963	4.62026
Cdo1	Metabolism	AA Metabolism	12583	970.899	857.238	1148.93	32.9968	46.6932	147.223

S3 Table. Continue...

Tdo2	Metabolism	AA Metabolism	56720	1235.42	763.142	1660.48	32.8213	53.4095	120.275
Aass	Metabolism	AA Metabolism	30956	108.433	101.357	118.028	12.1678	24.7667	20.8805
Pfas	Metabolism	AA Metabolism	237823	2.59423	2.86781	2.91844	15.3537	29.5456	25.6881
Kmo	Metabolism	AA Metabolism	98256	204.43	172.693	230.446	10.0596	18.1057	17.2544
Mat1a	Metabolism	AA Metabolism	11720	783.939	895.318	752.055	136.579	199.526	273.084
Hpd	Metabolism	AA Metabolism	15445	1411	1037.63	1463.68	151.519	212.209	250.634
Hal	Metabolism	AA Metabolism	15109	156.575	98.1736	111.925	17.0085	25.4609	25.0174
Bckdha	Metabolism	AA Metabolism	12039	156.391	157.345	185.96	40.3727	57.9775	60.3899
Atp7a	Metabolism	AA Metabolism	11977	1.54544	2.12767	2.44622	5.26355	8.06543	7.17267
Pycr1	Metabolism	AA Metabolism	209027	0.112023	0.0398445	0.0630344	1.97928	1.22969	1.23537
Dync2h1	Cell cycle	Cytoskeleton	110350	0.484288	0.636889	0.663329	0.808971	2.27786	2.17525
Tubb1	Cell cycle	Cytoskeleton	545486	0.128005	0.39681	0.291312	0.348011	1.04613	0.773343
Fry	Cell cycle	Cytoskeleton	320365	0.222743	0.317981	0.190701	0.480809	1.1027	1.24219
Myo5b	Cell cycle	Cytoskeleton	17919	6.02318	8.91516	7.26106	1.66926	3.22572	3.46425
Hook3	Cell cycle	Cytoskeleton	320191	10.6931	12.4695	12.0826	3.40359	6.48211	6.23057
Fign	Cell cycle	Cytoskeleton	60344	0.710961	0.884692	0.776386	1.34295	2.78754	1.97624
Mylk	Cell cycle	Cytoskeleton	107589	17.0926	13.7205	15.6869	2.52656	3.9463	4.50754
Kif21b	Cell cycle	Cytoskeleton	16565	0.285066	0.6342	0.426687	0.860376	1.31725	1.51671
Myh9	Cell cycle	Cytoskeleton	17886	32.4349	35.7807	33.2464	32.093	48.62	51.6254
Kif1b	Cell cycle	Cytoskeleton	16561	22.9156	28.0097	24.0625	14.2922	21.4989	21.7724
Kif4	Cell cycle	Cytoskeleton	16571	0.234799	0.545468	0.15834	7.48216	11.6421	10.6095
Kif21a	Cell cycle	Cytoskeleton	16564	13.0704	18.1875	12.1739	13.7651	19.8915	20.7381
Kif15	Cell cycle	Cytoskeleton	209737	0.129237	0.182538	0.126133	9.36834	14.4544	12.7831
Kif18a	Cell cycle	Cytoskeleton	228421	0.230591	0.683789	0.40409	6.35556	8.98033	8.68448
Tubb2a	Cell cycle	Cytoskeleton	22151	85.4791	101.492	43.401	30.3826	21.1793	22.2293
Dynll1	Cell cycle	Cytoskeleton	56455	9.53262	13.1156	9.59443	51.5283	35.9642	36.6893
Tuba1c	Cell cycle	Cytoskeleton	22146	20.8519	21.0131	17.878	28.8445	21.1253	19.4772
Kif3a	Cell cycle	Cytoskeleton	16568	1.2875	0.970097	0.846037	2.58064	1.51375	1.7693
Tuba1b	Cell cycle	Cytoskeleton	22143	6.35956	4.49191	3.72709	68.4525	45.1279	38.6402
Arhgap26	Cell cycle	Cytoskeleton	71302	4.30638	2.6711	5.41493	1.34419	0.63048	0.865912
Acta2	Cell cycle	Cytoskeleton	11475	1.03368	0.754496	0.590082	4.97397	2.29448	3.25509
Tubb2b	Cell cycle	Cytoskeleton	73710	0.0411882	0.083835	0.0421711	3.45933	1.05075	1.22738
Arhgap33	Cell cycle	Cytoskeleton	233071	0.271	0.0493644	0.0412007	0.604568	0.214841	0.169573
Pck1	Metabolism	Lipid metabolism	18534	918.92	719.164	835.034	11.1923	35.0723	101.482
Ppara	Metabolism	Lipid metabolism	19013	55.4984	73.7678	68.0827	6.31397	10.9255	11.0085
Xdh	Metabolism	Lipid metabolism	22436	42.4806	34.1214	41.1085	4.21336	5.99655	8.24714
Ucp2	Metabolism	Lipid metabolism	22228	4.96885	5.86362	3.60031	41.4419	17.5986	13.0017
Fmo3	Metabolism	Lipid metabolism	14262	62.9552	4.22061	0.0905709	1.00393	10.7866	24.1801
Apob	Metabolism	Lipid metabolism	238055	685.388	1073.89	1306.93	64.1165	273.856	301.918
Mfsd2a	Metabolism	Lipid metabolism	76574	13.6667	126.346	13.6242	1.0026	3.25121	3.59485
Pon1	Metabolism	Lipid metabolism	18979	769.487	570.234	948.382	9.8682	14.881	70.8428
Abca1	Metabolism	Lipid metabolism	11303	38.5915	45.2878	53.1533	6.37207	19.0831	21.8149

S3 Table. Continue...

Fgl1	Metabolism	Lipid metabolism	234199	384.289	616.401	309.871	6.99292	11.1144	43.1251
Serpina12	Metabolism	Lipid metabolism	68054	13.6209	10.5956	94.7699	0.298792	1.04264	0.816595
Rdh16	Metabolism	Lipid metabolism	19683	27.9745	128.305	24.4775	0.506841	1.38928	1.60437
Lrp2	Metabolism	Lipid metabolism	14725	0.122955	0.157165	0.192667	1.41535	4.72222	3.49622
Enpp2	Metabolism	Lipid metabolism	18606	33.6754	25.9356	43.196	0.909847	1.51359	3.96236
Bhmt	Metabolism	Lipid metabolism	12116	574.46	358.049	930.71	26.8695	46.8395	100.202
Kmt2c	Metabolism	Lipid metabolism	231051	3.30596	4.00989	4.68679	2.99514	7.7866	7.47866
Agmo	Metabolism	Lipid metabolism	319660	58.0646	50.5663	57.1873	2.30098	3.9068	5.92373
Klkb1	Metabolism	Lipid metabolism	16621	124.496	124.248	197.016	9.97186	20.1463	21.3513
Atp7b	Metabolism	Lipid metabolism	11979	8.77717	9.84069	11.8884	9.43978	17.7844	17.57
Abcc3	Metabolism	Lipid metabolism	76408	63.0418	34.1171	24.5779	6.20272	9.45258	14.0826
Apoc1	Metabolism	Lipid metabolism	11812	13246.8	21192.7	24573	665.342	1011.8	1508.36
Hsd3b7	Metabolism	Lipid metabolism	101502	447.867	385.188	589.193	22.598	36.7452	42.578
Plin5	Metabolism	Lipid metabolism	66968	30.9339	34.0374	24.3332	13.8109	23.1291	25.1004
Inpp4a	Metabolism	Lipid metabolism	269180	5.56429	3.02495	3.55337	1.22637	1.96743	2.24713
Apoa5	Metabolism	Lipid metabolism	66113	732.317	862.026	878.658	24.3025	38.4932	41.6285
Ephx2	Metabolism	Lipid metabolism	13850	552.221	459.377	697.339	29.6775	44.3242	53.264
Rock1	Metabolism	Lipid metabolism	19877	10.8019	12.9837	14.3388	9.66424	14.6649	16.5197
Abcb4	Metabolism	Lipid metabolism	18670	77.7872	118.663	99.22	15.6391	23.6301	25.4885
Ldlr	Metabolism	Lipid metabolism	16835	55.0703	83.2132	60.1769	14.4902	24.3832	19.8231
Rgn	Metabolism	Lipid metabolism	19733	1122.7	855.591	1507.95	83.2795	125.869	121.538
Abcd2	Metabolism	Lipid metabolism	26874	9.90599	22.7794	9.68618	7.59526	11.4756	10.5454
Icam1	Metabolism	Lipid metabolism	15894	7.0602	8.27788	4.76891	20.654	12.9089	15.1044
Sigmar1	Metabolism	Lipid metabolism	18391	142.451	111.553	66.4319	85.6257	63.2364	48.3989
Atf4	Metabolism	Lipid metabolism	11911	82.4753	79.8436	95.9158	110.548	64.4343	78.4641
Ifrd1	Metabolism	Lipid metabolism	15982	11.6508	13.1176	14.6186	36.0407	20.5324	23.8126
Dlk1	Metabolism	Lipid metabolism	13386	0.00669998	0.0022213	0.00530016	23.3465	12.471	5.56941
Serpine1	Metabolism	Lipid metabolism	18787	0.29163	0.404868	0.322971	5.39042	1.16235	2.63686
Cpe	Metabolism	Lipid metabolism	12876	0.699401	0.323205	0.456855	1.31341	0.205155	0.581969
Spta1	Cell cycle	Membrane cytoskeleton	20739	0.0148756	0.297295	0.08779	0.822048	3.72616	2.30455
Sptb	Cell cycle	Membrane cytoskeleton	20741	0.145886	0.204953	0.134918	0.581776	2.39471	1.40407
Syne1	Cell cycle	Membrane cytoskeleton	64009	6.23861	9.57686	7.31365	3.16799	7.98357	6.07093
Ank1	Cell cycle	Membrane cytoskeleton	11733	0.0103545	0.0432092	0.0230735	0.666656	1.69233	1.17891
Ank2	Cell cycle	Membrane cytoskeleton	109676	0.566139	0.272932	0.518449	7.43309	14.817	12.8292
Camsap2	Cell cycle	Membrane cytoskeleton	67886	4.78523	6.19843	6.36306	3.6853	6.16268	6.53247
Ankhd1	Cell cycle	Membrane cytoskeleton	108857	8.13654	7.09291	7.5707	9.64595	14.8516	15.2052
Tmem164	Cell cycle	Membrane proteins and trafficking	209497	3.57198	5.09646	3.34064	3.63854	5.08411	5.21873
Tmem43	Cell cycle	Membrane proteins and trafficking	74122	6.54315	8.12229	5.61944	11.7859	7.38855	8.53014
Tspan7	Cell cycle	Membrane proteins and trafficking	21912	16.171	18.4764	17.0314	34.9176	22.4105	24.6729
Tmem60	Cell cycle	Membrane proteins and trafficking	212090	17.9978	24.2556	22.8095	20.1469	12.9868	12.8196
Tspan3	Cell cycle	Membrane proteins and trafficking	56434	3.40651	3.28239	4.4617	24.9175	14.2098	17.2632
Tmem106a	Cell cycle	Membrane proteins and trafficking	217203	5.25512	4.40593	3.45923	3.84205	2.1923	2.0861

S3 Table. Continue...

Tmed2	Cell cycle	Membrane proteins and trafficking	56334	25.6032	21.7136	24.8537	26.7936	13.0234	13.6012
Rabgap1	Cell cycle	Membrane proteins and trafficking	227800	7.39046	9.23973	9.35624	8.65964	12.0792	12.2664
Rab4a	Cell cycle	Membrane proteins and trafficking	19341	22.365	16.6965	12.7164	20.1961	14.4569	12.0954
Rab23	Cell cycle	Membrane proteins and trafficking	19335	2.61523	3.59961	2.61488	3.28485	2.12972	1.99414
Nr3c1	Post-transcriptional regulation	Nuclear genes	14815	22.8601	27.3305	34.6809	11.0514	17.3751	16.8282
Ncoa1	Post-transcriptional regulation	Nuclear genes	17977	4.26535	4.00976	4.70976	2.73265	4.26015	3.97245
Nsd1	Post-transcriptional regulation	Nuclear genes	18193	9.94303	11.5861	13.3122	10.7479	25.9633	26.5309
Nfib	Post-transcriptional regulation	Nuclear genes	18028	12.4272	16.0515	18.6968	4.66113	9.98979	9.2737
Nrip1	Post-transcriptional regulation	Nuclear genes	268903	2.65492	4.98331	3.62108	2.91292	5.54355	5.03584
Nfia	Post-transcriptional regulation	Nuclear genes	18027	7.32361	5.93695	5.99585	2.44892	4.42809	4.43313
Ncor1	Post-transcriptional regulation	Nuclear genes	20185	16.8005	17.3884	18.4098	16.4098	24.8094	26.3445
Npat	Post-transcriptional regulation	Nuclear genes	244879	1.60865	2.20019	2.46592	4.28234	5.9672	6.2658
Numa1	Post-transcriptional regulation	Nuclear genes	101706	12.8414	12.5641	11.9361	17.9426	26.2188	24.4087
Nol12	Post-transcriptional regulation	Nuclear genes	97961	7.32312	4.32491	5.69704	15.0925	10.2579	10.1664
Npm3	Post-transcriptional regulation	Nuclear genes	18150	15.7956	17.2843	19.6421	118.507	82.0371	77.729
Npm3-ps1	Post-transcriptional regulation	Nuclear genes	108176	1.41698	0.83182	0.800284	11.0436	3.44123	2.94896
Snora74a	Post-transcriptional regulation	Nucleolar RNA	436583	0.231313	6608.41	3962.86	8.65221	3492.84	3480.06
Snora21	Post-transcriptional regulation	Nucleolar RNA	100302498	2.32567	975.128	589.745	9.56235	400.677	444.028
Snora44	Post-transcriptional regulation	Nucleolar RNA	100217418	9.20044	2352.35	1152.58	40.9403	1489.83	1926.8
Snora43	Post-transcriptional regulation	Nucleolar RNA	100306955	14.4881	143.213	79.2769	84.1719	1498.72	1596.82
Snora52	Post-transcriptional regulation	Nucleolar RNA	100217419	13.6934	222.851	105.349	30.8398	324.126	323.703
Snhg12	Post-transcriptional regulation	Nucleolar RNA	100039864	17.5197	14.1715	14.392	104.448	58.1682	68.0002
Psme4	Post-transcriptional regulation	Proteasome	103554	40.5564	46.1495	48.1725	18.4895	27.4824	31.0166
Psmg3	Post-transcriptional regulation	Proteasome	66506	23.2296	16.6606	20.3151	71.0961	51.2784	46.6889
Pomp	Post-transcriptional regulation	Proteasome	66537	231.703	199.982	259.31	161.713	113.092	108.936
Psmg2	Post-transcriptional regulation	Proteasome	107047	26.2209	16.967	21.8501	76.652	51.6958	43.0479
Ppp1r3g	Other	Protein phoshpatase	76487	0.120086	0.736107	0.042482	0.190198	0.475438	0.417482
Ppp1r12b	Other	Protein phoshpatase	329251	0.964694	1.38642	1.50694	2.0149	4.84776	4.15484
Ptprd	Other	Protein phoshpatase	19266	23.3905	25.0756	29.2647	3.16159	7.41497	5.92364
Ppm1l	Other	Protein phoshpatase	242083	2.98104	3.01908	3.17969	0.717929	1.29956	1.43387
Ptprf	Other	Protein phoshpatase	19268	43.5253	39.1824	50.5567	11.5597	16.7835	21.9224
Ptprg	Other	Protein phoshpatase	19270	5.37859	6.57338	8.28391	1.28555	1.93551	1.87666
Ppp2r3a	Other	Protein phoshpatase	235542	4.45812	5.54064	5.93772	7.06987	10.8378	9.57002
Ppp6r3	Other	Protein phoshpatase	52036	23.5296	28.9736	30.3056	23.8189	32.0718	32.3823
Ppp3r1	Other	Protein phoshpatase	19058	26.8194	17.8766	20.9367	42.3074	31.7689	31.0355
Ppp1r15a	Other	Protein phoshpatase	17872	6.46216	4.52831	4.75554	12.8799	6.9565	7.43524
Slc1a2	Metabolism	Solute carrier	20511	22.388	18.9111	29.5022	0.480527	1.06563	4.9327
Slc5a3	Metabolism	Solute carrier	53881	1.86786	2.6682	2.32584	1.58017	3.8214	3.07621
Slc4a4	Metabolism	Solute carrier	54403	12.8875	18.3948	13.3346	4.96336	8.69437	7.45739
Slc25a13	Metabolism	Solute carrier	50799	101.309	125.832	131.687	51.0919	74.8598	75.7748
Slc51a	Metabolism	Solute carrier	106407	0	0	0	0.824266	0.25918	0.0511009
Slc22a26	Metabolism	Solute carrier	236149	21.1204	14.1752	0.294786	0.0511078	0.461817	2.57619

S3 Table. Continue...

Slco1b2	Metabolism	Solute carrier	28253	558.104	662.559	1216.93	18.5822	39.7264	67.3312
Slc26a1	Metabolism	Solute carrier	231583	83.8795	66.987	84.3791	1.68817	3.27976	6.25413
Slc15a2	Metabolism	Solute carrier	57738	2.78214	1.3985	1.27556	0.893151	1.91681	2.88082
Slco1a4	Metabolism	Solute carrier	28250	30.1951	35.0361	12.92	3.69012	6.33966	13.8645
Slc25a21	Metabolism	Solute carrier	217593	5.32406	6.06099	7.1461	0.292023	0.6757	0.728538
Slc27a5	Metabolism	Solute carrier	26459	534.751	473.594	523.208	16.5125	32.4422	44.5173
Slc22a23	Metabolism	Solute carrier	73102	20.769	16.9646	19.6002	2.66499	3.95666	4.75945
Slc35e2	Metabolism	Solute carrier	320541	15.925	19.4554	20.7303	4.12592	6.7351	6.686
Slc7a6os	Metabolism	Solute carrier	66432	18.1118	13.2915	14.4784	31.7873	23.6702	23.4721
Slc7a5	Metabolism	Solute carrier	20539	1.14366	1.44802	1.22587	43.6018	31.0722	28.8977
Slc1a5	Metabolism	Solute carrier	20514	0.870181	1.43957	0.456374	35.5774	25.7034	23.0816
Slc35f2	Metabolism	Solute carrier	72022	0.197683	2.72149	0.294815	20.8535	14.7781	13.4949
Slc38a7	Metabolism	Solute carrier	234595	9.57863	5.34981	7.33101	7.29399	4.89841	4.81132
Slc50a1	Metabolism	Solute carrier	19729	10.5232	5.70227	9.36499	13.531	8.19907	8.50575
Slc39a10	Metabolism	Solute carrier	227059	1.22028	2.20414	1.65279	17.9994	10.7304	11.1006
Slc29a2	Metabolism	Solute carrier	13340	0.484702	0.23608	0.272912	13.6565	8.14463	6.69376
Slc22a6	Metabolism	Solute carrier	18399	0.00380818	0.00251714	0.0027013	1.54162	0.968934	0.315653
Slc4a11	Metabolism	Solute carrier	269356	0.0943242	0.058007	0.0418528	1.41324	0.131919	0.545071
Zbtb16	Other	Zinc Finger	235320	3.73205	2.41143	2.04398	2.51853	3.6781	5.16341
Zhx3	Other	Zinc Finger	320799	10.4295	15.4254	18.1434	4.0261	13.0754	11.4535
Zfp369	Other	Zinc Finger	170936	3.57052	3.43329	4.06234	3.39876	10.3102	7.68182
Zbed6	Other	Zinc Finger	667118	9.15339	11.9306	11.246	6.48371	15.7851	16.6799
Zkscan1	Other	Zinc Finger	74570	9.58165	9.3341	11.5147	5.84649	13.323	13.488
Zfp618	Other	Zinc Finger	72701	0.153065	0.307766	0.325645	2.33347	5.16748	5.06352
Zfp108	Other	Zinc Finger	54678	0.934182	1.23269	1.58327	0.307647	0.58615	0.668783
Zfp551	Other	Zinc Finger	619331	0.67232	0.689908	0.919304	0.478811	0.936431	0.994984
Zfp292	Other	Zinc Finger	30046	4.07073	3.79299	4.21445	3.49229	6.72136	7.24873
Zfp26	Other	Zinc Finger	22688	1.698	2.19256	2.48874	1.85492	3.69197	3.71243
Zfhx4	Other	Zinc Finger	80892	2.39541	1.62454	1.80176	1.31968	2.71576	2.48695
Zzef1	Other	Zinc Finger	195018	6.8182	7.46194	8.02898	3.53074	6.72965	6.88602
Zfp619	Other	Zinc Finger	70227	0.581412	0.623371	0.746912	0.336657	0.611003	0.623937
Zfp169	Other	Zinc Finger	67911	1.34992	1.10734	1.07847	0.473462	0.821101	0.900578
Zfp780b	Other	Zinc Finger	338354	1.91097	2.24988	2.82228	1.1342	1.9059	2.13158
Zfp953	Other	Zinc Finger	629016	1.40454	2.03545	2.34986	0.72637	1.25021	1.33265
Zfp39	Other	Zinc Finger	22698	1.95503	2.52954	3.03031	1.76361	2.94936	3.19314
Zbtb37	Other	Zinc Finger	240869	1.55514	1.65299	2.17926	1.18837	2.0474	2.07127
Zfp334	Other	Zinc Finger	228876	0.62485	0.826697	0.582907	0.74523	1.29287	1.27266
Gm14326	Other	Zinc finger	665211	0.986226	1.2863	1.75927	1.23287	2.06041	2.06915
Zfp324	Other	Zinc Finger	243834	3.62481	4.16872	5.2717	1.76547	2.96919	2.87773
Zfp799	Other	Zinc Finger	240064	2.28886	2.74283	3.20941	1.55586	2.50132	2.5654
Zfp715	Other	Zinc Finger	69930	5.92893	5.90386	7.25379	1.99671	2.95115	3.50667
Zfp646	Other	Zinc Finger	233905	3.70063	4.29763	4.76777	4.89541	7.53277	8.14947

S3 Table. Continue...

Zfp58	Other	Zinc Finger	238693	3.15274	3.54202	4.65036	1.7359	2.76492	2.78345
Znrf3	Other	Zinc Finger	407821	1.60743	2.17191	2.17728	3.56976	6.26741	5.17588
Zc3hav1	Other	Zinc Finger	78781	13.2114	17.0524	18.8102	10.4713	16.3457	15.9623
Zfp606	Other	Zinc Finger	67370	3.03149	3.6713	4.33876	2.52028	4.02461	3.72995
Zfp568	Other	Zinc Finger	243905	4.4166	5.31497	5.27749	18.9018	28.853	28.7499
Zbtb1	Other	Zinc Finger	268564	3.44025	4.52199	5.32474	5.36007	7.90402	8.42707
Zfp654	Other	Zinc Finger	72020	4.57083	5.74159	6.07213	2.76315	4.12221	4.28328
Zfp318	Other	Zinc Finger	57908	6.82285	5.0101	5.99139	4.60649	7.17501	6.57321
Zfp866	Other	Zinc Finger	330788	3.93358	3.80608	4.19822	2.65595	3.74774	4.1546
Zfp84	Other	Zinc Finger	74352	2.47481	2.48736	2.69853	2.91401	4.33926	4.31512
Zfyve9	Other	Zinc Finger	230597	6.88888	7.47805	9.05843	4.27203	6.4726	6.19726
Zmym2	Other	Zinc Finger	76007	6.2163	7.92237	9.70765	5.69603	7.98772	8.89036
Zfp281	Other	Zinc Finger	226442	7.15736	8.55073	10.6808	7.76819	10.5629	11.4309
Zhx1	Other	Zinc Finger	22770	13.5915	16.9848	20.8073	10.0151	13.6771	14.0308
Zfp236	Other	Zinc Finger	329002	4.01295	3.68927	3.93947	3.84015	5.41513	5.19065
Zranb2	Other	Zinc Finger	53861	32.2845	23.1104	27.9003	40.0962	28.3591	28.388
Zmat2	Other	Zinc Finger	66492	19.0522	12.6233	14.863	26.5442	17.3253	19.3316
Zcrb1	Other	Zinc Finger	67197	32.428	27.0054	29.616	47.0153	28.6789	31.4613
Zfp706	Other	Zinc Finger	68036	31.8971	21.8451	25.9959	66.4699	41.0808	36.9531
Zfp750	Other	Zinc Finger	319530	4.94476	3.28164	2.50034	8.07995	3.50723	3.14015
Clec4a1	Other	Zinc finger	269799	0.996633	1.31291	0.642256	3.09577	0.917376	1.28293
Zfp365	Other	Zinc Finger	216049	0.00937858	0.00715714	0.00916461	0.404439	0.0741774	0.0780681
Uox	Metabolism		22262	1070.46	1267.11	1274.63	54.6146	93.2579	97.5349
Upp2	Metabolism		76654	100.459	40.9934	130.963	4.73936	8.21194	7.09587
Sdhc	Metabolism		66052	243.909	173.223	212.579	162.778	121.525	109.914
Sdhaf1	Metabolism		68332	22.7831	21.4137	24.1448	29.5602	20.8427	19.3031
Rapgef6	Other		192786	4.35621	5.70603	5.81832	5.23475	9.32437	9.0466
Ccdc171	Other		320226	1.50285	2.28787	1.78622	0.575022	1.35175	1.28509
Cc2d2a	Other		231214	1.03731	1.23121	1.23923	2.32362	4.52169	3.96003
Ccdc85c	Other		668158	3.6667	4.27667	4.76217	8.88416	15.4776	14.2252
Rb1cc1	Other		12421	10.648	13.7877	16.1497	6.93463	10.4367	11.6028
Ccdc59	Other		52713	13.5935	10.7994	12.5445	27.4622	18.9821	18.3089
Ccdc85b	Other		240514	1.97026	1.0766	1.47741	2.7428	1.70383	1.85963
Scoc	Other		56367	4.96724	4.24193	4.33131	10.8284	6.93508	5.18403
Ddi2	Other		68817	50.0034	50.9838	65.5771	24.8882	39.1912	38.6282
Dram1	Other		71712	0.205976	0.536116	0.422425	7.73488	3.55194	2.83429
Ddit4	Other		74747	7.7561	5.82262	2.66198	33.6938	12.2518	14.8311
Ddit4l	Other		73284	0.516688	0.601877	0.262987	1.26545	0.259131	0.246328
Foxo3	Other		56484	6.41155	8.38284	10.8169	7.96183	13.3276	13.382
Foxm1	Other		14235	0.340573	0.367083	0.0843666	11.4935	8.07492	7.44101
Foxq1	Other		15220	10.7563	7.81597	8.97662	5.75851	3.22229	3.21818
Pde4dip	Other		83679	23.0299	20.2067	24.8104	10.9972	15.2353	18.125

S3 Table. Continue...

Pde6d	Other		18582	6.95469	4.95198	4.90282	9.90362	4.82198	5.47202
Polq	Other		77782	0.0524592	0.114713	0.0366272	2.96999	4.53646	4.34526
Pole3	Other		59001	20.6826	21.5545	22.3329	75.1711	51.7336	54.0409
Polr2m	Other		28015	50.1276	40.8673	48.5449	76.0422	53.8134	51.4542
Polr1d	Other		20018	44.9397	54.8608	54.8887	121.391	80.7555	86.1873
Polr3gl	Other		69870	6.90119	6.68552	6.42261	13.6122	8.78056	8.36406
Rasal2	Other		226525	2.09138	2.77371	3.28393	0.87259	1.42106	1.46719
Ralgapa1	Other		56784	2.32867	2.96024	2.77809	6.46528	10.4579	10.0575
Rassf1	Other		56289	5.70304	5.12594	4.43362	13.7288	8.75862	9.04603
Bclaf1	Survival / apoptosis		72567	12.8385	14.6285	17.1241	32.2664	49.7927	51.9506
Bcl7b	Survival / apoptosis		12054	10.5652	8.44297	8.09776	10.4076	7.14942	6.97347
Pdrg1	Survival / apoptosis		68559	25.1704	14.9982	22.3329	26.1262	15.884	17.1504
Lrp1	Survival / apoptosis		16971	69.2632	73.3359	88.6388	6.72563	25.17	26.7833
Irs1	Survival / apoptosis		16367	6.08433	4.24331	6.53753	1.4163	3.1355	3.08476
Apc	Survival / apoptosis		11789	4.68073	6.62072	5.87197	5.08265	8.18136	8.05819
Brca1	Survival / apoptosis		12189	0.401963	0.779712	0.477109	5.82544	9.33882	9.11337
Pik3r1	Survival / apoptosis		18708	20.606	16.9512	26.5642	7.7748	12.0835	12.0515
Gnai1	Survival / apoptosis		14677	0.283872	0.480046	0.655933	1.30017	0.56328	0.559279
Adcy9	Survival / apoptosis		11515	5.94761	7.02724	9.90179	1.22106	2.86736	3.27425
Cdk12	Survival / apoptosis		69131	5.19607	6.70909	6.30042	8.44749	17.2465	17.8049
Arhgef12	Survival / apoptosis		69632	21.9387	28.2851	28.501	10.2111	18.9665	18.7715
Gna12	Survival / apoptosis		14673	32.7889	27.4259	45.8873	5.66096	9.13298	11.7508
Lrp6	Survival / apoptosis		16974	13.9159	16.7706	16.623	11.2476	19.7544	21.0091
Pik3c2b	Survival / apoptosis		240752	1.02647	1.50228	1.31911	1.45044	2.87086	2.15244
Cdk13	Survival / apoptosis		69562	6.80246	8.84372	9.3391	7.17823	12.01	12.4288
Plcb1	Survival / apoptosis		18795	3.47389	3.7337	5.1767	1.63509	2.66401	2.60669
E2f2	Survival / apoptosis		242705	1.6987	1.35112	1.19872	1.97597	3.24249	3.05802
Cflar	Survival / apoptosis		12633	22.2717	22.2699	23.4281	2.9768	4.18506	5.17663
Hipk2	Survival / apoptosis		15258	4.63481	5.57458	5.97756	2.42433	3.62392	3.71188
Braf	Survival / apoptosis		109880	3.56504	3.62136	3.6392	3.45707	4.97734	5.33493
Rasa1	Survival / apoptosis		218397	8.49197	11.0144	11.7556	7.48218	10.0824	10.1358
Gnai2	Survival / apoptosis		14678	39.727	34.9834	29.0965	31.9147	20.9115	23.3877
Rhob	Survival / apoptosis		11852	13.8959	11.7681	11.8433	30.6528	20.0341	22.5001
E2f6	Survival / apoptosis		50496	11.5874	8.26812	9.10638	9.4346	6.29214	6.40608
Arhgef5	Survival / apoptosis		54324	7.79765	9.8226	6.19065	3.95614	2.52274	2.50411
E2f7	Survival / apoptosis		52679	0.184891	0.0817424	0.0855549	1.80355	1.21673	1.00386
Bmp8b	Survival / apoptosis		12164	0.0716599	0.833316	0.00754486	17.6589	12.1236	8.49944
Rras	Survival / apoptosis		20130	22.6202	16.5427	10.125	8.1816	3.87211	4.78705
Mdm2	Survival / apoptosis		17246	14.0571	13.2556	13.4132	32.1851	14.8116	18.4603
Bax	Survival / apoptosis		12028	28.6932	24.8083	19.2522	84.1316	51.2334	57.2665
Bbc3	Survival / apoptosis		170770	6.03129	7.931	4.95231	22.1724	10.6899	11.2818
Abl2	Survival / apoptosis		11352	1.69857	1.8986	1.90218	8.70624	12.2303	12.4148

S3 Table. Continue...

Raver1-fdx1l			100568458	0.106611	1.65748	1.83041	0.0403268	8.21207	6.41781
Vaultrc5			378472	0.0534982	72.5792	57.344	0.763596	87.7635	60.4724
Tbrg3			21378	0.00591693	0.634788	0.430887	0.0262155	2.33554	2.13863
E030024N20Rik			595139	0.00117898	0.28936	0.191446	0.0125722	0.910212	0.759776
Gm20199			100504377	0.00801781	0.590652	0.499269	0.0394545	2.1996	1.80636
Adam4			11498	0.19501	0.374724	0.248096	0.132693	1.20031	0.886146
Ces3b			13909	400.337	143.059	458.274	0.3901	0.841753	6.2286
BC089597			216454	89.0549	136.562	70.7969	0.765134	1.59405	11.9721
Gm4787			214321	0.170526	0.378237	0.261827	0.139615	0.836457	0.726896
Fam46c			74645	3.25292	4.85181	4.52143	1.05453	6.08228	5.01201
Xlr3a			22445	9.75156	5.29394	5.91362	0.553895	1.93596	3.72295
C430002E04Rik			78706	0.644953	0.708285	1.01059	0.900786	4.69158	3.78949
1810053B23Rik			69857	0.140031	3.39117	0.386422	0.405099	1.14678	2.92234
Sacs			50720	0.108073	0.504789	0.303017	0.538671	3.45799	1.6854
Mug1			17836	1338.69	516.882	1567.6	11.4061	20.1197	118.1
D830031N03Rik			442834	0.406342	1.18724	1.94548	0.998363	4.34605	3.96667
Pkhd1l1			192190	0.0828855	0.111146	0.0966517	0.105317	0.498928	0.358197
Serpina3k			20714	12335.8	8241.87	17560	26.918	41.7692	275.707
Syt14			329324	0.00949467	0.0475232	0.00977258	1.08513	4.38423	3.9156
E330033B04Rik			319722	0.356441	0.503787	0.415475	0.211637	0.801633	0.767917
Serpina3m			20717	813.188	812.956	1144.28	5.35259	9.82265	38.2647
Ces1d			104158	495.715	281.535	340.526	4.85833	9.38404	32.7063
Ugt3a2			223337	293.047	221.83	354.366	2.5445	4.13703	20.1633
Hsd17b6			27400	109.881	99.757	93.7412	3.55987	6.15064	25.6587
BC039771			408057	0.287915	0.442878	0.608354	0.235817	0.902179	0.731591
Snx29			74478	2.74257	2.67374	8.30083	0.581179	1.96989	1.92772
Dpys			64705	184.873	122.639	190.974	2.87625	4.64797	18.7934
Akap5			238276	0.26998	0.280402	0.239991	0.195863	0.656628	0.6166
Mug2			17837	143.971	107.591	109.37	1.22494	2.32492	6.40504
Onecut2			225631	11.9544	8.50224	10.1391	0.28314	0.585244	1.30961
Cd177			68891	0.00309775	0.0300163	0.018881	0.376011	1.7642	0.760086
Gm5176			382421	0.280279	0.483135	0.388871	1.01817	3.04527	3.08451
E230029C05Rik			319711	0.04964	0.310986	0.202766	0.206961	0.563068	0.681695
D130040H23Rik			211135	0.0543989	0.312553	0.236807	0.128788	0.361372	0.404434
Itih1			16424	377.375	264.31	370.426	4.87163	7.41242	25.3855
F5			14067	120.481	77.5117	109.532	3.12283	5.93603	12.9297
Apol11b			328563	0.0202906	0.359078	0.108834	0.311166	0.7025	1.04559
F830016B08Rik			240328	11.393	7.99165	8.39645	0.281909	0.538915	1.11208
Mboat2			67216	0.0318761	0.161576	0.107073	0.161599	0.476013	0.38841
Atxn1			20238	3.88373	4.4168	7.22289	0.915905	2.39686	2.46736
Sgsm1			52850	2.31129	4.22134	1.84523	0.540525	1.28757	1.58475
Epb4.2			13828	0.00207333	0.117742	0.0159349	0.711225	2.25305	1.54563

S3 Table. Continue...

Peg3			18616	2.34563	6.85888	0.909158	8.05344	24.0695	18.3401
Deptor			97998	29.1129	29.7838	44.7511	8.50385	21.429	22.5541
Alas2			11656	22.4585	28.3489	29.4968	8.22457	22.4202	20.1095
Lifr			16880	247.275	57.6541	380.362	3.48644	7.12363	11.1885
Dst			13518	6.92885	11.9974	7.8425	3.26105	9.38383	7.4226
Akap13			75547	7.35246	9.13532	9.5254	3.62639	9.04975	9.12799
Casc5			76464	0.091723	0.387529	0.120888	5.96719	15.7254	14.0389
Abhd2			54608	37.1085	43.9811	38.1704	7.17528	15.5957	20.3865
Grem2			23893	6.20716	1.79901	7.1808	0.190218	0.44082	0.495196
March3			320253	0.76142	0.951126	1.02947	0.230404	0.57987	0.550574
Enpp3			209558	22.3557	17.9652	24.9354	0.967577	2.24012	2.50832
Fbxo40			207215	0.130642	0.140226	0.151789	0.396305	0.910066	1.00339
Inmt			21743	783.09	506.069	694.661	8.4292	22.1893	18.2462
Hsd3b2			15493	79.2007	12.9316	85.4496	0.476224	0.910059	1.39726
Exph5			320051	5.26088	4.22264	6.35937	0.531245	1.44069	1.09134
Itpr2			16439	8.02017	10.416	9.09977	2.64664	6.50975	5.97167
Pcsk5			18552	3.08389	3.78142	2.14775	0.731749	1.66279	1.74316
BC005561			100042165	3.17947	3.68075	4.4392	1.93906	4.28551	4.74561
Fryl			72313	2.93125	3.5322	3.54327	2.0119	5.1138	4.23323
Emc1			230866	6.80911	8.68389	9.19971	5.88111	14.0851	12.9857
Celsr1			12614	2.08628	3.07057	1.54844	1.5182	3.33981	3.64945
Fat1			14107	4.78118	5.3981	6.29144	2.7733	5.99358	6.68021
Gm4890			234479	0.300952	0.452795	0.412233	0.264561	0.63794	0.563808
Tnrc6b			213988	3.71256	3.62076	3.96586	2.754	6.57487	5.92564
Kcnj2			16518	0.48256	0.268717	0.483635	0.24163	0.470962	0.635157
Tenm4			23966	0.0121083	0.0648039	0.041103	0.443481	0.8517	1.16609
Scml4			268297	0.0289726	0.105168	0.0442516	0.251698	0.618458	0.508298
Trpm4			68667	0.304976	0.78662	0.494356	0.19146	0.386254	0.467209
Aff2			14266	0.0119958	0.0279427	0.0489917	0.492491	1.11144	1.07161
5031425E22Rik			269630	4.54894	6.44262	5.67742	1.99267	4.33555	4.48154
Itih4			16427	909.043	792.528	750.592	33.7482	53.6116	103.085
Atp11c			320940	60.3497	67.5464	112.378	7.10124	13.6311	17.8333
Adamts12			239337	0.0899789	0.176165	0.192322	0.805469	1.96953	1.57016
Gbp11			634650	32.3857	11.7653	50.419	0.624336	1.29612	1.40613
Psd3			234353	8.11597	10.1776	10.454	0.668422	1.2681	1.64344
A630089N07Rik			320586	0.206407	0.307368	0.38374	1.16297	2.62277	2.38327
Gm15800			269700	3.46348	2.88262	3.51879	2.55801	5.64134	5.32665
Brwd3			382236	2.24513	2.66152	3.09559	2.22587	5.1428	4.38448
Ctse			13034	3.15679	8.38394	2.99955	4.33567	10.2141	8.15337
Enpep			13809	33.3269	43.2421	37.6591	1.89549	3.26198	4.8765
Tnxb			81877	1.11943	1.50765	1.92092	0.226976	0.497164	0.458583
AU041133			216177	1.77576	2.62399	3.21866	0.855371	1.85806	1.7336

Adamts6			108154	0.192384	0.770536	0.263519	0.56407	1.2149	1.13769
Pard3b			72823	1.15086	1.12937	1.95618	0.945952	1.63887	2.36024
Ldlrad4			52662	2.07121	2.88951	3.08269	0.978739	2.20649	1.87048
Bod1l			665775	3.21532	4.08319	4.28962	4.70854	9.29219	10.1887
Mical3			194401	4.36263	3.87248	4.15881	2.43	5.20464	4.79667
Aspm			12316	0.177051	0.393661	0.14209	4.28495	9.39394	8.22074
Med13			327987	6.00383	7.66723	8.19735	5.73634	11.6566	11.8547
Trim10			19824	0.0297617	0.2696	0.0687077	1.27995	3.02168	2.27091
Rhd			19746	0	0.00815581	0	2.05359	5.06521	3.41044
Dpyd			99586	151.437	137.517	134.971	8.98921	16.8575	19.5264
Cpeb4			67579	15.8377	13.6158	17.1425	4.42811	7.92002	9.98402
Serpina3n			20716	249.57	181.079	178.878	12.0676	19.5136	29.1361
Prr14l			215476	4.49665	5.74786	6.15953	4.68675	9.57915	8.93904
Car1			12346	8.66601	1.2784	7.85318	4.59922	10.6704	7.69061
Itpr1			16438	9.57108	14.4001	12.3267	5.31396	11.2224	9.75606
Thada			240174	1.85439	2.38727	2.5762	4.23237	9.08839	7.57941
Gypa			14934	0.0188054	0.737027	0.203936	4.28496	10.072	6.97613
Vwf			22371	1.17743	1.61164	1.49189	0.527336	1.17484	0.905066
Rfx3			19726	0.575665	0.765123	0.717489	0.927531	1.82833	1.79749
Spata13			219140	5.96939	8.64326	7.56105	4.13082	7.82379	8.31767
Nufip2			68564	7.40127	9.60935	10.9465	8.79534	17.2087	16.8931
Fn3krp			238024	6.59411	7.10792	9.98511	3.80934	7.80083	6.98024
Lrrc8a			241296	8.12292	10.087	11.285	4.82226	9.08179	9.54998
Pcdh17			219228	0.116629	0.299493	0.343989	0.346964	0.689719	0.646623
A830082N09Rik			414093	0.148919	0.127384	0.130719	0.210805	0.416639	0.394308
2410089E03Rik			73692	0.77455	1.08743	0.94619	1.24693	2.29836	2.47842
Pgap1			241062	3.67263	4.42271	5.35847	1.92529	3.25874	4.154
Acvr2b			11481	1.35363	1.00595	1.4939	6.02465	14.5125	9.12803
Cdon			57810	0.390935	0.378194	0.430876	0.886776	1.99851	1.42148
Ces1e			13897	141.093	50.1407	141.811	1.52806	2.71179	3.10781
Mier3			218613	4.7846	6.00843	6.96252	2.62863	4.62334	5.37607
Atp8a2			50769	0.0231039	0.103881	0.0838487	5.29803	11.9372	8.37736
Cd24a			12484	0.96481	2.7253	1.57995	9.91255	23.0132	15.1498
Wdfy3			72145	6.09286	7.82595	7.88566	7.24952	14.709	12.6618
Sntb1			20649	21.4585	34.1731	27.9937	4.57405	7.40087	9.89105
Brip1			237911	0.423245	0.74463	0.628504	2.27804	3.98345	4.55302
Exoc3l4			74190	0.381805	0.775675	0.459837	3.94356	6.50873	8.32952
Hykk			235386	19.5076	21.2476	22.3187	1.69378	2.59459	3.8426
Mcf2l			17207	0.0773624	0.0964585	0.0902393	0.597991	1.15583	1.07066
Spg11			214585	3.56081	4.7325	4.76353	3.34031	6.40517	6.01091
Rarres1			109222	101.99	146.861	133.306	1.82526	3.14283	3.64825
Chpf2			100910	10.4262	11.5304	12.2614	3.22182	5.55324	6.40719

Nbas			71169	7.76248	10.6391	10.0139	3.92309	7.53957	6.98226
Adap2			216991	25.616	19.5612	24.6406	3.09495	5.27892	6.13882
Tet2			214133	3.89473	2.34536	2.08472	2.30557	4.70684	3.8197
Epg5			100502841	5.49639	6.94904	6.82717	3.44144	6.65419	6.00491
Bche			12038	29.7599	35.7461	32.272	5.65131	10.9373	9.76851
Tulp4			68842	2.84341	3.80245	4.33715	0.816847	1.36547	1.63442
Ppap2b			67916	51.1729	49.0961	69.2606	5.93648	8.961	12.8292
N4bp2			333789	6.22811	4.81937	7.27206	4.34072	6.60776	9.29615
Pdpr			319518	7.29566	7.65081	10.1712	5.85741	11.2956	9.89829
Lpp			210126	5.98973	6.95339	7.04324	3.48719	6.60422	5.99463
Trim2			80890	8.60121	14.9964	7.71597	5.37821	9.92632	9.45572
Tln1			21894	17.3126	20.0703	21.1401	13.3754	24.3833	23.8035
Hmbox1			219150	4.39449	4.64417	4.96159	3.29811	5.8214	6.03567
Tacc1			320165	11.9153	11.2058	16.0053	3.30094	5.06977	6.88752
Plxna2			18845	4.55834	5.78913	4.04405	2.55018	4.57738	4.5355
Ino80d			227195	2.75161	2.33146	2.34536	3.47924	6.41804	6.00612
Wdr19			213081	0.847495	0.914188	0.98433	0.687492	1.22261	1.22238
Ash1l			192195	6.04248	6.46127	6.44799	6.2258	11.6651	10.4057
Strbp			20744	3.91721	5.26119	4.71308	3.3654	6.0274	5.87422
Megf9			230316	6.41046	9.26968	7.37504	6.64742	11.3323	12.1862
H2-Q10			15007	1253.22	2027.8	1348.58	62.0802	111.694	107.394
Abhd15			67477	16.8022	15.8595	19.8358	3.194	5.15574	6.08894
Nipbl			71175	8.36648	8.64384	9.79334	8.05166	13.567	14.7041
Lama2			16773	0.426249	0.630396	0.910292	0.754794	1.20363	1.45597
Fbxl18			231863	2.37819	1.80281	2.23809	2.71215	4.92776	4.56718
4930422G04Rik			71643	0.144501	0.263645	0.209185	1.22534	2.21101	2.06271
Sult1a1			20887	195.508	234.25	228.58	25.3841	36.4434	53.2101
Brwd1			93871	8.91218	11.0286	13.8009	8.24183	14.1173	14.4026
Frat1			14296	3.05162	3.71523	3.57931	4.65427	7.93755	8.14015
Nrp1			18186	21.6734	27.8233	46.8635	2.82759	4.54602	5.21689
Sbno1			243272	8.84707	10.2048	10.9556	10.4418	18.4608	16.9596
Golgb1			224139	12.0207	10.5776	11.0725	5.05768	8.11856	9.03309
Huwe1			59026	20.2978	17.0469	17.8424	23.6491	42.1725	37.9514
Pan3			72587	5.50841	6.24226	6.87132	7.61715	12.7608	13.0047
2810474O19Rik			67246	4.45959	5.82838	10.8126	3.52998	5.56172	6.32358
Smg1			233789	5.60741	7.42554	6.60059	14.6863	26.5231	22.8034
6430548M08Rik			234797	2.94953	2.53485	2.4222	1.03895	1.73805	1.73684
Fhod3			225288	0.33354	0.161329	0.353703	3.34847	6.01122	5.17953
Il1rap			16180	91.1674	77.4248	142.775	6.40733	9.71223	11.6777
Magi1			14924	5.90957	6.89187	6.7864	3.16031	5.01481	5.49655
Fam179b			328108	4.35345	5.58188	6.36864	6.81177	11.0198	11.6199
Tdrd7			100121	14.2306	17.9509	17.5605	8.5845	13.1945	15.3173

S3 Table. Continue...

Tnrc6c			217351	3.16702	2.9216	3.02597	2.77097	4.56313	4.61212
D3Ertd254e			241944	1.5155	2.25592	2.07943	1.26927	2.04126	2.15953
Optn			71648	45.6876	44.9796	45.9264	4.70831	7.77	7.79652
Ttc37			218343	6.78928	7.85376	8.449	9.3822	14.9077	16.1117
Cnot1			234594	19.0262	20.6207	22.8598	30.1588	50.9597	48.4725
Pcmtd1			319263	30.5317	38.9719	41.4862	6.02277	9.47086	10.3946
Pink1			68943	95.8341	117.082	107.841	15.1879	23.3051	26.7908
Rsad2			58185	2.67796	4.20365	3.73574	3.43842	5.58821	5.70551
Atp9a			11981	28.5152	43.0191	35.6194	3.85256	5.6001	7.13764
Ftx			78878	1.27802	0.990193	1.09851	1.79667	3.01097	2.85749
Ttc3			22129	4.45584	7.38043	5.33017	8.12893	12.9374	13.5629
Cldn1			12737	60.3152	60.1576	66.8431	5.36304	9.01631	8.45897
Blvrb			233016	115.842	170.435	138.253	18.4073	27.423	32.7489
Qrich1			69232	19.3479	24.029	28.1731	22.9648	36.8426	37.8887
Scai			320271	1.10156	0.728389	1.19932	0.502453	0.775171	0.854898
Dyrk2			69181	14.2468	14.2239	16.4856	9.0327	14.7429	14.3059
Med13l			76199	2.36429	2.87176	2.85706	2.03635	3.24661	3.28862
Garem			381126	9.79894	7.70801	7.38651	3.11555	5.02657	4.95365
Adrbk2			320129	1.92188	3.25746	2.32758	1.92738	2.93535	3.23754
Agap1			347722	1.42416	1.71028	1.63663	2.75275	4.0929	4.73394
Lrrc3			237387	42.842	24.2301	34.816	3.14973	4.6005	5.50909
Pcnt			18541	1.85717	2.26762	2.23356	5.19987	8.74002	7.86248
Sash1			70097	9.66216	12.6264	11.0207	3.82348	5.97025	6.22211
Diap3			56419	0.16316	0.30805	0.0880608	3.21334	5.13824	5.10203
Prrc2c			226562	7.71633	5.89526	6.2635	25.4301	41.7629	39.3031
Crocc			230872	0.443669	0.439088	0.327715	0.931507	1.50464	1.46332
Ly75			17076	0.876063	1.9426	1.05401	6.44725	11.9228	8.82527
Alkbh8			67667	2.98231	3.48434	4.21392	7.25452	11.6303	11.4341
Gcn1l1			231659	10.5962	7.72132	9.82997	20.2977	35.5036	29.2124
Scarb2			12492	60.2558	77.0054	91.2868	23.7263	37.7057	37.3273
Baz2b			407823	3.01599	2.72479	2.95475	2.05986	2.96931	3.55764
Irf2bp2			270110	12.1182	26.9716	15.5344	14.7743	21.9348	24.7629
Marc1			66112	234.963	249.137	262.713	11.0723	15.7935	19.314
Aff4			93736	14.2394	15.7165	18.0962	10.8565	16.9125	17.1618
Narf			67608	20.1614	16.3986	21.1187	4.13757	6.33148	6.65778
Socs4			67296	6.21835	7.18012	9.76557	7.19946	11.2511	11.2169
Bptf			207165	3.94789	4.00775	4.23617	7.61776	11.7305	12.042
Tnfaip2			21928	19.1278	17.0853	13.2031	5.3171	8.12534	8.44365
Masp1			17174	40.4641	26.3686	38.4433	6.50421	10.7253	9.56314
Secisbp2l			70354	14.9209	29.4015	16.3673	6.0397	9.58843	9.2091
Mbd5			109241	1.27937	1.38605	1.33759	0.935259	1.40856	1.50217
Dido1			23856	14.0324	11.6519	12.8053	10.7066	16.5754	16.6784

S3 Table. Continue...

Fancm			104806	1.50103	1.72981	1.70145	2.9062	4.66964	4.35578
Itih2			16425	518.126	417.096	533.387	45.6391	61.477	81.5576
Ago2			239528	2.98892	3.7076	2.86281	12.6961	22.5864	17.1196
Cnot6l			231464	11.5616	11.1919	12.2106	10.9292	17.4417	16.4249
Ppfibp2			19024	12.5493	10.9235	8.45219	3.9451	5.56975	6.69069
A530054K11Rik			212281	2.13588	2.67565	3.56349	1.38209	2.18989	2.0859
C4b			12268	361.179	334.48	279.63	28.4817	42.1815	45.967
Sall4			99377	0.0637566	0.126481	0.0256109	3.46676	5.50257	5.20703
Dennd4a			102442	6.51899	8.09113	8.68023	9.2181	14.8405	13.5307
Gm17296			212728	2.15204	1.79145	2.64928	11.8068	20.0106	16.3933
Ahctf1			226747	14.7211	17.3275	13.7258	29.5555	47.4689	43.2797
Arid5b			71371	8.81758	5.15768	8.73036	3.93101	5.61967	6.42594
Alcam			11658	20.1658	24.5725	28.0019	7.12629	10.85	10.8669
Sec16a			227648	27.5778	22.3032	27.1489	11.1851	16.7579	17.3281
AI314180			230249	24.6668	28.5417	28.6596	29.8865	48.3589	42.8618
Heatr5a			320487	4.28752	4.8951	5.04396	4.99516	7.48389	7.7223
Gpcpd1			74182	23.5845	15.2549	41.3728	4.9876	7.49259	7.68002
Atad2b			320817	5.00777	4.22736	5.57765	5.25299	7.94329	8.0166
Setd2			235626	7.76493	8.46144	9.0926	11.311	16.9339	17.391
Ggact			223267	93.22	87.7292	167.417	25.8927	40.0563	38.476
Arid1b			239985	3.61831	3.47555	3.83341	2.81818	4.321	4.21115
Tecpr2			104859	2.97734	3.0449	2.98483	1.62863	2.34887	2.58697
Fan1			330554	2.95856	2.57366	3.18081	2.1743	3.32726	3.24688
Atad5			237877	0.396877	0.558005	0.496336	4.2383	6.59609	6.21295
Asxl2			75302	4.7773	5.45098	5.57645	6.44868	10.4885	9.01908
Jmjd1c			108829	7.9793	9.68994	9.33982	7.32599	10.5792	11.5211
2310035C23Rik			227446	6.35274	7.86331	8.07693	5.03239	7.69417	7.46806
Atg2b			76559	6.47211	6.87277	7.25822	8.38352	13.1642	12.0994
Dnajc6			72685	0.0224968	0.0363671	0.0430545	8.16265	11.6699	12.9157
Plch1			269437	0.104861	0.118678	0.149391	2.39112	3.34569	3.82851
Mysm1			320713	3.67811	4.23132	4.62045	3.3335	4.79137	5.19235
Wdr33			74320	14.9264	17.7649	20.291	18.1104	27.4063	26.7249
Thoc2			331401	6.19129	6.9686	7.43627	8.38058	11.7577	13.3045
Eea1			216238	9.37984	11.2527	12.8998	9.07034	13.7642	13.3093
Lmtk2			231876	4.50975	5.5069	5.50204	4.99969	7.46308	7.4567
Uggt1			320011	22.5299	18.9175	22.861	8.15584	11.0687	13.3703
Ints1			68510	5.94432	5.69008	5.73122	14.5186	23.2684	20.1519
Kidins220			77480	12.4977	15.6983	13.2753	6.87261	9.86041	10.6514
Stxbp5			78808	3.06916	3.49294	3.11821	2.04206	2.87847	3.21599
Rsbn1			229675	2.19573	2.7548	3.05295	5.02472	7.64322	7.31208
Agtpbp1			67269	4.24864	4.46534	5.21602	9.26228	14.2179	13.3519
Nudt12			67993	32.6078	38.0671	44.1979	6.92309	9.9706	10.6234

S3 Table. Continue...

Lrig2			269473	2.28227	2.78412	2.68844	1.44379	2.08367	2.21037
Tpmt			22017	34.6567	36.9634	48.4938	4.55797	6.72074	6.82342
Rspry1			67610	9.49475	11.8162	13.3776	4.88283	7.06629	7.43876
Gm608			207806	8.33845	5.85874	5.72582	3.06641	4.74484	4.3615
Pgm2l1			70974	0.80772	1.06676	1.0139	2.03458	2.97946	3.04605
Sfxn5			94282	12.4916	14.0969	13.5653	2.43599	3.54874	3.66279
Ncapg2			76044	0.345655	0.793291	0.403896	8.1185	13.2134	10.9119
Mbnl2			105559	13.0407	12.6229	12.5255	9.22823	12.9251	14.3966
Rsf1			233532	2.22332	2.86455	2.47685	2.80804	4.11338	4.17319
Pvrl3			58998	27.9243	27.5081	35.99	7.38967	10.8341	10.909
Phf20l1			239510	8.64566	8.70797	10.7752	3.65647	5.19905	5.53393
Dmd			13405	2.80937	3.37981	3.70237	2.54313	3.77165	3.68617
Ltn1			78913	7.79637	8.71048	10.2149	10.6727	15.143	16.1464
Man1a			17155	72.6379	86.4045	99.4739	20.9063	28.9285	32.4174
Trip12			14897	21.3236	24.0485	24.1911	25.1256	36.9956	36.4292
Tnrc18			231861	6.38112	3.28708	4.0887	1.81867	2.59501	2.72018
Atp2b1			67972	9.12549	8.6775	8.92638	5.84141	7.75807	9.35096
Hivep1			110521	3.26533	2.29039	2.33861	1.83781	2.78069	2.57231
Tshz1			110796	4.36282	4.00744	5.16274	4.64557	6.92568	6.58462
Asb7			117589	3.67586	4.32038	4.69168	3.63835	5.43177	5.13572
Glg1			20340	9.80818	13.2317	11.139	11.5438	16.4779	17.0087
Depdc5			277854	3.32113	4.10707	3.61025	4.67361	6.55939	6.99498
Rap1gap2			380711	0.109232	0.438827	0.201662	4.32757	6.01786	6.50892
Erap1			80898	35.1558	40.4358	47.0157	12.8424	18.112	19.0318
Utp20			70683	4.17241	4.54015	5.31901	15.2442	22.6585	21.3845
Evi5			14020	38.6155	46.7061	53.0468	22.7345	31.5863	33.8789
Man1a2			17156	8.69609	13.0284	10.2895	16.3071	24.9306	22.0354
Otud4			73945	10.6842	11.536	12.0705	25.2578	37.2045	35.3253
Smc2			14211	1.04653	2.28178	1.23141	17.1045	24.7477	24.3159
Arfgef2			99371	13.1863	15.7295	11.5679	8.50079	12.4937	11.891
Edem3			66967	16.8088	16.092	19.8443	8.44533	11.492	12.7319
2900097C17Rik			347740	10.1124	12.8054	13.1045	10.0214	14.4577	14.2443
Ythdf2			213541	9.22972	10.7594	12.1024	14.7017	20.7608	21.3076
Pcf11			74737	5.77907	7.46588	8.25398	7.97715	11.1035	11.7076
Larp4b			217980	23.0859	21.2491	29.0583	23.2532	33.1352	33.3355
Ppip5k2			227399	17.4986	21.2401	21.2947	24.0732	34.9689	33.6905
Ccser2			72972	10.4872	11.8954	11.2192	4.70311	6.82219	6.58817
Arel1			68497	13.1923	15.0491	16.8984	4.71641	6.51625	6.90387
C230081A13Rik			244895	2.22866	3.05035	2.98921	2.07117	2.88641	2.99222
Marf1			223989	20.3019	12.5659	14.019	8.24422	12.372	11.0349
Wwp1			107568	20.7977	26.0424	29.3235	10.2585	14.7304	14.3408
Ibtk			108837	21.2965	29.0202	25.6786	24.563	36.3629	33.2719

S3 Table. Continue...

Gapvd1			66691	12.0929	11.9853	14.0247	15.9613	23.2913	21.9081
Smcr7l			239555	11.374	9.14271	12.0236	8.1097	11.1649	11.7948
Ubn2			320538	3.93871	2.37243	2.61775	3.66038	5.37663	4.97725
Mreg			381269	27.7386	30.6334	35.2776	14.0937	19.5561	20.228
Heatr1			217995	5.43441	5.62944	7.29156	19.5753	28.5042	26.6719
Snx30			209131	2.22585	3.05789	2.81913	4.57313	6.54955	6.32042
Hipk1			15257	15.1136	14.693	15.4139	10.876	14.9292	15.6819
Scaf11			72193	12.3417	12.0168	13.8139	26.9977	38.2773	37.4104
Tmtc3			237500	2.55922	3.55076	3.70052	4.95219	6.81838	7.04184
Nrde2			217827	4.47419	4.55465	5.61501	4.40892	6.12568	6.12813
Ipo8			320727	20.5584	21.8224	27.5007	11.1481	15.0168	15.6123
Phf17			269424	10.7534	13.9092	20.0848	12.9136	17.6923	17.3832
Tsc22d2			72033	3.89395	4.25101	4.27609	4.01826	5.46565	5.42101
Smarca5			93762	9.28659	11.9053	13.2205	21.7298	29.3436	29.4521
Topors			106021	10.5455	13.478	14.5384	13.7227	18.4501	18.6655
Car12			76459	0.0126428	0.00304627	0.0143034	29.129	21.831	21.7482
Wdr45b			66840	23.2697	15.0673	26.1111	24.9214	18.4743	18.5404
Dut			110074	4.8614	5.35645	5.72821	59.7015	44.5084	43.6079
Atl2			56298	48.3162	47.4667	39.4258	37.8384	27.3374	28.2729
Rpl35			66489	114.569	193.835	169.179	514.087	378.311	374.178
Med4			67381	11.7373	8.28427	9.82674	34.5221	25.0501	25.267
Lamtor1			66508	46.9607	46.186	46.0289	40.2903	28.8451	29.7783
Impdh1			23917	1.91431	1.92212	1.39769	50.0073	35.5434	37.0377
Get4			67604	10.7204	10.3592	10.1407	24.1183	17.8822	17.0183
Hspbap1			66667	1.0824	1.40461	0.729373	11.2517	8.11773	8.10241
Mrpl47			74600	23.6275	23.4891	25.7476	68.4189	51.0086	47.4851
Mmachc			67096	12.4753	9.47477	11.2486	28.3814	21.1066	19.7331
Exosc1			66583	8.14115	5.43716	7.11417	27.644	19.7235	19.9989
Pla2g12a			66350	14.361	7.55019	14.4299	66.8313	48.9713	47.0076
Naca			17938	162.868	220.452	215.381	417.772	296.267	303.367
Spon2			100689	9.11554	24.0896	8.68416	26.3165	18.7601	18.8972
Dusp16			70686	14.4648	8.56137	11.9282	12.6374	8.85889	9.20296
Eif3h			68135	91.7911	94.3787	91.4457	177.248	125.151	128.102
Amn			93835	0.0553253	0.136104	0.0776305	36.0667	26.6879	24.8529
Rplp2			67186	519.846	597.173	606.364	2107.56	1518.56	1488.4
Cox19			68033	51.8453	51.2467	54.5928	53.2041	38.4872	37.3748
0610009D07Rik			66055	33.4144	43.6569	45.6425	52.0682	37.2877	36.8758
Lsm12			268490	16.7524	11.6725	12.2436	26.8826	18.833	19.4617
Mpdu1			24070	50.0245	38.3246	50.4811	42.1327	31.1091	28.8167
Fam195b			192173	19.202	13.5209	16.5891	20.8301	14.9202	14.6024
Ist1			71955	30.2234	23.7485	25.1479	30.1964	21.0006	21.7855
Ninj1			18081	148.274	103.536	125.392	64.6239	47.4594	44.0749

S3 Table. Continue...

Pno1			66249	32.623	26.9434	36.5132	46.4892	32.8958	32.828
Hsbp1			68196	67.6756	62.0941	71.2228	46.4741	32.0221	33.6813
Bloc1s6			18457	7.65751	5.94452	7.46588	8.95317	6.5785	6.05555
Sec11a			56529	73.3492	57.9472	81.2272	97.654	71.3617	65.8239
Dad1			13135	146.205	87.5991	112.309	113.795	78.0217	81.7074
Arpc4			68089	33.4635	28.8871	30.586	49.4177	35.9427	33.429
Srsf9			108014	30.838	23.9886	26.9036	53.6427	37.267	37.9199
Leprotl1			68192	11.855	9.64317	10.03	10.5363	7.5753	7.17154
Hprt			15452	66.7832	67.152	75.7474	150.739	110.199	99.5413
Pkig			18769	17.1546	17.78	17.1275	24.2804	16.4113	17.3381
Cnih4			98417	5.23567	5.60858	5.66411	10.8449	7.57716	7.35451
Arpc2			76709	99.229	75.5824	79.7712	107.973	72.6903	75.9587
Clhc1			69885	0.17147	0.172612	0.0556251	23.0326	15.5803	16.1045
Ormdl3			66612	73.9408	54.9466	59.4139	16.3743	11.1155	11.3503
Ociad1			68095	98.9754	77.3802	92.3256	98.0323	68.8155	65.5495
Vat1			26949	14.4147	11.2724	8.91762	20.5014	14.2708	13.7043
Gmpr2			105446	12.491	7.49609	9.44955	17.994	12.5074	11.929
Cd68			12514	4.29218	5.91146	4.63399	32.4974	22.3389	21.7191
Tm2d1			94043	16.6664	24.3645	23.2534	16.0751	10.6083	11.1762
Dyrk3			226419	0.825039	2.18478	0.761394	13.4223	9.16151	9.00204
Lsm5			66373	8.21506	17.051	12.9664	46.4764	31.4944	31.3376
Marcksl1			17357	7.18146	7.85323	6.86209	37.2046	26.9057	23.4962
Pmf1			67037	14.8709	17.7387	12.7686	59.5035	42.9258	37.6591
Cmtm8			70031	45.0053	33.3783	42.3155	54.087	39.8149	33.4612
Tma16			66282	3.81172	3.05477	3.40218	25.917	18.0176	16.9574
Gm11974			100041286	5.11886	5.64797	4.55299	53.4799	35.3992	36.7061
Gtf2e2			68153	12.1616	9.37414	10.9263	36.7499	25.9847	23.5514
Bsn			12217	0.00141513	0.00248424	0.00226326	1.91183	1.33831	1.22336
Laptm5			16792	5.09217	5.79948	4.25412	21.997	15.4527	14.0179
Denr			68184	19.4031	13.9871	17.4588	44.6757	28.6648	31.0444
Ssbp4			76900	2.57888	2.98438	2.3749	32.3633	22.5735	20.6311
Spc24			67629	8.73636	7.97201	8.07316	104.203	77.178	62.1009
Pgrmc2			70804	31.4506	39.6092	21.8436	22.6037	15.2062	14.7787
Scpep1			74617	38.3149	39.6761	24.0607	46.7367	32.8252	29.1265
Glrx5			73046	111.725	103.909	106.572	216.036	152.923	133.18
2700029M09Rik			72612	8.75752	6.71449	8.49937	35.6152	23.6013	23.2699
Crtap			56693	5.09489	4.2564	3.52157	22.7001	16.2498	13.7102
Cyb5r1			72017	6.70201	4.66759	5.05995	23.1466	16.5455	13.9909
Aqp11			66333	30.3614	27.5732	25.2564	43.3829	31.7767	25.5444
Rps27l			67941	465.435	1166.56	761.179	665.428	440.282	432.827
2010012O05Rik			66439	5.08538	4.14584	3.13361	5.87264	3.9333	3.75598
Bloc1s2			73689	10.6419	10.8842	11.6448	21.6606	14.9613	13.339

S3 Table. Continue...

Aaas			223921	6.26801	4.96001	3.85873	34.5017	23.7339	21.2255
Prps1			19139	21.4126	19.7447	18.3947	73.4076	49.5581	45.9367
Pgap3			320655	1.27871	0.749728	0.911037	4.12891	2.74788	2.60337
Gltp			56356	2.42057	2.58124	1.98144	5.94691	3.79629	3.87645
Stard3nl			76205	10.0542	13.7801	15.7262	14.5478	9.11804	9.56397
Reep5			13476	8.30148	7.60085	5.19542	5.48084	3.43181	3.58627
Mrpl12			56282	134.256	96.3492	105.514	324.602	225.877	190.631
Loxl2			94352	1.24531	0.855285	0.898939	2.27916	1.38028	1.53271
Ndufc1			66377	215.106	237.342	223.251	191.623	129.428	114.931
1110004E09Rik			68001	16.905	12.2807	15.1422	34.0627	21.9281	21.3426
Fam162a			70186	114.157	105.014	102.204	176.206	113.714	108.673
3110082I17Rik			73212	4.36738	2.61912	7.95313	26.9294	17.3672	16.5813
Stbd1			52331	53.5669	30.5473	49.4971	33.0813	23.869	18.2023
Fbln5			23876	1.52593	1.25198	1.84586	1.89963	1.18451	1.20757
Utp23			78581	2.53017	2.05008	2.32536	4.44966	2.75316	2.84941
Nt5c3b			68106	3.36802	3.8696	2.80224	12.0179	7.4874	7.62522
Egln3			112407	9.17866	4.86529	7.69651	14.1154	8.59009	9.12277
2510049J12Rik			70291	6.59201	12.5569	5.50219	5.6538	3.41291	3.65699
Htatip2			53415	35.7095	38.5993	15.5458	26.9829	18.1861	15.5795
Tulp3			22158	0.540599	0.424238	0.468069	6.73598	4.46596	3.93598
Dnph1			381101	9.64607	6.27244	10.2254	33.476	23.6602	18.0454
Sdr39u1			654795	7.59297	6.6755	6.40892	12.3045	8.18733	7.00035
Eif4a2			13682	117.94	147.732	133.561	86.1642	48.9236	57.1176
Atp5g3			228033	960.315	674.992	710.717	1004.83	661.568	572.579
Synpo			104027	1.76448	1.76738	0.949644	1.83793	1.03564	1.219
Tnip1			57783	5.33385	4.13141	3.26906	6.94692	4.15654	4.33362
Atg9b			213948	0.101983	0.0975928	0.0548812	7.16277	4.94364	3.83656
Msn			17698	6.88445	9.36274	8.24915	19.913	10.366	14.0858
Them4			75778	19.628	12.5011	21.1914	15.7053	9.78112	9.27144
Ost4			67695	124.12	126.655	114.043	121.672	73.2622	74.1679
Mmp12			17381	0.058291	0.362402	0.158552	3.04655	1.95268	1.74347
Rpl22l1			68028	112.919	190.617	203.303	455.467	286.812	263.824
Atp6v0e			11974	51.8251	54.5509	53.1135	79.6037	48.0588	47.9532
Atp5j			11957	359.587	250.285	302.979	272.604	164.773	162.841
Gpr56			14766	1.40943	1.40163	0.942608	19.8038	11.4091	12.3948
Uqcrq			22272	712.75	669.76	652.722	435.069	260.354	261.665
Kctd17			72844	2.40949	1.73093	1.07635	18.5146	11.3454	10.8419
Arl6ip5			65106	34.7008	28.7056	31.7844	48.2705	29.876	27.6978
Plgrkt			67759	8.46624	6.55579	6.05566	19.9201	12.5309	11.2054
Gm561			228715	47.2817	77.0044	63.1639	60.0033	39.2311	32.4483
Ptp4a3			19245	2.88356	2.65405	1.60731	17.5648	10.8637	10.0379
Gpnmb			93695	0.0327583	0.0734773	0.0495319	5.01807	3.15193	2.79184

Triap1			69076	26.6754	16.0255	24.1696	27.6064	15.5841	17.0035
Csnk1e			27373	2.92998	2.44361	2.0971	8.54969	4.97569	5.09661
Golga7			57437	32.1797	23.1604	29.1659	23.4973	13.8761	13.6273
Pgd			110208	24.236	35.9128	10.7495	63.7528	42.2959	32.802
Enc1			13803	1.17532	2.70465	1.09604	2.47805	1.24191	1.68691
Mthfd2			17768	2.40679	0.948113	2.02212	42.2679	23.4958	25.6728
Camta1			100072	1.98961	1.73383	1.28083	1.94919	1.14319	1.1178
Tmsb10			19240	6.31121	5.00254	6.98509	123.609	57.4078	88.6497
Tpt1			22070	676.054	685.575	599.712	880.012	483.642	533.123
Defb1			13214	1.12263	15.9747	1.99821	19.8875	12.1334	10.8093
BC030867			217216	0.0687582	0.0416828	0.0496313	5.54745	3.59498	2.82222
Cox17			12856	121.842	111.368	123.039	228.655	139.123	123.624
Cnnm1			83674	0.065019	0.0275856	0.0272863	2.15658	1.40041	1.0895
Taf11			68776	14.9195	9.11425	11.3139	22.8989	13.4958	12.5813
Inpp5a			212111	8.83874	7.59268	6.77082	7.46668	3.78657	4.7369
Lsm2			27756	20.243	12.9882	14.5506	92.8286	57.2411	48.3466
Mir703			735265	1641.81	1625.95	1721.6	3062.93	1721.44	1735.64
Fblim1			74202	0.59016	0.417605	0.379759	2.22045	1.18254	1.32225
Sox18			20672	4.4184	2.746	2.93192	3.13145	1.70124	1.8049
Gyltl1b			228366	0.420235	0.937091	0.155432	14.5129	8.74861	7.52822
Espn			56226	0.582329	0.854826	0.240903	10.461	5.31781	6.43448
Dancr			70036	7.95275	2.72747	2.9324	24.9265	14.298	13.5162
Tceal5			331532	0.0295692	0.162607	0.0493906	7.64908	4.00584	4.52783
Cln6			76524	1.91116	2.06817	1.07421	14.3904	8.83003	7.25826
Them6			223626	2.90352	1.94281	2.01156	30.674	21.9755	13.1871
Apobec3			80287	0.828634	1.54011	0.704587	2.73455	1.57643	1.45547
Oasl2			23962	1.02554	1.33368	0.888044	2.0302	1.01402	1.24315
Glis1			230587	0.0115643	0.00723162	0.0104424	4.48276	2.7273	2.23694
2210404O07Rik			72273	0.485102	0.566523	0.441396	8.04984	4.64435	4.17935
Fau			14109	261.104	268.223	251.396	589.731	322.686	322.619
Psrc1			56742	0.143185	0.215538	0.0948048	27.6243	14.7909	15.4203
Adprh			11544	6.8803	3.90951	5.83657	26.0559	15.2842	13.1935
Gpsm3			106512	2.18335	1.40225	1.15343	2.68152	1.43483	1.48205
Mpzl1			68481	2.76725	2.55467	1.44465	16.8224	9.76925	8.47332
Mllt11			56772	0.696189	0.550767	0.458424	2.16062	1.20823	1.11542
Smco4			170748	13.1679	12.4539	9.32752	20.943	12.1734	10.3347
Nkain1			67149	0.0415014	0.0300507	0.0678236	5.89706	3.05023	3.21621
Vkorc1l1			69568	4.79873	4.77258	3.59095	5.4001	2.8397	2.85835
Smyd1			12180	1.69516	1.01948	2.02509	1.54399	0.892677	0.73844
Gpc3			14734	0.989699	1.48982	0.72051	4.17724	1.72891	2.72882
E130012A19Rik			103551	1.67394	1.48673	0.480192	17.7317	11.4695	7.40384
Troap			78733	0.21911	0.0955799	0.0178296	6.35131	3.46778	3.09902

2610001J05Rik			66520	11.2918	8.52642	8.7967	11.6995	6.10505	5.9331
Fgfbp3			72514	0.461118	0.162296	0.299976	1.48165	0.746425	0.777554
Laptm4b			114128	16.6975	15.5244	13.6264	19.1613	10.3546	9.26993
Jam3			83964	0.637119	1.42135	1.33872	1.27678	0.640585	0.66092
Rec8			56739	0.670036	1.04883	0.269055	2.746	1.58402	1.21942
Erh			13877	4.23838	3.5737	3.57001	25.0044	13.5717	11.6825
Mtfr2			71804	0.117973	0.0575007	0.0197419	3.0631	1.6189	1.46581
1500012F01Rik			68949	16.3657	23.5668	20.8758	153.637	66.1022	89.2685
Pkia			18767	0.408775	0.882593	0.8334	1.34302	0.657172	0.675993
Fmnl1			57778	0.402326	0.539951	0.335063	2.18406	0.971278	1.17821
Prss16			54373	0.0149121	0.00907694	0	1.78189	0.839748	0.89561
Mrps6			121022	23.2626	16.8826	15.8066	42.335	22.8966	18.4856
Bhlhe41			79362	0.333384	0.0385739	0.513524	0.52079	0.290741	0.2203
Krt20			66809	0.00420075	0.250477	0	4.4195	1.69204	2.71479
Niacr1			80885	0.0980577	0.278084	0.0739106	0.934898	0.453261	0.445286
Apbb1ip			54519	0.640723	0.872081	0.484785	3.2694	1.72449	1.37123
Cox7c			12867	370.704	319.206	292.531	337.166	167.393	150.083
Maff			17133	1.14372	1.56887	1.28158	12.7886	5.84652	6.05003
Lpcat4			99010	0.313441	0.197958	0.188126	48.6198	27.1632	18.7103
1810011O10Rik			69068	22.0176	37.1659	25.043	9.44364	3.16106	5.70369
Cbr3			109857	0.346621	0.624439	0.669198	2.38319	0.974421	1.16546
Krt10			16661	1.15347	0.707573	0.882622	0.742166	0.321098	0.340069
Evc2			68525	0.677647	0.902145	0.410027	5.1561	1.97567	2.63664
Thy1			21838	0.647781	0.785208	0.632927	1.42592	0.55413	0.708601
Tnfrsf10b			21933	0.267551	0.355397	0.0826662	3.42072	1.40126	1.58314
Nudt18			213484	3.76318	2.80184	1.41429	1.54395	0.598452	0.749191
Gng7			14708	0.33914	0.105191	0.0846317	0.692496	0.316205	0.273011
Fam212b			109050	0.0401957	0.049129	0.0276444	0.60379	0.20401	0.306892
Gm13710			672763	0.215418	0.114085	0.0142267	1.42692	0.594171	0.585657
Parvb			170736	1.25544	1.13784	0.731077	3.41362	1.84396	1.06764
Nat14			269854	0.455384	0.246048	0.128205	0.42554	0.161041	0.185505
Evc			59056	0.846837	1.17054	0.699856	2.20787	0.722805	1.09225
Oas2			246728	1.20673	0.822712	0.489114	1.83337	0.681021	0.793722
Cpne8			66871	1.38064	1.21337	1.33927	4.41734	1.87711	1.66013
Rps10			67097	206.889	177.668	161.342	849.516	358.403	314.343
Sh3bgr			50795	0.063205	0.0116589	0.04773	0.709372	0.252862	0.305204
Lypd8			70163	0	0	0.0245454	20.1524	8.61212	7.21816
Tnfrsf22			79202	0.135281	0.309925	0.144007	0.434106	0.174893	0.160131
Ly6a			110454	7.27226	9.25965	7.64615	13.805	4.81474	5.81757
Eda2r			245527	0.180152	0.712876	0.176811	3.12589	0.750262	1.76359
Cd63			12512	5.87684	8.35264	2.42435	73.904	20.5846	35.8809
Entpd2			12496	2.47389	1.96859	1.00203	0.828171	0.250706	0.360036

S3 Table. Continue...

Guca1b			107477	0.267716	0.0925246	0.0511018	0.371604	0.131337	0.137759
5730559C18Rik			67313	0.0644562	0.0276651	0.00318157	3.57627	0.886445	1.88674
Hrk			12123	0.0191692	0.0154857	0.0162596	2.44879	1.05133	0.72011
Prr22			100504446	0.340897	0.0678815	0.0871543	3.14748	1.3479	0.903366
Mro			71263	0.00729949	0	0	0.570724	0.249531	0.153441
Tnnt2			21956	0.089584	0.0654906	0.12933	0.400696	0.101214	0.179992
Efnb3			13643	0.104523	0.0488449	0.0207029	0.54526	0.176037	0.179057
Prr7			432763	0.0788756	0.0956301	0.0981199	3.10391	0.760839	1.31861
Pvrl4			71740	0.056126	0.102641	0.0940341	0.666449	0.181269	0.253791
Mmp13			17386	0.0557447	0.0932703	0.090225	0.936217	0.270758	0.32972
Pmaip1			58801	0.177138	0.518458	0.280695	7.2692	1.51143	3.42691
Dusp8			18218	0.433721	0.23624	0.074566	2.30176	0.608458	0.839635
Phlda3			27280	0.63696	0.725229	0.529193	8.51929	2.36283	2.88062
Adm2			223780	0	0	0	3.32907	0.658563	1.54424
Faxc			76132	0.00962209	0.00735168	0.00537381	0.386562	0.0991049	0.131222
Fbxl16			214931	0.0627076	0.10704	0.0407572	1.82706	0.725536	0.386129
Stc2			20856	0.0763757	0.0306249	0.0406343	1.84456	0.31133	0.907876
Ddr1			12305	0.639619	0.519839	0.327354	1.79082	0.445295	0.565052
Muc13			17063	0.0108578	0.0324276	0.0165587	1.40383	0.621902	0.232033
Atp8b5			320571	0.695578	0.782195	0.500133	0.413664	0.109745	0.0917984
Gpx3			14778	2.18909	1.46259	0.856921	32.9825	8.02837	7.78558
Mall			228576	0.0354578	0.0054522	0.00632915	0.434368	0.130129	0.0830586
Itm2a			16431	1.02432	0.793962	1.04883	4.26474	1.31864	0.789904
Cxcl1			14825	8.82203	4.75383	9.17879	16.1184	4.01207	3.62893
Saa3			20210	9.18784	9.25302	15.9853	88.5445	23.4565	15.1295
Gm3776			100042295	0.436167	0.105972	0	0.614546	0.163205	0.0882143
1700007K13Rik			69327	0.0997073	0.144168	0.0296807	0.983201	0.111876	0.258812
Sncg			20618	0.127632	0.110221	0.166873	1.44515	0.0925927	0.552402
Sprr1a			20753	0.0553657	0.0495458	0.0151932	1.7732	0.067833	0.63969
Cxcl2			20310	0.171558	0.0258883	0.109573	5.66193	0.429121	0.238521
