# Supplementary material for: Inhibition of hepatocellular carcinoma by metabolic normalization
Source: PLoS One. 2019 Jun 26;14(6):e0218186. doi: 10.1371/journal.pone.0218186 (PMC6594671; doi:10.1371/journal.pone.0218186)
Supplement: S4 Table — (PDF) [file pone.0218186.s015.pdf]

S4 Table. XLS spread sheet of the 682 transcripts from Fig. 3D that were deregulated in NFD tumors and significantly normalized by both HFDs.

Name	NCBI gene ID	Database object name	NT vs NL			MT vs NT			LT vs NT			Mean					
			Fold Change	P-Value	FDR P-Value	Fold Change	P-Value	FDR P-Value	Fold Change	P-Value	FDR P-Value	NL	ML	LL	NT	MT	LT
Hist2h2aa1	11778	Histone H2A	-69.677	0.00146	0.00725	934.447	2.3E-07	1.5E-05	813.365	4E-07	2.4E-05	6.4	15	13.6	0	125	106
Gabbr2	242425	Gamma-aminobutyric acid type B receptor subunit 2	-160.42	0	0	4.65989	0.00132	0.02432	7.19127	3.5E-05	0.0011	596	1609	701.8	6.25	25.2	37.8
Lrit1	239037	Leucine-rich repeat, immunoglobulin-like domain and transmemb	-325.92	0	0	4.68582	0.00102	0.01979	10.8968	2.2E-07	1.4E-05	596	350	422	3	12.4	27.8
Cyp2u1	71519	Cytochrome P450 2U1	-78.44	0	0	2.61347	0.00081	0.0164	3.80182	3E-06	0.00014	1995	1442	5989	44.25	97.8	131.4
Serpina12	68054	Serine (Or cysteine) peptidase inhibitor, clade A (Alpha-1 antiprote	-41.214	0	0	3.16508	0.0002	0.00525	2.52274	0.00288	0.03985	1317	746.4	6232	56.25	148.8	116.2
Gm11437	628813	Uncharacterized protein C17orf78 homolog	-58.526	0	0	4.02136	0.00125	0.02327	6.84224	6.1E-06	0.00025	141	333.2	141.6	4	13.8	22.4
Ugt1a5	394433	UDP-glucuronosyltransferase	-52.03	0	0	5.38017	2.3E-07	1.5E-05	4.32572	6.9E-06	0.00028	2251	8218	507.4	73.25	329.2	253
P2ry4	57385	MCG12206	-89.979	0	0	3.50773	0.0014	0.02545	8.83487	1.9E-08	1.5E-06	467.6	262.8	322.6	8.75	26	63.2
4921513D11Rik	67070	Protein 4921513D11Rik	-58.619	0	0	8.06781	4.7E-06	0.00021	12.6608	1.9E-08	1.5E-06	71.8	78.8	49.4	2	14.4	21.6
Plekhb1	27276	Pleckstrin homology domain-containing family B member 1	-81.85	0	0	4.17053	1.5E-07	9.9E-06	6.93862	6.2E-13	1.3E-10	700.8	423	307	14.75	52	83
Hist2h4	97122	Histone H4	-3.3755	7.8E-10	7.6E-09	91.1244	0	0	87.214	0	0	67	123.8	118.8	35	2639	2348
Hist1h2al		Histone H2A	-8.4303	0	0	81.3687	0	0	71.4938	0	0	228.4	208.2	299.8	47.25	3194	2631
Reln	19699	Reelin splicing isoform MR-1A	-10.735	0	0	3.38353	5.8E-06	0.00025	4.75616	6.4E-09	6.1E-07	1503	3882	5488	242.5	684.6	900.6
Esr1	13982	Estrogen receptor	-25.894	0	0	2.32253	0.00034	0.00804	3.27408	4.2E-07	2.5E-05	1149	1359	892.6	75.75	147	199
Gnat1	14685	Guanine nucleotide-binding protein G(t) subunit alpha-1	-41.409	0	0	2.26295	0.00154	0.02728	3.37252	1.9E-06	9.2E-05	498.8	203.8	272.2	21.25	40.4	56.6
Sned1	208777	Sushi, nidogen and EGF-like domain-containing protein 1	-43.041	0	0	2.7185	1.4E-06	7.3E-05	4.35301	8.1E-13	1.6E-10	1047	379.4	473.8	42	95.4	145.6
Ube2d2b	19942	Ubiquitin-conjugating enzyme E2 D2B	-3.7458	6.2E-05	0.00037	21.2029	0	0	21.2194	0	0	12.4	32.2	24.4	5.75	103.8	98.6
4632427E13Rik	666737	MCG147203	-3.3857	2.7E-10	2.8E-09	16.775	0	0	17.7972	0	0	128.2	459.2	431.6	67.75	943.8	944.6
Enpp3	209558	Enpp3 protein	-20.921	0	0	2.15257	0.00172	0.02964	2.37199	0.00041	0.00852	1648	986.6	1330	138	248.2	251.8
Cped1	214642	Uncharacterized protein	-12.999	0	0	1.96333	0.00028	0.00685	2.607	2.3E-07	1.5E-05	2090	2003	2593	282.3	462.8	576
Apob	238055	Apolipoprotein B-100	-9.5584	0	0	3.89449	7.1E-08	5.2E-06	4.26618	8.9E-09	8.1E-07	3E+05	3E+05	3E+05	46809	2E+05	2E+05
Plxnc1	54712	Plexin-C1	-5.7514	4.8E-11	5.2E-10	2.20728	0.00295	0.04566	3.65403	1.1E-06	5.8E-05	886.4	2433	1808	272	503.6	776.6
F8	14069	Coagulation factor VIII	-2.908	4.6E-06	3.1E-05	3.03645	1.8E-06	9.2E-05	3.38188	1.6E-07	1E-05	429.6	2258	1849	258.8	657	672.4
Psd3	234353	PH and SEC7 domain-containing protein 3	-10.332	0	0	2.23245	5.2E-05	0.00169	2.87489	1E-07	7E-06	2337	2523	2495	399	744.6	903.6
Nrp1	18186	Neuropilin-1	-8.1899	0	0	1.82456	0.00013	0.00366	2.0988	2.3E-06	0.00011	2271	2398	3796	494.5	750.6	817.8
Zbtb20	56490	Zinc finger and BTB domain-containing protein 20	-10.987	0	0	3.27933	2.8E-08	2.3E-06	3.57424	2.6E-09	2.6E-07	5155	3242	4674	835.8	2285	2317
Gda	14544	Guanine deaminase	-13.354	0	0	2.10444	0.00042	0.00979	2.53379	1.1E-05	0.0004	1587	892	1048	208.8	365.8	428.6
Ppara	19013	Peroxisome proliferator-activated receptor alpha	-7.5312	0	0	2.06618	6.4E-05	0.00202	2.05682	7.2E-05	0.00199	3800	5120	4575	880.8	1500	1424
Lrp1	16971	Prolow-density lipoprotein receptor-related protein 1	-9.2083	0	0	3.98331	1.7E-07	1.1E-05	3.60063	4.3E-08	3.3E-06	28530	22424	26002	5462	15411	15554
Exph5	320051	Exophilin-5	-8.8142	0	0	3.29505	5.9E-14	1.3E-11	2.41465	3.3E-08	2.5E-06	778	604.2	841.2	155	424.2	290.6
Hgf	15234	Hepatocyte growth factor	-2.0653	0.00384	0.01791	2.22454	0.00139	0.02535	3.86648	6.1E-08	4.5E-06	220	1002	1044	185	343	549
Ptprb	19263	Receptor-type tyrosine-protein phosphatase beta	-3.9752	2E-11	2.2E-10	2.14624	0.00021	0.0054	2.80134	5.5E-07	3.1E-05	2582	6294	5837	1144	2046	2502
Dpysl2	12934	Dihydropyrimidinase-related protein 2	-1.7543	0.00101	0.00512	4.15947	0	0	4.28191	0	0	82.4	469.4	347.8	81.5	283	278.2
Calcrl	54598	Calcitonin receptor-like	-3.0224	3.3E-09	3.1E-08	2.16911	3.4E-05	0.00118	2.55569	5.1E-07	2.9E-05	458.8	1428	1346	264.8	479.4	528.4
Rec114	73673	Meiotic recombination protein REC114	-4.8448	6.6E-05	0.00039	6.08834	4.2E-06	0.00019	3.29803	0.00257	0.03644	42.2	59.4	44.2	15.25	78.6	41.4
Selo	223776	1300018J18Rik protein	-6.4363	0	0	1.63772	0.00147	0.02635	1.67718	0.00086	0.01535	2714	2603	2779	737.5	1003	966.4
Klf12	16597	Krueppel-like factor 12	-6.4294	0	0	2.62045	4.5E-07	2.7E-05	2.69855	2E-07	1.3E-05	294	236.6	275.8	81	175.6	172.8
Atrnl1	226255	Attractin-like protein 1	-5.4829	0	0	1.91095	2.2E-08	1.8E-06	2.33751	2E-13	4.4E-11	1590	1559	1933	507.5	808.4	939.2
Stab2	192188	Scavenger receptor FEEL-2a	-3.0652	4E-08	3.4E-07	2.38925	2E-05	0.00073	2.36943	2.4E-05	0.00079	2081	4537	4186	1193	2366	2164
Slc36a4	234967	Proton-coupled amino acid transporter 4	-6.4629	0	0	1.66251	0.00192	0.03252	1.98425	2.8E-05	0.00092	496.4	432.2	433	136	188.8	213.8
Abca1	11303	ATP-binding cassette sub-family A member 1	-5.4105	0	0	2.74164	1.2E-07	8.2E-06	3.12318	2.2E-09	2.3E-07	10520	9383	10516	3452	7860	8646
Abca6	76184	Putative uncharacterized protein	-4.7	3.1E-09	2.9E-08	2.42408	0.0007	0.01464	2.20114	0.00252	0.03591	7557	8945	9623	2831	5691	4829
Slc39a8	67547	Zinc transporter ZIP8	-5.7597	0	0	1.72737	0.00014	0.00397	1.77934	6.1E-05	0.00176	1207	1236	1133	365.3	526.6	506.6
Dock4	238130	Dock4 protein	-7.1143	0	0	1.67603	0.00041	0.00942	2.25095	2.6E-08	2.1E-06	1337	746.4	991.4	330.3	461	586.6
Adap2	216991	Arf-GAP with dual PH domain-containing protein 2	-7.2451	0	0	1.56363	0.00069	0.01445	1.79792	8.2E-06	0.00033	1077	627.2	741	262	341.2	371.2
Kdr	16542	Vascular endothelial growth factor receptor 2	-2.7527	1.5E-13	2E-12	1.6267	0.00039	0.00909	1.80162	1.8E-05	0.00063	1485	3272	2950	941.3	1279	1330
Osbpl8	237542	Oxysterol-binding protein	-3.8924	0	0	1.81499	7.5E-07	4.3E-05	1.97668	1.5E-08	1.3E-06	2316	2750	3459	1041	1576	1629
Adcy9	11515	Adenylate cyclase type 9	-4.3314	0	0	2.16998	2.2E-06	0.00011	2.45583	4E-08	3.1E-06	720.2	644.6	858.2	292.3	528.8	566.8
Dock1	330662	Dedicator of cytokinesis protein 1	-3.9732	7.8E-16	1.2E-14	1.77315	0.00085	0.01715	2.14802	8.4E-06	0.00033	702.6	809.2	850	310.8	460.8	525.4
Atxn1	20238	Ataxin-1	-3.7997	1.4E-13	1.9E-12	2.41129	1.1E-06	5.9E-05	2.44992	7E-07	3.8E-05	1096	922.2	1387	506.5	1016	981.6
Fam126b	213056	Protein FAM126B	-3.2032	1.1E-16	1.8E-15	1.84065	1.3E-05	0.00053	2.08256	1.7E-07	1.1E-05	1434	1807	2342	804	1230	1315
Lyst	17101	Lysosomal trafficking regulator, isoform CRA_a	-2.4738	1.2E-10	1.2E-09	3.15009	3.3E-16	9.8E-14	2.67003	2.7E-12	4.9E-10	1252	2319	1898	885.5	2333	1860
Ppm1k	243382	Protein phosphatase 1K (PP2C domain containing)	-3.8057	7.4E-15	1.1E-13	2.28885	1.5E-06	7.6E-05	2.01099	4.8E-05	0.00144	2505	2532	2897	1140	2157	1783
Ptprj	19271	Protein-tyrosine-phosphatase	-3.578	0	0	2.03411	2.1E-06	0.00011	1.93124	1.1E-05	0.00041	2777	3606	2860	1362	2314	2081
Stab1	192187	FELE-1	-2.5561	1.8E-09	1.7E-08	1.73247	0.00043	0.00987	1.75551	0.00031	0.00678	1676	3276	2736	1148	1657	1583
Abcc9	20928	Hemi ATP-binding cassette protein SUR2A-55	-1.7612	0.00053	0.00282	1.8019	0.0003	0.00742	2.26457	5.3E-07	3E-05	661.8	1664	1600	657	977	1185
Ppp6r2	71474	Saps2 protein	-3.7428	0	0	1.89715	2.6E-05	0.00093	1.93111	1.5E-05	0.00056	783	760	833	368.3	578.8	555.6
Irs1	16367	Insulin receptor substrate 1	-3.8759	3.3E-16	5.2E-15	2.71005	1.8E-09	1.9E-07	2.55545	1.5E-08	1.3E-06	1089	691.8	1018	490	1105	968.6
Klhl24	75785	Kelch-like protein 24	-2.5722	1.3E-09	1.2E-08	2.08043	2.5E-06	0.00012	2.26586	1.5E-07	9.8E-06	1930	2814	3000	1333	2292	2363
Nbeal1	269198	Protein Nbeal1	-2.1044	1.6E-10	1.6E-09	2.79401	0	0	2.89834	0	0	1452	2435	2367	1215	2824	2746
Shank2	210274	SH3 and multiple ankyrin repeat domains protein 2	-4.3686	1.7E-11	2E-10	2.01641	0.0014	0.02541	2.15455	0.00047	0.00946	730.8	651.2	380	289	485	490.4
Lmbrd2	320506	LMBR1 domain-containing protein 2	-2.5576	9.7E-15	1.4E-13	1.9455	4E-08	3.1E-06	2.16011	2.1E-10	2.7E-08	1455	2034	2197	1001	1616	1698
Atp8b1	54670	Phospholipid-transporting ATPase IC	-2.9581	1.5E-11	1.7E-10	1.64725	0.00191	0.03233	1.6779	0.00129	0.0212	2334	2784	3201	1388	1907	1824
Kansl1l	68691	1110028C15Rik protein	-3.0489	5.2E-14	7.1E-13	1.82435	5E-05	0.00164	2.07143	9E-07	4.8E-05	464.8	446.4	546.8	266.8	405.6	431.4
Fam46c	74645	Protein FAM46C	-2.467	0.00047	0.00253	3.96564	9.2E-08	6.5E-06	3.40732	2E-06	9.7E-05	438.6	394	331.8	311.5	1029	871
Myo5b	17919	Unconventional myosin-Vb	-3.2522	1.1E-16	1.8E-15	1.7785	4.6E-05	0.00152	1.89019	6.7E-06	0.00027	1086	1207	931.2	585.5	868	882.4
Pcsk5	18552	Proprotein convertase subtilisin/kexin type 5	-3.8494	1.2E-12	1.4E-11	2.09928	9.4E-05	0.00279	2.16829	4.6E-05	0.00138	544	498.4	263.8	244.5	428.6	418.4
Pkhd1	241035	Protein Pkhd1	-1.9414	1.3E-06	9.6E-06	3.59221	0	0	3.22677	0	0	326	407.2	366	291.8	873.6	741.2
Foxn3	71375	Forkhead box protein N3	-1.3716	0.00659	0.02955	3.25249	0	0	3.25272	0	0	450	778	928.2	578.5	1558	1463
Gprin3	243385	RIKEN cDNA C030038J10	-1.9577	2.3E-07	1.8E-06	2.43182	6.8E-12	1.2E-09	2.90951	1.1E-16	3.4E-14	1219	1312	2206	1107	2243	2539
Slc35e2	320541	Solute carrier family 35 member E2	-3.3709	0	0	1.52717	0.00036	0.00842	1.51769	0.00044	0.00889	2737	2532	2575	1422	1810	1695
Tbck	271981	TBC domain-containing protein kinase-like protein	-1.8037	9.3E-08	7.5E-07	2.51591	0	0	2.52888	0	0	647.6	1236	888	629	1318	1244
Tnfrsf14	230979	Protein Tnfrsf14	-2.7302	6.8E-06	4.5E-05	2.90919	1.4E-06	7.5E-05	2.42359	6.9E-05	0.00193	101.4	89.8	90.8	65	157	127
Yod1	226418	YOD1 OTU deubiquitinating enzyme 1 homologue (S. cerevisiae)	-2.3311	2E-08	1.8E-07	1.59994	0.00184	0.03137	1.74305	0.00023	0.00526	617.2	807.8	868.8	468.5	623.8	643.2
Mgam	232714	Protein Mgam	-2.5579	5.2E-11	5.6E-10	2.11589	1.6E-07	1.1E-05	1.84017	2E-05	0.0007	5428	5904	5950	3715	6525	5298
Tacc1	15267	Transforming acidic coiled-coil-containing protein 1	-3.1603	0	0	1.45939	0.00094	0.01844	1.98717	1.8E-09	1.9E-07	2131	1556	2119	1196	1450	1878
Sp4	20688	Transcription factor Sp4	-2.168	3.5E-08	2.9E-07	1.79793	2.7E-05	0.00097	2.15067	4E-08	3.1E-06	239	281.2	349.6	192.8	288	328.4
Zhx3	320799	Zinc fingers and homeoboxes protein 3	-2.548	3.3E-09	3E-08	2.59053	1.7E-09	1.8E-07	2.20899	5.3E-07	3E-05	3007	2597	3007	2077	4473	3563
Itpr2	16439	Inositol 1,4,5-trisphosphate receptor type 2	-2.7209	5.2E-12	6.1E-11	2.28634	1.2E-08	1E-06	2.08421	4.1E-07	2.4E-05	2567	2534	2112	1659	3145	2665
Wdfy2	268752	WD repeat and FYVE domain-containing protein 2	-2.3369	1.1E-08	9.4E-08	1.81847	5.4E-05	0.00173	1.86093	2.8E-05	0.00091	230.4	275.8	280.4	172.8	261.4	253.2
Alas2	11656	5-aminolevulinate synthase	-2.4459	0.00032	0.00177	2.58255	0.00014	0.0038	2.30687	0.00078	0.01414	1105	1018	1012	780.3	1683	1481
Dst	13518	Dystonin	-2.0921	4.4E-07	3.4E-06	2.74066	5.1E-12	9.1E-10	2.17971	9.7E-08	6.7E-06	4004	5514	3817	3418	7760	5743
Nfib	18028	Nuclear factor 1 B-type	-2.3849	1.9E-10	2E-09	2.23691	3.6E-09	3.5E-07	2.05211	1.4E-07	9.2E-06	2920	2853	3176	2136	3972	3420

S4 Table. Continue...

Name	NCBI gene ID	Database object name	NT vs NL			MT vs NT			LT vs NT			Mean					
			Fold Change	P-Value	FDR P-Value	Fold Change	P-Value	FDR P-Value	Fold Change	P-Value	FDR P-Value	NL	ML	LL	NT	MT	LT
Itga1	109700	Itga1 protein	-1.6551	6.9E-05	0.00041	2.25234	1.2E-10	1.6E-08	2.13061	2.1E-09	2.1E-07	898.6	1562	1319	948.5	1779	1573
Vps13d	230895	Protein Vps13d	-2.068	2.1E-08	1.9E-07	2.64926	5.7E-14	1.3E-11	2.37682	2.5E-11	3.9E-09	3819	4333	3939	3257	7161	6032
Ogn	18295	Osteoglycin	-2.3929	4.2E-05	0.00026	3.05215	1.2E-07	8.2E-06	2.68789	2.9E-06	0.00013	92	49.6	85	66.5	168.6	138
Chpf2	100910	MCG16489, isoform CRA_a	-3.4405	0	0	1.50641	0.00137	0.02501	1.70247	3.2E-05	0.00103	1311	899.4	879.8	666	833.4	904.8
Nf1	18015	Neurofibromin	-1.588	0.00031	0.00168	2.41727	4.8E-12	8.4E-10	2.61886	4.7E-14	1.1E-11	1079	1431	1536	1201	2410	2468
Hook3	320191	Protein Hook homolog 3	-2.8035	0	0	1.7466	4E-06	0.00018	1.66338	2.6E-05	0.00086	3744	3267	3000	2334	3393	3018
Dennd5b	320560	DENN domain-containing protein 5B	-2.214	4.9E-11	5.3E-10	1.79022	1.5E-06	7.6E-05	1.90704	9.3E-08	6.5E-06	3653	4078	3980	2893	4313	4331
Herc2	15204	E3 ubiquitin-protein ligase HERC2	-1.4952	0.00176	0.00865	2.73152	5E-15	1.2E-12	2.61441	7.2E-14	1.7E-11	2298	3122	2810	2706	6135	5517
Pikfyve	18711	1-phosphatidylinositol 3-phosphate 5-kinase	-1.4385	0.00038	0.00204	2.24277	2E-15	5.3E-13	2.10234	2.9E-13	6.2E-11	1098	1772	1661	1337	2499	2203
Kif13b	16554	Kinesin-like protein	-1.9022	3.5E-08	2.9E-07	2.48871	4.4E-15	1.1E-12	2.20254	1.2E-11	2E-09	1497	1555	1508	1384	2862	2386
Usp9x	22284	Probable ubiquitin carboxyl-terminal hydrolase FAF-X	-1.6131	0.00969	0.04203	2.31214	5.8E-06	0.00025	2.34459	4E-06	0.00018	7338	8787	9696	8001	15377	14743
Dennd1b	329260	ASL1/9AS1-1 fusion	-1.5347	6.4E-05	0.00039	2.03764	2.5E-11	3.8E-09	1.99626	9.3E-11	1.3E-08	887.4	1265	1343	1022	1732	1594
AU041133	216177	Protein AU041133	-1.8541	0.0023	0.01111	1.98478	0.00065	0.01382	1.84338	0.0024	0.03443	117	124.6	141.8	111.8	184.4	160.8
4932438A13Rik	229227	Protein 4932438A13Rik	-1.7037	0.0007	0.00365	2.47708	7.8E-09	7.1E-07	2.15489	1E-06	5.4E-05	3379	4262	3453	3495	7167	5839
Kdm7a	338523	Putative uncharacterized protein	-2.0636	6.7E-10	6.6E-09	1.49624	0.00059	0.01279	1.94488	1.4E-08	1.2E-06	1038	1062	1169	893	1109	1366
Dstyk	213452	Dual serine/threonine and tyrosine protein kinase	-2.7539	1.1E-16	1.8E-15	1.45146	0.00237	0.03828	1.49921	0.00096	0.01683	642.4	511.4	484.2	409.8	494.2	480.2
Akap13	320165	Protein Akap13	-1.817	1.7E-06	1.2E-05	2.30933	1.9E-11	3E-09	2.30487	2.2E-11	3.5E-09	2516	2361	2344	2437	4667	4397
Wdr7	104082	WD repeat-containing protein 7	-1.7783	3.6E-08	3E-07	1.85591	3E-09	3E-07	1.91364	4.8E-10	5.7E-08	1210	1430	1315	1195	1847	1801
Elk4	13714	Elk4 transcript variant c	-1.8627	3.5E-07	2.7E-06	2.04878	3.9E-09	3.8E-07	1.91566	9.6E-08	6.7E-06	1312	1270	1366	1243	2117	1838
Sash1	70097	SAM and SH3 domain-containing protein 1	-2.2599	1.6E-12	2E-11	1.45285	0.00122	0.02271	1.48806	0.00058	0.01115	1867	1825	1520	1458	1759	1704
Map3k2	26405	Mitogen-activated protein kinase kinase kinase 2	-1.8938	3.1E-08	2.6E-07	1.51601	0.00031	0.00745	1.89806	2.7E-08	2.1E-06	1390	1379	1475	1286	1621	1938
Pik3r1	18708	Phosphatidylinositol 3-kinase regulatory subunit alpha	-2.3694	2.5E-12	3E-11	1.48481	0.00133	0.0244	1.47298	0.00167	0.02592	3678	2383	3537	2741	3381	3162
Zfp759	268670	Protein Zfp759	-1.5812	0.00325	0.01535	1.70738	0.00053	0.01173	1.60199	0.00233	0.0336	185.2	209.8	258.8	206.8	293.8	258.4
Nfia	18027	Nuclear factor 1	-2.6582	1.6E-09	1.5E-08	1.71928	0.00082	0.01659	1.70253	0.00102	0.01762	1845	1096	1090	1211	1727	1604
Mcc	328949	Protein Mcc	-1.5758	0.00495	0.02272	2.59145	3.7E-09	3.7E-07	1.86617	0.00011	0.0029	1024	1063	960.8	1123	2422	1626
Zbtb38	245007	Protein Zbtb38	-1.574	3.6E-05	0.00023	2.00904	1.8E-10	2.3E-08	1.81306	5.6E-08	4.1E-06	927.6	1057	1002	1030	1722	1456
Zfp871	208292	RIKEN cDNA 9030612M13	-1.5152	0.00085	0.00435	2.1246	1.3E-09	1.4E-07	2.15527	6.3E-10	7.1E-08	1052	903.4	1206	1225	2158	2059
Bmpr2	12168	Bone morphogenetic protein receptor type-2	-1.3475	0.00995	0.04305	1.83668	1.4E-07	9.4E-06	1.95744	5.9E-09	5.6E-07	1440	1730	1928	1882	2873	2883
Nbas	71169	Protein Nbas	-1.7634	4E-07	3.1E-06	1.7635	3.8E-07	2.3E-05	1.60978	2.1E-05	0.00071	1501	1536	1371	1491	2183	1894
Itpr1	16438	Inositol 1,4,5-trisphosphate receptor type 1	-1.606	0.00023	0.00128	1.97452	1.2E-07	8.2E-06	1.69833	3.8E-05	0.00116	2532	2913	2353	2793	4584	3679
Lcorl	209707	Ligand-dependent nuclear receptor corepressor-like protein	-1.3673	0.00418	0.0194	2.00993	1.2E-10	1.6E-08	1.82663	2.8E-08	2.2E-06	577	707.4	663.6	742	1243	1063
Rabgap1l	29809	Rab GTPase-activating protein 1-like	-1.472	0.00034	0.00184	1.87105	5.6E-09	5.3E-07	1.68377	1.3E-06	6.6E-05	1292	1679	1289	1535	2387	2007
Nr3c1	14815	Glucocorticoid receptor	-1.8504	9.3E-08	7.6E-07	1.45304	0.00118	0.0222	1.39773	0.00367	0.0482	3864	3456	4176	3675	4438	4039
Zfp729b	1E+08	Protein Zfp729b	-1.5068	0.00074	0.00381	1.44207	0.00245	0.03928	1.44975	0.00214	0.03155	388.4	463.2	510.8	453.8	544.6	512.2
Zfp780b	338354	Protein Zfp780b	-1.5614	0.00048	0.00256	1.74063	1.2E-05	0.00049	1.89332	4.8E-07	2.8E-05	378	350.8	416.6	426.5	616.6	635.6
Trip11	109181	Thyroid hormone receptor interactor 11	-1.7272	2E-06	1.5E-05	1.62017	2.7E-05	0.00097	1.76149	8.4E-07	4.5E-05	1836	1798	1661	1884	2534	2572
Mier3	218613	Mier3 protein	-1.6263	5.1E-05	0.00031	1.61054	6.8E-05	0.00211	1.85279	2.5E-07	1.5E-05	667.4	629.6	695.4	721.8	969.4	1050
Fat1	14107	Protein Fat1	-1.5415	0.00724	0.03218	1.96796	2.6E-05	0.00094	2.17218	1.4E-06	7.3E-05	1866	1582	1768	2156	3525	3659
Ccser2	72972	Serine-rich coiled-coil domain-containing protein 2	-1.9596	1.5E-10	1.6E-09	1.43286	0.00061	0.01322	1.36163	0.00331	0.04459	1835	1687	1527	1645	1964	1765
Magi3	99470	Membrane-associated guanylate kinase, WW and PDZ domain-co	-1.5417	2.6E-05	0.00016	1.72006	1.2E-07	8.6E-06	1.79243	1.3E-08	1.1E-06	1431	1479	1470	1627	2328	2283
BC005561	1E+08	Protein BC005561	-1.511	0.00441	0.02038	1.9897	1.8E-06	9.5E-05	2.15847	9.6E-08	6.7E-06	456.4	378	428.6	537	888.2	887.8
Fam208a	218850	Protein FAM208A	-1.2983	0.00838	0.03679	1.8087	1.9E-09	2E-07	1.77013	7.2E-09	6.7E-07	1315	1583	1553	1776	2672	2459
Hist1h2bc	68024	Histone H2B type 1-C/E/G	-1.6292	0.00074	0.00382	1.86948	1.5E-05	0.00057	1.62915	0.00074	0.01361	3289	2475	3290	3585	5537	4515
Golgb1	224139	Golgb1 protein	-2.1221	3.7E-09	3.5E-08	1.48538	0.00193	0.03269	1.63193	0.00012	0.00315	3624	2382	2368	2987	3681	3749
Zzef1	195018	Zinc finger ZZ-type and EF-hand domain-containing protein 1	-1.7174	4E-06	2.8E-05	1.70996	4.7E-06	0.00021	1.73486	2.6E-06	0.00012	2154	1710	1755	2207	3136	3011
Tmem170b	621976	Transmembrane protein 170B	-1.3943	0.0022	0.01065	1.7001	9.2E-07	5.1E-05	1.36804	0.00382	0.04967	969.2	1326	959.4	1221	1726	1296
Klhl28	66689	Kelch-like protein 28	-1.5084	0.00775	0.03426	1.60034	0.00212	0.03512	1.68175	0.00069	0.01286	172.8	178.6	169.8	202.3	269.2	265.4
Zkscan1	74570	Zinc finger protein with KRAB and SCAN domains 1	-1.4613	0.00169	0.00833	2.06717	1.7E-09	1.9E-07	2.06702	1.8E-09	1.9E-07	2111	1516	1774	2536	4349	4063
Zfyve9	230597	Zinc finger FYVE domain-containing protein 9 splice variant Z2.1	-1.4888	0.00019	0.00108	1.5964	1.1E-05	0.00044	1.57347	2E-05	0.00071	953.8	899.8	1015	1127	1496	1379
Trim2	80890	Tripartite motif-containing protein 2	-1.4466	0.00218	0.01057	1.75098	3.2E-06	0.00015	1.64092	3.8E-05	0.00119	1671	2232	1084	2047	2973	2626
Pik3ca	18706	Phosphatidylinositol 3-kinase, catalytic, alpha polypeptide, isofor	-1.3776	0.00239	0.01151	1.40488	0.00124	0.02316	1.39578	0.00155	0.02452	1544	1922	1798	1968	2299	2148
Zfp672	319475	Zinc finger protein 672	-1.7331	7E-06	4.7E-05	1.45657	0.00208	0.03453	1.43477	0.00315	0.04291	625	490.8	546.6	634	768.6	715.6
Lpp	210126	Lipoma-preferred partner homolog	-1.5608	0.00305	0.01448	1.77967	0.00012	0.00353	1.59732	0.00182	0.02779	2475	2118	2073	2784	4096	3487
Magi1	14924	Magi1 protein	-1.6927	1.8E-06	1.3E-05	1.4329	0.00109	0.02069	1.57354	3.8E-05	0.00118	1228	1054	995	1274	1517	1570
Epg5	1.01E+08	Ectopic P granules protein 5 homolog	-1.4316	0.00155	0.00766	1.77983	3.4E-07	2.1E-05	1.58368	4.8E-05	0.00143	1409	1335	1256	1731	2558	2154
Dennd4c	329877	DENN domain-containing protein 4C	-1.5427	6.2E-05	0.00037	1.55582	4.3E-05	0.00144	1.5049	0.00016	0.0038	1759	1519	1632	2023	2615	2384
Plxna2	18845	Plexin-A2	-1.5869	0.00098	0.00499	1.67778	0.00022	0.0056	1.63896	0.00041	0.00855	1361	1292	874.6	1491	2082	1925
Rb1cc1	12421	RB1-inducible coiled-coil 1	-1.366	0.00481	0.02213	1.45178	0.00074	0.01532	1.58693	2.9E-05	0.00095	2117	2107	2327	2737	3299	3389
Shroom2	110380	Protein Shroom2	-1.4436	0.002	0.00971	1.53827	0.00028	0.00687	1.47075	0.00114	0.01923	993.6	772.4	983	1225	1565	1415
Zfp799	240064	Protein Zfp799	-1.3807	0.01133	0.04861	1.53065	0.00075	0.01554	1.52734	0.00081	0.01472	294	249.6	278.8	374.5	476	446.8
Zfp652	268469	Zinc finger protein 652	-1.4375	0.00164	0.00807	1.46783	0.00086	0.01719	1.4749	0.00074	0.01362	2115	1865	1800	2583	3150	2983
Pkd1	18763	Polycystin-1	-1.3812	0.00669	0.02993	1.71191	6E-06	0.00026	1.58865	9.8E-05	0.00259	1224	1011	990.6	1561	2214	1972
Zfhx4	80892	Zinc finger homeobox protein 4	-1.6288	0.00122	0.00611	1.91928	1.5E-05	0.00057	1.74721	0.00021	0.00487	899.6	451.8	473.8	964	1536	1313
Vcpip1	70675	Vcpip1 protein	-1.4371	0.00149	0.0074	1.41247	0.00246	0.03939	1.55026	0.00012	0.00307	1974	1475	1744	2423	2845	2947
Dyrk2	69181	Dual specificity tyrosine-phosphorylation-regulated kinase 2	-1.4187	0.00147	0.00728	1.50291	0.0002	0.00526	1.44493	0.00079	0.01431	755.4	549.6	615.4	934.5	1165	1058
Rbm47	245945	RNA-binding protein 47	-1.605	0.0027	0.01291	1.67619	0.00105	0.0201	1.75617	0.00035	0.00753	2502	1203	1441	2774	3865	3853
Baz2b	407823	Baz2b protein	-1.3619	0.00497	0.0228	1.43046	0.00108	0.02059	1.65981	3.7E-06	0.00016	731.2	527.6	534	949.5	1127	1229
Lsm6	78651	U6 snRNA-associated Sm-like protein LSm6	1.35056	0.00706	0.03141	-1.4813	0.00041	0.00955	-1.4747	0.00048	0.0097	844.6	573.2	707	2003	1126	1060
Ube2d2a	56550	MCG121966	1.35796	0.0077	0.03404	-1.4582	0.00101	0.01958	-1.4349	0.00165	0.02572	2840	1982	1922	6798	3876	3710
Pdcl3	68833	Phosducin-like protein 3	1.38027	0.00892	0.03897	-1.4511	0.00243	0.039	-1.4499	0.0025	0.03567	528	421.4	309.2	1279	733.4	688.2
Ttc9c	70387	Tetratricopeptide repeat protein 9C	1.3872	0.00108	0.00545	-1.3597	0.00207	0.03442	-1.3816	0.0012	0.02014	957.8	615.4	605.8	2328	1424	1326
Mrps34	79044	28S ribosomal protein S34, mitochondrial	1.35997	0.0092	0.04008	-1.4211	0.00286	0.04459	-1.5353	0.00028	0.00614	1314	799.8	884.8	3142	1837	1575
Pitpna	18738	Phosphatidylinositol transfer protein alpha isoform	1.47497	0.00046	0.00248	-1.4128	0.00183	0.03118	-1.3819	0.00354	0.04681	1979	1395	1118	5113	3016	2845
Sec11a	56529	Signal peptidase complex catalytic subunit SEC11	1.48111	0.00045	0.00241	-1.442	0.00106	0.02024	-1.5872	3.6E-05	0.00113	1926	1130	1453	4995	2883	2460
Tmem9	66241	Transmembrane protein 9	1.6819	4E-05	0.00024	-1.6101	0.00015	0.00423	-1.5181	0.00092	0.01615	422.2	395.8	308	1242	639.8	649.8
Atp1b3	11933	Sodium/potassium-transporting ATPase subunit beta-3, Na K-ATP	1.69931	2.2E-07	1.8E-06	-1.4113	0.00073	0.01524	-1.3749	0.00181	0.02769	1179	897.6	880.4	3528	2081	2020
Zranb2	53861	Zinc finger Ran-binding domain-containing protein 2	1.34337	0.00803	0.03538	-1.4735	0.00049	0.01106	-1.4972	0.00029	0.00631	2328	1238	1371	5527	3125	2887
Cmtm8	70031	CKLF-like MARVEL transmembrane domain-containing protein 8	1.34045	0.0064	0.02873	-1.4708	0.00032	0.00776	-1.7636	1.3E-07	8.7E-06	1106	605.6	724.8	2599	1465	1154
Sdhaf1	68332	Succinate dehydrogenase assembly factor 1, mitochondrial	1.46795	0.00627	0.02823	-1.5509	0.00173	0.02977	-1.6841	0.0002	0.00471	500.6	337.4	349.8	1283	689.6	587.8
Pkig	18769	cAMP-dependent protein kinase inhibitor gamma	1.54866	0.00022	0.00124	-1.5798	0.0001	0.00305	-1.5159	0.00042	0.00858	444.6	339.6	295.4	1204	633.8	624.6
Akirin2	433693	Akirin-2	1.4227	0.00154	0.0076	-1.4651	0.00057	0.01248	-1.4224	0.00149	0.02382	678.2	386.4	414	1707	967.6	938.8
Mrpl52	68836	39S ribosomal protein L52, mitochondrial	1.68183	8.3E-06	5.5E-05	-1.4253	0.00229	0.03729	-1.584	7.7E-05	0.00211	893.8	706	679.8	2644	1542	1305
Traf4	22032	TNF receptor-associated factor 4	1.446	0.00055	0.00292	-1.4537	0.00044	0.01015	-1.4601	0.00038	0.00806	1207	772.8	701.8	3070	1755	1642

S4 Table. Continue...

Name	NCBI gene ID	Database object name	NT vs NL			MT vs NT			LT vs NT			Mean					
			Fold Change	P-Value	FDR P-Value	Fold Change	P-Value	FDR P-Value	Fold Change	P-Value	FDR P-Value	NL	ML	LL	NT	MT	LT
Cd2bp2	70233	Putative uncharacterized protein	1.37814	0.00155	0.00766	-1.377	0.00155	0.02745	-1.3423	0.00359	0.04741	1182	564.4	644.2	2866	1732	1671
Oser1	66680	Oxidative stress-responsive serine-rich protein 1	1.5107	0.00035	0.00189	-1.6034	4.1E-05	0.00137	-1.5606	0.00011	0.00284	872.2	576.4	595	2314	1199	1162
Pno1	66249	RNA-binding protein PNO1	1.58568	4.7E-05	0.00029	-1.5291	0.00017	0.00465	-1.5481	0.00011	0.00287	1229	752	938.8	3435	1871	1744
Scpep1	74617	Putative uncharacterized protein	1.37074	0.00389	0.01813	-1.565	4E-05	0.00137	-1.7964	8.1E-08	5.8E-06	2044	1582	908	4877	2590	2158
Zdhhc16	74168	Probable palmitoyltransferase ZDHHC16	1.47874	0.00055	0.0029	-1.4234	0.00173	0.02977	-1.4239	0.00172	0.02658	567.2	287.4	327.8	1475	861	813.4
Ubp1	22221	Ubp1 protein	1.34014	0.00735	0.03261	-1.4419	0.0008	0.01624	-1.5131	0.00015	0.00363	2462	1175	1226	5810	3345	3031
Cdc123	98828	Cell division cycle protein 123 homolog	1.74017	2E-08	1.8E-07	-1.4056	0.00054	0.01195	-1.3908	0.00081	0.01462	1477	974.4	982.8	4512	2668	2525
H2afj	232440	Histone H2A.J	1.82138	3.6E-08	3E-07	-1.392	0.00225	0.03673	-1.6335	6E-06	0.00025	777	578.6	609.2	2499	1491	1197
Zwint	52696	ZW10 interactor	1.53591	4.9E-05	0.0003	-1.4352	0.00062	0.01325	-1.6528	2E-06	9.5E-05	2011	1476	986	5426	3138	2556
1110004F10Rik	56372	MCG7316, isoform CRA_b	1.95037	3.8E-10	3.9E-09	-1.3785	0.00254	0.04056	-1.3992	0.00159	0.02505	1334	973.6	1027	4575	2762	2548
Arpc4	68089	Actin-related protein 2/3 complex subunit 4	1.64988	2.9E-06	2E-05	-1.4792	0.00025	0.00631	-1.607	9.2E-06	0.00036	1919	1255	1235	5551	3122	2755
Anapc11	66156	Anaphase-promoting complex subunit 11	1.6697	0.00012	0.00072	-1.6301	0.00024	0.00606	-1.5964	0.00044	0.00891	416	268.8	301.2	1218	622.4	608.4
Rfxap	170767	Regulatory factor X-associated protein	1.42163	0.00303	0.01438	-1.5378	0.00027	0.00674	-1.5155	0.00044	0.00892	454	221.4	234.4	1133	612.4	586
Zcrb1	67197	Zinc finger CCHC-type and RNA-binding motif-containing protein 1	1.49675	0.00035	0.00191	-1.6842	3.6E-06	0.00017	-1.6353	1.3E-05	0.00047	737	422.8	437.6	1947	960	913.6
Ndufab1	70316	Acyl carrier protein	1.6969	9.1E-06	6E-05	-1.451	0.00176	0.03028	-1.6202	5.1E-05	0.00149	3021	2041	1839	9030	5169	4312
Dda1	66498	DET1- and DDB1-associated protein 1	1.40617	0.00198	0.00966	-1.5049	0.0002	0.00525	-1.5404	8.5E-05	0.0023	672.4	299	319.6	1663	918.6	851
Ap3s2	20637	Adaptor-related protein complex 3, sigma 2 subunit	1.34317	0.003	0.01427	-1.4911	5.7E-05	0.00181	-1.6232	1.1E-06	5.6E-05	1134	472	511.8	2676	1491	1291
Phf5a	68479	PHD finger-like domain-containing protein 5A	1.92318	3.8E-10	3.8E-09	-1.3822	0.00183	0.03122	-1.3717	0.00236	0.03393	891.2	562	592.6	3011	1813	1735
Ciapin1	109006	Anamorsin	1.4447	0.00048	0.00258	-1.4319	0.00064	0.01369	-1.7037	4.2E-07	2.5E-05	1331	693.2	609.4	3373	1961	1556
Dnttip1	76233	Deoxynucleotidyltransferase terminal-interacting protein 1	1.53038	0.00021	0.0012	-1.4238	0.00197	0.03319	-1.539	0.00016	0.00395	446.8	227	217.8	1203	702	610.8
Mfsd5	106073	Molybdate-anion transporter	1.35162	0.00691	0.03083	-1.6446	7.8E-06	0.00033	-1.6786	3.3E-06	0.00015	589.8	291.2	268.2	1400	708.4	662.4
Atp6v0e	11974	Atp6v0e protein	1.67677	5E-05	0.0003	-1.7932	4.4E-06	0.0002	-1.8091	3.2E-06	0.00014	879.2	660.6	591.2	2595	1204	1143
Polr2m	28015	DNA-directed RNA polymerase II subunit GRINL1A	1.70473	1.1E-06	8.1E-06	-1.5485	6.4E-05	0.00201	-1.6427	5.7E-06	0.00024	2790	1686	1872	8341	4470	3994
Pigu	228812	Phosphatidylinositol glycan anchor biosynthesis, class U	1.41912	0.00213	0.01033	-1.4247	0.00181	0.03096	-1.5282	0.00019	0.00446	544.4	235.2	224	1354	789.6	695.2
Senp3	80886	Sentrin-specific protease 3	1.88542	6.5E-10	6.4E-09	-1.3522	0.00317	0.04818	-1.3578	0.00279	0.03893	1361	761.6	817.6	4513	2780	2639
Phf23	78246	PHD finger protein 23	1.70728	1.2E-06	8.7E-06	-1.4337	0.00101	0.01962	-1.378	0.00345	0.04601	782.8	405.2	419.6	2348	1364	1339
Pbx2	18515	Pre-B-cell leukemia transcription factor 2	1.58315	6.2E-05	0.00038	-1.4561	0.00101	0.01961	-1.4553	0.00104	0.01789	753.4	362.6	380.4	2092	1196	1142
Arl6ip5	65106	PRA1 family protein 3	1.5513	6.3E-05	0.00038	-1.7602	2.5E-07	1.6E-05	-1.9347	1.8E-09	1.9E-07	1074	659.6	670.2	2931	1386	1171
Ubald2	319370	Fam100b protein	1.55754	0.00241	0.01159	-1.7719	8.7E-05	0.00263	-1.6168	0.00099	0.01714	754	423	427.2	2063	964.4	1020
Ywhab	54401	14-3-3 protein beta/alpha	1.39885	0.00554	0.02517	-1.6268	5.7E-05	0.00183	-1.5064	0.00071	0.01315	4289	1782	1827	10534	5382	5488
Arglu1	234023	Putative uncharacterized protein	1.48065	0.00048	0.00257	-1.5493	9.7E-05	0.00288	-1.4819	0.00046	0.00932	2252	946.6	1018	5848	3146	3059
Cdk16	18555	Cyclin-dependent kinase 16	1.30289	0.00756	0.03349	-1.496	4.7E-05	0.00153	-1.5584	7.4E-06	0.0003	1407	501.4	473.2	3211	1784	1617
Hypk	67693	Huntingtin-interacting protein K	1.51228	0.00129	0.00646	-1.5656	0.00047	0.01053	-1.6469	0.0001	0.00263	523	227	277.2	1385	736.6	661.4
Lsm12	268490	Protein LSM12 homolog	1.60727	5.6E-06	3.8E-05	-1.4503	0.00036	0.00845	-1.4612	0.00027	0.00606	1027	479.2	479.6	2910	1668	1562
Zmat2	66492	Zinc finger matrin-type protein 2	1.53491	0.00044	0.00236	-1.6924	1.5E-05	0.00059	-1.5455	0.00035	0.00742	1112	549	595.2	3001	1478	1498
Ift52	245866	Intraflagellar transport protein 52 homolog	1.37367	0.00544	0.02477	-1.5885	4.9E-05	0.00161	-2.0254	6.6E-10	7.5E-08	971.2	431.8	476.4	2341	1222	893.2
B4galt3	57370	Beta-1,4-galactosyltransferase 3	1.71839	1.2E-06	8.5E-06	-1.4516	0.00074	0.01532	-1.4513	0.00075	0.01388	415.6	231.6	197.4	1254	718.6	683.4
Vat1	26949	Putative uncharacterized protein	1.57099	0.00021	0.00119	-1.5541	0.00029	0.00712	-1.6209	7.2E-05	0.00201	1037	601.6	460.2	2865	1530	1410
Fam192a	102122	2310065K24Rik protein	1.45685	0.0006	0.00317	-1.5898	2.3E-05	0.00083	-1.5983	1.8E-05	0.00065	933.6	385	437	2383	1245	1174
Eif3h	68135	Eukaryotic translation initiation factor 3 subunit H	2.11749	7.3E-09	6.6E-08	-1.5045	0.00162	0.02835	-1.4833	0.00235	0.03388	3161	2377	2168	11762	6491	6141
Mrpl22	216767	39S ribosomal protein L22, mitochondrial	2.12794	1.1E-11	1.3E-10	-1.4404	0.00095	0.01871	-1.556	6.4E-05	0.00181	674.4	463.2	519	2521	1457	1265
Sypl	19027	Synaptophysin-like protein 1	1.3674	0.00236	0.01135	-1.6426	1.4E-06	7.3E-05	-1.9121	3E-10	3.7E-08	2248	1039	961.6	5378	2721	2203
Taf1a	21339	TATA box-binding protein-associated factor RNA polymerase I sub	1.85202	3.5E-06	2.4E-05	-1.479	0.00286	0.04456	-1.4699	0.00337	0.04523	218.4	130.8	121.2	711.5	400.4	378
Tnip1	57783	TNFAIP3-interacting protein 1	1.46618	0.01083	0.04662	-1.7835	0.00011	0.00328	-1.7898	0.0001	0.00272	389.8	231.4	173	1008	469	438
Fam162a	70186	Protein FAM162A	1.72956	7E-05	0.00042	-1.7059	0.0001	0.00307	-1.8011	1.9E-05	0.00067	1378	908.2	833.8	4201	2045	1817
Fam104a	28081	Protein Fam104a	1.58064	0.00146	0.00726	-1.5862	0.00131	0.02409	-1.8678	1.4E-05	0.00051	583.6	358.8	271.2	1623	850.6	674.8
Acbd6	72482	Acyl-CoA-binding domain-containing protein 6	2.16758	2.6E-14	3.6E-13	-1.349	0.00295	0.04566	-1.4828	9.3E-05	0.00248	693.4	586.8	363.2	2636	1621	1395
Tiprl	226591	TIP41-like protein	2.21749	5.6E-15	8.1E-14	-1.4038	0.00079	0.01613	-1.5028	5.7E-05	0.00164	668.2	497.4	480.8	2610	1544	1357
Ppp3r1	19058	Calcineurin subunit B type 1	1.75203	8E-07	5.9E-06	-1.4588	0.00087	0.01749	-1.5174	0.00024	0.00542	1925	936.8	1053	5954	3391	3058
Slc7a6os	66432	Probable RNA polymerase II nuclear localization protein SLC7A6OS	1.84925	2E-08	1.8E-07	-1.4126	0.00153	0.02722	-1.4698	0.00042	0.00857	738.8	382.6	378.6	2407	1416	1273
Vps72	21427	Vacuolar protein sorting-associated protein 72 homolog	1.50135	0.00014	0.0008	-1.5281	6.5E-05	0.00203	-1.6238	5.1E-06	0.00022	545.2	221	227	1437	782.4	690.6
Rheb	19744	RHEB-like protein	1.90585	2.4E-10	2.5E-09	-1.5034	6E-05	0.00189	-1.5757	7.7E-06	0.00031	1544	855	985	5183	2866	2574
Ube2n	93765	MCG4297	1.81888	9E-08	7.3E-07	-1.6168	1.7E-05	0.00064	-1.5898	3.3E-05	0.00105	1624	875.8	1002	5203	2677	2553
Haus2	66296	HAUS augmin-like complex subunit 2	2.50692	7.3E-14	9.9E-13	-1.5	0.00087	0.01735	-1.5499	0.00032	0.00696	439.8	363.4	388.4	1946	1078	985.8
Daxx	13163	Fas death domain-associated protein	1.87162	1E-08	9.3E-08	-1.4737	0.00036	0.00859	-1.3785	0.00317	0.04314	570.2	266.8	298	1874	1058	1070
Gmpr2	105446	GMP reductase 2	1.62493	0.00017	0.00099	-1.5648	0.00051	0.01128	-1.674	6.4E-05	0.00183	477.2	209.4	245.2	1362	724.2	633.8
Dgcr14	27886	Protein DGCR14	1.76078	6.5E-06	4.3E-05	-1.5042	0.00099	0.01927	-1.5411	0.0005	0.00991	231.2	107.6	121.6	714.8	395.2	362.4
Smg8	74133	Protein SMG8	1.88648	8.5E-08	6.9E-07	-1.4712	0.00103	0.01991	-1.4914	0.00069	0.0129	483.2	226	294.4	1610	911	851.2
Wasf2	242687	Wiskott-Aldrich syndrome protein family member 2	1.67687	3E-06	2.1E-05	-1.4307	0.00116	0.02192	-1.5798	3.4E-05	0.00108	1254	565	539.8	3691	2140	1834
Mark4	232944	Putative uncharacterized protein	1.37542	0.00946	0.04112	-1.5352	0.00047	0.01053	-1.791	2.1E-06	9.9E-05	571.8	218.4	192.8	1381	748.4	609
Get4	67604	Golgi to ER traffic protein 4 homolog	2.42554	3.3E-16	5.2E-15	-1.3805	0.00279	0.04361	-1.466	0.00039	0.00823	862.8	637.4	593.2	3670	2205	1944
Mrps2	118451	Putative uncharacterized protein	1.70484	8.3E-08	6.7E-07	-1.528	1.9E-05	0.00072	-1.7025	8.5E-08	6E-06	1596	850.4	774.2	4783	2601	2193
Zfp346	26919	Zinc finger protein 346	1.6224	0.00012	0.0007	-1.4736	0.0019	0.03219	-1.5603	0.00038	0.00794	299.4	115.4	132	853.8	480.6	428.2
Hmg20a	66867	High mobility group protein 20A	1.59489	4.4E-05	0.00027	-1.4223	0.00195	0.03285	-1.5051	0.00033	0.00707	648	234.4	247.2	1811	1060	940.6
Nfkbia	18035	NF-kappa-B inhibitor alpha	1.79497	0.00358	0.0168	-2.2702	4.4E-05	0.00147	-1.88	0.00166	0.02584	820.8	548.8	627.2	2603	949.4	1062
Arfrp1	76688	ADP-ribosylation factor-related protein 1	1.38015	0.006	0.02708	-1.8467	1.6E-07	1.1E-05	-1.7378	2.4E-06	0.00011	716.8	277	288.4	1736	781.4	781
Srsf9	108014	Serine/arginine-rich-splicing factor 9	1.91617	3.1E-10	3.1E-09	-1.5893	6.6E-06	0.00029	-1.5791	9E-06	0.00035	855.2	496.4	518.2	2883	1509	1425
Cpsf7	269061	Cleavage and polyadenylation specificity factor subunit 7	1.42798	0.00088	0.00449	-1.5541	3.7E-05	0.00127	-1.5524	3.9E-05	0.00121	1465	468	494.8	3688	1970	1854
Cnih4	98417	Cornichon homolog 4 (Drosophila), isoform CRA_a	2.32745	1.9E-12	2.3E-11	-1.5535	0.00021	0.00554	-1.623	4.8E-05	0.00142	450.4	356.2	342.8	1849	990.2	891
Papd4	100715	Poly(A) RNA polymerase GLD2	1.47254	0.00055	0.00293	-1.6356	1.1E-05	0.00044	-1.8342	6.1E-08	4.4E-06	807.2	312.2	350.2	2092	1059	889.6
Tob2	57259	Protein Tob2	1.5215	0.00086	0.00442	-1.4882	0.00157	0.02768	-1.7179	1.7E-05	0.00061	1411	507.2	556.8	3747	2088	1728
Reps1	19707	RalBP1-associated Eps domain-containing protein 1	2.20951	1.6E-12	2E-11	-1.4069	0.00218	0.03592	-1.3824	0.00367	0.04818	594.8	360.6	314.8	2322	1369	1316
Alkbh2	231642	Alpha-ketoglutarate-dependent dioxygenase alkB homolog 2	1.6461	0.00434	0.0201	-1.6954	0.00235	0.038	-1.9368	0.00015	0.00362	139.4	67.4	78.6	405.3	198.6	163.2
Trmt112	67674	Uncharacterized protein	1.75475	1.4E-07	1.1E-06	-1.4787	0.00023	0.00585	-1.6435	2.9E-06	0.00013	718.4	297	375.6	2212	1244	1062
Gtf3c5	70239	General transcription factor 3C polypeptide 5	1.59229	0.00137	0.00684	-1.5919	0.00132	0.02429	-1.5686	0.00189	0.02853	370.6	139.4	158.2	1038	541.8	521.4
Bak1	12018	Bcl-2 homologous antagonist/killer	1.67599	1E-05	6.7E-05	-1.43	0.00208	0.03453	-1.6055	4.8E-05	0.00142	448	169.4	194.8	1314	764.4	649.6
Ppp1r18	76448	Phostensin	1.78318	0.00057	0.00302	-1.8308	0.00028	0.00693	-1.7006	0.00143	0.02297	125.2	86.8	56.2	391.3	177.8	182
Cox17	12856	Cytochrome c oxidase, subunit XVII assembly protein homolog (Ye	1.95737	3.3E-06	2.3E-05	-1.6515	0.0005	0.01116	-1.8829	1.1E-05	0.00043	988	635.2	625	3397	1708	1409
Rtf1	76246	RNA polymerase-associated protein RTF1 homolog	1.4066	0.00289	0.01377	-1.7467	1.1E-06	5.9E-05	-1.6377	1.6E-05	0.00058	1061	375.4	357	2618	1245	1225
Tmem161a	234371	Transmembrane protein 161A	2.20829	1.5E-14	2.1E-13	-1.3682	0.00215	0.03549	-1.4769	0.00014	0.00341	665.8	371.6	368.2	2593	1571	1373
Bcas2	68183	Pre-mRNA-splicing factor SPF27	2.66769	0	0	-1.4489	0.00055	0.01211	-1.4539	0.00049	0.00983	853.6	671.6	633.4	4007	2299	2161
Mpzl2	14012	Myelin protein zero-like protein 2	1.73924	1.3E-06	9.7E-06	-1.4902	0.00048	0.01087	-1.585	5.6E-05	0.00163	1663	728	679.8	5073	2828	2499
Edc3	353190	Putative uncharacterized protein	1.65844	5.7E-05	0.00034	-1.4968	0.00128	0.02371	-1.6129	0.00014	0.00341	819	292.6	356.8	2389	1326	1153

S4 Table. Continue...

Name	NCBI gene ID	Database object name	NT vs NL			MT vs NT			LT vs NT			Mean					
			Fold Change	P-Value	FDR P-Value	Fold Change	P-Value	FDR P-Value	Fold Change	P-Value	FDR P-Value	NL	ML	LL	NT	MT	LT
Rnf220	66743	E3 ubiquitin-protein ligase Rnf220	2.31399	1.1E-16	1.8E-15	-1.341	0.00318	0.04823	-1.4045	0.00065	0.01222	635	367	332	2579	1599	1448
Utp23	78581	rRNA-processing protein UTP23 homolog	1.9538	5.5E-06	3.7E-05	-1.7693	9.3E-05	0.00277	-1.7103	0.00024	0.00543	190.6	116.8	122	658.5	310	302.8
Snrpb2	20639	U2 small nuclear ribonucleoprotein B''	3.28254	0	0	-1.4396	0.00125	0.02328	-1.4177	0.00201	0.02998	475.4	481.6	438.2	2743	1587	1512
Cdk9	107951	Cyclin-dependent kinase 9	1.55786	8.8E-06	5.8E-05	-1.7535	1.7E-08	1.4E-06	-1.7695	1E-08	9.1E-07	1252	570	502.2	3434	1623	1500
Glrx5	73046	Putative uncharacterized protein	2.17092	2.3E-08	2E-07	-1.5529	0.00149	0.02667	-1.8065	2E-05	0.00069	2225	1542	1453	8486	4542	3655
Egln3	112407	Egl nine homolog 3	1.71928	0.00054	0.00287	-1.803	0.00016	0.00446	-1.701	0.00068	0.01281	621.2	243	352.2	1881	863.6	872.6
Zfp36l2	12193	Zinc finger protein 36, C3H1 type-like 2	1.67473	6E-05	0.00036	-1.5902	0.0003	0.00732	-1.612	0.0002	0.00465	1696	653	727.2	5057	2646	2460
Dmwd	13401	Dystrophia myotonica WD repeat-containing protein	1.51407	0.0007	0.00365	-1.5935	0.00013	0.00378	-1.7762	2.6E-06	0.00012	674.6	243.6	246.2	1791	933	797
Taf11	68776	Transcription initiation factor TFIID subunit 11	1.66326	0.00041	0.00223	-1.7844	5.5E-05	0.00178	-1.9526	3.3E-06	0.00015	462.2	216.6	238.8	1347	627.2	540.4
Map4	17758	Microtubule-associated protein	2.1093	5.6E-10	5.6E-09	-1.5189	0.0005	0.01124	-1.5835	0.00013	0.00328	1901	1148	953	7071	3858	3467
Med25	75613	Mediator of RNA polymerase II transcription subunit 25	1.90463	4.5E-10	4.5E-09	-1.3565	0.00307	0.04697	-1.4876	0.00012	0.00297	1425	525.4	545.2	4763	2914	2524
Ddx47	67755	Probable ATP-dependent RNA helicase DDX47	2.1724	7.5E-14	1E-12	-1.4359	0.00046	0.01042	-1.4817	0.00014	0.00349	1154	542	626	4410	2553	2314
Snrnp35	76167	U11/U12 small nuclear ribonucleoprotein 35 kDa protein	1.72126	0.00222	0.01071	-1.8824	0.00034	0.00815	-1.6899	0.00297	0.04083	174.4	80.2	92	530.3	234.2	243.6
Il2rg	16186	Cytokine receptor common subunit gamma	2.16117	0.00084	0.00431	-2.1533	0.00074	0.01538	-1.9351	0.00367	0.04819	42	43.8	23.8	158.3	61	65.2
AU019823	270156	Protein AU019823	1.87049	6E-08	5E-07	-1.6724	7.8E-06	0.00033	-1.6226	2.6E-05	0.00086	648.6	304.2	322.6	2142	1061	1025
Emd	13726	Emerin	1.60115	2.3E-05	0.00014	-1.761	3.2E-07	2E-05	-2.0268	2E-10	2.6E-08	530	251.4	231.6	1489	701.8	576.4
Prune	229589	Protein prune homolog	2.48269	2.8E-15	4.1E-14	-1.4114	0.00257	0.04098	-1.5247	0.00023	0.00519	657	540	299.2	2866	1690	1483
Bloc1s2	73689	Biogenesis of lysosome-related organelles complex 1 subunit 2	2.31465	7.2E-08	5.9E-07	-1.67	0.00094	0.01856	-1.8396	8.6E-05	0.00232	437.2	306	331.2	1784	888.4	759.4
Ccdc59	52713	Thyroid transcription factor 1-associated protein 26	1.95923	5.3E-09	4.9E-08	-1.636	1.7E-05	0.00065	-1.6973	3.9E-06	0.00017	538	287.2	287	1857	944	847.6
Zrsr2	22184	U2 small nuclear ribonucleoprotein auxiliary factor 35 kDa subunit	1.66645	9.1E-05	0.00054	-1.892	9.5E-07	5.2E-05	-1.6376	0.00015	0.00363	394.4	169.2	178.2	1156	508.4	545.2
Polr3c	74414	Polymerase (RNA) III (DNA directed) polypeptide C	1.88595	3.5E-08	3E-07	-1.4503	0.00118	0.02213	-1.5653	9.4E-05	0.00248	861.2	360.8	345.4	2860	1638	1398
Pde6d	18582	Retinal rod rhodopsin-sensitive cGMP 3',5'-cyclic phosphodiestera	1.5936	0.00664	0.02973	-2.1754	5.8E-06	0.00026	-1.9683	7.8E-05	0.00214	171.8	89.6	83.2	479	183.4	190.4
Mrps36	66128	28S ribosomal protein S36, mitochondrial	2.53079	4.1E-12	4.8E-11	-1.5158	0.00174	0.02994	-1.6327	0.00023	0.00519	416.8	313.2	281.6	1857	1019	900
Ndor1	78797	NADPH-dependent diflavin oxidoreductase 1	1.59127	1.5E-05	9.5E-05	-1.5916	1.4E-05	0.00053	-1.6469	3.1E-06	0.00014	934.6	306.6	331.2	2616	1366	1261
Smco4	170748	Single-pass membrane and coiled-coil domain-containing protein	1.77918	1.5E-05	9.7E-05	-1.8918	1.5E-06	7.7E-05	-2.2227	1.9E-09	2E-07	267.8	187.8	131	835.5	367.6	297.6
Polr3gl	69870	DNA-directed RNA polymerase III subunit RPC7-like	2.15199	3E-07	2.3E-06	-1.6919	0.00039	0.00914	-1.8495	3.5E-05	0.0011	262.8	182.6	162.6	992.3	486.8	410.4
Srrt	83701	Serrate RNA effector molecule homolog	1.94127	6.8E-09	6.1E-08	-1.4325	0.00166	0.0289	-1.4445	0.00129	0.02123	2682	960.2	1027	9163	5323	4950
Wdr83os	414077	Protein Asterix	1.35104	0.00902	0.03937	-1.9384	9.2E-09	8.2E-07	-2.2247	4.3E-12	7.8E-10	868.4	312.8	307.6	2057	882.4	728.6
Pigb	55981	GPI mannosyltransferase 3	1.75066	1.6E-06	1.2E-05	-1.4174	0.00266	0.04209	-1.779	7.5E-07	4.1E-05	539.8	192.8	206.6	1659	971.6	726.6
Srebf2	20788	Sterol regulatory element-binding protein 2	1.61843	6.7E-05	0.0004	-1.5361	0.00037	0.00878	-2.0567	2.4E-09	2.4E-07	2159	957.8	725.6	6099	3285	2339
Mrpl47	74600	39S ribosomal protein L47, mitochondrial	2.84599	0	0	-1.4291	0.0013	0.02398	-1.5754	4.3E-05	0.00131	588.4	423.8	437.2	2954	1718	1462
Rbms2	56516	RNA-binding motif, single-stranded-interacting protein 2	1.46298	0.00583	0.02639	-1.7736	3.2E-05	0.00112	-2.0177	3.7E-07	2.2E-05	688.6	255.4	231.4	1769	828	681.4
Cdc42ep1	104445	Cdc42 effector protein 1	1.67693	5E-06	3.4E-05	-1.6704	5.6E-06	0.00025	-1.7105	2E-06	9.8E-05	1122	425.2	436.2	3291	1639	1529
Pick1	18693	PRKCA-binding protein	2.02353	1.9E-10	1.9E-09	-1.5219	0.00014	0.0038	-1.5804	3.2E-05	0.00103	746.8	343.8	338.2	2656	1449	1322
Gtf2f2	68705	General transcription factor IIF subunit 2	3.36845	0	0	-1.4008	0.00187	0.03175	-1.5088	0.00015	0.00365	342.2	296.2	305.8	2029	1205	1040
Atf1	11908	Cyclic AMP-dependent transcription factor ATF-1	1.42594	0.00085	0.00434	-1.9739	1.6E-10	2E-08	-2.0085	5.5E-11	8.1E-09	1070	372	405.8	2682	1130	1027
Sarnp	66118	SAP domain-containing ribonucleoprotein	2.41339	2.3E-11	2.6E-10	-1.729	3.1E-05	0.00108	-1.7418	2.4E-05	0.00081	1012	672.8	764	4284	2064	1857
Elp6	72341	Elongator complex protein 6	2.2189	7.1E-09	6.4E-08	-1.5103	0.00232	0.03772	-1.6581	0.0002	0.00459	171.2	85.6	101	668.3	367.8	319.8
Prpf19	28000	Pre-mRNA-processing factor 19	2.03613	9.8E-06	6.5E-05	-1.6189	0.00273	0.04281	-1.6844	0.00118	0.01987	4170	2410	1890	14944	7666	6930
Lsm14a	75547	Protein LSM14 homolog A	1.85875	1.3E-07	1E-06	-1.6117	4.6E-05	0.00152	-1.6772	1E-05	0.00039	1284	541.8	533.4	4192	2162	1931
Phpt1	75454	14 kDa phosphohistidine phosphatase	2.58685	1.3E-10	1.3E-09	-1.6571	0.00058	0.01259	-1.573	0.00203	0.03029	367.4	261.8	241.8	1662	833.2	845.8
Hnrnpdl	50926	Heterogeneous nuclear ribonucleoprotein D-like	2.18896	4E-10	4E-09	-1.5073	0.00104	0.01992	-1.4467	0.00316	0.04293	2854	1251	1253	10954	6044	5852
Cops7a	26894	COP9 signalosome complex subunit 7a	1.78073	2.6E-08	2.2E-07	-1.5481	2.4E-05	0.00086	-1.9291	2.2E-10	2.8E-08	1853	733.6	821.2	5790	3102	2381
E130309D02Rik	231868	Putative uncharacterized protein	2.19005	1.2E-12	1.5E-11	-1.4732	0.0004	0.00927	-1.6106	1.4E-05	0.0005	576.8	271.4	287.8	2221	1256	1091
Srsf11	69207	Sfrs11 protein	1.54493	0.00067	0.00349	-1.6927	3.8E-05	0.0013	-1.7451	1.3E-05	0.00049	3751	1107	1181	10224	5015	4531
Mmachc	67096	Methylmalonic aciduria and homocystinuria type C protein homol	2.55375	0	0	-1.4693	0.00034	0.00812	-1.6008	1.2E-05	0.00045	623	364	394.8	2796	1583	1353
Fcer1g	14127	High affinity immunoglobulin epsilon receptor subunit gamma	3.4658	2.2E-14	3.1E-13	-1.6382	0.00194	0.03282	-1.5927	0.0035	0.04644	122.4	154.2	99.8	741.3	377.2	376.8
Nol12	97961	Nucleolar protein 12	2.10734	7.2E-08	5.9E-07	-1.5933	0.00071	0.01479	-1.6377	0.00034	0.00727	414.4	178.6	215.4	1539	803.4	727.4
Lmf2	105847	Lipase maturation factor 2	1.93391	1.1E-09	1.1E-08	-1.5089	0.00013	0.00377	-1.5205	0.0001	0.00265	861.2	312	293.4	2921	1607	1509
Zfp593	68040	Putative uncharacterized protein	2.63541	1.5E-14	2.1E-13	-1.569	0.00027	0.0067	-1.5746	0.00024	0.00553	200.2	124.8	134.8	928.5	492.2	461.6
Pfdn6	14976	Prefoldin subunit 6	2.66226	0	0	-1.4352	0.00114	0.02162	-1.5155	0.00018	0.00435	764.2	376.4	500.4	3580	2074	1839
Ccdc85b	240514	Coiled-coil domain-containing protein 85B	1.68357	0.00285	0.0136	-1.94	0.00013	0.00374	-1.9966	7E-05	0.00196	101.8	40.8	51	301	129	118.6
Rhob	11852	Rho-related GTP-binding protein RhoB	2.45982	5.3E-11	5.7E-10	-1.6887	0.00013	0.0036	-1.524	0.00205	0.03047	778.8	482.2	456.4	3378	1662	1761
Zcchc8	70650	Putative uncharacterized protein	2.50242	0	0	-1.3763	0.00103	0.01989	-1.4628	9.4E-05	0.0025	801.2	358.4	383	3528	2130	1888
Rexo1	66932	Rexo1 protein	1.88705	7.7E-09	6.9E-08	-1.4455	0.00078	0.01595	-1.5468	7E-05	0.00196	1439	447.8	440.2	4764	2736	2428
Setd7	73251	Histone-lysine N-methyltransferase SETD7	2.85744	0	0	-1.5217	0.00061	0.0132	-1.6531	4.2E-05	0.00127	404.8	380.4	218.8	2060	1119	966.8
Zfp326	54367	DBIRD complex subunit ZNF326	1.67681	1.5E-05	1E-04	-1.7007	8.4E-06	0.00035	-1.717	5.8E-06	0.00024	625	209.2	212.8	1842	900.6	826
Camsap3	69697	Marshalin-Ld transcript variant 1	1.80208	1.4E-05	8.9E-05	-1.4894	0.00318	0.04818	-1.6716	0.00014	0.00354	809	299.6	218.6	2552	1422	1213
Sart3	53890	Squamous cell carcinoma antigen recognized by T-cells 3	2.48874	1.1E-16	1.8E-15	-1.4044	0.00184	0.03143	-1.4126	0.00154	0.02447	713.8	321.8	306	3113	1844	1725
Tomm6	66119	Mitochondrial import receptor subunit TOM6 homolog	1.63592	7.2E-05	0.00043	-1.7075	1.6E-05	0.00059	-1.9325	1.1E-07	7.3E-06	1538	539.2	555.8	4415	2144	1767
Mettl1	17299	tRNA (guanine-N(7)-)-methyltransferase	2.40978	9.1E-15	1.3E-13	-1.5159	0.00022	0.00561	-1.6016	2.9E-05	0.00094	517.4	257.8	275	2191	1201	1069
1110004E09Rik	68001	Protein 1110004E09Rik	2.24599	9.9E-11	1E-09	-1.6967	2.1E-05	0.00077	-1.7959	2.5E-06	0.00012	460.2	248.4	284.2	1820	892.2	787.2
Nus1	52014	Dehydrodolichyl diphosphate syntase complex subunit Nus1	1.97324	1.9E-08	1.6E-07	-1.6056	8.8E-05	0.00263	-1.814	8.1E-07	4.4E-05	2764	1175	1227	9567	4952	4083
Pkia	18767	cAMP-dependent protein kinase inhibitor alpha	3.65339	2.5E-07	2E-06	-2.2334	0.00105	0.02018	-2.2006	0.00132	0.02156	39	59.2	53.8	247.8	92.8	87.2
Gsk3a	606496	Glycogen synthase kinase-3 alpha	1.34289	0.00428	0.01986	-2.1558	1E-13	2.2E-11	-2.207	1.8E-14	4.6E-12	1745	562.8	534.4	4116	1588	1478
Npepps	19155	Puromycin-sensitive aminopeptidase	2.03563	1.6E-09	1.5E-08	-1.5646	0.00014	0.00385	-1.6081	5.2E-05	0.00154	1373	566.6	512.6	4910	2611	2388
Slc3a2	17254	4F2 cell-surface antigen heavy chain	2.21383	6.7E-13	8.5E-12	-1.5775	3.6E-05	0.00123	-1.6185	1.3E-05	0.00048	1587	770.6	713.6	6157	3239	2973
Eif3j2	1E+08	Eukaryotic translation initiation factor 3 subunit J-B	2.14163	3.6E-10	3.6E-09	-1.7008	1.2E-05	0.00047	-1.6536	3.4E-05	0.00107	2744	1203	1444	10379	5073	4859
Bcl7a	77045	B-cell CLL/lymphoma 7 protein family member A	2.05347	6.7E-11	7.1E-10	-1.389	0.00268	0.04224	-1.5435	7.5E-05	0.00207	642.8	178	218.4	2312	1385	1180
Srsf4	57317	Serine/arginine-rich-splicing factor 4	1.4683	0.00042	0.00227	-1.9142	2.4E-09	2.5E-07	-1.9999	2E-10	2.6E-08	1384	420.2	437	3573	1550	1380
Tdg	21665	G/T mismatch-specific thymine DNA glycosylase	2.53262	0	0	-1.4179	0.00084	0.01691	-1.4581	0.00031	0.00676	937.8	381.2	440.4	4176	2446	2211
Gps2	56310	Protein Gps2	2.15314	7E-11	7.4E-10	-1.7773	8.2E-07	4.6E-05	-1.5363	0.00023	0.00526	397.8	180.8	180.8	1504	702.8	763.4
Ap1m1	11767	AP-1 complex subunit mu-1	2.10264	7.1E-13	9E-12	-1.5156	5.5E-05	0.00176	-1.7336	9.7E-08	6.7E-06	1017	433.4	401.8	3743	2055	1681
Hdac1	433759	Histone deacetylase 1	2.77181	0	0	-1.4785	0.00117	0.02198	-1.4374	0.0026	0.03676	731	388.2	361.2	3561	2005	1910
Pinx1	72400	PIN2/TERF1-interacting telomerase inhibitor 1	1.67153	0.00552	0.02508	-1.9522	0.00029	0.00706	-1.7976	0.00148	0.02358	165.8	52.4	65.4	491	209.6	210.2
Mrpl37	56280	39S ribosomal protein L37, mitochondrial	1.80517	4.7E-09	4.3E-08	-1.6584	4.9E-07	3E-05	-1.9581	2.5E-11	3.9E-09	1479	534.6	589.6	4698	2354	1887
Ppp5c	19060	Serine/threonine-protein phosphatase	2.62062	0	0	-1.3922	0.00266	0.04207	-1.6075	1.6E-05	0.00059	1560	800.2	726.2	7167	4280	3506
Scoc	56367	Short coiled-coil protein	2.45804	3.3E-14	4.6E-13	-1.578	0.00011	0.00312	-2.0968	3.5E-10	4.3E-08	561.2	370.6	345.6	2430	1282	899.6
Slc35c2	228875	Solute carrier family 35 member C2	2.01259	3.3E-11	3.6E-10	-1.6072	6.2E-06	0.00027	-1.7384	1.4E-07	9.5E-06	969.8	387.2	385.6	3412	1764	1564
Apobec3	80287	Apobec3	3.15403	5.3E-10	5.2E-09	-1.7254	0.00243	0.03903	-1.8981	0.00038	0.00808	68.6	87.2	41	379.3	183.4	157.8
Ube2e1	22194	Ubiquitin-conjugating enzyme E2 E1	2.23892	1.3E-13	1.7E-12	-1.8482	1.5E-08	1.3E-06	-1.8504	1.4E-08	1.2E-06	1224	649.4	738.4	4815	2164	2014
Ncoa5	228869	Nuclear receptor coactivator 5	1.81912	1.9E-06	1.3E-05	-1.618	0.00012	0.00345	-1.7211	1.5E-05	0.00053	843.4	269.4	272.4	2703	1390	1229
Zc3h3	223642	Zc3h3 protein	1.66009	0.00131	0.00656	-1.6044	0.0026	0.04127	-1.7626	0.00031	0.0068	279.4	75.8	74	817.3	422	365.4

S4 Table. Continue...

Name	NCBI gene ID	Database object name	NT vs NL			MT vs NT			LT vs NT			Mean					
			Fold Change	P-Value	FDR P-Value	Fold Change	P-Value	FDR P-Value	Fold Change	P-Value	FDR P-Value	NL	ML	LL	NT	MT	LT
Ube2v1	66589	Ubiquitin-conjugating enzyme E2 variant 1	1.56127	4E-05	0.00025	-1.976	3.3E-10	4.1E-08	-2.1442	2E-12	3.8E-10	2895	1089	960.8	7944	3339	2905
Eif4enif1	74203	Eukaryotic translation initiation factor 4E transporter	2.70636	0	0	-1.4197	0.0012	0.02245	-1.5081	0.00015	0.00362	868.6	405.6	415.6	4148	2426	2120
Dhps	330817	Deoxyhypusine synthase	3.11824	0	0	-1.4513	0.00315	0.04792	-1.6045	0.00018	0.00429	324.6	211.8	212	1783	1020	875.2
Scamp4	56214	Secretory carrier membrane protein 4, isoform CRA_e	1.45594	0.00129	0.00646	-2.0061	2.4E-09	2.5E-07	-2.3989	7.3E-14	1.7E-11	939	320.4	311.6	2391	990.6	792
Msn	17698	Moesin	3.18543	0	0	-2.0986	3.5E-09	3.5E-07	-1.5553	0.00043	0.00879	709.4	698	583.6	3946	1567	2013
Safb	224903	Scaffold attachment factor B1	1.54082	9.5E-05	0.00056	-1.785	1.7E-07	1.1E-05	-1.754	3.9E-07	2.3E-05	2360	608.2	549.2	6387	2973	2790
Utp3	65961	Utp3 protein	1.89934	8.9E-08	7.2E-07	-2.0532	1.9E-09	2E-07	-1.6839	1.3E-05	0.0005	1114	429.6	503.8	3722	1507	1661
Mterf1b	208595	Transcription termination factor 1b, mitochondrial	2.13818	0.00028	0.00155	-2.0191	0.00062	0.01332	-1.8781	0.00215	0.0317	43.8	22.8	25.6	165.3	68	68.6
Denr	68184	Density-regulated protein	2.54212	0	0	-1.743	5.1E-07	3E-05	-1.6332	9.2E-06	0.00036	1148	599.6	692	5118	2442	2398
Gnb1	14688	Guanine nucleotide-binding protein G(I)/G(S)/G(T) subunit beta-1	2.70432	1.1E-16	1.8E-15	-1.5274	0.00044	0.01001	-1.6028	9E-05	0.00241	2773	1518	1415	13208	7178	6466
Polr1d	20018	DNA-directed RNA polymerases I and III subunit RPAC2	3.68534	0	0	-1.5668	0.00095	0.01867	-1.6123	0.00044	0.00892	1490	1339	1216	9675	5127	4708
Mphosph8	75339	M-phase phosphoprotein 8	2.5461	2.2E-16	3.5E-15	-1.5462	0.00012	0.00333	-1.465	0.00073	0.01351	711.6	313.2	302.6	3198	1719	1661
Fam98a	72722	Protein FAM98A	1.505	0.00192	0.00936	-1.8427	3.5E-06	0.00016	-1.8392	3.7E-06	0.00016	1599	351	417.6	4238	1917	1781
Plk2	20620	Serine/threonine-protein kinase PLK2	1.93004	0.00392	0.0183	-3.1515	4.8E-07	2.9E-05	-2.9265	2.5E-06	0.00012	637.2	444.2	463.4	2180	575	562.8
Hsf1	15499	Heat shock transcription factor 1 gammabeta isoform	2.32168	1.9E-12	2.3E-11	-1.423	0.00297	0.04594	-1.5955	8.5E-05	0.0023	590.4	197	218.8	2407	1407	1183
Arhgap17	70497	Rho GTPase-activating protein 17	1.66155	0.00011	0.00062	-1.7838	9.3E-06	0.00039	-2.1168	1E-08	9.1E-07	528.8	198.6	151.8	1542	717	568.6
Poc1b	382406	Wdr51b protein	2.38689	8.2E-11	8.7E-10	-1.5223	0.00146	0.02623	-1.7768	1.4E-05	0.00052	232.4	96.4	117	969.3	528.6	426.2
Sec24b	99683	Protein Sec24b	1.83646	7E-06	4.7E-05	-1.652	0.0002	0.00528	-1.7694	2.4E-05	0.0008	1111	340.8	330.6	3609	1809	1601
Lgals3bp	19039	Galectin-3-binding protein	1.83786	4.6E-08	3.8E-07	-1.9001	7.8E-09	7E-07	-2.234	5.1E-13	1E-10	1951	964	749.2	6300	2752	2243
Snrnp70	73318	U1 small nuclear ribonucleoprotein polypeptide A, isoform CRA_a	1.54067	0.00092	0.00472	-1.8944	9.7E-07	5.3E-05	-1.7934	7.6E-06	0.00031	5285	1311	1258	14267	6268	6118
Taf3	209361	Transcription initiation factor TFIID subunit 3	2.48325	9.3E-10	9.1E-09	-1.5571	0.00256	0.04086	-1.5442	0.0031	0.04239	220.2	86.8	97.2	961.5	513.2	482.2
Tmem69	230657	Transmembrane protein 69	1.71557	1.1E-05	7.4E-05	-1.723	9E-06	0.00037	-2.1253	8.4E-10	9.4E-08	550.6	160	198.8	1666	804.4	607
Hnrnpa0	77134	Heterogeneous nuclear ribonucleoprotein A0	3.08624	0	0	-1.4222	0.00321	0.04849	-1.4844	0.00095	0.01663	1810	898.8	917.4	9807	5726	5201
Mdm2	17246	E3 ubiquitin-protein ligase Mdm2	2.38765	9.1E-10	8.9E-09	-2.2843	5.8E-09	5.5E-07	-1.859	1.2E-05	0.00046	1225	837	776.8	5208	1886	2171
Ddx39b	53817	Spliceosome RNA helicase Ddx39b	2.22971	1.6E-11	1.8E-10	-1.5197	0.00043	0.00987	-1.6896	1E-05	0.00039	2873	1065	1024	11307	6179	5286
Eef1d	66656	Elongation factor 1-delta	3.71285	0	0	-1.4794	0.00115	0.02173	-1.4825	0.00108	0.01852	2446	1662	1717	15962	8966	8449
Pvr	52118	Tumor-associated glycoprotein E4	2.53255	4.2E-09	3.9E-08	-1.8738	6.4E-05	0.00201	-1.7598	0.00032	0.00694	289.6	176.2	172	1306	577.4	582.8
Gatc	384281	Glutamyl-tRNA(Gln) amidotransferase subunit C, mitochondrial	1.74154	6.1E-08	5E-07	-1.8457	1.9E-09	2.1E-07	-2.1162	2.4E-13	5.1E-11	804.2	276.2	290	2464	1109	905.6
E2f3	13557	E2F transcription factor 3	3.03186	0	0	-1.4062	0.00266	0.04207	-1.5359	0.00016	0.00384	419.2	236.2	173.2	2231	1316	1144
Ccdc9	243846	Coiled-coil domain-containing protein 9	2.03434	5.1E-09	4.7E-08	-1.6824	1.7E-05	0.00064	-1.7224	7.1E-06	0.00029	624.6	220.2	212.8	2235	1102	1011
U2af2	22185	Splicing factor U2AF 65 kDa subunit	2.75164	0	0	-1.4512	0.00154	0.02728	-1.5721	0.00012	0.00304	2453	1000	1156	11842	6789	5912
Dffa	13347	DNA fragmentation factor subunit alpha	2.65929	0	0	-1.404	0.00152	0.02704	-1.6414	3.7E-06	0.00016	834	331.2	337.4	3885	2302	1832
Srp19	66384	Signal recognition particle 19 kDa protein	2.04038	2.2E-07	1.7E-06	-2.1486	2.6E-08	2.1E-06	-1.8216	1.3E-05	0.00047	872.8	389.2	391.4	3120	1210	1309
Jund	16478	Transcription factor jun-D	2.13882	6.3E-06	4.2E-05	-2.2717	1.1E-06	5.8E-05	-1.9377	8.4E-05	0.00228	2026	1200	1023	7693	2797	3113
Ppp1r15a	17872	Myeloid differentiation primary response gene 116	2.19222	1.6E-06	1.1E-05	-2.0342	1.3E-05	0.00051	-1.904	7.7E-05	0.00212	437.6	221.2	230.4	1696	686.8	682
Gltp	56356	Glycolipid transfer protein	2.78792	9.9E-08	8E-07	-1.8144	0.00167	0.02904	-1.7795	0.00238	0.03418	103.2	76.4	58	503	230.6	228.4
Minos1	433771	MICOS complex subunit Mic10	1.75477	0.0027	0.0129	-2.3066	8.2E-06	0.00035	-2.3761	3.9E-06	0.00017	1454	588.6	655.4	4487	1616	1425
Med4	67381	Mediator of RNA polymerase II transcription subunit 4	3.31351	0	0	-1.5047	0.00215	0.03555	-1.4993	0.00237	0.03407	366	196.6	216	2135	1180	1092
Maz	17188	Myc-associated zinc finger protein	1.83278	9.8E-08	7.9E-07	-1.6429	1.2E-05	0.00048	-1.795	2.6E-07	1.6E-05	2709	720.8	721	8700	4405	3832
Icam1	15894	Intercellular adhesion molecule 1	3.29112	0	0	-1.7479	5.1E-06	0.00023	-1.5119	0.00073	0.01351	446.2	394.2	213.4	2598	1235	1355
Hnrnpc	15381	Putative uncharacterized protein	2.52853	6.4E-13	8.1E-12	-1.7123	3E-05	0.00105	-1.7232	2.4E-05	0.00081	3704	1690	1969	16462	7999	7504
Pole3	59001	DNA polymerase epsilon subunit 3	3.61062	0	0	-1.5893	9.7E-06	0.0004	-1.6146	4.8E-06	0.00021	651.4	474.8	484.4	4123	2160	1989
Surf2	20931	Surfeit locus protein 2	3.01357	0	0	-1.5995	4.9E-05	0.00161	-1.5584	0.00013	0.0032	484.8	277.4	246.2	2578	1340	1270
Llph	66225	Protein LLP homolog	3.28409	0	0	-1.6512	0.00013	0.00376	-1.5835	0.00046	0.00932	782.2	491.6	531.4	4528	2281	2188
Erf	13875	ETS domain-containing transcription factor ERF	2.62459	3.2E-12	3.8E-11	-1.6651	0.00022	0.00563	-1.5416	0.0017	0.02625	790	335.6	358.2	3617	1807	1855
Kif3a	16568	Kinesin-like protein	2.45815	8E-08	6.5E-07	-1.9722	4.1E-05	0.00138	-1.6603	0.00217	0.0319	140.8	81	64	606.8	255.2	280.2
Utp11l	67205	Probable U3 small nucleolar RNA-associated protein 11	3.30791	0	0	-1.4434	0.00089	0.01774	-1.5033	0.00022	0.00515	754.6	380.6	374.6	4404	2535	2247
Nudt16l1	66911	Protein syndesmos	2.64942	1.8E-15	2.7E-14	-1.6256	6.4E-05	0.00202	-1.8934	1.6E-07	1E-05	520.2	262	298	2400	1227	996.6
Zfp706	68036	Zinc finger protein 706, isoform CRA_a	2.3277	2.1E-11	2.3E-10	-1.8141	2.3E-06	0.00011	-2.0548	1.1E-08	9.9E-07	4210	2076	2330	17335	7938	6626
Zdhhc24	70605	Probable palmitoyltransferase ZDHHC24	2.36629	1.2E-09	1.2E-08	-1.7228	1E-04	0.00295	-1.7322	8.7E-05	0.00233	183.2	77.6	81	763.8	368	347.6
Pgd	110208	6-phosphogluconate dehydrogenase, decarboxylating	2.83188	3.3E-16	5.2E-15	-1.637	0.00011	0.00314	-2.1552	1.6E-09	1.7E-07	1473	1579	451.6	7304	3711	2658
Mllt11	56772	Protein AF1q	2.68812	3.5E-07	2.7E-06	-1.7922	0.00208	0.0346	-1.8827	0.00087	0.01555	62.8	37.6	37.8	296.8	137.8	123
Zfp692	103836	Zinc finger protein 692	1.88012	2.3E-05	0.00015	-1.7825	9.9E-05	0.00294	-1.8488	3.6E-05	0.00112	361.4	118.2	99.8	1195	556.8	509.4
Mrps6	121022	28S ribosomal protein S6, mitochondrial	2.04475	8.2E-06	5.5E-05	-2.0071	1.3E-05	0.00051	-2.5233	7.7E-09	7.1E-07	363.4	196.6	170.2	1308	542.2	411
Dtymk	21915	Thymidylate kinase	3.6474	0	0	-1.4238	0.00233	0.03778	-1.615	3.7E-05	0.00114	624.8	332.4	407	4017	2345	1959
Sf1	22668	Splicing factor 1	1.43746	0.0023	0.01107	-2.058	1.3E-09	1.5E-07	-2.1701	7.6E-11	1.1E-08	4230	885	995.8	10720	4321	3852
Eid1	58521	Putative uncharacterized protein	3.16365	0	0	-1.4967	0.00036	0.00853	-1.9604	2.8E-09	2.8E-07	458	272	309	2530	1404	1001
Ldb1	16825	LIM domain-binding protein 1	2.54588	0	0	-1.5842	1.5E-05	0.00056	-1.6995	6E-07	3.3E-05	1160	470	469.2	5194	2719	2384
Cdc37	12539	Hsp90 co-chaperone Cdc37	2.36701	1.1E-12	1.3E-11	-1.6849	1.6E-05	0.0006	-1.7595	2.9E-06	0.00013	2718	1039	1128	11295	5569	4936
Stra13	20892	Centromere protein X	2.12044	1.3E-09	1.3E-08	-1.834	8.7E-07	4.9E-05	-1.9876	2.7E-08	2.1E-06	498.6	180.8	218.6	1860	841.8	731.8
Psmg3	66506	Proteasome assembly chaperone 3	3.44497	0	0	-1.5026	0.00067	0.01411	-1.6592	2.4E-05	0.0008	406.8	231.6	248.2	2464	1364	1178
Ptpn23	104831	Tyrosine-protein phosphatase non-receptor type 23	1.6156	0.00097	0.00492	-1.9415	4.9E-06	0.00022	-2.1324	1.9E-07	1.2E-05	835	229	203.6	2384	1014	872
Gtf2e2	68153	General transcription factor IIE subunit 2	3.33103	0	0	-1.5314	0.0002	0.00535	-1.7181	2.5E-06	0.00012	459	265	282.2	2688	1460	1224
Casc3	192160	Protein CASC3	1.85524	1E-08	9.1E-08	-1.9048	2.2E-09	2.3E-07	-1.8276	2.1E-08	1.8E-06	1089	310.4	320.4	3556	1550	1512
Mapk3	26417	Mitogen-activated protein kinase	2.00698	6E-09	5.4E-08	-2.0329	2.9E-09	2.9E-07	-1.9147	5.4E-08	4E-06	875	380.8	291.6	3067	1255	1251
Ints9	210925	Integrator complex subunit 9	1.72075	9.5E-05	0.00056	-1.8407	1.1E-05	0.00043	-2.0066	5.3E-07	3E-05	377.4	94	97.4	1142	514.8	444.8
Ifi30	65972	Gamma-interferon-inducible lysosomal thiol reductase	5.21675	0	0	-1.4701	0.00066	0.01405	-1.4615	0.0008	0.01457	315	291	260.4	2882	1634	1540
Thy1	21838	Thy-1 membrane glycoprotein	2.45942	0.00182	0.00889	-2.8517	0.00025	0.00627	-2.3129	0.00323	0.04376	28	24.4	18.4	120.8	35.4	40.4
Nfkbib	18036	NF-kappa-B inhibitor beta	1.81824	5.4E-06	3.7E-05	-2.0161	9E-08	6.4E-06	-2.1113	1.3E-08	1.1E-06	590.4	200.2	197.6	1891	778.4	695
Psmd7	17463	Proteasome (Prosome, macropain) 26S subunit, non-ATPase, 7	2.35823	2.8E-10	2.8E-09	-1.8441	6.6E-06	0.00028	-1.7744	2.4E-05	0.00081	2474	998	1067	10279	4633	4397
Terf2	21750	Telomeric repeat-binding factor 2	2.3109	1E-12	1.3E-11	-1.8299	2.1E-07	1.4E-05	-1.8044	4.1E-07	2.4E-05	365.2	147.4	146.8	1482	673	640
Prpf38b	66921	Pre-mRNA-splicing factor 38B	1.77562	1.4E-06	1E-05	-2.1359	1.8E-10	2.4E-08	-1.875	1.3E-07	8.7E-06	2194	639.4	654	6851	2671	2788
Exosc1	66583	Exosome complex component CSL4	3.75325	0	0	-1.5124	0.00135	0.02464	-1.5143	0.00131	0.02141	321.8	162.8	196.4	2125	1169	1082
Ap1g2	11766	AP-1 complex subunit gamma-like 2	2.55374	1.8E-14	2.5E-13	-1.4934	0.00091	0.01813	-1.7867	1.7E-06	8.4E-05	375	147.8	113.8	1686	937	728.6
Tbc1d14	100855	TBC1 domain family member 14	2.40132	1E-11	1.2E-10	-1.7046	3.3E-05	0.00113	-1.8924	6.9E-07	3.8E-05	877.2	366.2	332.4	3703	1801	1510
Sox13	20668	Transcription factor SOX-13	2.11253	8.7E-07	6.4E-06	-1.6736	0.00066	0.01399	-1.9474	1.1E-05	0.00041	346.2	96.6	117.2	1292	641.4	525
Dot1l	208266	Protein Dot1l	1.6504	0.00024	0.00132	-1.6998	9.8E-05	0.00289	-1.9148	1.9E-06	9.1E-05	1738	269	270.2	5046	2461	2040
Ddx51	69663	ATP-dependent RNA helicase DDX51	2.79146	1.1E-16	1.8E-15	-1.6195	9E-05	0.00271	-1.742	6.7E-06	0.00027	499	203.8	223.8	2459	1262	1094
Hmgb3	15354	High mobility group protein B3	3.84807	0	0	-1.6113	5.7E-05	0.00182	-1.5858	0.0001	0.00264	474	306	298	3200	1654	1549
Ppm1g	14208	Protein phosphatase 1G	2.94977	0	0	-1.6281	0.00014	0.00383	-1.5405	0.00072	0.01338	1991	772.8	774.4	10348	5286	5084
Mta1	116870	Metastasis-associated protein MTA1 isoform 4	3.26864	0	0	-1.4851	8.6E-05	0.00258	-1.6958	1.6E-07	1E-05	1076	518.8	480.2	6179	3450	2853
1110002L01Rik	1E+08	Protein 1110002L01Rik	2.31527	1.5E-08	1.4E-07	-1.7638	0.00012	0.00343	-1.6838	0.00041	0.00853	343.6	94.6	117.2	1394	658.2	650.2
Ubqln2	54609	Ubiquilin-2	1.63692	0.00211	0.01022	-1.9053	5.7E-05	0.00182	-2.3166	1.6E-07	1E-05	1064	268	234.8	3055	1328	1045

S4 Table. Continue...

Name	NCBI gene ID	Database object name	NT vs NL			MT vs NT			LT vs NT			Mean					
			Fold Change	P-Value	FDR P-Value	Fold Change	P-Value	FDR P-Value	Fold Change	P-Value	FDR P-Value	NL	ML	LL	NT	MT	LT
Rtn3	20168	Putative uncharacterized protein	1.67044	7.8E-06	5.2E-05	-2.0963	1.1E-10	1.5E-08	-2.5081	1.1E-15	3.2E-13	4000	1189	1213	11730	4647	3669
Mdh2	17448	Malate dehydrogenase, mitochondrial	2.21216	6.7E-07	5E-06	-1.7311	0.00059	0.01281	-2.0861	4.2E-06	0.00018	6171	2375	2051	24030	11517	8966
2410002F23Rik	668661	Protein 2410002F23Rik	3.07265	0	0	-1.469	0.00138	0.02511	-1.6019	9E-05	0.0024	718	244.2	255.2	3903	2208	1919
Bax	12028	Apoptosis regulator BAX	3.23566	0	0	-1.7842	1.9E-07	1.2E-05	-1.6165	1.5E-05	0.00055	583.4	366.6	254	3327	1549	1615
Ppp2r3c	59032	Serine/threonine-protein phosphatase 2A regulatory subunit B'' su	4.56418	0	0	-1.7458	1.9E-06	9.6E-05	-1.6152	4.1E-05	0.00125	505.8	451.6	414.6	4063	1932	1961
Rnps1	19826	RNA/DNA-binding protein	4.92178	0	0	-1.4109	0.00235	0.038	-1.4929	0.0004	0.00834	828	514.4	521.6	7176	4223	3777
Fam206a	230234	Protein Simiate	2.35973	7.4E-11	7.9E-10	-1.5505	0.00074	0.0153	-1.9906	1.3E-07	9E-06	224.4	68.4	71.8	933	501.2	367.4
Smndc1	76479	Putative uncharacterized protein	2.16599	1.1E-11	1.2E-10	-1.9667	2.3E-09	2.4E-07	-2.0381	3.3E-10	4E-08	621	211.4	251.6	2366	999	912.8
Diexf	215193	Digestive organ expansion factor homolog	3.55922	0	0	-1.4838	0.0011	0.02094	-1.5161	0.00058	0.0112	420.8	151	181.8	2649	1482	1376
U2af1	108121	MCG14259, isoform CRA_b	2.97989	0	0	-1.6685	8.1E-06	0.00034	-1.8212	1.7E-07	1.1E-05	1660	832	766.8	8740	4353	3715
Sf3a1	67465	Putative uncharacterized protein	3.39633	0	0	-1.4647	0.00051	0.01138	-1.5875	2.6E-05	0.00086	1132	455.8	427.4	6765	3838	3337
Gkap1	56278	G kinase-anchoring protein 1	4.85371	0	0	-1.5934	6.3E-05	0.00198	-1.4283	0.00219	0.03211	414.4	301.2	264	3535	1842	1905
Kat2a	14534	Putative uncharacterized protein	3.30759	0	0	-1.4333	0.00068	0.01441	-1.5625	2.6E-05	0.00086	905	294.6	316	5262	3053	2646
Rhno1	72440	RAD9, HUS1, RAD1-interacting nuclear orphan protein 1	3.15536	0	0	-1.4919	0.00088	0.01766	-1.5967	0.0001	0.00267	332.2	129.4	102.2	1836	1023	892.4
Pmf1	67037	Polyamine-modulated factor 1	4.4017	0	0	-1.5097	0.00015	0.00412	-1.7566	2.2E-07	1.4E-05	344.4	302.6	199.8	2652	1458	1181
Nt5c3b	68106	7-methylguanosine phosphate-specific 5'-nucleotidase	3.79104	1.9E-12	2.3E-11	-1.7358	0.00307	0.04697	-1.7476	0.00274	0.03838	120.8	99.4	67.8	804	386.2	359.8
Bcl9	77578	B-cell CLL/lymphoma 9 protein	1.64479	0.00482	0.02215	-1.9849	0.0001	0.00296	-1.8502	0.00048	0.00966	395.6	66	60	1130	471.8	484.8
Ddx19a	13680	ATP-dependent RNA helicase DDX19A	2.46955	8.9E-14	1.2E-12	-1.6941	1.3E-05	0.00051	-1.9076	9.3E-08	6.5E-06	1143	377.4	430.2	4955	2428	2026
Prps1	19139	Putative uncharacterized protein	3.84096	0	0	-1.6166	1.7E-05	0.00063	-1.7777	2.5E-07	1.5E-05	1108	764.4	662.2	7492	3843	3258
Rere	68703	Arginine-glutamic acid dipeptide repeats protein	3.12757	0	0	-1.5125	0.00218	0.03591	-1.7059	7.7E-05	0.00212	1160	435	444	6392	3505	2922
Rnf126	70294	Putative uncharacterized protein	2.91101	0	0	-1.5526	6.5E-05	0.00203	-1.7358	5.6E-07	3.1E-05	814.2	306	274.2	4143	2219	1874
Ppig	228005	Peptidyl-prolyl cis-trans isomerase	2.24525	1.1E-12	1.3E-11	-2.1541	1.3E-11	2.1E-09	-1.839	7.6E-08	5.4E-06	1370	480.6	539.6	5421	2094	2244
Btg2	12227	Protein BTG2	4.28977	3.6E-09	3.3E-08	-3.1766	2.7E-06	0.00013	-2.3753	0.00044	0.00892	370.8	902.2	221.8	2829	734.2	926
Ywhah	22629	14-3-3 protein eta	2.71038	0	0	-1.9715	5.6E-09	5.2E-07	-1.8402	1.6E-07	1.1E-05	1531	836.4	625.8	7295	3083	3120
Ubap2l	74383	Putative uncharacterized protein	2.50108	3.1E-14	4.3E-13	-1.6119	7.4E-05	0.00228	-1.8037	9.9E-07	5.2E-05	2231	621.2	662.8	9800	5042	4239
Psmg2	107047	Proteasome assembly chaperone 2	3.27804	0	0	-1.606	1.1E-05	0.00044	-1.9576	4.8E-10	5.7E-08	611	301.6	359.2	3524	1822	1426
Csnk1e	27373	Casein kinase I isoform epsilon	2.98797	3.5E-14	4.8E-13	-1.8937	8.1E-06	0.00034	-1.9171	5.5E-06	0.00023	291.6	186.4	136	1529	668.8	630.8
0610010K14Rik	104457	Protein 0610010K14Rik	2.41918	1.2E-10	1.3E-09	-1.9386	1.1E-06	5.9E-05	-2.2899	1.2E-09	1.3E-07	226.6	102.4	105.6	961.8	412	327.4
E2f5	13559	Transcription factor E2F5	4.36182	0	0	-1.4828	0.00217	0.03567	-1.8127	3.8E-06	0.00017	264.8	164.6	187.4	2039	1145	884.8
Pigs	276846	Putative uncharacterized protein	2.54266	0	0	-1.7127	8E-07	4.5E-05	-2.1373	3.5E-12	6.4E-10	981.8	376.8	422.4	4394	2132	1624
Agpat1	55979	1-acyl-sn-glycerol-3-phosphate acyltransferase	2.46052	2E-12	2.4E-11	-1.656	7.2E-05	0.00223	-1.7641	8.1E-06	0.00032	492.2	130.8	133	2122	1066	946.6
Snrpe	20643	Small nuclear ribonucleoprotein E	5.18854	0	0	-1.7443	0.00071	0.01484	-1.8865	0.00011	0.0029	673.8	715.8	674.2	6156	2934	2581
Cfl1	12631	Cofilin-1	2.92256	1.4E-10	1.4E-09	-1.6966	0.00155	0.02749	-1.8783	0.00016	0.00389	5698	2485	2477	29270	14341	12186
Las1l	76130	Ribosomal biogenesis protein LAS1L	2.641	0	0	-1.7891	2.1E-07	1.4E-05	-1.6215	1.6E-05	0.00058	1437	438.6	425.4	6663	3096	3138
Mbd3	17192	Methyl-CpG-binding domain protein 3	1.98134	1.5E-10	1.6E-09	-1.9756	1.7E-10	2.2E-08	-2.1396	1E-12	2E-10	1744	520.6	476.8	6069	2553	2198
Psme3	19192	Proteasome activator complex subunit 3	2.0496	2.5E-10	2.5E-09	-2.0237	4.9E-10	5.9E-08	-2.1478	1.5E-11	2.6E-09	2739	835.8	886.6	9918	4075	3608
Pigl	327942	Pigl protein	2.49618	3.4E-12	4.1E-11	-1.7365	1.9E-05	0.00072	-2.0844	1.5E-08	1.3E-06	178.2	61.8	68.4	781.3	374	293.8
Prr12	233210	Protein Prr12	1.85844	0.00033	0.0018	-1.7265	0.00151	0.02694	-2.0468	3.2E-05	0.00103	553	94	88.4	1790	857.4	687.4
Eif5b	226982	Eukaryotic translation initiation factor 5B	1.69228	0.00076	0.00391	-2.3792	2.9E-08	2.3E-06	-1.9748	1.3E-05	0.00049	3688	735.8	783.2	10977	3831	4171
Dck	13178	Deoxycytidine kinase	5.73851	0	0	-1.4676	0.00156	0.02759	-1.6365	5E-05	0.00147	213.2	190.2	136.4	2144	1215	1042
Patl1	225929	Protein PAT1 homolog 1	1.98789	2.8E-08	2.4E-07	-1.9178	1.3E-07	9.1E-06	-2.2868	2.2E-11	3.6E-09	966.6	265.2	267.6	3373	1460	1165
Pgap3	320655	Post-GPI attachment to proteins factor 3	3.60661	2.9E-15	4.3E-14	-1.6271	0.00194	0.03274	-1.7373	0.00045	0.00913	80	34.6	41.4	507.5	259.4	229.8
Scaf4	224432	MKIAA1172 protein	1.87585	2E-07	1.6E-06	-1.896	1.2E-07	8.2E-06	-2.1161	5.7E-10	6.6E-08	918.2	181.4	193.2	3035	1328	1108
Dph6	66632	Diphthine--ammonia ligase	3.13972	0	0	-1.634	4.3E-05	0.00144	-1.85	3.1E-07	1.9E-05	290.6	117.6	116.4	1601	815.2	676.6
Cyb5r1	72017	NADH-cytochrome b5 reductase 1	3.84838	0	0	-1.5381	0.00029	0.00704	-1.7999	7.7E-07	4.1E-05	314	157.2	159.2	2136	1154	931
Prkab2	108097	5'-AMP-activated protein kinase subunit beta-2	3.04315	0	0	-1.6693	8.2E-05	0.00248	-1.8293	3.6E-06	0.00016	191.6	81.8	66.2	1022	507.6	437
Ddx18	66942	DEAD (Asp-Glu-Ala-Asp) box polypeptide 18	4.52076	0	0	-1.4286	0.00148	0.02653	-1.585	4.1E-05	0.00125	805.2	340	357.4	6425	3735	3160
Pfdn2	18637	Prefoldin subunit 2	2.10128	5.6E-08	4.6E-07	-2.4546	4.9E-11	7.1E-09	-2.3427	4.6E-10	5.4E-08	970.4	299.8	466.6	3591	1215	1148
Usp36	72344	Ubiquitin carboxyl-terminal hydrolase 36	3.07861	0	0	-1.5523	6.5E-05	0.00204	-1.6718	3.1E-06	0.00014	1075	237.8	346.2	5814	3102	2739
Nabp2	69917	SOSS complex subunit B1	1.7431	2.3E-07	1.8E-06	-2.5732	0	0	-2.6966	0	0	936.2	292.8	297	2869	925.6	829.4
Snrpa	53607	U1 small nuclear ribonucleoprotein A	2.30881	3.8E-12	4.5E-11	-1.8243	5.6E-07	3.3E-05	-1.9084	7.5E-08	5.4E-06	1084	260.2	307.8	4397	2003	1803
Evc	59056	Ellis-van Creveld syndrome protein homolog	2.95846	2.6E-06	1.8E-05	-3.2553	2.8E-07	1.7E-05	-2.2335	0.00045	0.00903	101.4	102.8	58.6	521	133.4	182
Brix1	67832	Ribosome biogenesis protein BRX1 homolog	3.4056	0	0	-1.7163	1.5E-05	0.00057	-1.7691	4.8E-06	0.00021	935.2	367	448.6	5633	2729	2431
Ino80e	233875	Protein Ino80e	3.48291	0	0	-1.6406	6.3E-05	0.00198	-1.7152	1.3E-05	0.00049	421.6	169.8	170.6	2582	1310	1168
Rpl35	66489	60S ribosomal protein L35	4.69777	6E-15	8.7E-14	-1.8258	0.00236	0.0381	-1.9071	0.00111	0.01888	1480	1351	1094	12247	5568	4992
9430015G10Rik	230996	Protein 9430015G10Rik	3.28665	0	0	-1.599	0.00043	0.00986	-1.8693	2.8E-06	0.00013	266.2	104.8	102.2	1538	800	643.6
Nop14	75416	Putative uncharacterized protein	4.01373	0	0	-1.5765	8.1E-05	0.00246	-1.5426	0.00017	0.00416	845.4	333.4	295	5987	3150	2941
Marcksl1	17357	MARCKS-related protein	5.84067	0	0	-1.4732	0.00093	0.01838	-1.7242	3.3E-06	0.00015	342	279.6	229	3481	1967	1595
Rnf44	105239	RING finger protein 44	1.60078	2.7E-05	0.00017	-2.2319	7.8E-13	1.6E-10	-2.6344	0	0	2178	421.2	461	6148	2283	1839
Pla2g12a	66350	Phospholipase A2, group XIIA	5.28052	0	0	-1.502	0.00046	0.01047	-1.5851	7.4E-05	0.00204	525	206	374.4	4897	2700	2421
Pnn	18949	Pinin	1.99821	8E-09	7.2E-08	-2.1882	6.5E-11	9.4E-09	-1.8489	3E-07	1.8E-05	2248	429.2	511	7920	3011	3251
Cnot3	232791	CCR4-NOT transcription complex subunit 3	1.46988	0.00129	0.00646	-2.3915	3.4E-13	6.9E-11	-2.6007	1.7E-15	4.6E-13	832.6	133	142	2151	744.8	647.2
Hira	15260	Protein HIRA	4.1871	0	0	-1.5226	0.00061	0.01315	-1.7387	6.6E-06	0.00027	587.4	267.8	270.6	4315	2354	1927
Nr2c2ap	75692	Nuclear receptor 2C2-associated protein	2.06441	6E-07	4.5E-06	-2.4224	1E-09	1.2E-07	-2.574	7.4E-11	1.1E-08	502	188.8	179.4	1817	623	540
Nol10	217431	Putative uncharacterized protein	3.1322	1.9E-13	2.5E-12	-1.6916	0.00065	0.01375	-1.7932	0.00015	0.0037	368.6	116.6	135.2	2042	1007	868.4
Lsm5	66373	U6 snRNA-associated Sm-like protein LSm5	5.86575	0	0	-1.8176	0.00051	0.01138	-1.9049	0.00018	0.00428	148.4	177.6	135.6	1521	697.4	628.4
Npm3	18150	Putative uncharacterized protein	8.17628	0	0	-1.4522	0.00144	0.02602	-1.5675	0.00012	0.00317	354	311.4	318.6	5112	2927	2574
Mef2d	17261	Myocyte-specific enhancer factor 2D	1.91162	4.5E-08	3.7E-07	-1.971	9.5E-09	8.5E-07	-2.2321	1.2E-11	2E-09	1478	327.4	262.6	4949	2082	1740
Samd10	229011	Samd10 protein	2.21306	2E-05	0.00013	-2.2605	9.9E-06	0.00041	-2.1963	2.1E-05	0.00071	87.4	30.8	28	340.8	125.2	122.8
Rad9a	19367	Cell cycle checkpoint control protein RAD9A	3.19368	4.9E-15	7.1E-14	-1.8404	2.9E-05	0.00104	-1.8969	1.2E-05	0.00045	178.4	93.6	59.8	995.3	449.6	419.2
Ccdc86	108673	Coiled-coil domain-containing protein 86	4.0057	0	0	-1.592	0.00018	0.00472	-1.8053	1.9E-06	9.3E-05	500.2	201.6	250.6	3541	1850	1554
G6pc3	68401	Glucose-6-phosphatase 3	2.57366	9.1E-12	1.1E-10	-1.8846	3.7E-06	0.00017	-2.1012	6.3E-08	4.6E-06	206.6	61.2	73.6	933.5	412.2	353
Tardbp	230908	TAR DNA-binding protein 43	2.38084	1.3E-08	1.2E-07	-2.0946	1.3E-06	6.7E-05	-2.0016	5.4E-06	0.00023	5431	1491	1713	22797	9055	8816
Gnai1	14677	Guanine nucleotide-binding protein G(i) subunit alpha-1	5.20877	1.1E-08	9.8E-08	-2.504	0.00098	0.01909	-2.5872	0.00066	0.01242	22.8	29.6	36.8	208.8	70	64.4
Prr3	75210	Proline-rich protein 3	2.38307	1.9E-11	2.1E-10	-1.7998	4.3E-06	0.0002	-2.0066	5.6E-08	4.1E-06	264.8	52.6	67.6	1109	512.6	435.2
Max	17187	Max protein	3.28773	0	0	-1.8326	5E-08	3.8E-06	-1.9478	2E-09	2.1E-07	678	301.6	294.8	3919	1781	1580
Hnrnpa3	229279	Heterogeneous nuclear ribonucleoprotein A3	1.92614	3.7E-06	2.6E-05	-2.445	2.8E-10	3.5E-08	-2.4008	6.4E-10	7.3E-08	5165	1173	1606	17446	5920	5567
Ccdc137	67291	Coiled-coil domain-containing protein 137	5.21634	0	0	-1.4434	0.00061	0.0131	-1.7055	6.3E-07	3.5E-05	486.6	209.8	237.8	4481	2578	2041
Proser1	212127	Putative uncharacterized protein	1.84515	0.00037	0.00202	-2.1385	9.7E-06	0.0004	-2.4211	2.8E-07	1.7E-05	423.8	78.8	89.4	1369	530.6	438.6
1700037H04Rik	67326	Protein 1700037H04Rik	4.77453	0	0	-1.6596	0.00074	0.01531	-1.7044	0.00039	0.00817	86.8	47	48.2	729	364	338.6
D2Wsu81e	227695	Putative methyltransferase C9orf114 homolog	5.97739	0	0	-1.483	0.00015	0.00403	-1.6177	3.6E-06	0.00016	590.8	300.4	322.2	6214	3478	3000
Ubald1	207740	UBA-like domain-containing protein 1	1.7863	1.1E-05	7.2E-05	-2.641	1.8E-13	3.8E-11	-2.6063	4E-13	8.5E-11	553.4	135.4	145.2	1740	544.4	521.8
Tmem41a	66664	Transmembrane protein 41A	8.71728	0	0	-1.4053	0.00221	0.03629	-1.6124	1.8E-05	0.00062	234.8	263	110.6	3616	2137	1764

S4 Table. Continue...

Name	NCBI gene ID	Database object name	NT vs NL			MT vs NT			LT vs NT			Mean					
			Fold Change	P-Value	FDR P-Value	Fold Change	P-Value	FDR P-Value	Fold Change	P-Value	FDR P-Value	NL	ML	LL	NT	MT	LT
Foxp4	74123	Forkhead box protein P4	2.03342	3.7E-07	2.9E-06	-2.0408	3.1E-07	1.9E-05	-2.1591	3.4E-08	2.7E-06	890	192	148.6	3169	1286	1165
Ppp2r2a	71978	Serine/threonine-protein phosphatase 2A 55 kDa regulatory subu	3.16507	8.2E-12	9.5E-11	-1.8656	0.00021	0.00541	-1.8514	0.00025	0.00561	734.4	258	248.2	4115	1829	1695
Tjap1	74094	Protein incorporated later into tight junctions	2.05968	1.5E-08	1.3E-07	-2.3756	9.6E-12	1.6E-09	-2.3594	1.5E-11	2.5E-09	315.6	86.2	90.4	1146	400.4	381.8
Cks1b	54124	Cyclin-dependent kinases regulatory subunit	7.2095	0	0	-1.4994	0.00287	0.04466	-1.5609	0.00105	0.01808	223.8	123.2	146	2811	1559	1412
Gga3	260302	ADP-ribosylation factor-binding protein GGA3	2.2708	1.3E-11	1.5E-10	-1.9878	1.2E-08	1.1E-06	-2.0605	2.1E-09	2.2E-07	649.4	135	145.8	2602	1088	1004
Pde4a	18577	Phosphodiesterase 4A, cAMP specific	2.83639	1.9E-08	1.6E-07	-1.8703	0.00066	0.01398	-2.2743	8.2E-06	0.00033	180	98	32.4	886.5	393.8	308
Gm14221	1.05E+08	Protein Gm14221	2.54489	0.00044	0.00238	-2.1516	0.00301	0.04629	-2.4336	0.00064	0.01219	21	10.4	5.6	93.75	36.2	29.6
Rogdi	66049	Protein rogdi homolog	2.18426	3.2E-10	3.2E-09	-2.4444	5.3E-13	1.1E-10	-2.5028	1.4E-13	3.1E-11	533.4	219.2	134.4	2047	692.8	645
Carm1	59035	Histone-arginine methyltransferase CARM1	4.15363	0	0	-1.5712	1.4E-05	0.00054	-1.9818	5.1E-11	7.6E-09	897.4	381.4	375.6	6553	3459	2595
Wbp11	60321	Phka1 protein	2.76648	0	0	-1.8651	1.6E-09	1.8E-07	-2.0518	3.7E-12	6.7E-10	1281	344.2	374	6244	2779	2376
E2f4	104394	Transcription factor E2F4	2.53941	1.1E-16	1.8E-15	-1.9703	9.5E-10	1.1E-07	-2.0752	4.7E-11	7.1E-09	787	195.2	211.6	3525	1487	1342
Wiz	22404	Protein Wiz	1.8083	3.1E-05	0.00019	-2.0745	2.8E-07	1.7E-05	-2.5211	7.9E-11	1.1E-08	858	129	132.4	2730	1090	857.2
Rrs1	59014	MKIAA0112 protein	4.40359	0	0	-1.594	0.0021	0.03487	-1.6921	0.00052	0.0103	734.6	205.2	287.8	5812	3033	2662
Bbc3	170770	Bcl-2 binding component 3	4.16541	3.6E-09	3.3E-08	-2.2737	0.00065	0.01382	-2.2062	0.00102	0.01769	305.4	311.8	172.8	2222	807.8	779.2
Crtap	56693	Cartilage-associated protein	4.9658	0	0	-1.6041	0.00013	0.0037	-1.8941	2.5E-07	1.5E-05	247	151.2	111.2	2145	1111	891.2
Noc4l	100608	Nucleolar complex associated 4 homolog (S. cerevisiae)	4.80088	0	0	-1.4776	0.00053	0.01164	-1.7905	2.4E-07	1.5E-05	555.6	181.4	203.2	4728	2657	2085
Cited2	17684	Cbp/p300-interacting transactivator 2	1.94901	5.6E-06	3.8E-05	-2.4546	9.8E-10	1.1E-07	-2.5536	1.8E-10	2.4E-08	881.2	175.6	241.2	3028	1018	923.8
Mid1	17318	E3 ubiquitin-protein ligase Midline-1	2.08075	0.00038	0.00207	-2.1205	0.00026	0.00661	-2.1896	0.00014	0.00353	692.6	144.2	105.6	2563	1004	901.2
Lrrc47	72946	Leucine rich repeat containing 47	2.90767	0	0	-1.9446	1E-09	1.1E-07	-2.0099	1.5E-10	2E-08	1055	305	322.6	5392	2302	2072
Ccnk	12454	Cyclin-K	1.73965	0.0007	0.00363	-2.3892	9.6E-08	6.8E-06	-2.8393	1.7E-10	2.3E-08	723.6	124.2	130.8	2205	764.4	599.8
Parvb	170736	Beta-parvin	2.96565	1.6E-08	1.4E-07	-1.9957	0.00028	0.00688	-3.4802	7.9E-11	1.1E-08	129	84.8	52.2	670.5	279.2	148.4
Fam122b	78755	Synoviocyte proliferation associated in collagen-induced arthritis	3.49296	0	0	-1.7039	0.00011	0.00328	-2.3111	1.6E-09	1.7E-07	135.2	54	50.2	827.5	403.2	280.2
Zmiz2	52915	Zinc finger MIZ domain-containing protein 2	1.50645	0.00158	0.00782	-2.3982	1.6E-11	2.5E-09	-3.3915	0	0	1369	176.2	185.4	3613	1249	844.4
Ung	22256	Uracil-DNA glycosylase	5.95018	0	0	-1.5104	0.00253	0.04039	-1.6195	0.00042	0.00859	332.8	80.4	130	3525	1939	1711
Dut	110074	Protein Dut	13.8397	0	0	-1.4537	0.00068	0.0143	-1.5053	0.0002	0.00475	269.6	222.6	222.8	6554	3750	3411
Slc35a4	67843	Probable UDP-sugar transporter protein SLC35A4	4.29275	0	0	-1.6614	3.8E-06	0.00018	-1.9265	2.5E-09	2.5E-07	592.2	199.4	242.2	4474	2238	1841
Hspbap1	66667	HSPB1-associated protein 1	11.5832	0	0	-1.513	0.00072	0.01493	-1.5362	0.00046	0.00919	131.2	130	61.8	2684	1474	1363
Aaas	223921	Aladin	6.12409	0	0	-1.5821	1.3E-05	0.00053	-1.8132	1.7E-08	1.5E-06	285.2	169.6	119.4	3077	1613	1326
Pcbp2	18521	Poly(RC) binding protein 2	2.32338	3.8E-06	2.6E-05	-2.1774	2E-05	0.00074	-2.4693	7.2E-07	3.9E-05	4722	1133	1125	19214	7315	6051
Fntb	110606	Protein farnesyltransferase subunit beta	3.86632	0	0	-1.7608	7.5E-05	0.00231	-1.9149	5.6E-06	0.00024	192.2	57.8	61.8	1305	615.8	538.4
Adprh	11544	[Protein ADP-ribosylarginine] hydrolase	4.25077	0	0	-1.8569	7.6E-07	4.3E-05	-2.1726	6.1E-10	7E-08	262.2	113.6	155.2	1946	869.8	709.4
Ap1s1	11769	AP-1 complex subunit sigma-1A	3.16325	0	0	-1.8882	1.7E-09	1.9E-07	-2.566	0	0	910.6	371.6	326.4	5067	2224	1541
Raver1	71766	Ribonucleoprotein PTB-binding 1	3.31913	0	0	-1.7558	3.7E-06	0.00017	-2.0759	1.9E-09	2E-07	1112	266.2	285	6471	3055	2461
BC048403	270802	Protein BC048403	2.83833	8.9E-13	1.1E-11	-1.9038	8.1E-06	0.00034	-2.3206	6.1E-09	5.7E-07	221.4	59.2	56.2	1113	484.8	371.8
Git1	216963	Putative uncharacterized protein	6.64929	0	0	-1.749	6.8E-05	0.00211	-1.5928	0.00091	0.01608	419.4	220.2	187	4892	2318	2432
Reep4	72549	Receptor expression-enhancing protein 4	7.64803	0	0	-1.4424	0.0017	0.02944	-1.8343	2.1E-07	1.3E-05	204.4	124.8	82.6	2742	1580	1169
Ywhae	22627	14-3-3 protein epsilon	2.24769	2.4E-06	1.7E-05	-2.494	1E-07	7.3E-06	-2.6208	2.1E-08	1.7E-06	7520	1959	2172	29746	9895	8740
Sp2	78912	Sp2 transcription factor	1.75967	0.00113	0.00571	-2.5786	4.9E-08	3.7E-06	-2.6272	2.8E-08	2.2E-06	205	27	30.4	636	204	187.8
Nup62	18226	Nuclear pore glycoprotein p62	6.24205	0	0	-1.5469	0.00051	0.01142	-1.7652	6.1E-06	0.00025	497.6	195.6	184.4	5503	2955	2464
BC052040	399568	Protein BC052040	3.53649	3.8E-13	4.9E-12	-1.7809	0.00073	0.01524	-2.1754	5.8E-06	0.00024	127.8	42.4	36.8	788	366.4	283.4
C1qtnf6	72709	Complement C1q tumor necrosis factor-related protein 6	1.76218	0.00888	0.0388	-3.009	4.1E-07	2.5E-05	-3.0058	4.5E-07	2.6E-05	77.2	16.6	15.4	238	65.6	63
Znhit6	229937	Box C/D snoRNA protein 1	3.38094	0	0	-1.9787	7E-07	4E-05	-2.0601	1.5E-07	1E-05	281.8	75.4	98.6	1677	704.4	622.4
Lsm2	27756	U6 snRNA-associated Sm-like protein LSm2	4.9599	0	0	-1.7293	9E-07	5E-05	-2.0489	1.3E-10	1.8E-08	402.8	191.8	200	3507	1689	1348
Snrpd3	67332	Small nuclear ribonucleoprotein Sm D3	2.84036	3.6E-11	4E-10	-2.1988	5.6E-07	3.3E-05	-2.0755	3.5E-06	0.00016	1226	256.2	352	6125	2315	2204
Fgfbp3	72514	Fibroblast growth factor-binding protein 3	3.54138	8.9E-08	7.2E-07	-2.0931	0.00096	0.01884	-2.1477	0.00067	0.01261	20.4	5.2	10.2	128	50.6	46.6
Rcc1	100088	Regulator of chromosome condensation	15.1207	0	0	-1.4854	0.00094	0.01856	-1.482	0.00101	0.01755	275.6	148.2	144	7342	4111	3908
Erdr1	170942	Protein Erdr1	2.03536	0.00089	0.00457	-2.0089	0.00111	0.021	-2.4124	3.8E-05	0.00118	3033	223.8	287.6	10893	4494	3376
Srrm1	51796	Serine/arginine repetitive matrix protein 1	2.24902	1.3E-08	1.2E-07	-2.3435	2.3E-09	2.4E-07	-2.5329	7.3E-11	1.1E-08	2289	434	479.4	9053	3200	2745
Zbed3	72114	Putative uncharacterized protein	2.88995	1.2E-11	1.4E-10	-2.0403	3.8E-06	0.00018	-2.2992	7.5E-08	5.4E-06	154.6	35.2	40.6	783.3	318.2	266
Ube2d1	216080	Ubiquitin-conjugating enzyme E2 D1	2.45712	1.1E-09	1E-08	-2.2769	2.1E-08	1.7E-06	-2.5587	1.6E-10	2.2E-08	360.2	91.4	82.6	1549	563.8	467
Ppif	105675	Peptidyl-prolyl cis-trans isomerase F, mitochondrial	5.12348	0	0	-1.7828	2.1E-06	0.00011	-2.0687	2.5E-09	2.5E-07	1280	743.6	514.4	11549	5375	4376
Mnt	17428	Max-binding protein MNT	2.49051	1.7E-06	1.2E-05	-2.362	6.3E-06	0.00027	-2.887	2.7E-08	2.1E-06	400	144.8	91.8	1738	609.6	470.4
Clec4a1	269799	C-type lectin domain family 4, member a1	3.51274	6.1E-07	4.6E-06	-3.6082	2.4E-07	1.6E-05	-2.6424	8.3E-05	0.00227	36	33.8	16.4	222.5	51.2	66.8
Zbtb12	193736	Protein Zbtb12	5.87991	0	0	-1.6906	0.00042	0.00962	-1.7535	0.00016	0.00391	116.6	33.6	35	1213	596.4	532
Tdrkh	72634	Tdrkh protein	4.76957	0	0	-2.1089	4.2E-05	0.00142	-1.7067	0.00333	0.04481	178.6	67	58	1504	591.4	696.2
Ptp4a3	19245	Protein tyrosine phosphatase type IVA 3	6.8553	0	0	-1.7516	5.6E-05	0.0018	-1.9341	2.2E-06	0.0001	268	185.8	106.4	3210	1522	1263
Cpne8	66871	Cpne8 protein	4.84389	4.7E-12	5.6E-11	-2.3477	0.00015	0.00405	-2.7432	7.4E-06	0.0003	83.8	76.8	62	728.8	259.6	207.4
Fmnl1	57778	Formin-like protein 1	5.87302	7.3E-13	9.3E-12	-2.408	0.00024	0.00621	-2.0181	0.00335	0.04507	41.6	40.6	24.8	424.3	147.4	167.8
Rpl27	1.08E+08	60S ribosomal protein L27	4.73339	0	0	-1.9131	2.8E-08	2.3E-06	-1.9706	6.5E-09	6.1E-07	396.8	144.6	119.8	3323	1443	1325
Spc24	67629	SPC24, NDC80 kinetochore complex component, homolog (S. cere	13.4103	0	0	-1.4764	0.00129	0.02387	-1.8635	2.8E-07	1.7E-05	285.8	198.4	188.8	6719	3790	2813
Impdh1	23917	Inosine-5'-monophosphate dehydrogenase 1	29.5259	0	0	-1.5266	0.00011	0.00321	-1.4883	0.00028	0.00615	117.2	90.4	62.6	6116	3328	3229
Pelp1	75273	Proline, glutamic acid and leucine rich protein 1, isoform CRA_b	5.31046	0	0	-1.7357	8E-07	4.5E-05	-1.9062	7.9E-09	7.2E-07	618.8	165.6	170.4	5791	2767	2359
Hmgn2	15331	Non-histone chromosomal protein HMG-17	8.5217	0	0	-2.015	2.9E-06	0.00014	-1.66	0.00071	0.0132	688.4	368.6	499	10204	4212	4896
Nle1	217011	Notchless protein homolog 1	6.49878	0	0	-1.6438	0.00025	0.00628	-1.8895	2.8E-06	0.00013	162.2	51.2	48	1867	943	770.6
Cbr3	109857	Carbonyl reductase [NADPH] 3	7.57078	2.3E-11	2.5E-10	-2.6849	0.00032	0.00774	-2.2196	0.00356	0.04702	9.2	12.6	11.8	123.3	38.4	44.6
Lyar	17089	Cell growth-regulating nucleolar protein	10.3421	0	0	-1.6949	4.3E-05	0.00144	-1.6179	0.00019	0.00451	406.6	164.8	188.2	7421	3644	3481
Tulp3	22158	Tubby-related protein 3	13.5979	0	0	-1.5352	0.00284	0.04431	-1.7668	7.7E-05	0.00211	49.8	29.6	31.6	1186	642.6	522.4
1810032O08Rik	66293	Protein 1810032O08Rik	6.73762	0	0	-1.7787	0.00086	0.01719	-1.7704	0.00095	0.01663	97.8	32.2	35.2	1175	548.6	518
Hnrnpa2b1	53379	Heterogeneous nuclear ribonucleoprotein A2/B1	2.18066	0.00013	0.00076	-2.8792	2.1E-07	1.4E-05	-2.6576	1.6E-06	8.1E-05	15910	2778	3413	60954	17630	17264
Ttyh3	78339	Protein tweety homolog 3	5.67908	0	0	-1.6966	0.00012	0.00342	-2.0695	1.2E-07	8.3E-06	411.8	149.4	103.8	4078	1992	1571
Pa2g4	18813	Proliferation-associated protein 2G4	5.25713	0	0	-1.837	0.00021	0.0055	-1.972	3.5E-05	0.00111	3093	789.6	1065	28647	12945	11188
Wasf1	83767	Wiskott-Aldrich syndrome protein family member 1	2.8846	2.1E-06	1.5E-05	-2.113	0.00071	0.0148	-2.2691	0.00021	0.00496	89.2	12	13.4	448.3	176.4	154.8
Tmem136	235300	Transmembrane protein 136	5.06252	2.2E-16	3.5E-15	-1.8774	0.00088	0.01751	-2.1764	4.3E-05	0.0013	46	20.2	14.4	410.8	181.6	147.2
Plagl2	54711	Putative uncharacterized protein	6.29383	0	0	-1.6357	0.00037	0.00861	-2.0495	2.1E-07	1.3E-05	263.6	78.6	68.4	2907	1474	1121
Ssbp4	76900	Single stranded DNA binding protein 4, isoform CRA_b	14.173	0	0	-1.642	1.2E-05	0.00047	-1.7912	2.7E-07	1.7E-05	112.8	95.6	67.2	2809	1423	1247
Car12	76459	Carbonic anhydrase 12	2350.51	0	0	-1.4536	0.00202	0.03379	-1.4793	0.00123	0.02049	1.2	0.4	1.2	5576	3187	2947
Syne4	233066	Nesprin-4	21.9082	0	0	-1.5372	0.00249	0.03978	-1.609	0.00083	0.01485	69	19.4	20.4	2654	1435	1310
Pidd1	57913	p53-induced death domain-containing protein 1	4.07557	0	0	-1.7903	1.2E-05	0.00046	-2.3396	1.9E-10	2.5E-08	146.6	34.6	23.2	1055	489	351.8
Banf1	23825	Barrier-to-autointegration factor	5.65265	0	0	-2.038	1.4E-10	1.8E-08	-2.11	1.7E-11	2.8E-09	796.2	363.8	348.2	7916	3226	2899
Pcbp1	23983	Poly(rC)-binding protein 1	2.11547	6.1E-09	5.5E-08	-2.8806	2.2E-16	6.7E-14	-3.1789	0	0	5636	988.6	997.2	20967	6033	5085
Nob1	67619	RNA-binding protein NOB1	5.86714	0	0	-1.8008	4.1E-06	0.00019	-2.0429	2.3E-08	1.9E-06	377.2	105	108.4	3926	1810	1494
Snrpf	69878	Small nuclear ribonucleoprotein F	10.1158	0	0	-1.8424	5.5E-06	0.00024	-1.7434	3.5E-05	0.00111	330.4	149.2	161.8	5904	2666	2581
Lmnb1	16906	Lamin-B1	25.5465	0	0	-1.5157	0.00305	0.04682	-1.6791	0.00022	0.00513	230.8	86.8	96.6	10299	5641	4731

S4 Table. Continue...

Name	NCBI gene ID	Database object name	NT vs NL			MT vs NT			LT vs NT			Mean					
			Fold Change	P-Value	FDR P-Value	Fold Change	P-Value	FDR P-Value	Fold Change	P-Value	FDR P-Value	NL	ML	LL	NT	MT	LT
Anp32e	66471	Acidic leucine-rich nuclear phosphoprotein 32 family member E	5.23876	0	0	-2.0161	1.4E-07	9.8E-06	-2.0303	1.1E-07	7.5E-06	1093	317.6	368.8	10077	4157	3802
Slc7a5	20539	Putative uncharacterized protein	42.3999	0	0	-1.5335	0.0006	0.01294	-1.6671	4.1E-05	0.00126	105.2	96.8	80.2	7807	4226	3699
Faap24	101831	Fanconi anemia-associated protein of 24 kDa	22.159	0	0	-1.5361	0.00095	0.01867	-1.7807	9.1E-06	0.00035	69.8	45.6	33.8	2742	1487	1188
Slc1a5	20514	Amino acid transporter	44.1578	0	0	-1.5082	0.00055	0.01199	-1.6972	8.7E-06	0.00034	66.2	82.8	24.2	5127	2825	2416
Alg8	381903	Putative uncharacterized protein	4.2039	0	0	-2.0424	7.5E-09	6.8E-07	-2.463	3.3E-13	7.1E-11	344.8	113	104	2555	1039	819.8
Arhgdia	192662	Rho GDP-dissociation inhibitor 1	4.12594	0	0	-2.1227	5E-10	5.9E-08	-2.3913	5.9E-13	1.2E-10	2446	764.4	749.2	17763	6948	5853
Fam98b	68215	Protein FAM98B	2.17337	1E-05	6.8E-05	-2.8433	2.8E-09	2.9E-07	-3.1638	6.1E-11	9E-09	495.8	89	77.6	1886	550.6	452.6
Cln6	76524	Cln6 protein	8.45983	0	0	-1.777	0.00021	0.00538	-2.1927	4.2E-07	2.5E-05	100.6	81.2	39.4	1497	699.6	545.2
Slc39a10	227059	Zinc transporter ZIP10	16.0952	0	0	-1.8767	1E-07	7.1E-06	-1.8516	1.9E-07	1.2E-05	178.2	228.2	165.8	5049	2227	2111
Ddit4l	73284	DNA-damage-inducible transcript 4-like	2.72367	0.00328	0.01545	-5.1864	1.6E-06	8.4E-05	-5.4571	8.5E-07	4.5E-05	35.8	31	12.2	171.8	27.6	23.4
Gm9493	670565	Protein Gm9493	4.06552	7.4E-12	8.6E-11	-4.0733	6.7E-12	1.2E-09	-2.8181	4.1E-07	2.4E-05	928.2	671.4	583.4	6674	1365	1744
Srm	20810	Putative uncharacterized protein	11.9329	0	0	-1.6174	0.00298	0.04604	-1.9284	5E-05	0.00148	1201	361.4	461.8	25139	12881	10183
Hnrnpa1	15382	Heterogeneous nuclear ribonucleoprotein A1	10.8415	0	0	-1.8275	0.00131	0.02413	-1.7841	0.00203	0.03024	2029	783.6	774.2	38597	17567	16510
Dcakd	68087	Dephospho-CoA kinase domain-containing protein	3.39726	0	0	-2.2642	3.2E-09	3.2E-07	-2.5549	1.1E-11	1.9E-09	909.2	188.2	210.6	5433	1991	1668
Fbl	14113	rRNA 2'-O-methyltransferase fibrillarin	6.95178	0	0	-1.9345	4E-07	2.4E-05	-1.9507	2.8E-07	1.7E-05	1014	285.8	336.8	12368	5317	4896
Mapre2	212307	Microtubule-associated protein RP/EB family member 2	9.1597	0	0	-1.7215	0.00017	0.00455	-2.1865	6.1E-08	4.4E-06	618.6	348.6	197.2	9861	4754	3583
Slc19a1	20509	Folate transporter 1	13.5482	0	0	-1.6432	2.1E-05	0.00076	-2.0093	2.3E-09	2.3E-07	300.4	135.8	118.2	7159	3605	2803
Gdf15	23886	Putative uncharacterized protein	4.57015	3.1E-09	2.9E-08	-2.6734	0.00011	0.00326	-2.2904	0.00113	0.01913	136.2	72.6	37.2	1087	337	372
Plp2	18824	Proteolipid protein 2	35.8944	0	0	-1.7407	4.2E-05	0.00142	-1.6022	0.0005	0.00989	56	24.6	18.2	3527	1685	1751
Tagln2	21346	Transgelin-2	4.57453	1.1E-16	1.8E-15	-3.1871	2.8E-10	3.5E-08	-1.9547	0.00026	0.00581	443.6	190.8	138.8	3530	919.4	1463
Cherp	27967	Calcium homeostasis endoplasmic reticulum protein, isoform CRA	2.34104	3.7E-10	3.7E-09	-2.69	3E-13	6.1E-11	-3.2372	0	0	1158	162.6	157.4	4779	1471	1150
Zfp296	63872	Protein Zfp296	12.392	0	0	-1.8498	0.00155	0.02748	-1.7612	0.00361	0.04758	22.4	8.4	5	494.5	221.8	221.6
Trp53	22059	Cellular tumor antigen p53	3.67943	0	0	-2.1499	1.1E-10	1.5E-08	-2.6951	1.1E-16	3.4E-14	810.6	156	170.2	5264	2030	1539
Ndrg1	17988	N-myc downstream regulated gene 1	7.88046	0	0	-2.008	3.8E-05	0.00129	-2.1616	5.2E-06	0.00022	940	328.2	509.4	13004	5316	4697
Erh	13877	MCG7617, isoform CRA_a	5.53352	0	0	-2.3573	6.3E-09	5.8E-07	-2.6348	5.5E-11	8.1E-09	484.8	258.4	235.2	4694	1655	1403
Them6	223626	Protein THEM6	11.8811	0	0	-1.5347	0.0027	0.04235	-2.5932	2.9E-11	4.5E-09	103.4	51.4	50	2152	1166	643.8
Tnfaip3	21929	Tumor necrosis factor alpha-induced protein 3	6.29632	0	0	-2.6325	2.1E-08	1.8E-06	-2.6668	1.4E-08	1.2E-06	99	81.4	67.6	1106	347.8	322.4
Ddr1	12305	Epithelial discoidin domain-containing receptor 1	3.18289	1.9E-05	0.00012	-4.3232	6.2E-08	4.7E-06	-3.4064	5.5E-06	0.00023	62.6	37	22.2	350.8	67.6	80.6
Gpatch4	66614	G patch domain-containing protein 4	4.82438	0	0	-2.4244	6.9E-08	5.1E-06	-2.0076	2.2E-05	0.00074	329	70.4	62.2	2804	963.8	1039
Hnrnpd	11991	Heterogeneous nuclear ribonucleoprotein D0	3.51472	0	0	-2.6	2.3E-11	3.5E-09	-2.5987	2.4E-11	3.7E-09	1551	306.2	339.4	9546	3044	2754
Naf1	234344	H/ACA ribonucleoprotein complex non-core subunit NAF1	8.09169	0	0	-1.9449	1.3E-07	8.7E-06	-2.2097	3.1E-10	3.9E-08	234.8	83.4	79.2	3361	1435	1189
Hmga1	15361	High mobility group protein HMG-I/HMG-Y	30.7682	0	0	-1.6181	0.00093	0.01838	-1.9863	2.4E-06	0.00011	280.6	168	109.8	15346	7867	5927
Pcgf2	22658	Polycomb group RING finger protein 2	2.57393	8.2E-08	6.6E-07	-2.5892	6E-08	4.5E-06	-3.3058	1.1E-11	2E-09	259	35.6	30.2	1178	376.8	273.2
2310022A10Rik	66367	Protein 2310022A10Rik	3.25408	0	0	-2.6419	3E-12	5.4E-10	-2.9051	2.2E-14	5.3E-12	223.2	49	46.4	1279	401.4	343.6
Mthfd2	17768	Bifunctional methylenetetrahydrofolate dehydrogenase/cyclohyd	18.7665	0	0	-1.9459	0.00037	0.00861	-1.8103	0.00149	0.02373	128.4	42	80.8	4331	1854	1848
Kctd17	72844	Kctd17 protein	8.17826	0	0	-2.0391	5.9E-06	0.00026	-2.2847	1.6E-07	1E-05	90.8	39.2	29.4	1318	536.4	452.6
Palm	18483	Putative uncharacterized protein	24.1894	0	0	-1.8509	2.8E-05	0.001	-1.8788	1.8E-05	0.00063	86.4	37	39.8	3634	1635	1526
Evc2	68525	Putative uncharacterized protein	8.44126	0	0	-2.8187	8.5E-07	4.7E-05	-2.1234	0.00034	0.00728	76.6	74.4	31.2	1123	332.4	413.6
Ddit4	74747	DNA-damage-inducible transcript 4	4.85723	1.1E-09	1E-08	-3.0034	2.1E-05	0.00077	-2.5189	0.00035	0.00748	325.2	183.4	75.8	2790	763.6	842.4
Pycr1	209027	Pyrroline-5-carboxylate reductase 1, mitochondrial	15.0584	0	0	-2.0129	0.00029	0.00717	-1.9305	0.00066	0.01249	22.4	8	8.2	594.5	244.4	237.2
Maff	17133	Transcription factor MafF	11.958	0	0	-2.3237	0.00089	0.01774	-2.3007	0.00103	0.01775	55.2	56	46.4	1200	420.6	398.4
Rbm38	56190	RNA-binding protein 38	37.0175	0	0	-1.6947	7.1E-06	0.00031	-1.9772	6.7E-09	6.2E-07	102.2	30.8	27	6648	3260	2635
Mybl2	17865	Myb-related protein B	157.949	0	0	-1.5373	0.0014	0.02546	-2.0862	4.8E-08	3.6E-06	29.8	7	6.2	8324	4483	3089
Prr5	109270	Proline-rich protein 5	6.03824	0	0	-2.3951	5.3E-08	4E-06	-2.4542	2.3E-08	1.9E-06	109.8	38.8	37.6	1160	402	367.6
Hmgb2	97165	High mobility group protein B2	40.7424	0	0	-1.8849	1.6E-05	0.0006	-1.8614	2.3E-05	0.00079	181.2	118.8	94.4	12844	5667	5225
Ubap2	68926	Ubiquitin-associated protein 2	5.54961	0	0	-2.1747	9.9E-10	1.1E-07	-2.4626	1.4E-12	2.7E-10	977.4	176	186.8	9546	3640	3027
Sf3a2	20222	Splicing factor 3A subunit 2	3.83944	0	0	-2.5114	2E-15	5.3E-13	-2.8019	0	0	607.8	96.6	110.6	4107	1358	1138
Cd276	102657	CD276 antigen	4.10564	0	0	-2.1653	7.2E-07	4.1E-05	-3.2007	9.6E-14	2.2E-11	400.2	59.6	77.6	2873	1099	708
C1qbp	12261	Complement component 1 Q subcomponent-binding protein, mito	5.9416	0	0	-2.2667	9.6E-08	6.8E-06	-2.6802	1.3E-10	1.8E-08	2816	907	846.8	29591	10823	8532
Rad51	19361	DNA repair protein RAD51 homolog 1	53.5573	0	0	-1.7493	0.0003	0.00727	-1.996	7.9E-06	0.00031	31.2	17.6	8.4	2942	1395	1132
Gyltl1b	228366	Glycosyltransferase-like protein LARGE2	34.6576	0	0	-1.8337	2.8E-05	0.00099	-2.1395	1.5E-07	1E-05	33.4	50.8	10.6	2034	921.8	746
Cct6b	12467	T-complex protein 1 subunit zeta-2	2.90675	1E-08	9.3E-08	-5.3592	0	0	-5.3689	0	0	47	18.4	23.8	240.3	37.2	35
E2f1	13555	E2F1	8.43379	0	0	-2.0766	4.7E-06	0.00021	-2.533	6.2E-09	5.8E-07	106.6	47	28	1583	630.6	483.8
Smn1	20595	Survival motor neuron protein	5.43693	0	0	-2.4041	6.3E-11	9E-09	-2.8158	1.4E-14	3.4E-12	225.2	63	69.2	2154	744.4	588.4
Fkbp1a	14225	Peptidyl-prolyl cis-trans isomerase	4.34805	0	0	-2.7339	4.1E-14	9.2E-12	-3.1162	0	0	1916	541	417.6	14617	4435	3606
Slc29a2	13340	Equilibrative nucleoside transporter 2	29.6142	0	0	-1.8366	4.3E-05	0.00144	-2.2623	4.2E-08	3.2E-06	32	10.4	12.4	1673	754.8	578
Sf3b4	107701	Splicing factor 3B subunit 4	4.08604	0	0	-2.7752	0	0	-3.1534	0	0	656.2	142.2	129.6	4717	1411	1176
Prmt1	15469	Protein arginine N-methyltransferase 1	12.2802	0	0	-2.0991	4.7E-09	4.5E-07	-2.3958	5.2E-12	9.4E-10	563	160.6	171.4	12179	4815	3925
Fus	233908	RNA-binding protein FUS	2.77541	8E-07	6E-06	-3.3838	3.8E-09	3.7E-07	-3.6541	3.8E-10	4.5E-08	3228	351.4	373.2	15774	3857	3160
Zcchc10	67966	Zinc finger CCHC domain-containing protein 10	4.13164	1.2E-14	1.8E-13	-3.271	7E-11	1E-08	-3.213	1.4E-10	1.9E-08	112.6	29	31	819	208	198.4
Gadd45b	17873	Growth arrest and DNA damage-inducible protein GADD45 beta	27.1224	0	0	-2.4285	5.9E-07	3.5E-05	-1.9059	0.00028	0.00617	43.6	31.4	10	2054	703	841.8
Psrc1	56742	Proline/serine-rich coiled-coil protein 1	195.687	0	0	-2.0943	0.00017	0.0045	-1.9939	0.00044	0.00891	7.2	8.4	2.4	2540	1003	985.6
Sox12	20667	Transcription factor SOX-12	7.43332	0	0	-2.1982	7.5E-07	4.3E-05	-2.8187	8E-11	1.1E-08	198	27	31.8	2560	968.6	711.2
Troap	78733	Tastin	31.4271	0	0	-1.9989	0.00068	0.01439	-2.2772	5.6E-05	0.00163	12.4	4.2	0.8	690.8	287.4	238
Asb16	217217	Asb16 protein	57.5305	0	0	-1.8222	0.00158	0.02773	-2.4391	2.9E-06	0.00013	7.8	1.6	1	802.3	365	252.8
Mtfr2	71804	Mitochondrial fission regulator 2	25.6962	0	0	-2.0558	0.0007	0.01472	-2.3085	8.8E-05	0.00237	8.4	2.6	1.2	386.3	156	130.6
Jun	16476	Transcription factor AP-1	6.55277	7E-13	8.9E-12	-3.8956	2E-07	1.3E-05	-2.4773	0.00052	0.01028	753.4	288.2	269.8	8731	1855	2700
Meg3		Putative uncharacterized protein	9.27508	0	0	-2.1518	0.00222	0.03644	-2.7543	5.3E-05	0.00156	116	4.2	12.4	1845	712.8	519
Tceal5	331532	Transcription elongation factor A protein-like 5	161.733	0	0	-2.2404	0.0001	0.00305	-2.1384	0.00026	0.00576	1.6	3.6	1	498.5	184.8	176.2
Cd63	12512	CD63 antigen	13.7457	0	0	-3.6781	9.8E-11	1.4E-08	-2.1565	0.00013	0.0033	125.6	139.8	37.2	3045	686.8	1108
Gar1	68147	H/ACA ribonucleoprotein complex subunit 1	7.69492	0	0	-2.8067	6.8E-08	5E-06	-2.5064	1.6E-06	7.8E-05	208.2	24	41.6	2856	847	852.2
Rad9b	231724	Cell cycle checkpoint control protein RAD9B	7.48976	0	0	-4.7422	3.4E-11	5.1E-09	-2.57	5.4E-05	0.00159	75	40.6	43.2	989.8	174	294.2
Ankrd13b	268445	Ankyrin repeat domain-containing protein 13B	8.79757	0	0	-2.5708	1.3E-10	1.8E-08	-2.8961	5.1E-13	1E-10	145	47	18	2238	723.4	592.6
Tnfrsf10b	21933	Tumor necrosis factor receptor superfamily member 10B	13.3872	0	0	-2.8603	4.9E-06	0.00022	-2.6218	2.8E-05	0.00091	28.6	23.4	7.4	681	197.6	203.6
Prss16	54373	Protease, serine, 16 (Thymus), isoform CRA_d	84.3073	0	0	-2.3332	0.00034	0.00815	-2.1513	0.0012	0.02014	1.2	0.4	0.2	198.8	71	74
Dram1	71712	DNA damage-regulated autophagy modulator protein 1	39.6705	0	0	-2.3668	0.00025	0.00641	-3.04	2.5E-06	0.00012	15.4	28.6	21	1087	387	282
Lpcat4	99010	Lysophospholipid acyltransferase LPCAT4	164.363	0	0	-1.9654	8.8E-05	0.00265	-2.8956	7.2E-10	8E-08	16	8.4	6.4	4666	1977	1252
Ncl	17975	Nucleolin	13.3626	0	0	-2.904	8.3E-06	0.00035	-2.4033	0.00025	0.00555	6313	1071	1122	1E+05	42291	44383
Hif3a	53417	Hypoxia-inducible factor 3-alpha	596.765	0	0	-2.1383	0.00017	0.00459	-2.5822	2.7E-06	0.00013	5.4	2.6	2.4	5829	2233	1746
Dusp8	18218	Dual-specificity protein phosphatase 8	5.95284	3.3E-07	2.5E-06	-4.108	4.6E-05	0.0015	-3.0519	0.00126	0.02087	51.2	20.4	7	574.3	116.2	149
Prr22	1.01E+08	Protein Prr22	10.335	0	0	-2.5477	1.4E-05	0.00055	-3.8709	7.9E-10	8.9E-08	10.6	1.6	1.8	194	63.4	39.4
Phlda3	27280	Pleckstrin homology-like domain family A member 3	14.5574	2.2E-16	3.5E-15	-3.9753	1.2E-05	0.00049	-3.3391	0.00013	0.00333	24.2	19.4	14	624.3	130.8	142.4
S100a11	20195	Protein S100-A11	3.05907	4.6E-07	3.5E-06	-7.2256	0	0	-7.6757	0	0	112.2	23	17.6	600.5	68.8	61.4

S4 Table. Continue...

Name	NCBI gene ID	Database object name	NT vs NL			MT vs NT			LT vs NT			Mean					
			Fold Change	P-Value	FDR P-Value	Fold Change	P-Value	FDR P-Value	Fold Change	P-Value	FDR P-Value	NL	ML	LL	NT	MT	LT
Pmaip1	58801	Phorbol-12-myristate-13-acetate-induced protein 1	44.4509	0	0	-5.2197	9.8E-11	1.4E-08	-2.3349	0.00085	0.01515	12	25.6	12.8	956.3	151.4	321.4
Saa3	20210	Serum amyloid A-3 protein	10.7445	0	0	-4.0308	6.7E-08	4.9E-06	-6.3403	9.9E-13	1.9E-10	87.4	63.6	98.2	1687	349.2	204
Lypd8	70163	RIKEN cDNA 2210415F13, isoform CRA_a	3504.71	4.1E-12	4.9E-11	-2.5704	0.0004	0.00926	-3.0785	2.5E-05	0.00085	0	0	0	930	301.8	236
Dlk1	13386	Delta-like 1	3257.23	0	0	-2.0532	1.1E-05	0.00044	-4.6798	0	0	0.8	0.2	0.6	5490	2216	941.6
Prr7	432763	Proline-rich protein 7	43.5301	0	0	-4.315	9.7E-08	6.8E-06	-2.5372	0.00058	0.01119	2.4	2.2	2.2	195.5	37.6	61.6
Tubb2b	73710	TUBB2B protein, MCG1395	72.7595	0	0	-3.6117	5.2E-06	0.00023	-2.854	0.00019	0.00454	4	6.6	2.8	534.8	123.6	146.8
Fbxl16	214931	F-box/LRR-repeat protein 16	27.4925	0	0	-2.7739	0.00139	0.02526	-5.1116	4.2E-07	2.4E-05	6.6	7.8	2.4	326.3	97.6	51
Muc13	17063	Mucin-13	120.39	0	0	-2.2275	0.0018	0.03082	-6.2169	5.5E-12	9.8E-10	1	2	1	242.3	90.8	30.2
Atf3	11910	Activating transcription factor 3	30.3051	0	0	-6.7152	3.9E-09	3.8E-07	-2.7025	0.00208	0.03088	60.6	83.6	36	3225	400.2	923.8
Sncg	20618	Gamma-synuclein	11.777	2.4E-07	1.9E-06	-17.531	2.3E-09	2.4E-07	-3.1074	0.00376	0.04908	2	1.4	1.8	44.75	2	10.8
Cdkn1a	12575	Cyclin-dependent kinase inhibitor 1	4.15257	8.3E-05	0.00049	-8.5325	3.1E-09	3.1E-07	-5.0406	7.7E-06	0.00031	713.2	130.4	22.2	5634	545.8	856.6
Stc2	20856	Stanniocalcin-2	34.2503	0	0	-7.36	1.2E-11	1.9E-09	-2.4898	0.00149	0.0238	4.4	2.6	2.6	273.3	31	90.2
Atp1b2	11932	ATPase, Na+/K+ transporting, beta 2 polypeptide	7.55682	1.1E-16	1.8E-15	-3.6688	2.6E-08	2.1E-06	-5.9264	6.6E-14	1.5E-11	26.8	1.2	4	356.8	80.2	46.6
Gpx3	14778	Glutathione peroxidase	16.7124	0	0	-4.4351	1.4E-14	3.4E-12	-4.6374	2.4E-15	6.6E-13	79.6	39	22.2	2349	435.6	399
Anp32b	67628	Putative uncharacterized protein	7.86576	0	0	-7.3731	0	0	-5.5217	0	0	1858	110	125.8	25713	2892	3294
Cxcl10	15945	C-X-C motif chemokine	25.3968	0	0	-12.923	2.3E-15	6E-13	-4.846	9.5E-07	5E-05	61.2	37.6	30.2	2759	177.6	453
Fosb	14282	FBJ osteosarcoma oncogene B	18.2043	1.1E-11	1.2E-10	-9.6307	2.7E-08	2.2E-06	-6.3214	5.1E-06	0.00022	8	1	3.4	259	22.2	31.4
Cxcl2	20310	C-X-C motif chemokine 2	33.2209	4.4E-16	6.9E-15	-13.323	6.5E-11	9.4E-09	-19.547	2E-13	4.4E-11	4.8	0.8	2	301.3	18.8	11.4
