# Supplementary material for: Inhibition of hepatocellular carcinoma by metabolic normalization
Source: PLoS One. 2019 Jun 26;14(6):e0218186. doi: 10.1371/journal.pone.0218186 (PMC6594671; doi:10.1371/journal.pone.0218186)

Fig.1-B_GAPDH_set1&set2

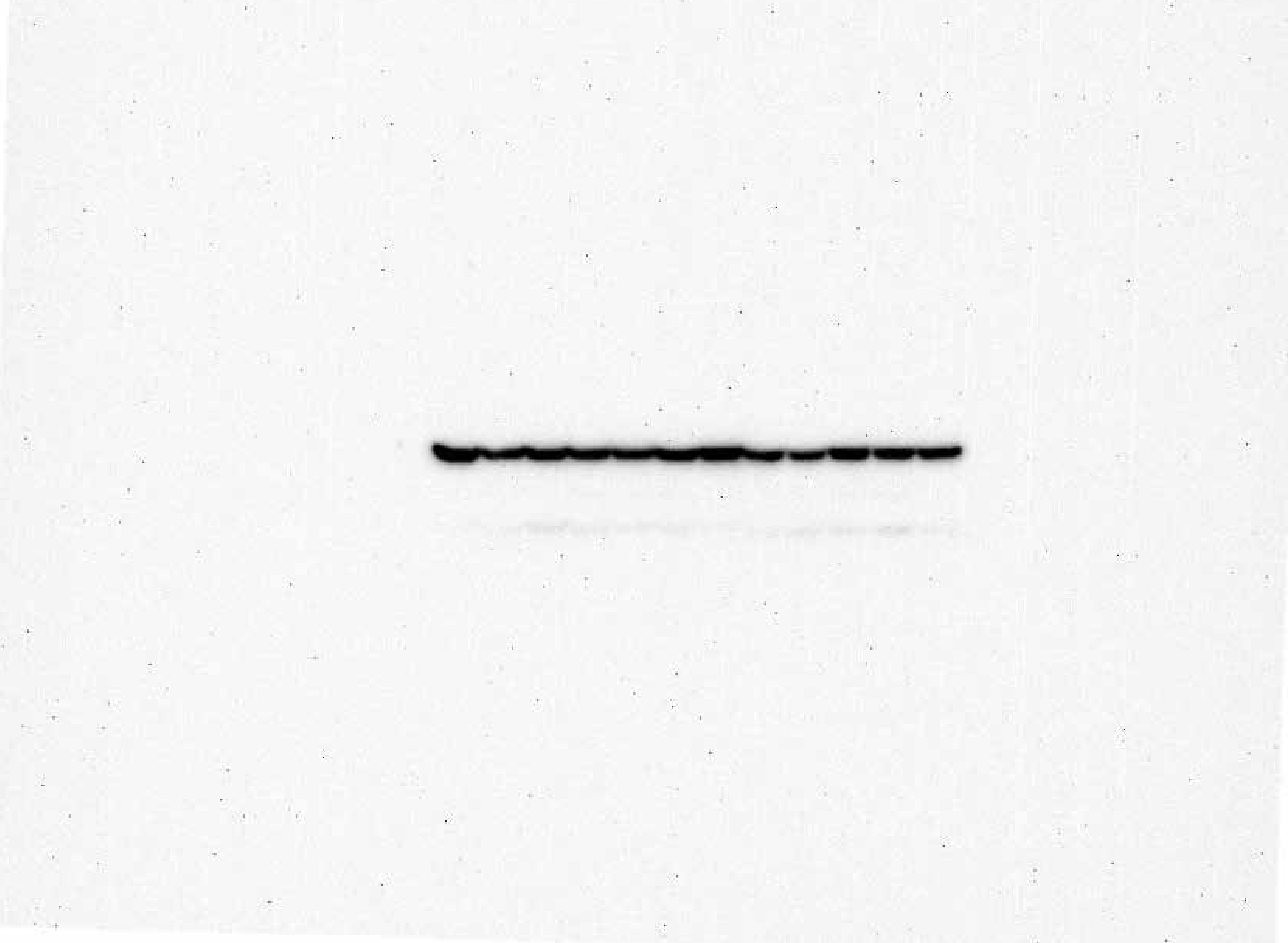

Fig.1-B_GAPDH_set1&set2_marker

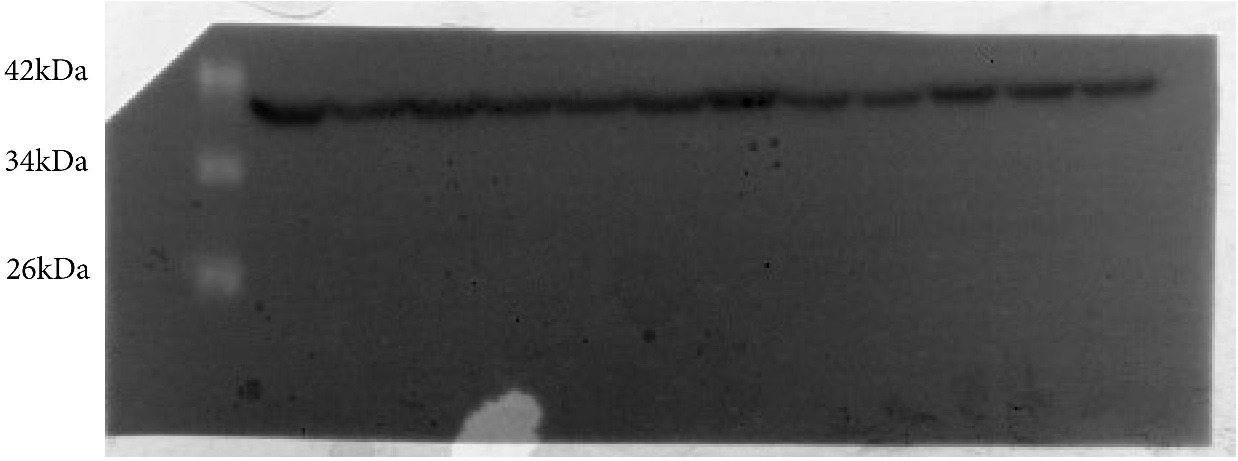

Fig.1-B_GAPDH_set3&set4

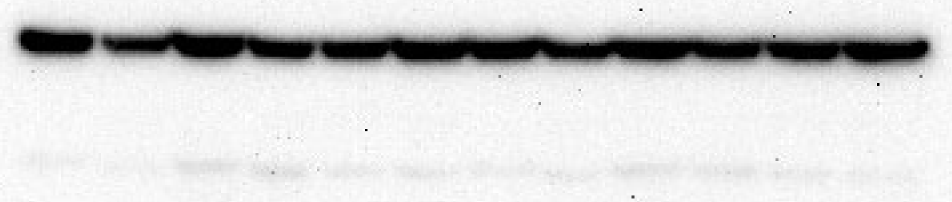

Fig.1-B_GAPDH_set3&set4_marker

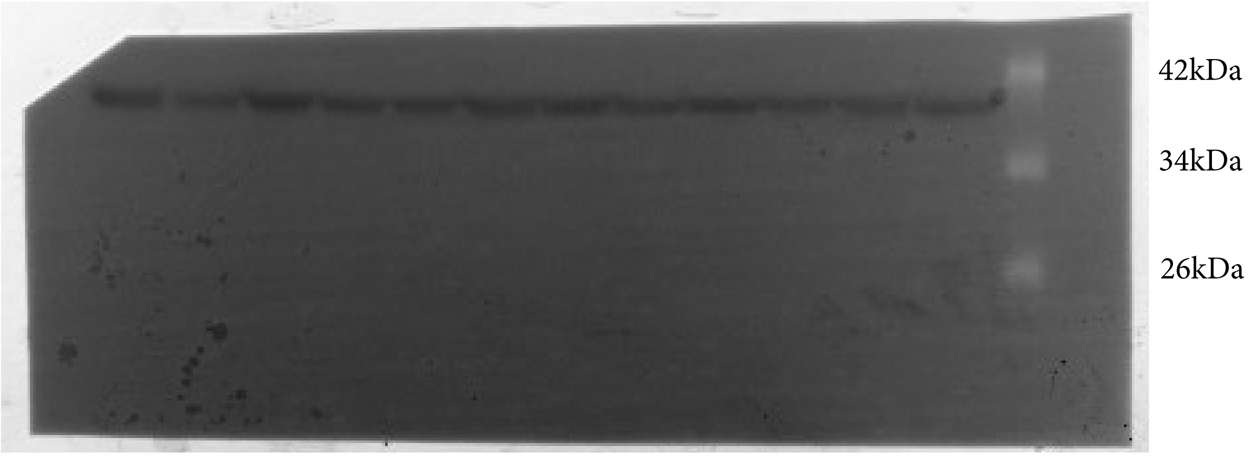

Fig.1-B_Myc_set1&set2

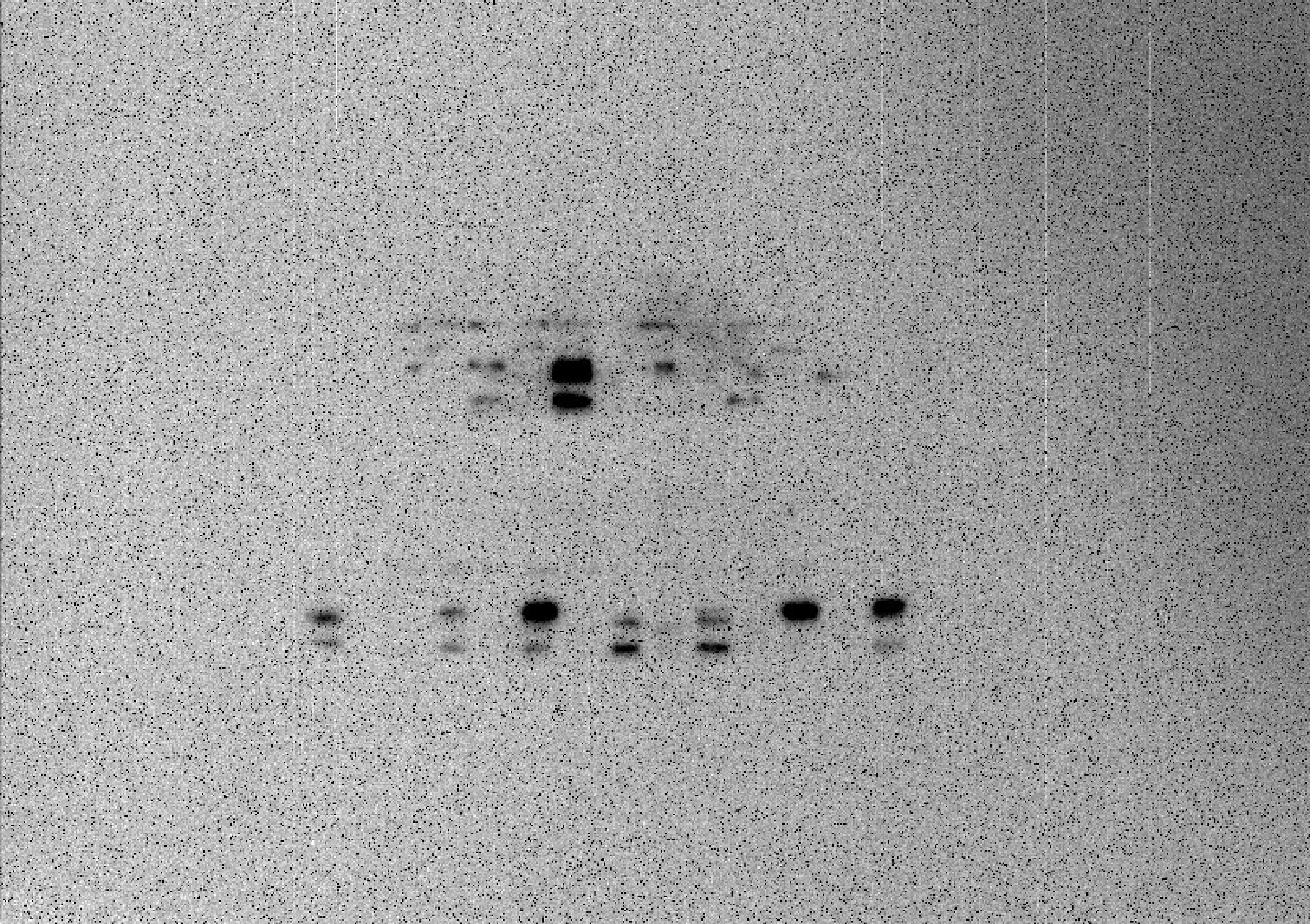

Fig.1-B_Myc_set1&set2_marker

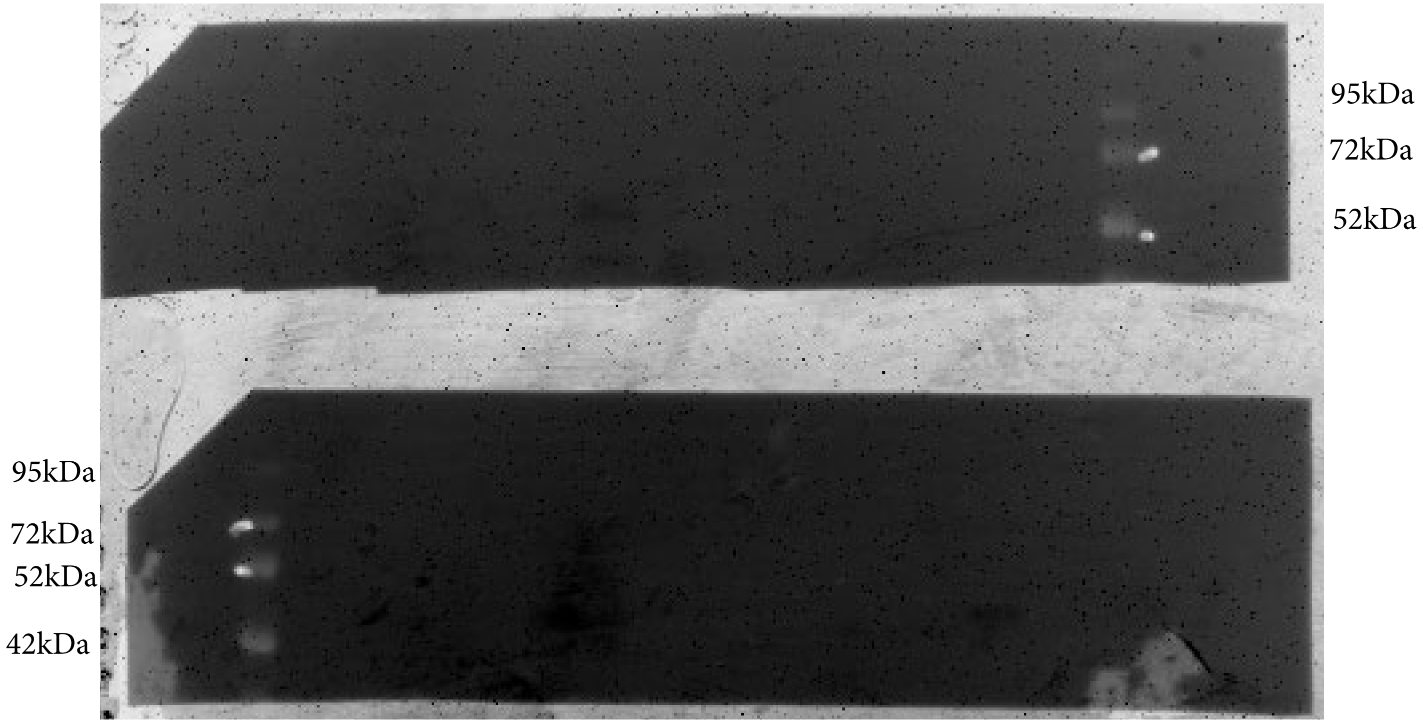

Fig.1-B_Myc_set3&set4

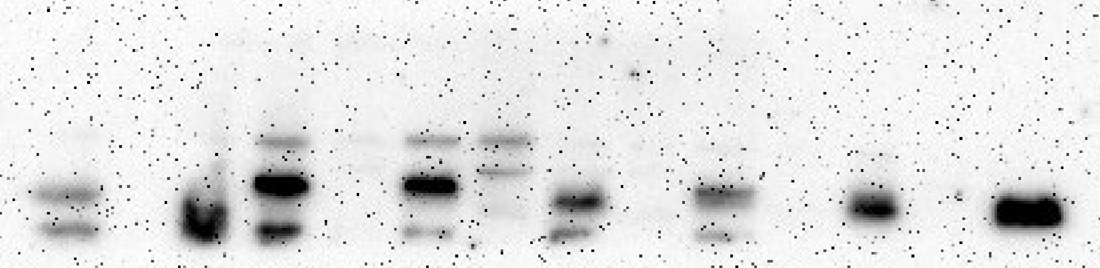

Fig.1-B_Myc_set3&set4_marker

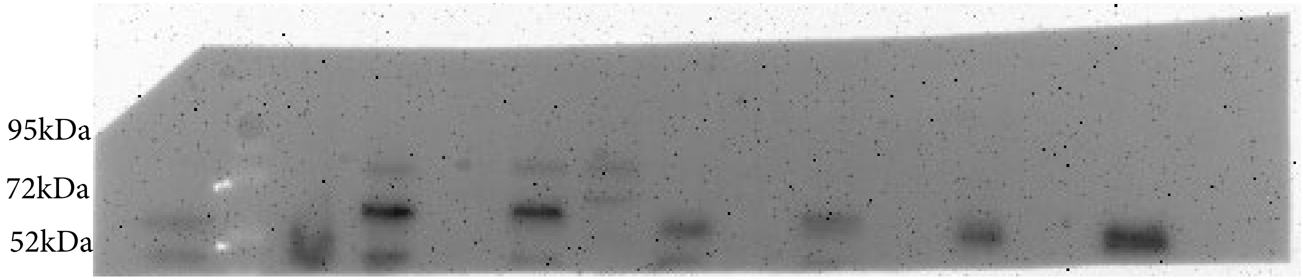

Fig.2-B

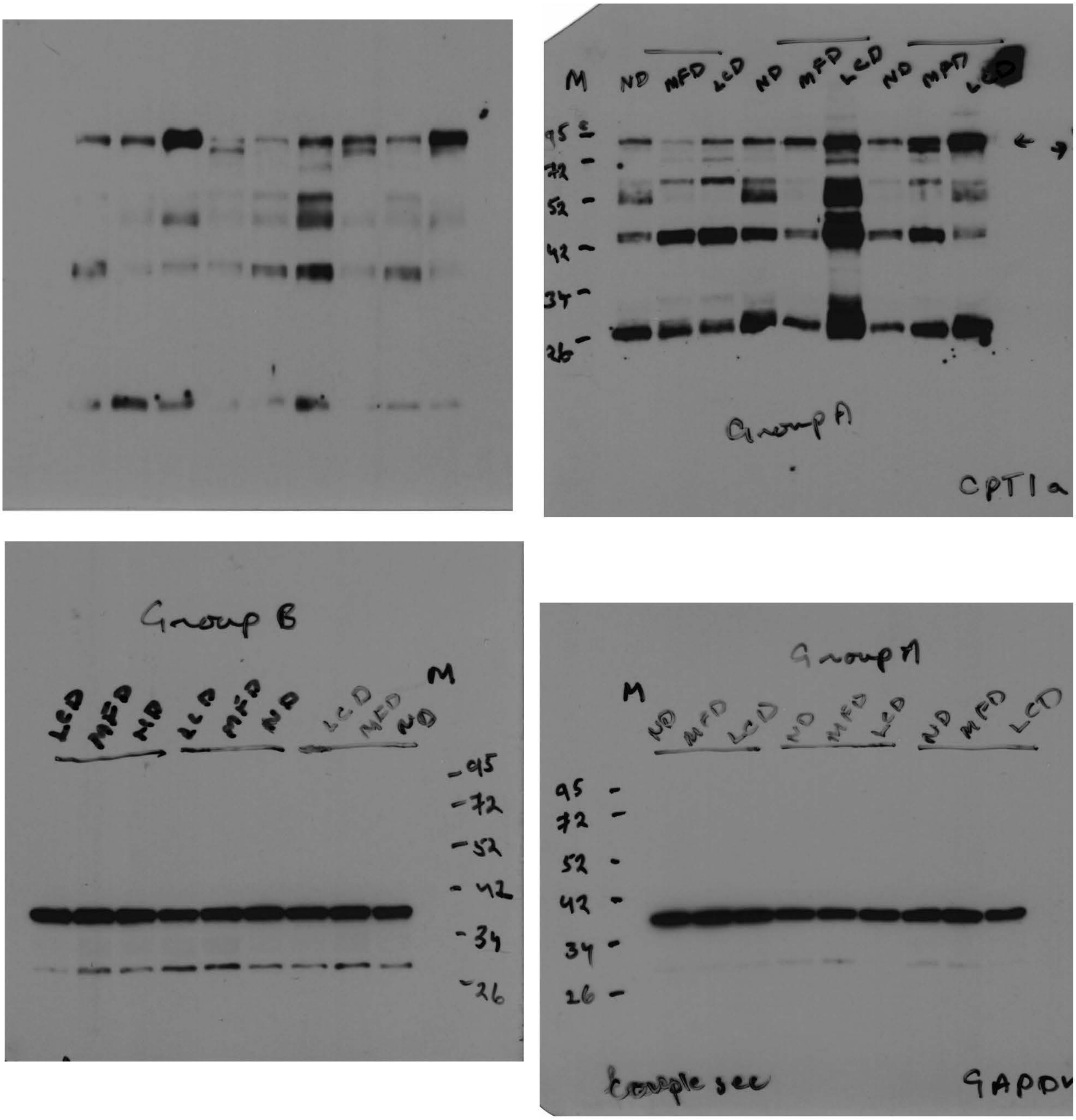

M
ND MFD LCD ND MFD LCD ND MFD LCD
95
72
52
42
34
26
Group A
CPT1a
Group B
LCD MFD ND LCD MFD ND LCD MFD ND
M
95
72
52
42
34
26
Group A
M
ND MFD LCD ND MFD LCD ND MFD LCD
95
72
52
42
34
26

Fig.2-D_GAPDH_set1&set2

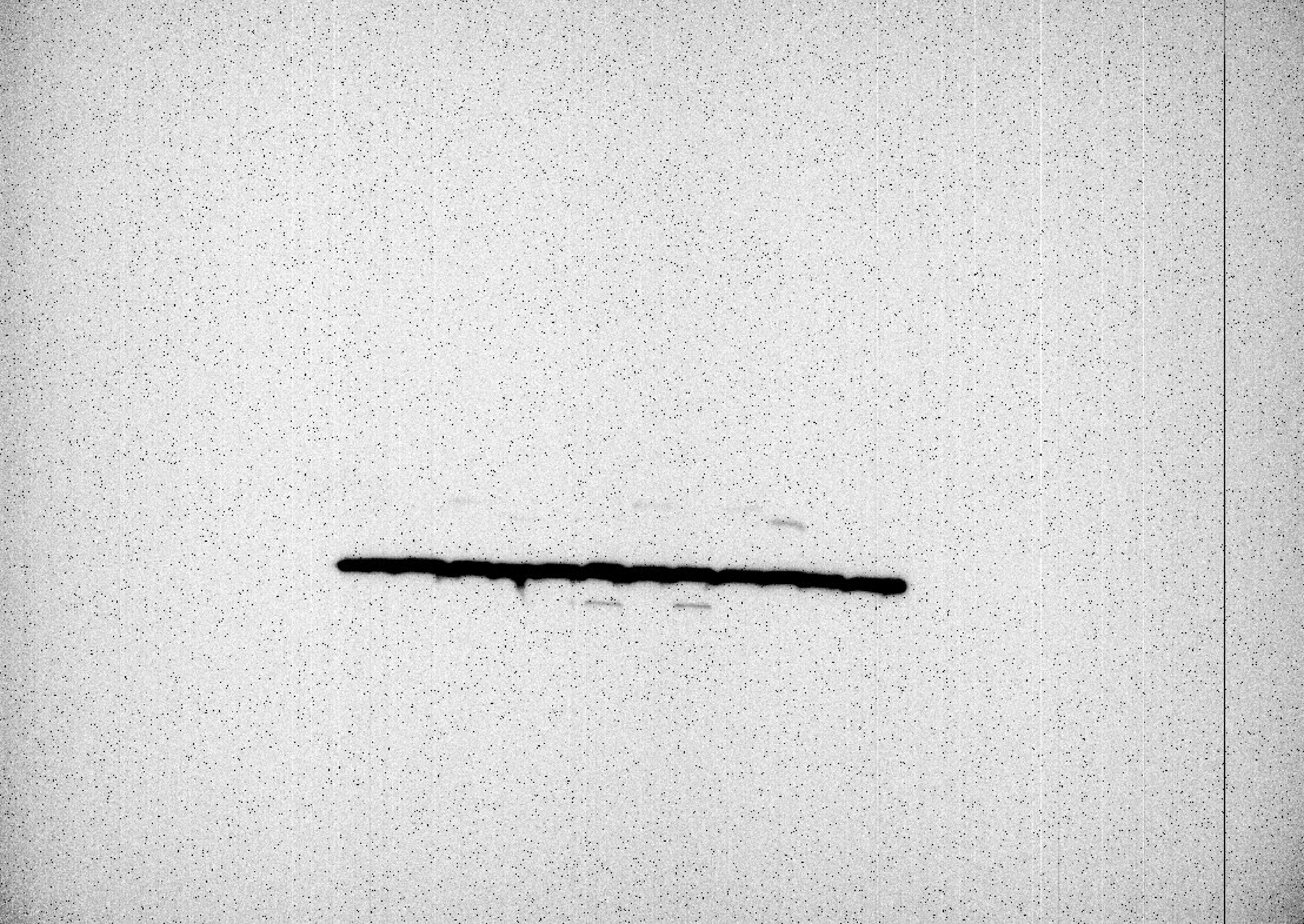

Fig.2-D_GAPDH_set1&set2_marker

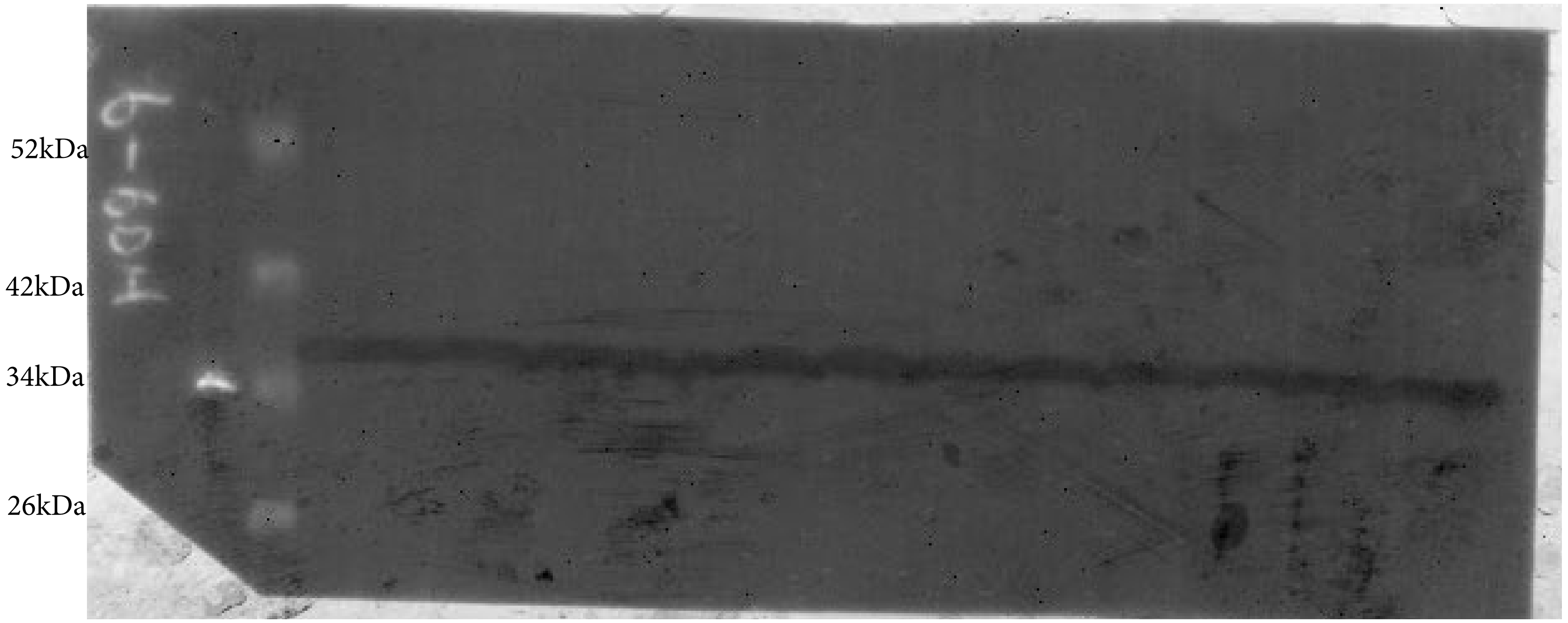

Fig.2-D_GAPDH_set3&set4

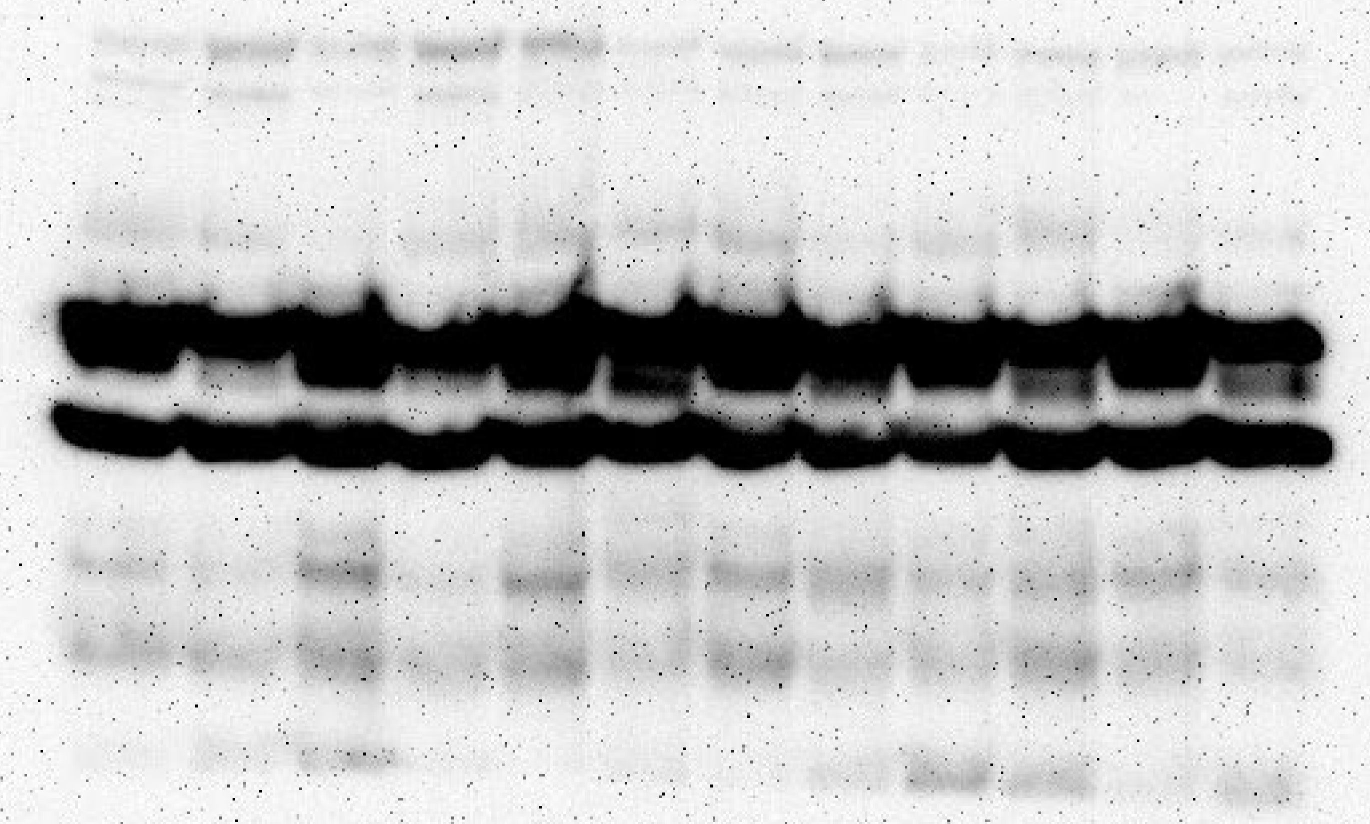

Fig.2-D_GAPDH_set3&set4_marker

52kDa

42kDa

34kDa

26kDa

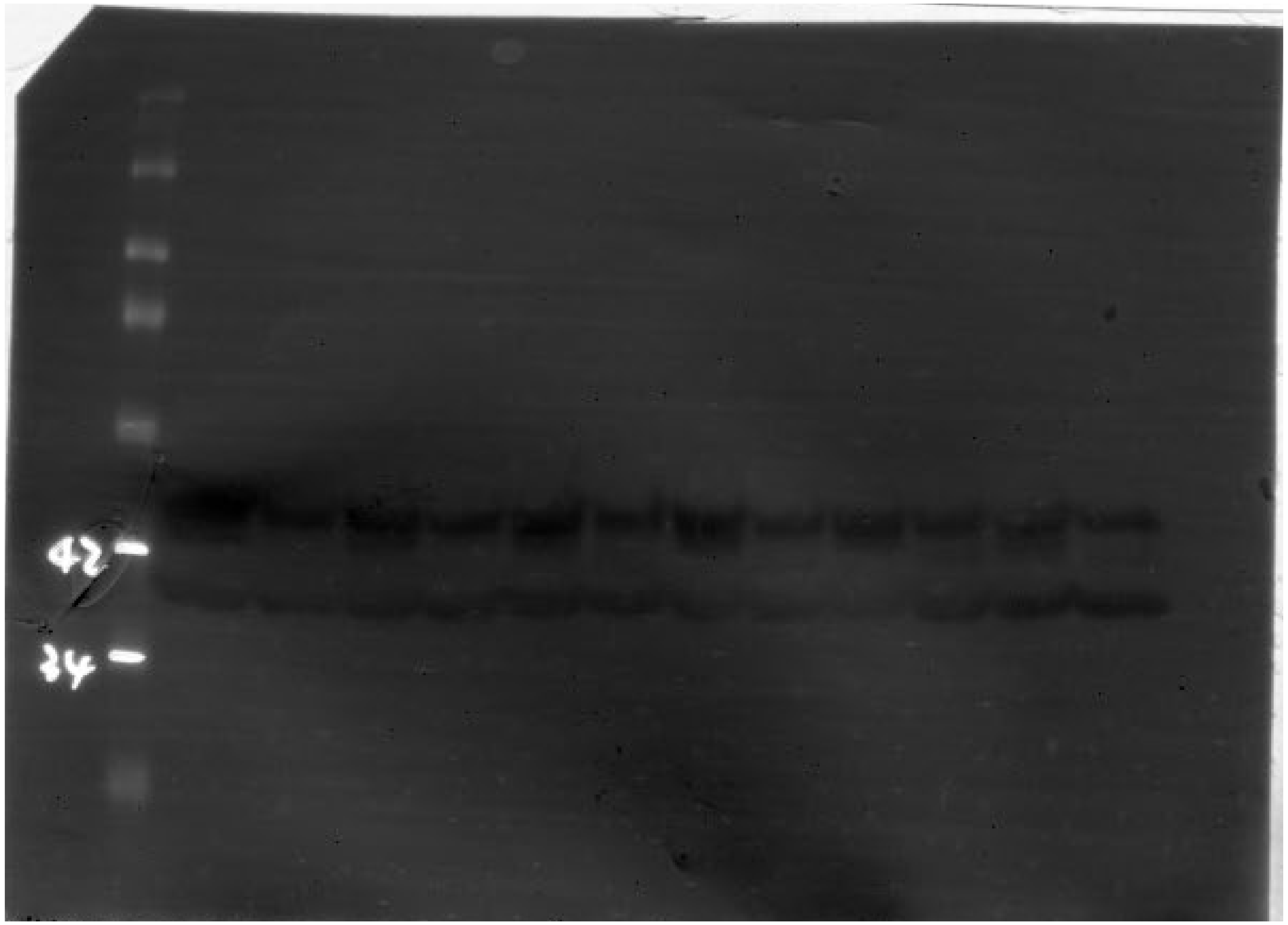

Fig.2-D_PDH_set1&set2

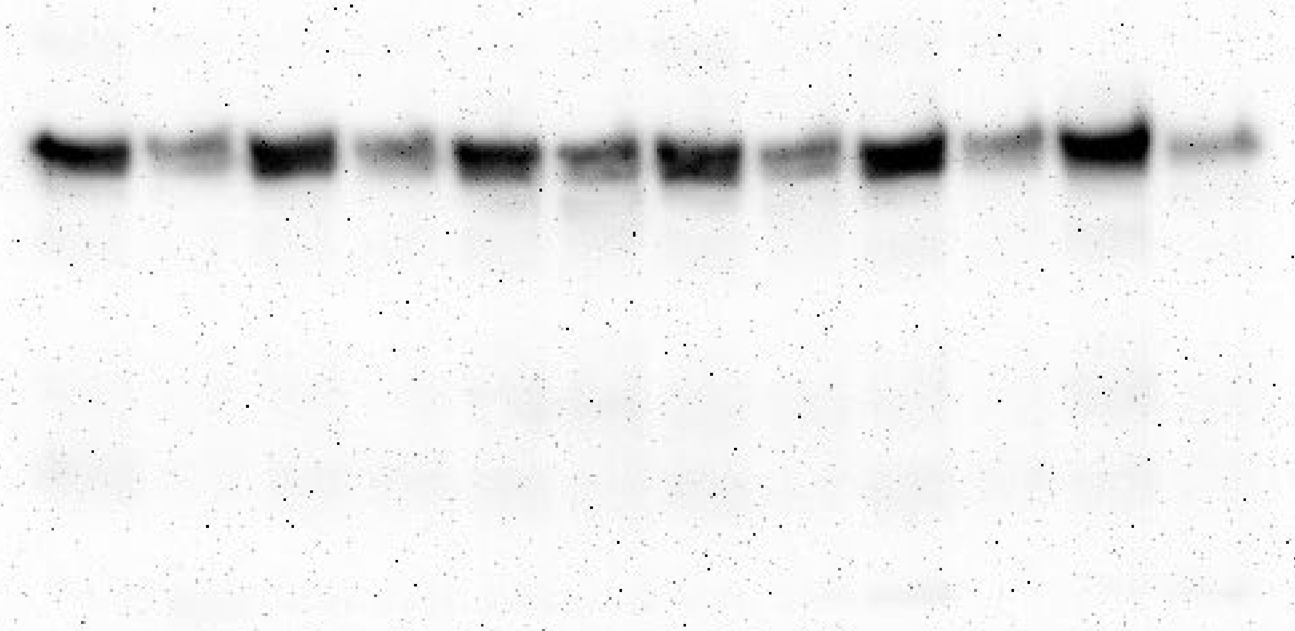

Fig.2-D_PDH_set1&set2_marker

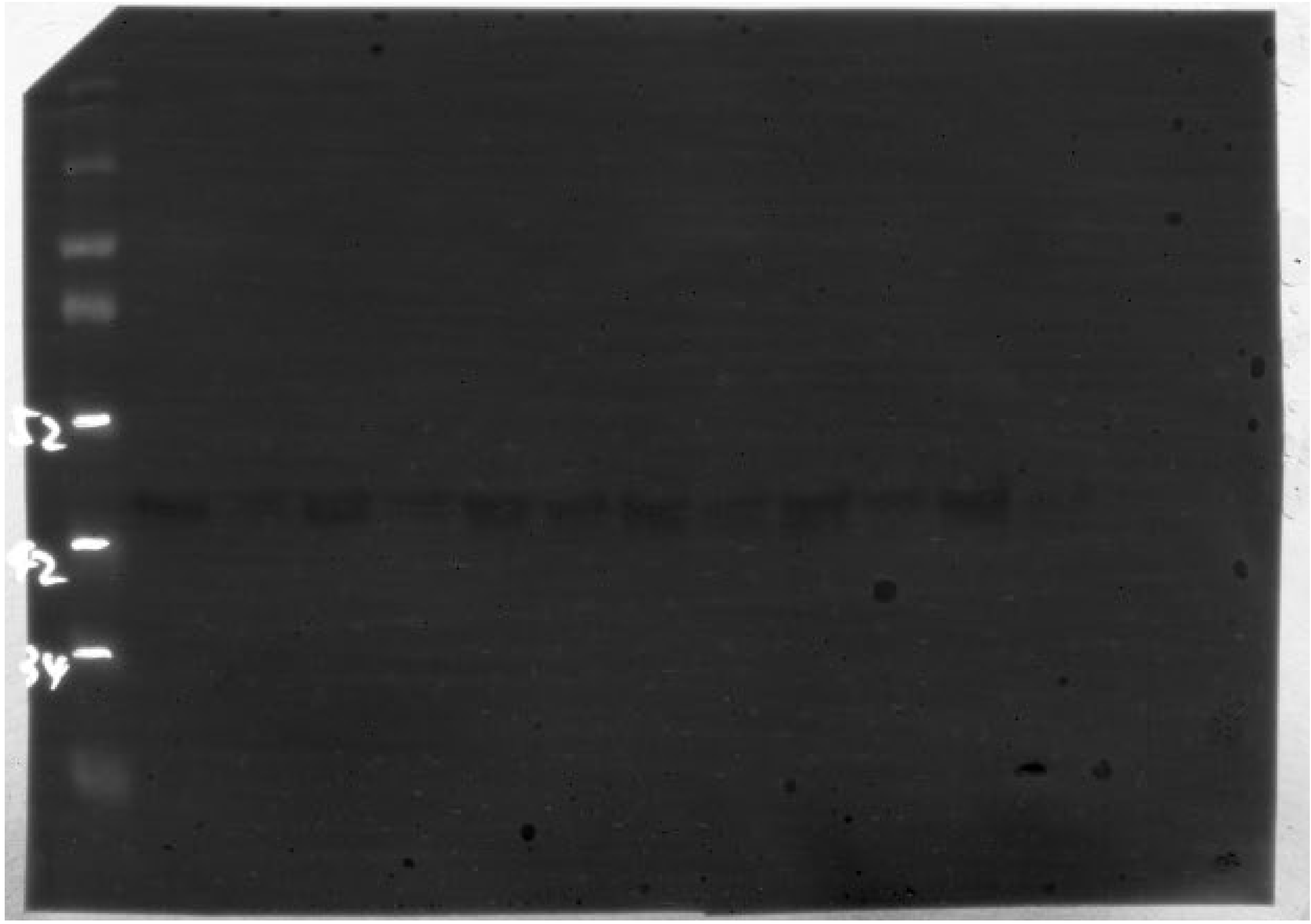

52kDa
42kDa
34kDa
26kDa

Fig.2-D_PDH_set3&set4

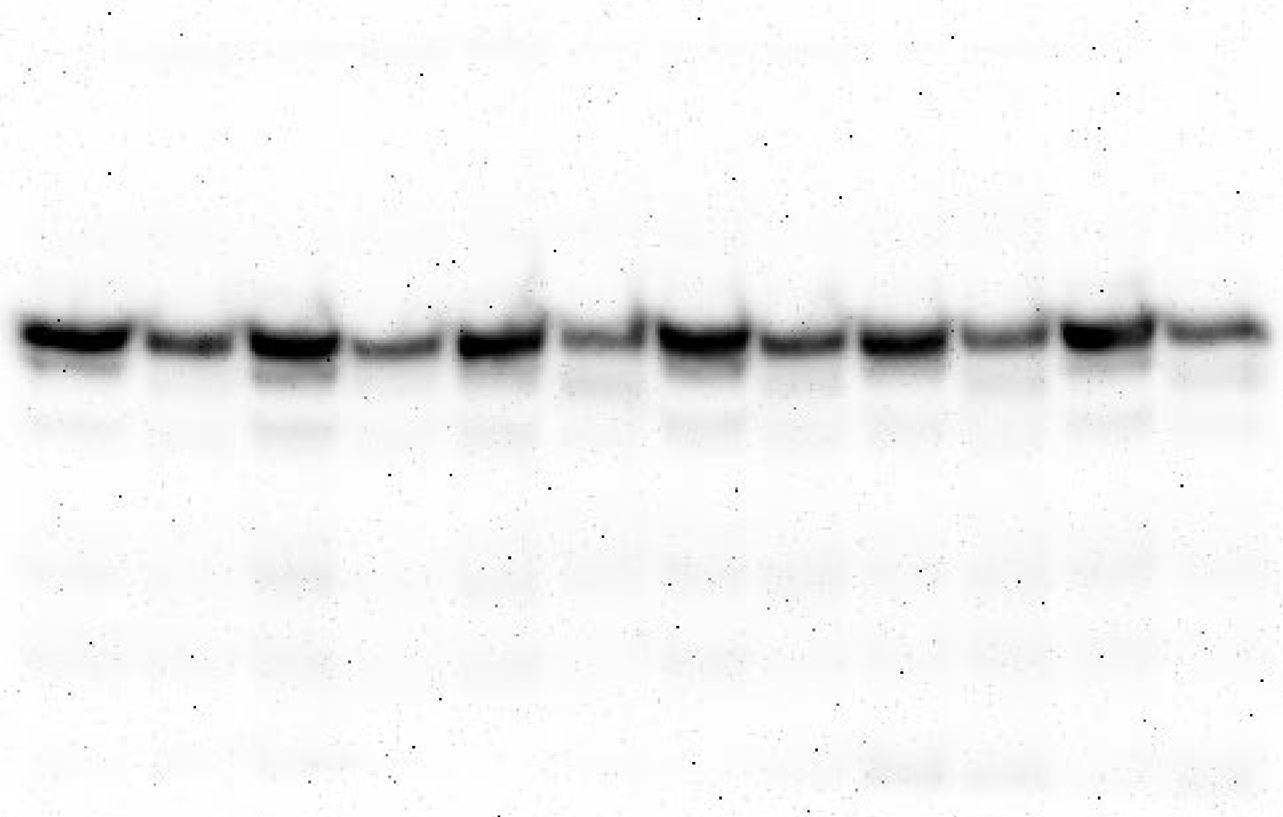

Fig.2-D_PDH_set3&set4_marker

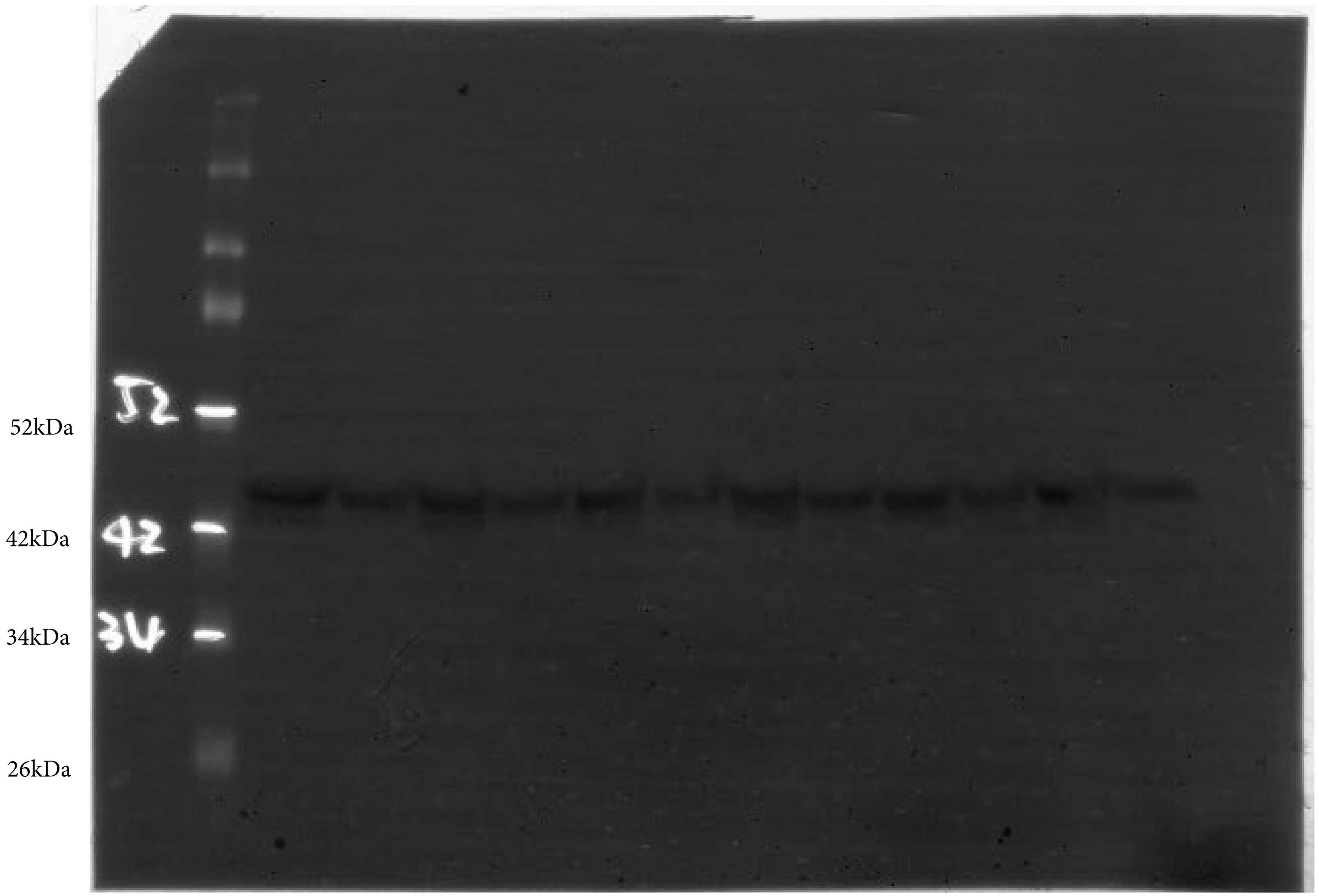

Fig.2-D_pPDH_set1&set2

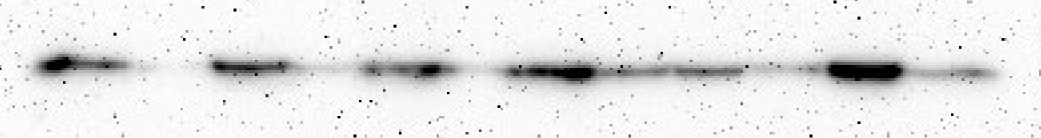

Fig.2-D_pPDH_set1&set2_marker

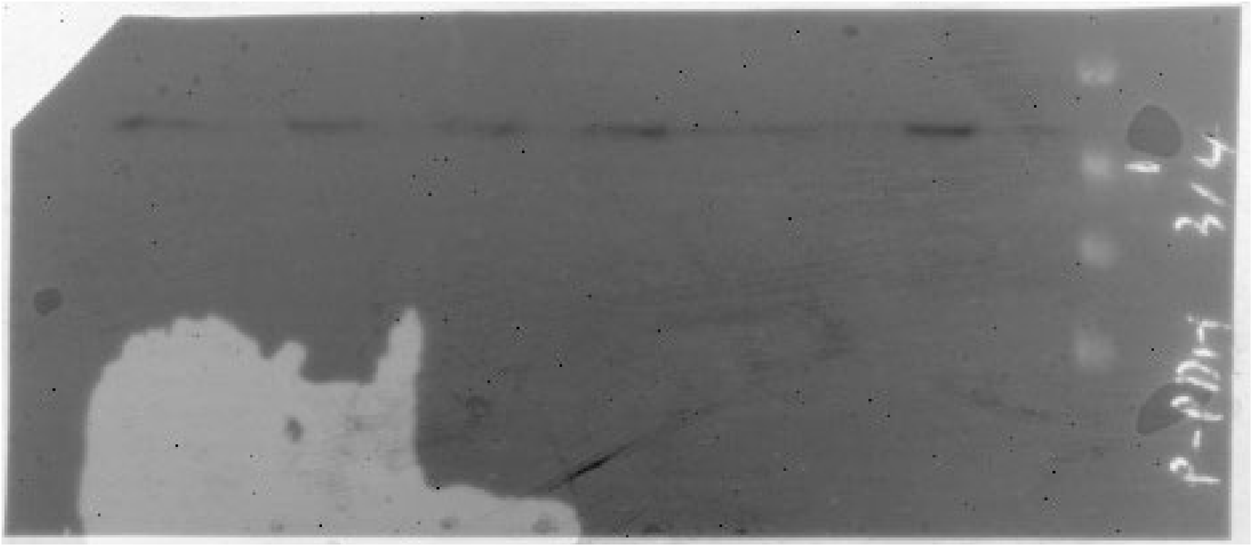

Fig.2-D_pPDH_set3&set4

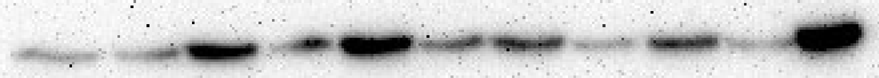

Fig.2-D_pPDH_set3&set4_marker

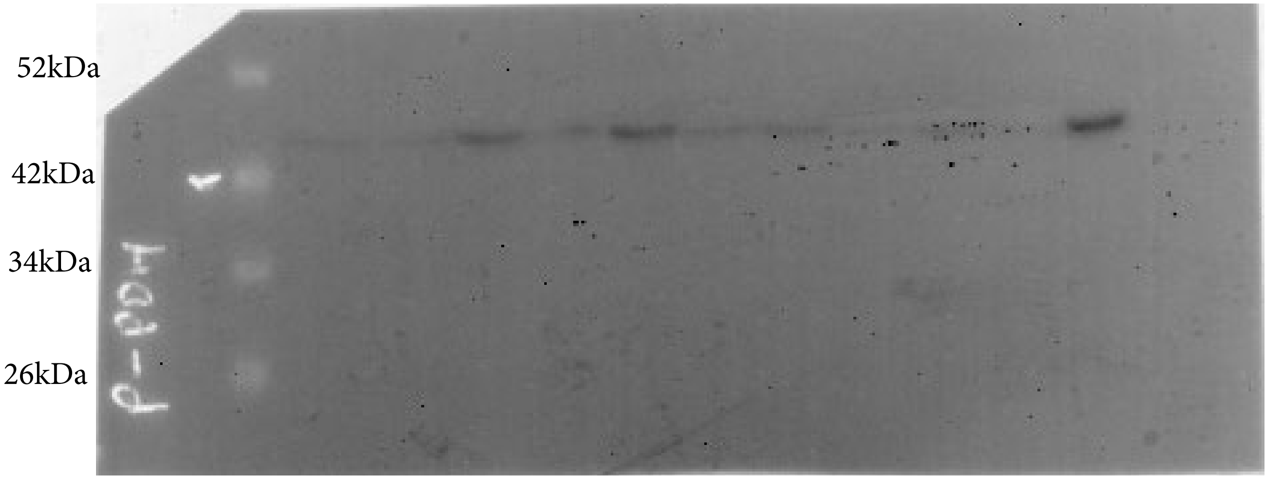

Fig.2-F_GAPDH_set1&set2

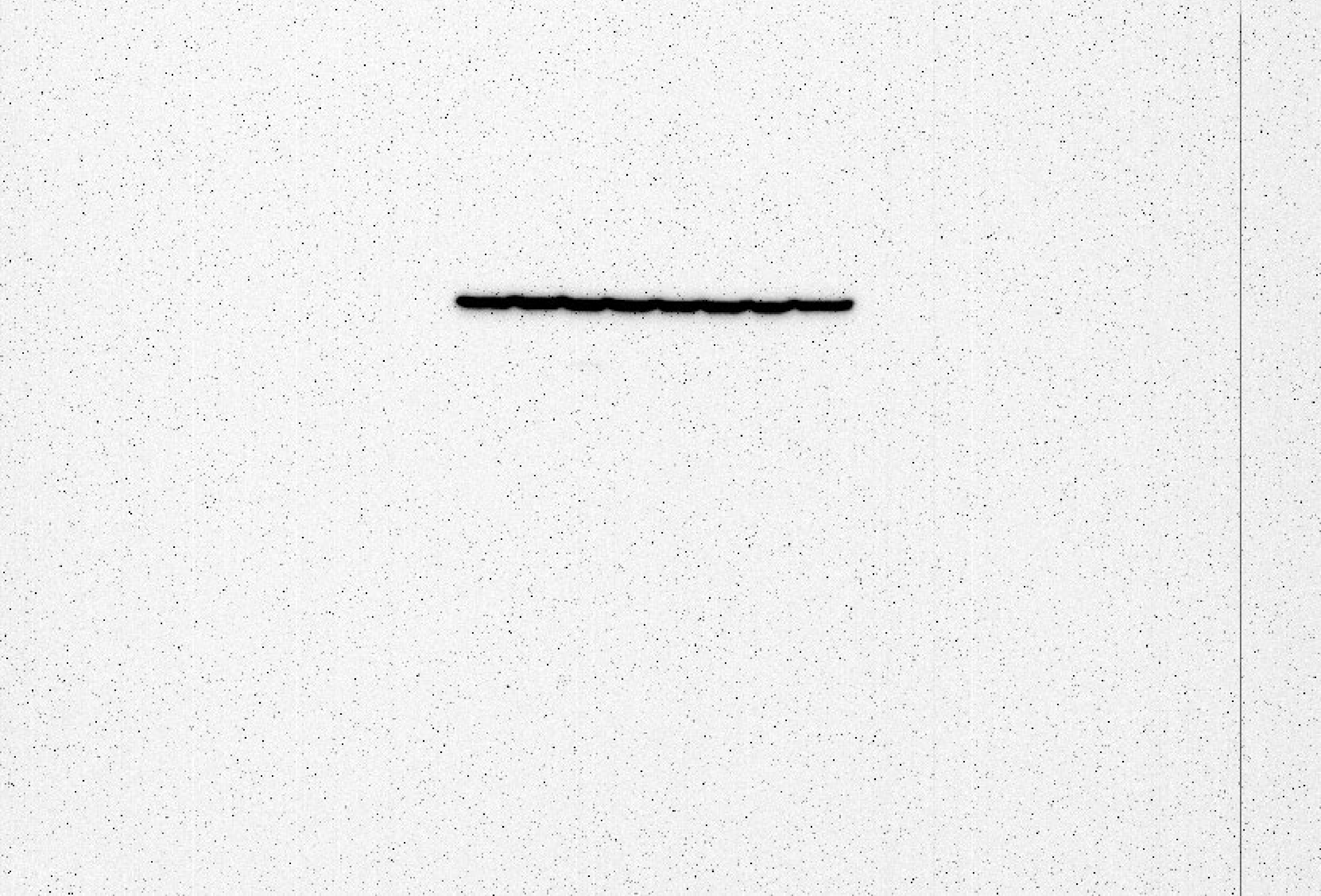

Fig.2-F_GAPDH_set1&set2_marker

42kDa
34kDa
26kDa

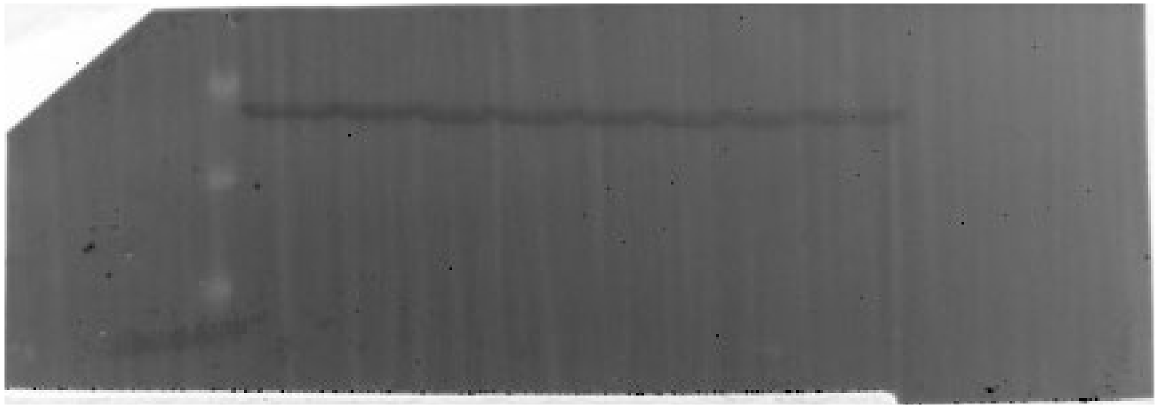

Fig.2-F_GAPDH_set3&set4

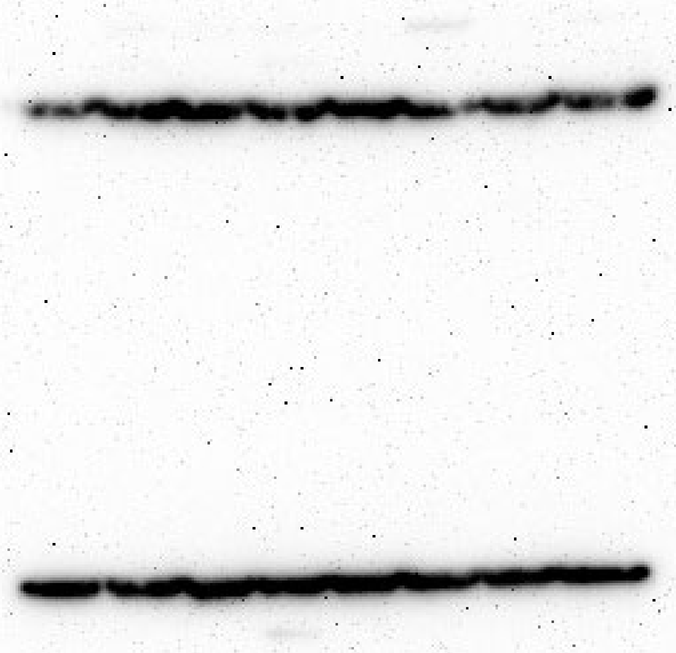

Fig.2-F_GAPDH_set3&set4_marker

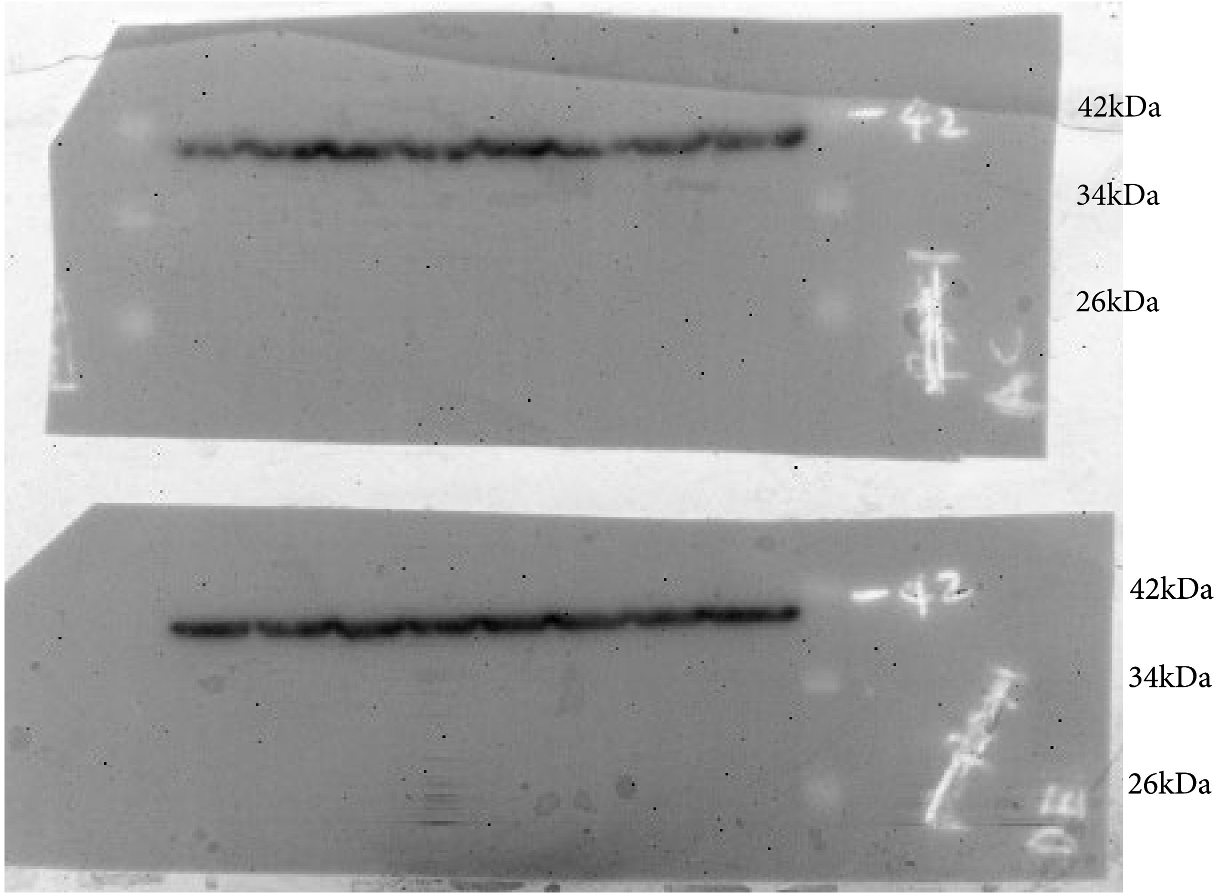

Fig.2-F_Pkm1_set1&set2

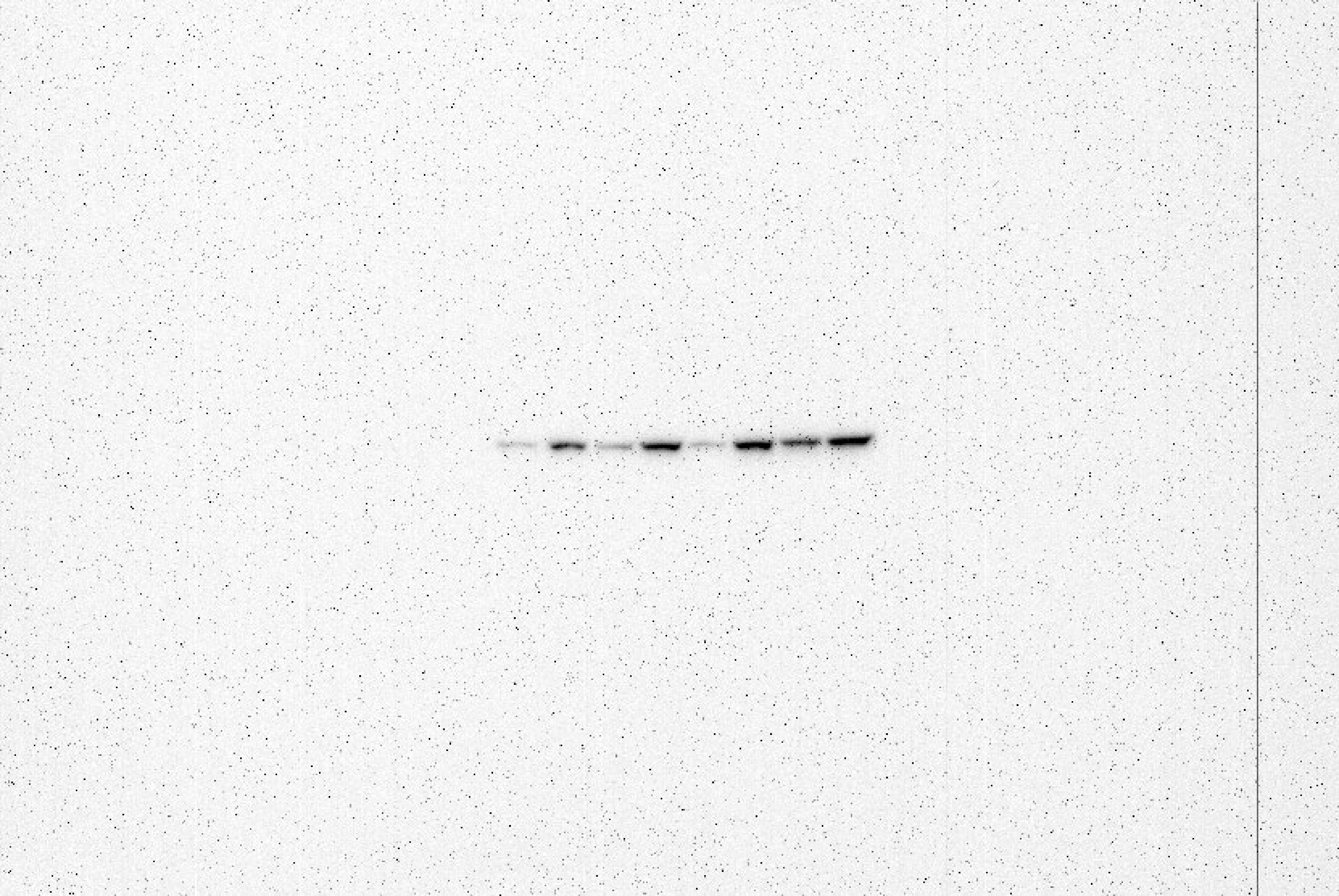

Fig.2-F_Pkm1_set1&set2_marker

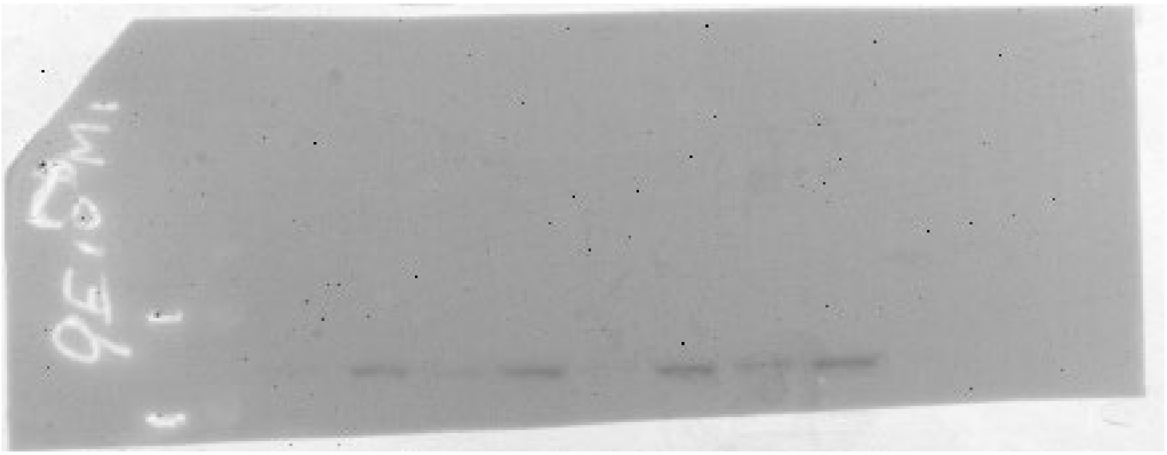

Fig.2-F_Pkm1_set3&set4

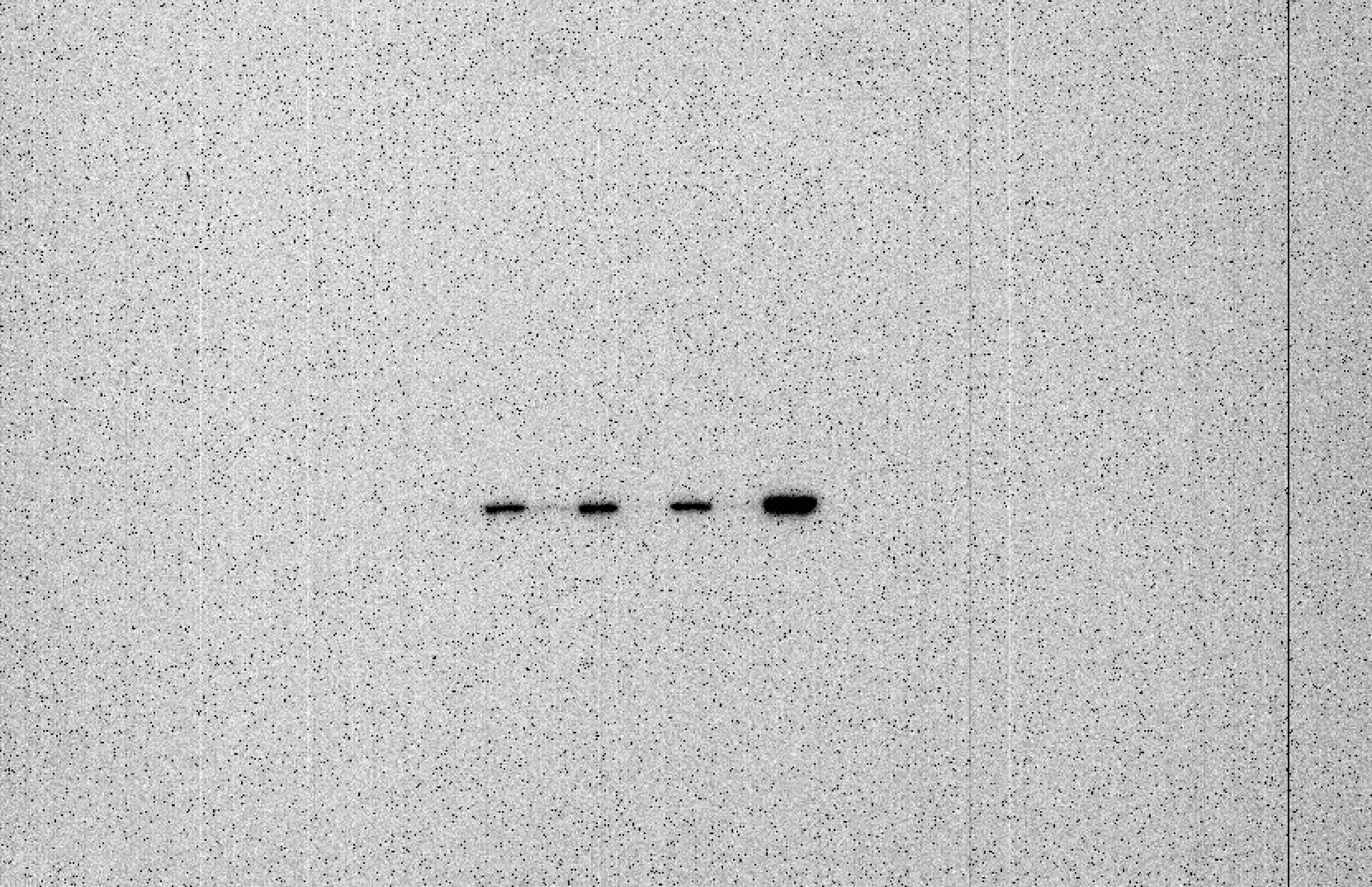

Fig.2-F_Pkm1_set3&set4_marker

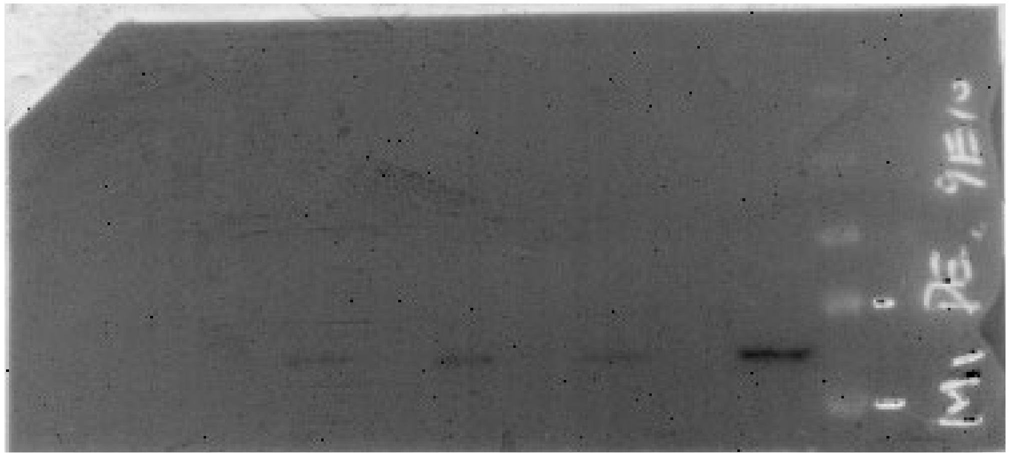

Fig.2-F_Pkm2_set1&set2

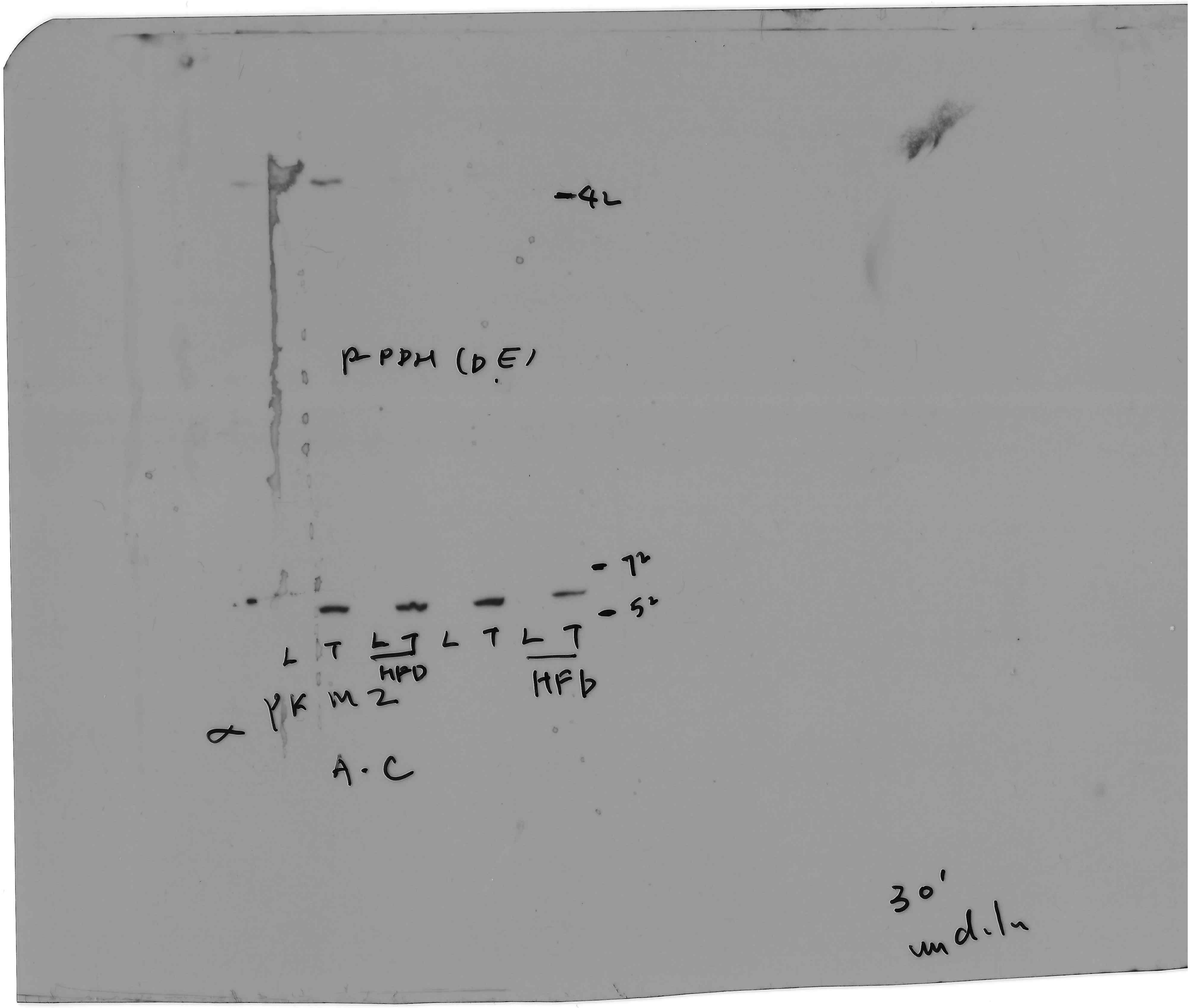

Fig.2-F_Pkm2_set3&set4

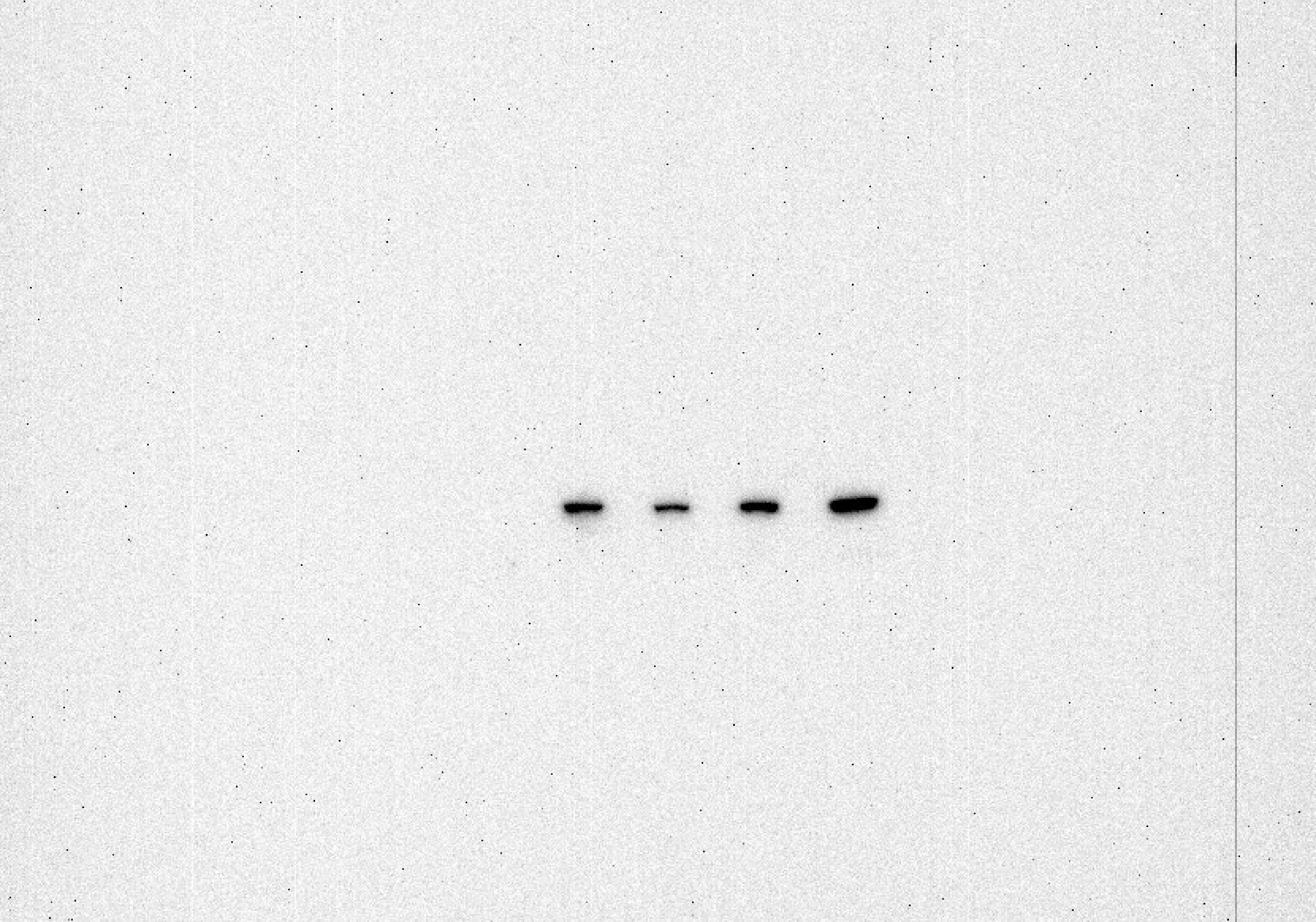

Fig.2-F_Pkm2_set3&set4_marker

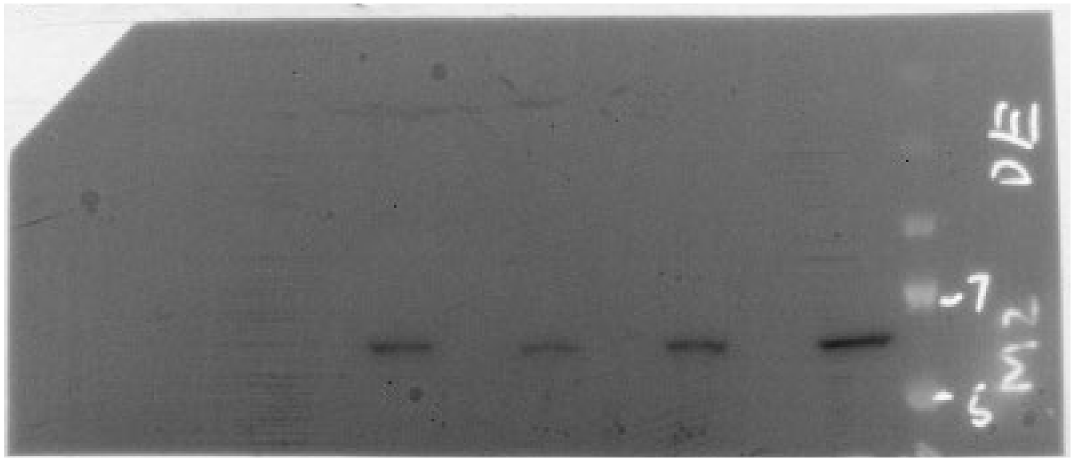

95kDa

72kDa

52kDa

Fig.2-I_GLUT1_set3&set4

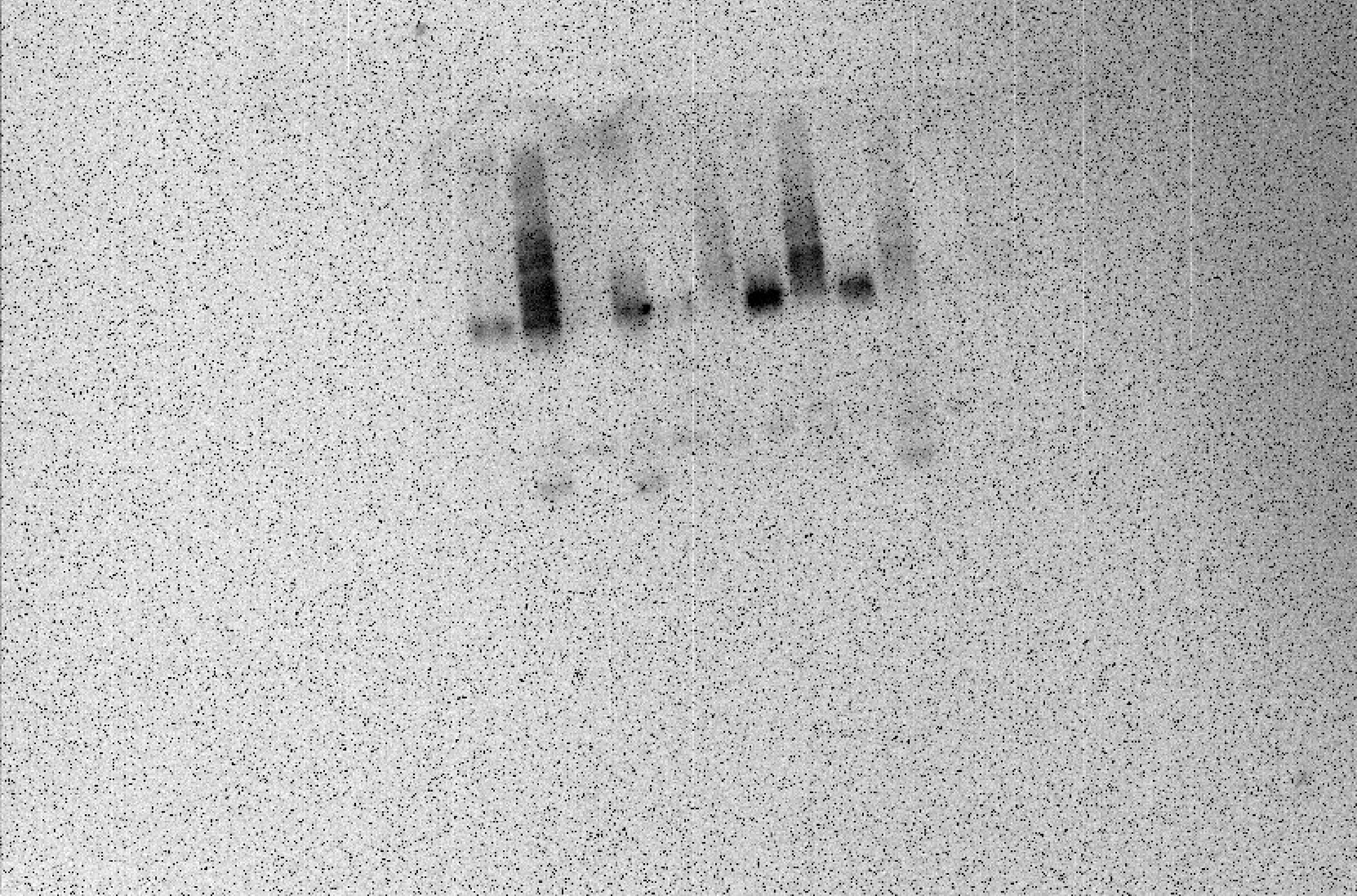

Fig.2-I_GLUT1_set3&set4_marker

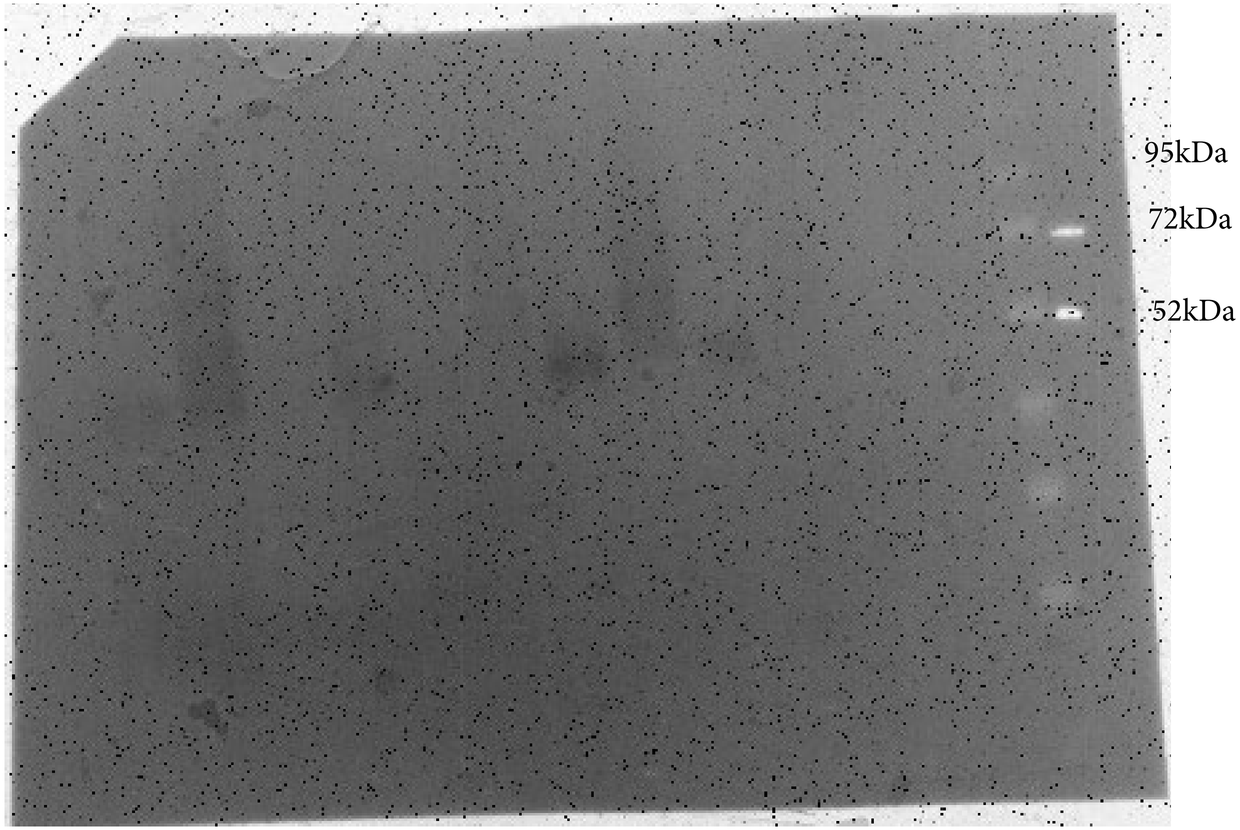

Fig.S3-C_ChREBP

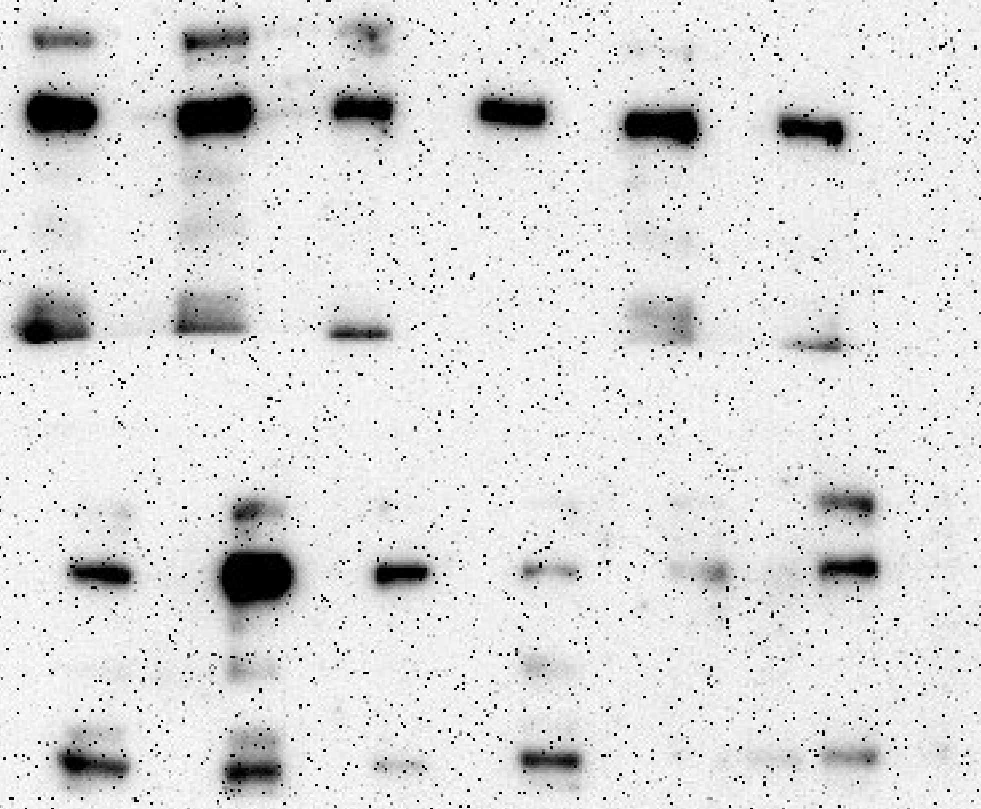

Fig.S3-C_ChREBP_marker

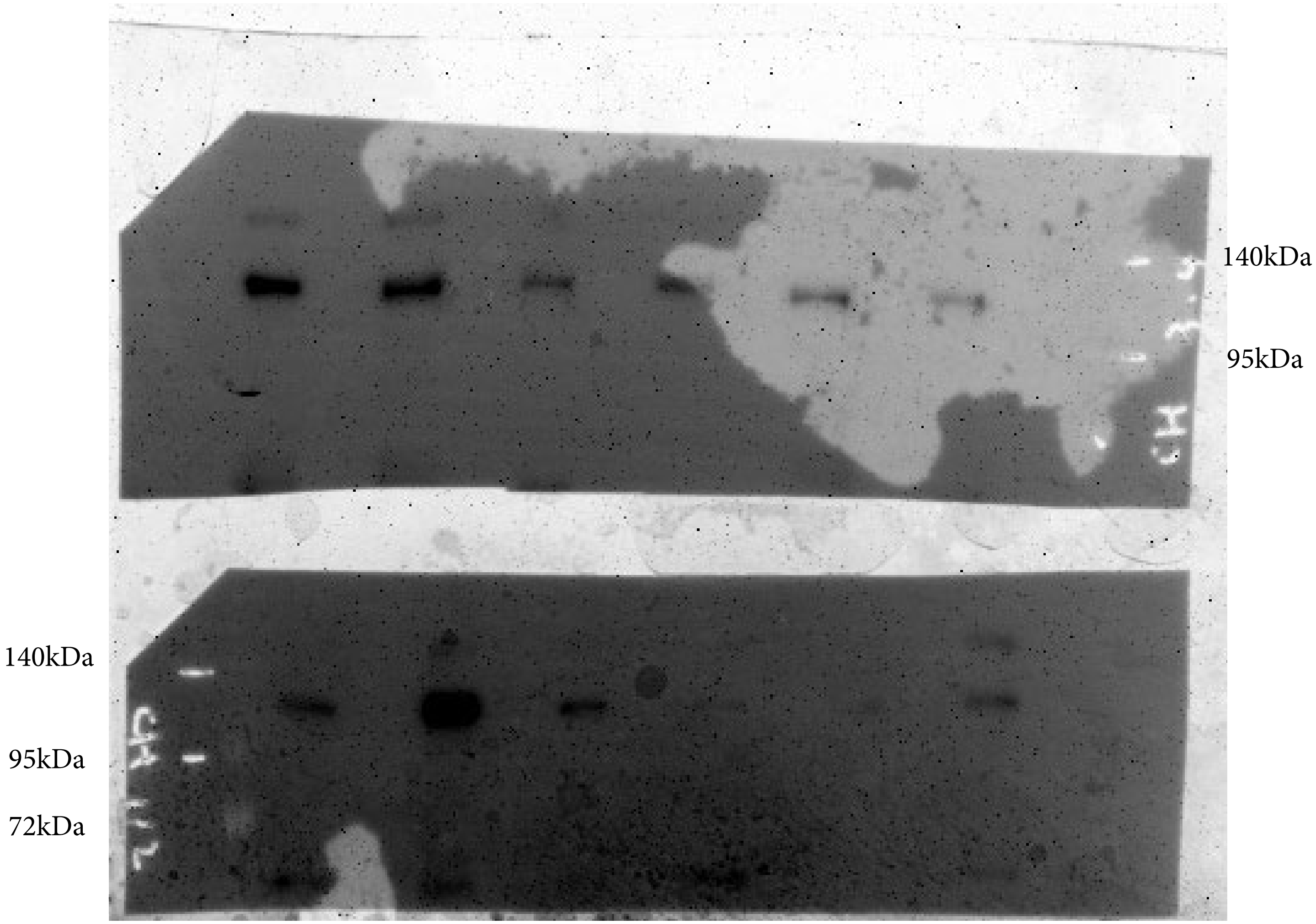

Fig.S3-C_GAPDH_set1&set2

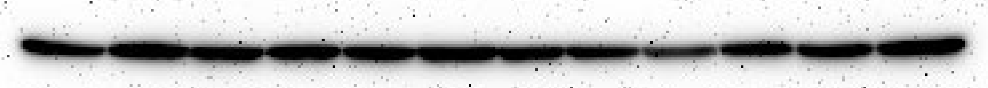

Fig.S3-C_GAPDH_set1&set2_marker

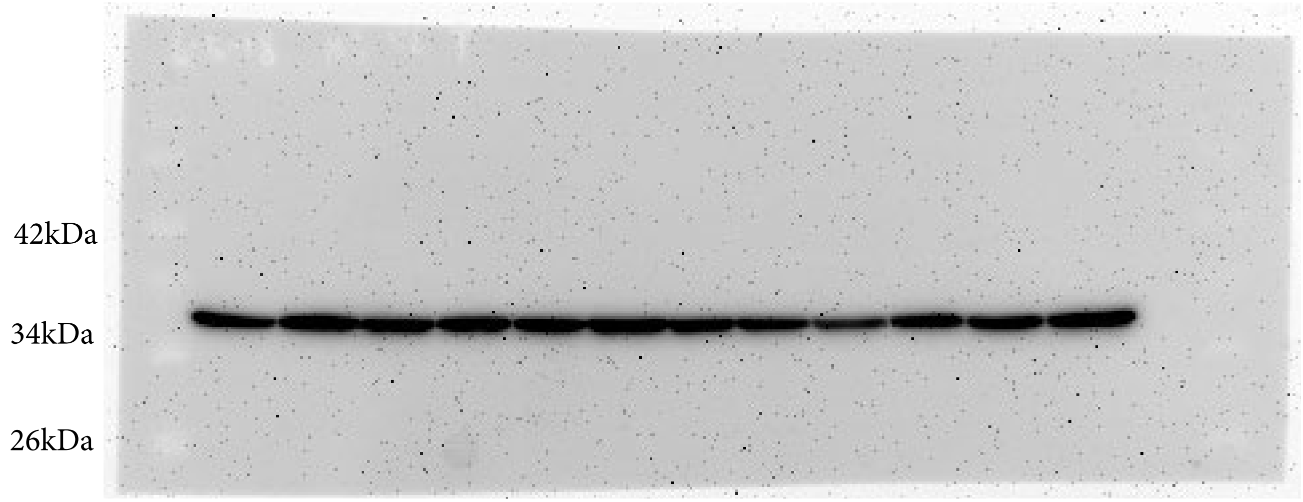

Fig.S3-C_GAPDH_set3&set4

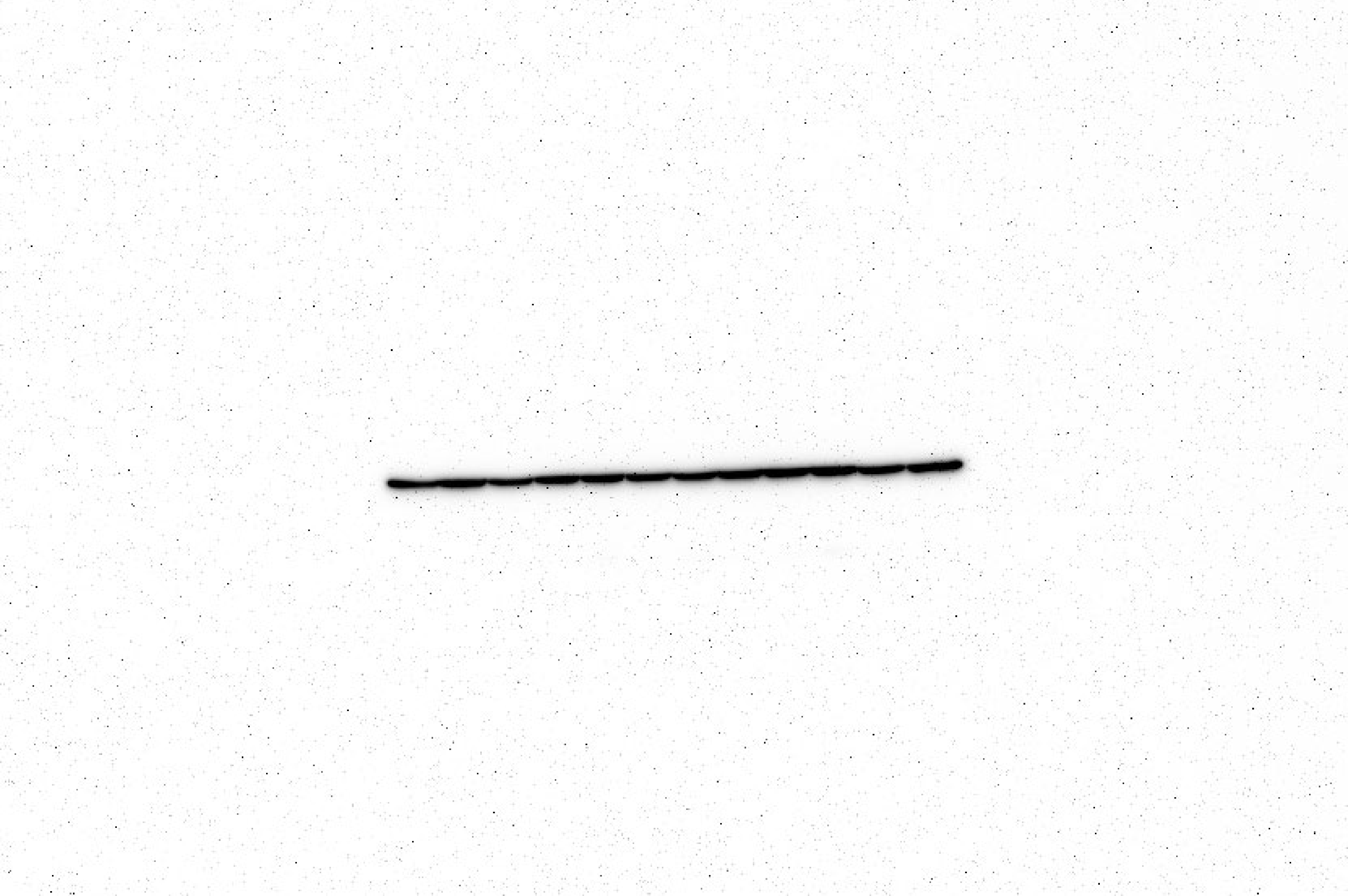

Fig.S3-C_GAPDH_set3&set4_marker

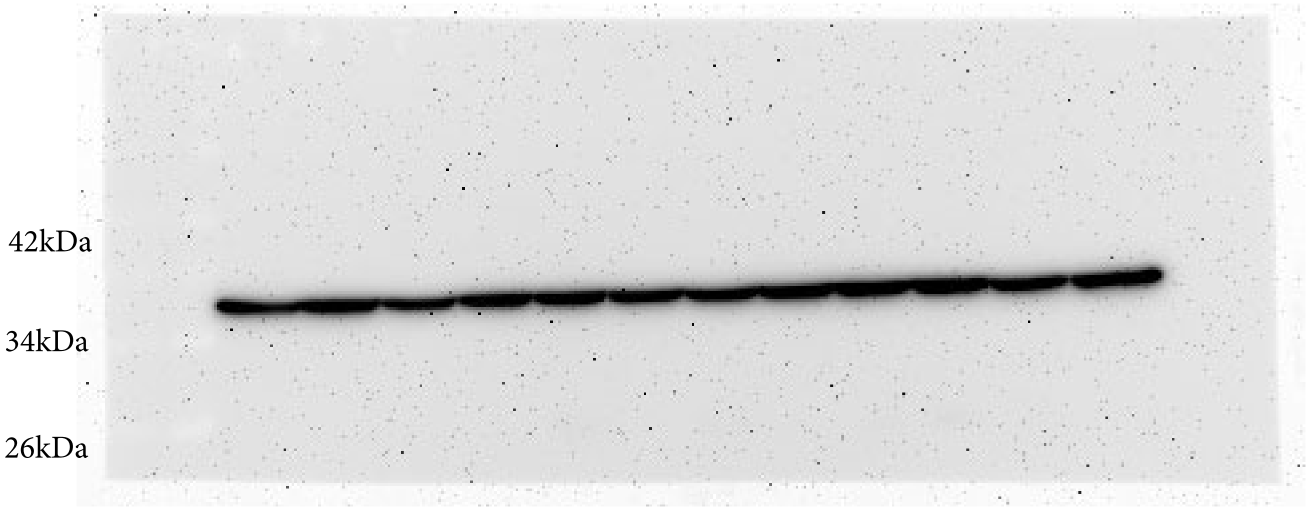

Fig.S3-C_L-Myc_set1&set2

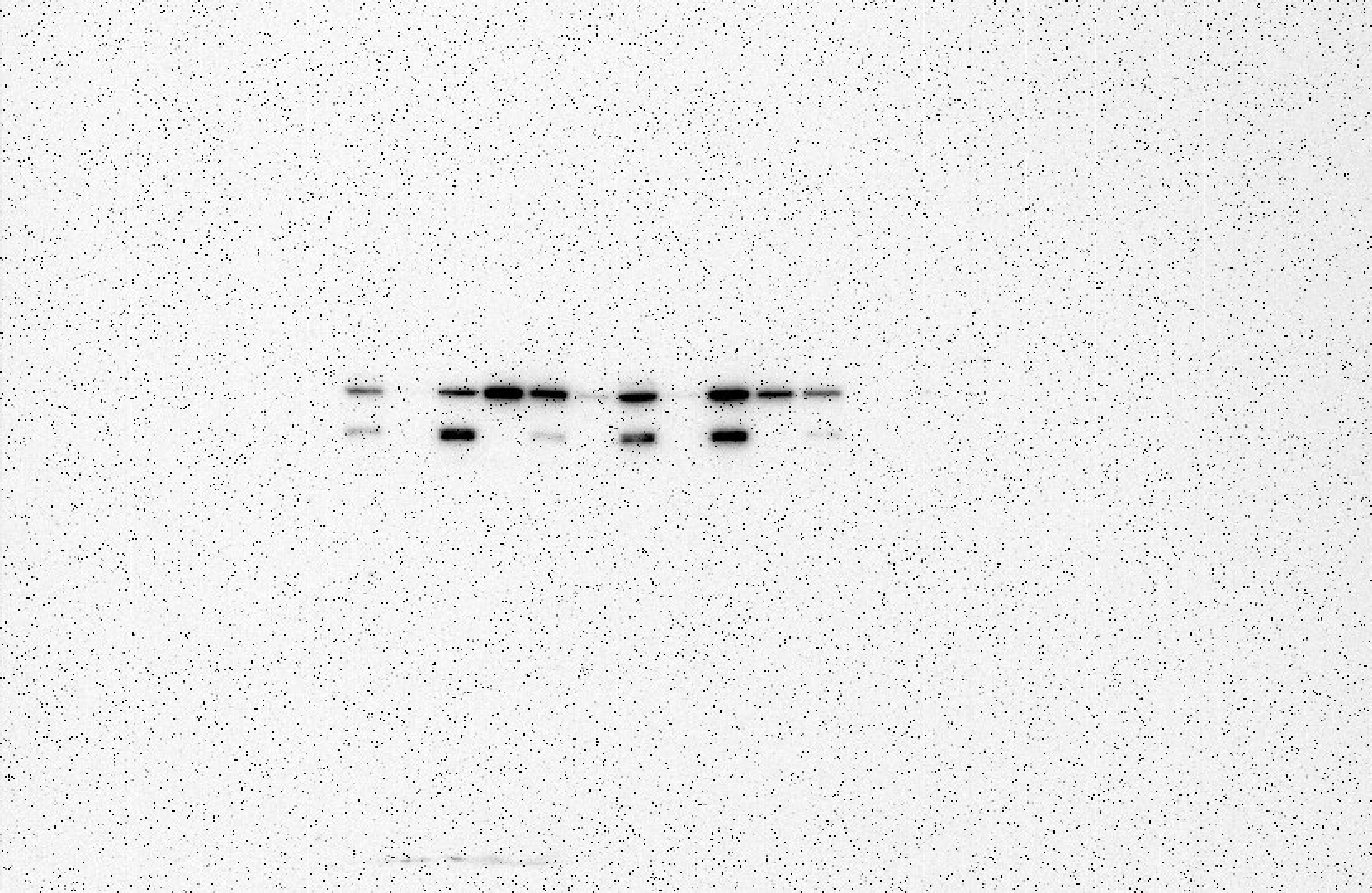

Fig.S3-C_L-Myc_set1&set2_marker

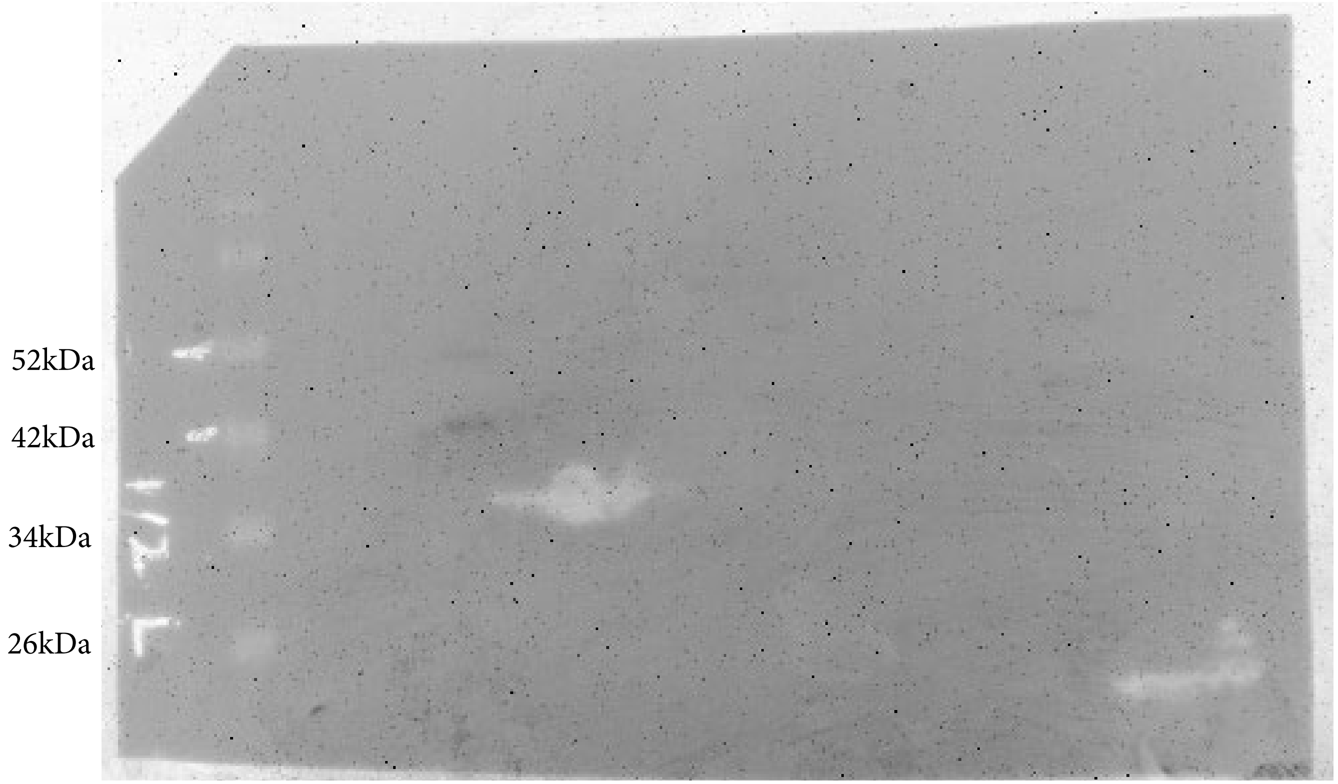

Fig.S3-C_L-Myc_set3&set4

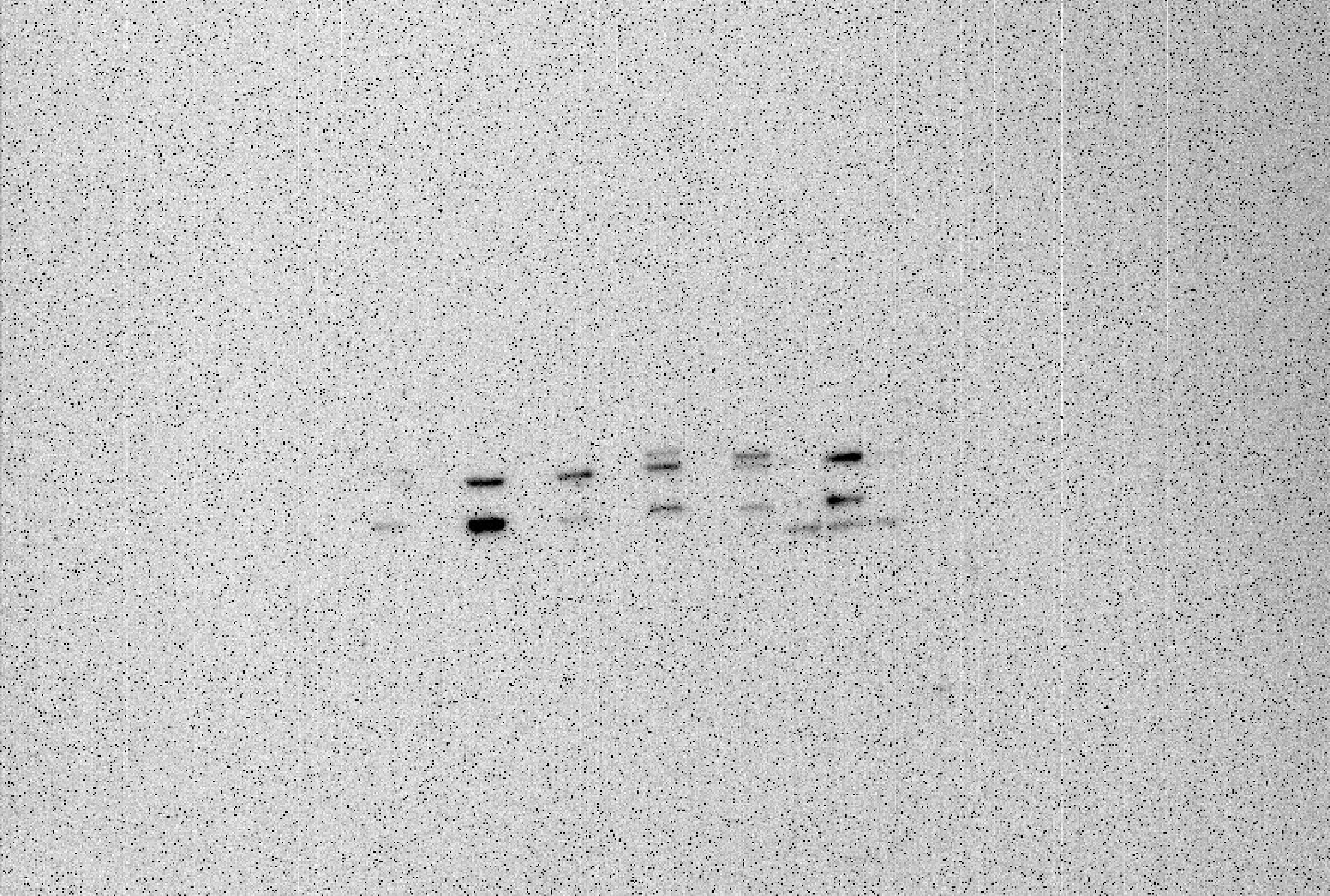

Fig.S3-C_L-Myc_set3&set4_marker

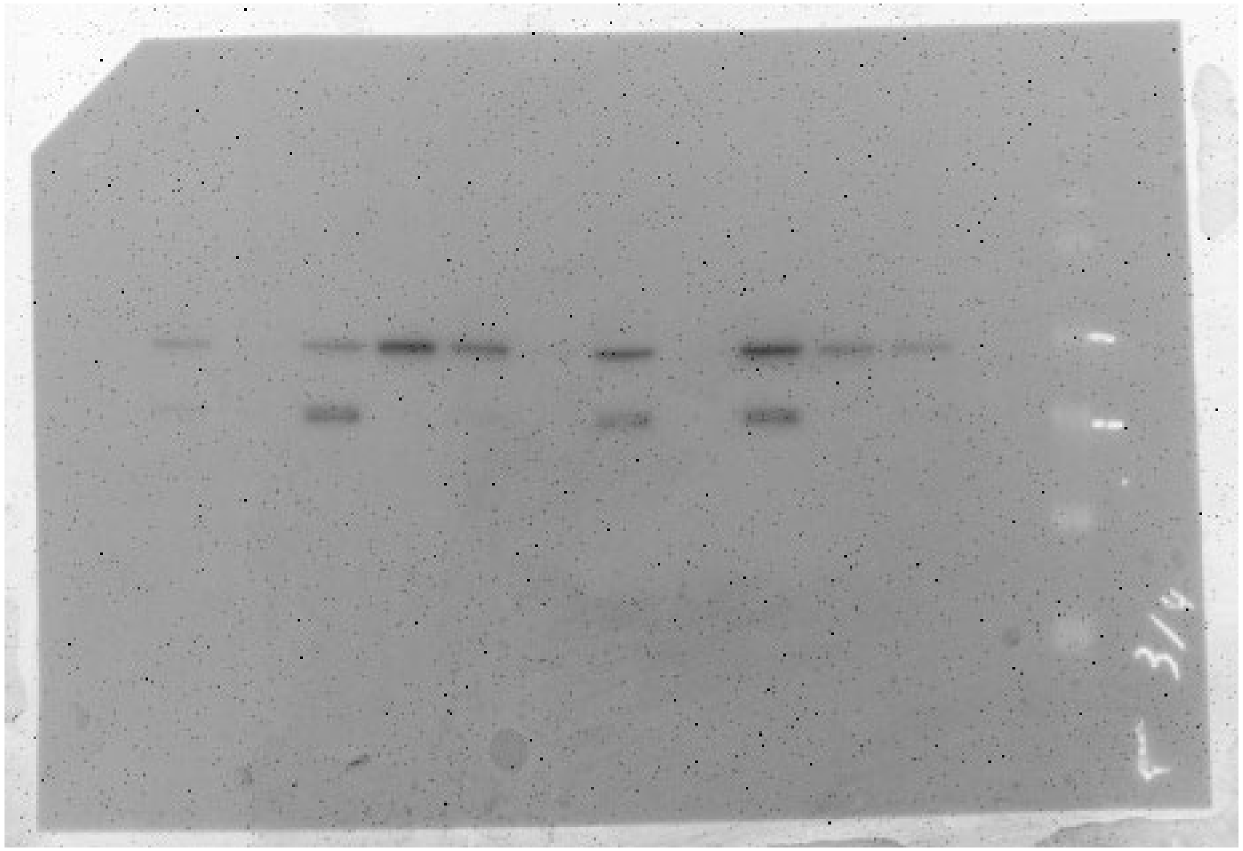

52kDa

42kDa

34kDa

26kDa

Fig.S3-C_MondoA_set1&set2

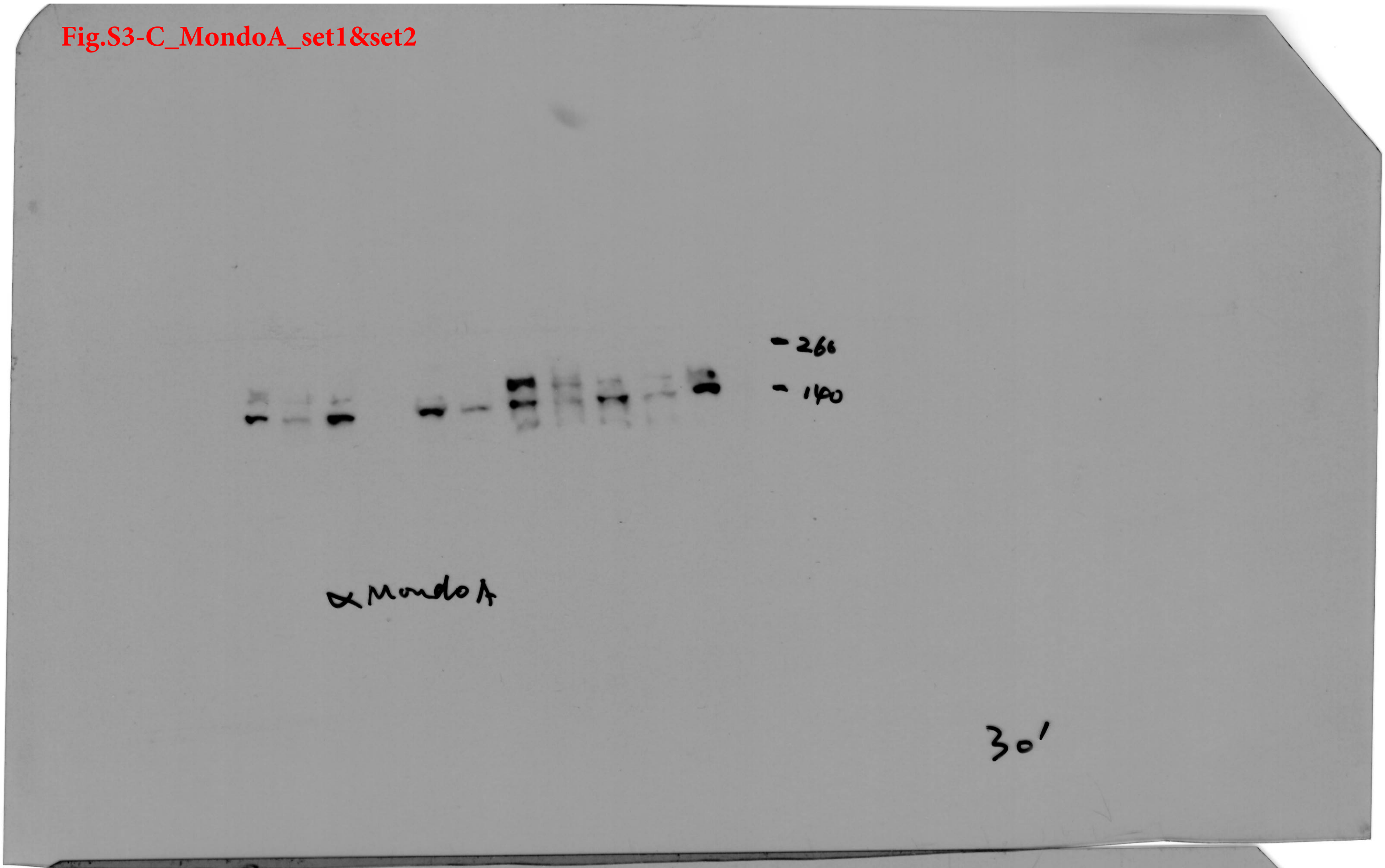

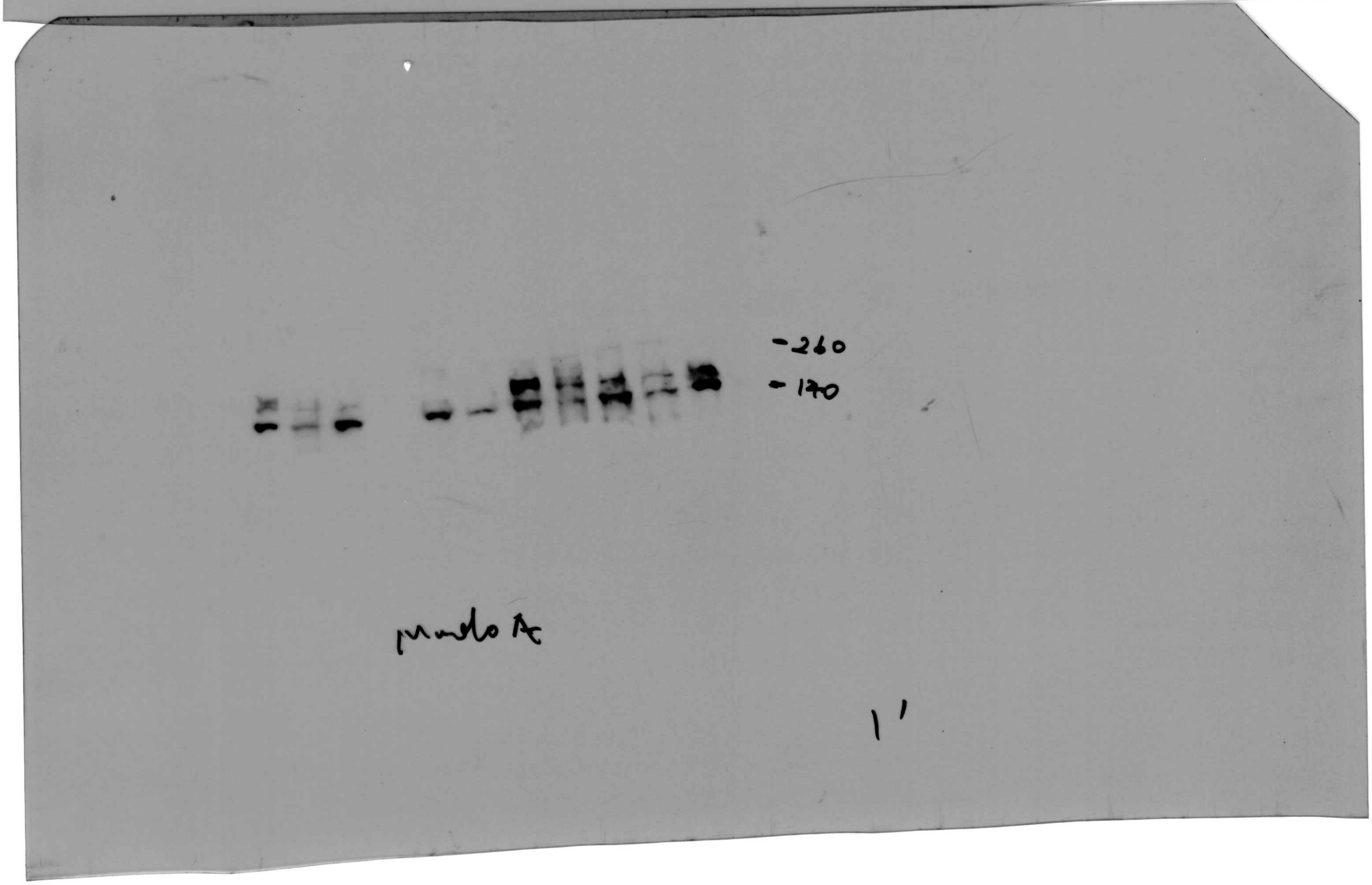

Fig.S3-C_MondoA_set3&set4

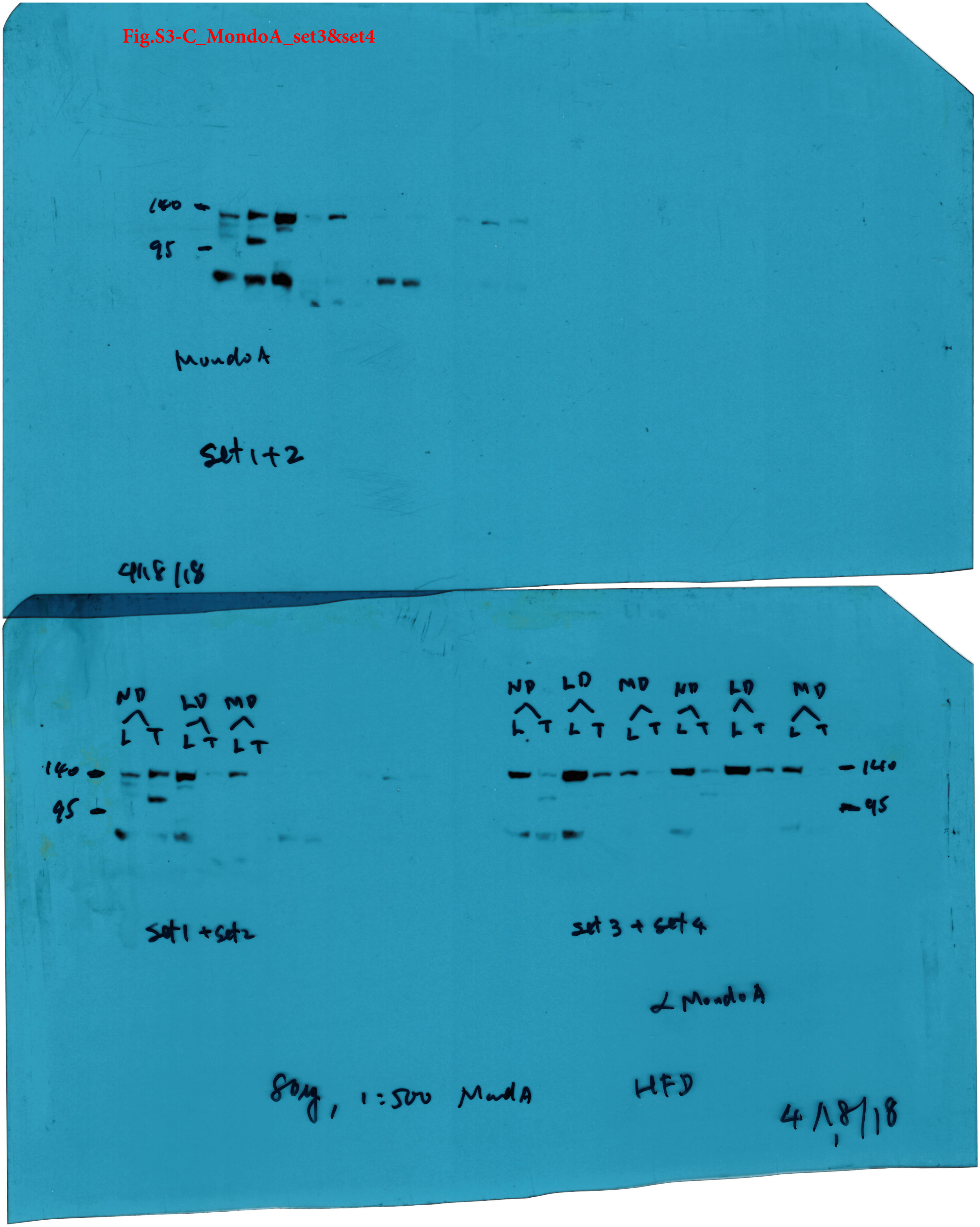

Fig.S3-C_N-Myc_set1&set2

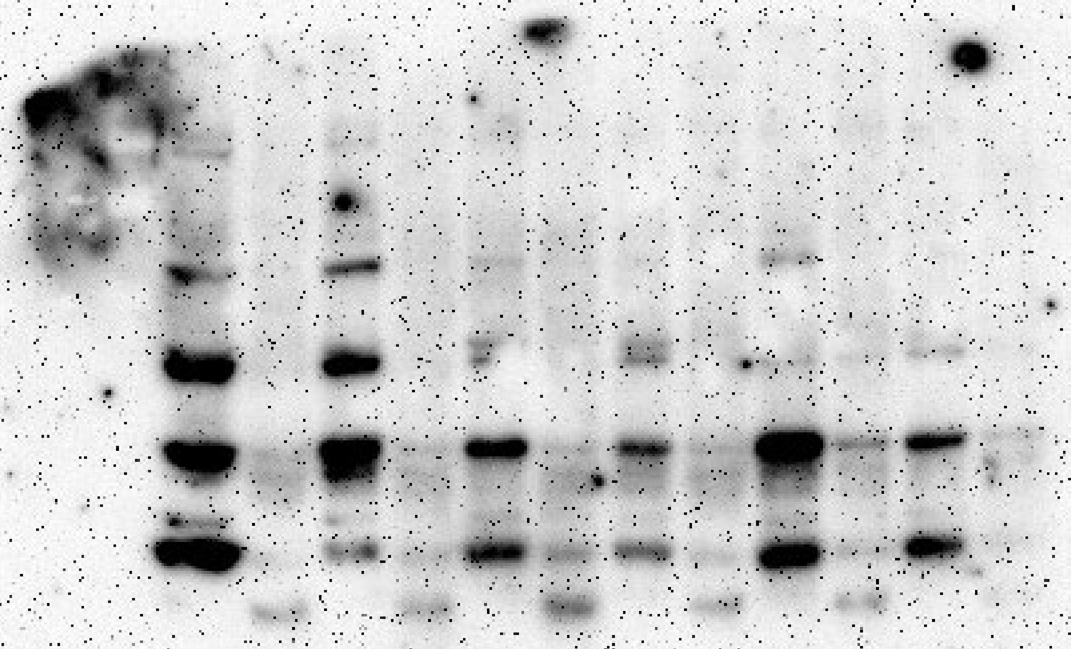

Fig.S3-C_N-Myc_set1&set2_marker

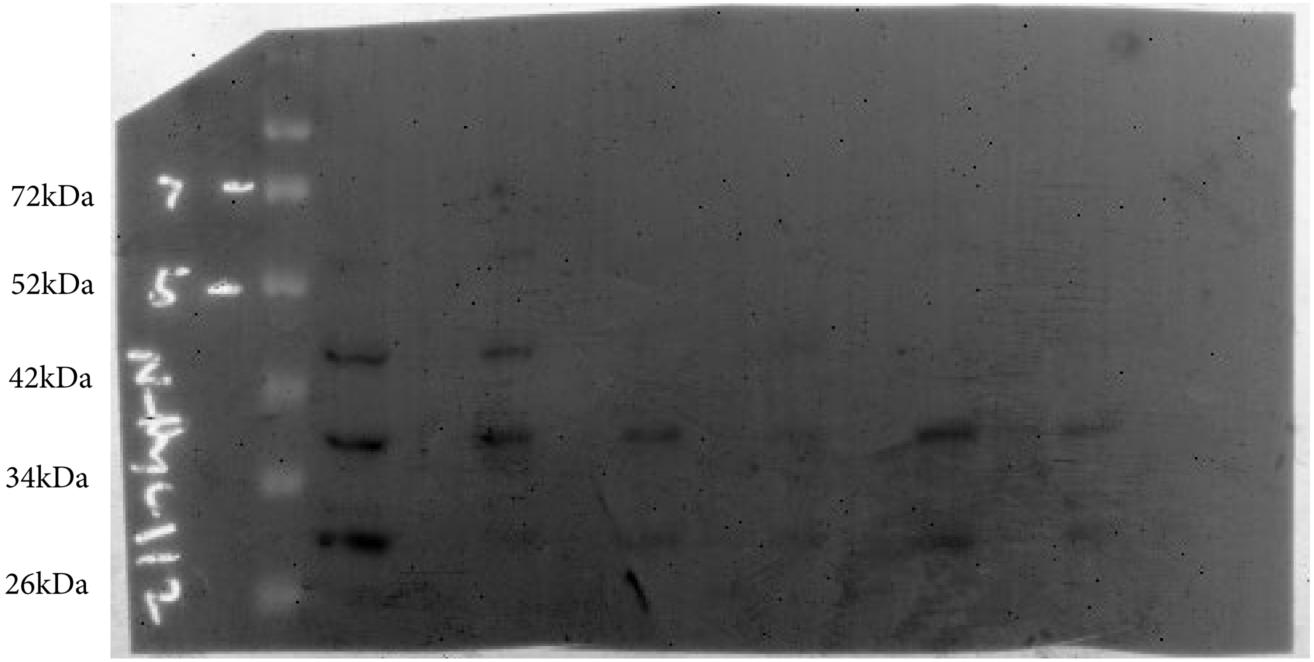

Fig.S3-C_N-Myc_set3&set4

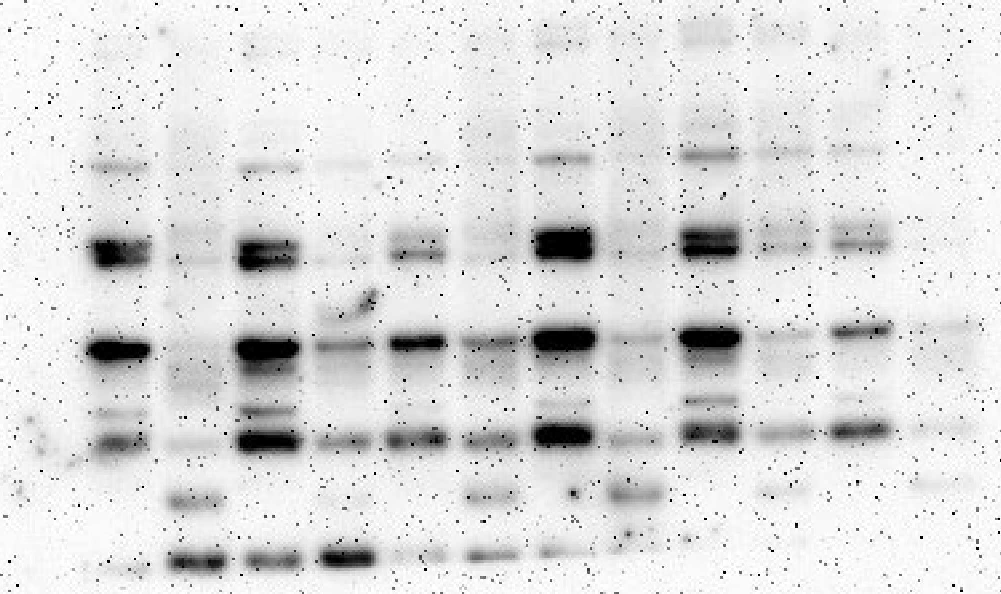

Fig.S3-C_N-Myc_set3&set4 with Marker

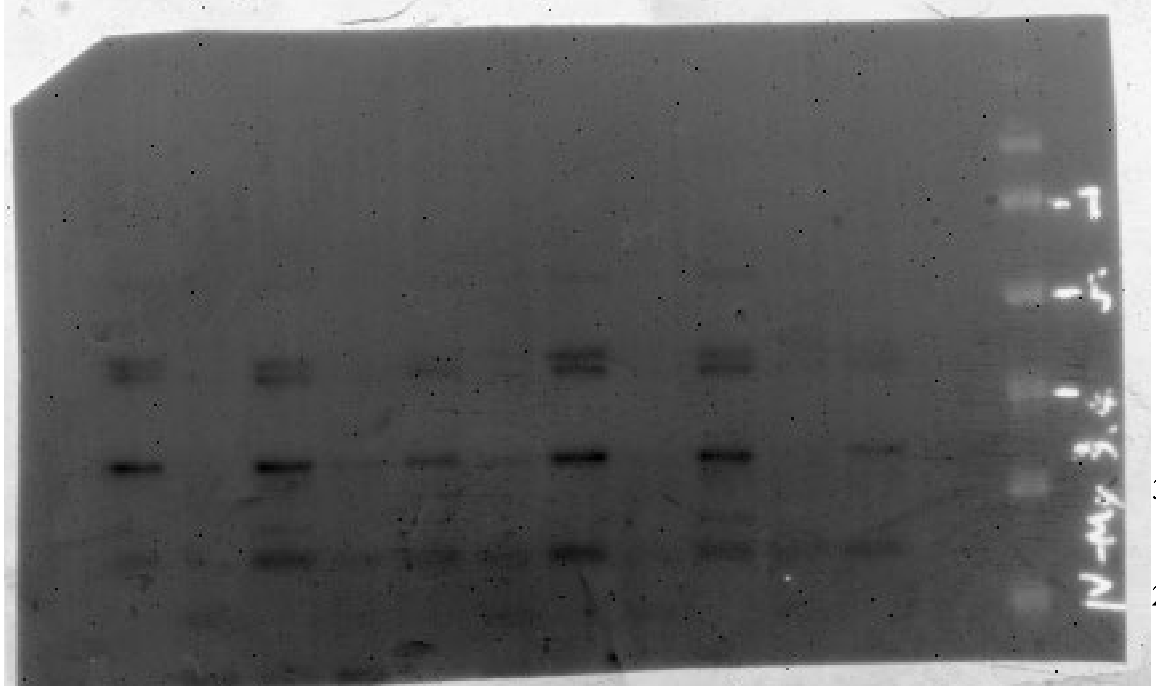

Supplement: S1 File — (PDF) [file pone.0218186.s016.pdf]
